# Development and Application of a Test for Food-Induced Emotions

**DOI:** 10.1371/journal.pone.0165991

**Published:** 2016-11-18

**Authors:** Uwe Geier, Arndt Büssing, Pamela Kruse, Ramona Greiner, Kirsten Buchecker

**Affiliations:** 1 Forschungsring e.V., Brandschneise 5, 64295, Darmstadt, Germany; 2 Department of Human Medicine, University of Witten/Herdecke, Gerhard-Kienle-Weg 4, 58313, Herdecke, Germany; 3 Technologie-Transfer-Zentrum (ttz) Bremerhaven, Fischkai 1, 27572, Bremerhaven, Germany; IRCCS Istituto Auxologico Italiano, ITALY

## Abstract

This study aimed to develop a test to measure food-induced emotions suitable for stable food and beverages. All of the experiments were conducted under the conditions of a consumer sensory evaluation according to German standard DIN 10974. Test development included descriptors’ derivation and factor analysis as well as a comparison between the new test (empathic food test, EFT) and a hedonic sensory test and an unspecific psychological test, known as a multidimensional mood questionnaire (MDMQ). Nineteen sensory experts derived twelve items using free-choice profiling. After an exploratory factor analyses, ten of the intended twelve items were integrated into two scales. To compare the new questionnaire (EFT) to the MDMQ and a hedonic test, panels of 59 (EFT), 64 (MDMQ) and 63 (hedonic sensory test) untrained individuals described their perceptions after consuming sensorially similar pairs of milk, water, bread and sugar. The benchmark of comparison was the power to discriminate between the food pairs. Test-retest replicability was demonstrated. All three tests presented slight differences in sample preference and effect size depending on the offered products. These findings underscore the need to test new methods with a wide range of products. Further research is needed to investigate the relationship between sensorial perception and emotional response.

## Introduction

Does food influence people’s emotions? In recent years, numerous studies have investigated food-induced emotions, leading to the development and application of different questionnaires focusing on emotional perceptions [1–4]. Current scholarly literature on ‘emotion questionnaires’ focuses on several topics, including the following: product specificity, questionnaire length, language [1], derivation of terms [5], the nationality of the observers, the frequency of consumption for certain products [6], the number of offered products, the order of questions [2], the temporal dynamic of sensorial and emotional effects [7], natural or laboratory settings [8] and the measurement period itself [9]. A current overview is given by Meiselman [10] and Köster [11].

The method developed in this study, the empathic food test (EFT), was designed suitable for stable foods and beverages. Other test methods focus primarily on highly-processed products and often concentrate on only one product [1,7,12,13].

In current methods measuring food-induced emotions, preparatory settings are neither integrated nor applied in sensory analyses. Therefore, a preparatory setting was developed as an element of the EFT, based on the concept of “mindfulness-based stress reduction” theorised by Kabat-Zinn [14]. The study included a preparatory setting because emotions are represented in the somatosensory system [15]. Furthermore, it was assumed that an improvement in body perception via the preparatory setting would lead to an improved capability to perceive the emotions caused by food.

Nineteen sensory experts were chosen to help develop items for the EFT questionnaire based on their training in the sensory perception of food [16]. However, previous studies have presented multiple possibilities for groups creating items for questionnaires regarding food-induced emotions. Examples include large groups of consumers via the internet [5,12], preselection by a small group of experts [17], groups of university students and employees [13,18], company employees [4] and the general population [6]. The product categories used for item derivation and EFT validation were identical.

While food-induced emotions are a psychological phenomenon [19], they are obviously effected by sensorial properties. Due to this division in two research fields, the EFT was compared to two other tests, one from each field. From the field of psychology, the unspecific German psychological test, the multidimensional mood questionnaire (MDMQ) [20,21], was chosen as a reference method. Olabi et al. [22] also used an unspecific psychological test called the profile of mood states [23] for the evaluation of food-induced emotions. As a second reference method, a hedonic test was chosen as it represents liking based on the products’ sensorial characteristics [24]. This test assists in characterizing the relationship between emotional responses and sensorial-based liking.

The current study was divided into two parts. In the first part, sensory experts generated free description items for four stable foods and beverages for the new questionnaire, the empathic food test (EFT). The EFT was based partially on a consumer sensory evaluation, according to German standard DIN 10974 [25] and Geier et al. [26]. In the second part, the EFT was validated by comparing the new test to established tests. The power to discriminate sensorially similar food pairs served as the benchmark for comparing the three applied tests. Thus, similar food pairs were chosen of milk, water, bread and sugar. Three tests were applied in parallel with independent panels. All three tests were conducted with individuals possessing the same level of knowledge—meaning untrained consumers—to ensure comparability of the results.

Test development led to the following research questions: Is the power of discrimination for the EFT higher than that of the established MDMQ? Do results of the emotional impression (EFT) and sensorial-based liking (hedonic test) tests correspond? Does the product influence the comparison of the three methods?

## Materials and Methods

### Experimental design

The three different approaches—the EFT, MDMQ and hedonic test—were compared to determine their power of discrimination by applying them to four similar coded pairs of food and beverage samples. Three consumer panels were formed, one for the hedonic test, one for the empathic food test (EFT) and one for the multidimensional mood questionnaire (MDMQ). Each panel experienced only one method to avoid learning effects. The benchmark between the three methods was the power to discriminate sensorially similar food pairs of milk, water, bread and sugar. Most items/evaluated characteristics of the three applied tests showed a positive or negative connotation, such as relaxed vs. restless (MDMQ), fresh vs. exhausted (EFT) and excellent taste vs. very bad taste (hedonic test). This connotation was the basis for the evaluation of differences in preferences.

All tests were carried out in the sensory laboratory of the ttz-Bremerhaven between November 2012 and January 2013. The tests were conducted under the conditions of the consumer sensory evaluation test according to German standard DIN 10974 [25].

Two food pairs were investigated per day. By performing a replication, the test-retest-replicability was guaranteed. The experiment sequence was as follows: All three panels (for each method) met four times. On the first date, milk and water were conducted, while bread and sugar occurred on the second date. The third date (milk and water) and the fourth date (bread and sugar) were replications of the first and second dates.

### Participant recruitment

The participants (volunteers older than 18 years) were recruited by the recruitment staff of the sensory laboratory ttz Bremerhaven via telephone. The recruitment staff used the ttz database as they are the only actors in the study who had access to participants’ identifying and personal information. People in the ttz database take part in hedonic sensory tests regularly. All participants received financial compensation and provided consent to participate in the study through verbal agreement on the telephone and by coming to the laboratory. Consent was documented by the conducting laboratory.

To ensure the test persons’ demands, the telephone interview verified the consumer consumption habits, frequency of consumption, familiarity with the products, and social demographic data. Furthermore they were asked in the telephone survey about their demands, possible refusals and allergies to the four products. Further questions were not asked concerning health and other medical issues. Participants received information about the study’s goals and the structure. All information about the participants was anonymized by ttz recruitment staff, and none of the authors had access to participants’ identifying information. Therefore, all data was completely anonymous and used solely for research purposes.

The food samples consisted of stable food and beverages. The food testing included the uptake of a small amount of stable food or beverage and a response given on a questionnaire. No harmful or unproved food, beverages or ingredients were served. Neither clinical tests nor behavioural tests took place within the study.

All experiments were carried out by professional staff of the sensory laboratory of the ttz Bremerhaven.

In sensory consumer testing with adult volunteers, it is uncommon to address ethical issues; therefore, the current study was not reviewed and approved by an institutional review board or ethics committee. However, the study was conducted in accordance with the ethics guidelines of the German Psychological Society [27], the ethics guidelines of the Institute of Food Science & Technology [28] and the ethical principles of the Market Research Society [29].

Finally, three consumer test panels were formed: one panel with 64 panellists for the MDMQ, one panel with 59 panellists for the EFT and one panel with and 63 panellists for the hedonic test. The number of participants in each panel is based on ISO 11136 [24].

The gender distribution for the three panels was about 38% male and 62% female (MDMQ), 38% male and 62% female (EFT) and 40% male and 60% female (hedonic sensory test). The age range was categorised in five age groups. The MDMQ panel had the following age distribution: 15.9% were between 20 and 30 years, 9.5% were between 31 and 40 years, 42.9% were between 41 and 50 years, 20.6% were between 51 and 60, and 11.1% were between 61 and 70 years old. 13.6% of the EFT panel was between 20 and 30 years, 20.3% were between 31 and 40 years, 33.9% were between 41 and 50 years, 23.7% were between 51 and 60 years, and 8.5% were between 61 and 70 years old. 17.5% of the hedonic sensory test was composed of 20-to-30-year-old panellists, 22.3% were between 31 and 40 years, 31.7% were between 41 and 50 years, 20.6% were between 51 and 60 years, and 7.9% were between 61 and 70 years old.

To guarantee that there were no major differences between the three panels, a chi^2^ test was conducted. The chi^2^ test found no correlations between the three panels regarding the characteristic features of age, sex, education level and application of stress reduction methods, meaning that the groups were comparable.

### Procedure

All tests were carried out in the sensory laboratory of the ttz-Bremerhaven, which is equipped according to ISO 8599 [30]. The laboratory is featured with 10 test booths and pass-through from each booth to the preparation room on the other side. The test persons are unable to see the test products before testing, guaranteeing that they are not influenced by information about the products. The test assistant served the product pairs in identical product conditions: means, temperature, dishes, portion sizes, and test-booth conditions.

### Food samples

Coded product pairs of water, milk, bread and sugar were served to the panels. The samples were selected to be sensorially similar and standardized while covering a wide range of stable foods and beverages (Table 1). It was important that there were few differences between the food pairs. As described by Hendy [9] and Bryan et al. [31], to prevent disturbance, the measurements were conducted directly after consumption of each food sample and not after a longer period.

**Table 1:** Applied food samples for the MDMQ, EFT and hedonic test panels.

	Water	Milk	Bread	Sugar
**Product specification**	Natural mineral water, (*Adelholzener)*	Cow milk, 3.8% fat content	Bread baked with full flour from wheat, same recipe	White, crystal sugar
**Product A**	1.5 l plastic bottle (served in a drinking glass)	Conventional, ESL *(Landliebe)*	Organic flour from Naturastar wheat variety	From sugar beet, Germany *(Südzucker)*
**Product B**	0.75 l glass bottle (served in a drinking glass)	Organic, pasteurized, non-homogenized (*Söbbeke)*	Organic flour from Goldblume wheat variety	From sugar cane, Brazil (*Guarani*)
**Assumed discriminating factor**	Bottle/package	Origin and processing of milk	Wheat variety	Crop

### Applied methods

#### Multidimensional mood questionnaire (MDMQ)

The MDMQ [20,21] is a psychological test in German for therapy evaluation and applied psychological research. The MDMQ uses 24 (long form) or 12 (short form) items to cover three bipolar dimensions of mood (scales), i.e., good mood—bad mood (scale 1), alertness—fatigue (scale 2), and ease—unease (scale 3). A short form of the MDMQ was chosen in order to apply a comparable number of items in both psychological tests, namely the MDMQ and EFT. There are two versions of the MDMQ short form, type A and B. As mentioned above, both short forms cover the three scales, and only a slight difference appears between the 12 items. Because this minor difference has no impact on the results, we decided to use type A (Table 2). Participants measured all items following five steps where one indicates “not at all” and five means “very much”.

**Table 2:** Elements of the MDMQ test (short form A).

	English	German	Scale
**I feel at the moment…**	good	Gut	1
	bad	schlecht	1
	content	zufrieden	1
	unwell	unwohl	1
	alert	munter	2
	tired	müde	2
	rested	ausgeruht	2
	flabby	schlapp	2
	relaxed	entspannt	3
	restless	ruhelos	3
	calm	gelassen	3
	agitated	unruhig	3

#### Empathic food test (EFT)

Analogue to an element of Kabat-Zinn’s concept of mindfulness-based stress reduction [14,32], participants in the empathetic food test (EFT) carried out a short body scan to calm down before observing food effects. The participants were guided to be attentive to certain body parts for a few seconds, starting with the forehead, followed by the nose, chin, shoulders, arms, hands, hip bone, top side of the thigh, knee, shin, heel, foot, toes, sole of the foot, calves, hollow of the knee, bottom side of the thigh, the buttocks, the back, the neck, and the back of the head to the vertex. The body scan took approximately 3 minutes.

The EFT questionnaire includes 12 bipolar attributes (Table 3), rated in five degrees: “warm”, “rather warm”, “neutral”, “rather cold” and “cold”. The following paragraph will outline how the items for the EFT questionnaire were developed and selected.

**Table 3:** The 12 bipolar items of the EFT questionnaire in English and German, divided into three categories.

	English		German	
**My body feels…**	warm	cold	warm	kalt
	bright	dark	hell	dunkel
	light	heavy	leicht	schwer
	fresh	exhausted	erfrischt	matt
**I feel…**	energized	not energized	energetisiert	nicht energetisiert
	awake	tired	wach	müde
	concentrated	not concentrated	konzentriert	unkonzentriert
	relaxed	nervous	entspannt	nervös
	comforting	unwell	wohlig	unwohl
	satisfied	unsatisfied	zufrieden	unzufrieden
	balanced	unbalanced	ausgewogen	unausgewogen
**The impact feels…**	long lasting	short	lang anhaltend	kurz anhaltend

To conduct the terms for the EFT test, a panel of 19 sensory experts untrained in psychological terms developed attributes to describe participants’ behaviour after consumption of food samples in accordance with the sensory descriptive method and the free choice profiling (ISO 13299, [16)]). Sensory experts were chosen for test development due to their training in food characterisation. To develop the EFT questionnaire for foods and beverages, four different products were offered: water, milk, bread and sugar. The 19 sensory experts received the products semi-monadicly and developed new items to describe people’s mood after consuming (swallowing) the food and beverage samples. During analyses, phrase items with the same meanings were combined into one item based on consensus. Finally, the items were assessed according to the frequency of nomination per product (Table 4). Items were excluded if they were limited to single body parts, such as stomach or head.

**Table 4:** Effects of coded food samples on physical and mental states. Free description of 19 sensory experts, depicting the most frequently-used terms; the table also provides the frequency of nomination.

Water	Milk	Bread	Sugar
*Natural mineral water*, *Adelholzener*, *1*.*5 L plastic bottle*	*Cow’s milk*, *3*.*8% fat content*, *Conventional*, *ESL*, *(Landliebe)*	*Bread baked with full flour from organic wheat*, *variety Naturastar*	*White*, *crystal sugar from sugar beet*, *Germany (Südzucker)*
refreshed, alive (18)	relaxation, rest (9)	positive mood (12)	positive mood (10)
alert (11)	warmth (7)	exhausting (9)	alert (8)
positive mood (9)	positive mood (5)	warmth (7)	energized (7)
	invigorating, bracing (4)	invigorating (5)	darkness (2)
			heaviness (2)

#### Hedonic test

The hedonic test was carried out with untrained consumers according to German standard DIN 10974 [25]. Participants were asked to evaluate the samples’ appearance, taste, smell, texture (consistency) and general impression by rating them from very bad (1) to excellent (7). The products were served semi-monadic per product group in a randomized order. In between two samples, the participants neutralized with water or toast.

### Statistical analysis

#### Factor analysis of the EFT items

An exploratory factor analyses was conducted [33] to determine the dimensionality of the set of 12 EFT items using the SPSS FACTOR routine. Data came from the experimental application of the EFT considering 8 experiments (4 products with 2 replications). Factor analysis is a statistical technique commonly applied to the construction and evaluation of questionnaires and psychological measurements. Based on the assumption that correlations between a set of items can be explained by a few underlying latent factors, this technique allows for the assessment of the latent dimensionality of a set of items. A Cronbach’s alpha analysis also was conducted to prove the reliability of each factor scale.

#### Analysis of the variance for the EFT, MDMQ and hedonic test

An analysis of the variance was conducted (ANOVAs; [34]) to compare between panels’ ratings of each food pair and between the two replications of the assessment. In general, ANOVA partitions the overall variation in the data into variance due to experimental factors, such as food type and error variance. This procedure allowed testing of the statistical significance rating differences between each pair of foods and beverages as well as between the two replications and possible interactions between these two factors. Each analysis thus yields three results: the two main effects (product type and replication) and their interaction. The main effect of the product is of central interest here because it indicates whether the ratings for the two products differ significantly. The main effect of the replication indicates whether or not there are statistically reliable differences in the ratings between both replications. Last, a significant interaction indicates whether or not the difference in ratings between both products differs between the two replications.

#### Descriptive statistics and Cohen’s d-value for the EFT, MDMQ and hedonic tests

The analysis variance was supplemented by descriptive statistics (the mean value and the standard deviation (SD)). On this basis, the Cohen’s d-value was calculated to compare the results of the hedonic test, EFT and MDMQ. The Cohen’s d-value was used as a measure of the effect size to compare the discriminating power of the three methods. A d-value of 0.2 indicated a small effect, a d-value of 0.5 showed a medium effect, and a d-value of 0.8 indicated a large effect [35]. All data analyses were carried out with SPSS.

## Results

### Results of the factor analysis (test development)

The average correlations between the 12 items of the EFT varied between 0.40 and 0.87. Due to these substantial correlations, a factor analysis approach was chosen to extract the underlying factor structure and to construct coherent measurement scales from the items. For this purpose, factor analyses were conducted separately for the responses of the participants regarding each of the eight different products on the 12 EFT items. These analyses employed the SPSS FACTOR routine. A two-dimensional factor solution excluding item 1 (“warm—cold”) and item 12 (“long-lasting—short”) provided the best approximation of a simple structure and the most consistent solution across all products. Significantly, items 1 and 12 showed no substantial loadings on any factor for most products and were therefore omitted from scale construction. The number of factors to be extracted was based on the criterion that their eigenvalues would be larger than 1. Thus, two factors with eigenvalues larger than 1 were extracted, and a varimax rotation was performed on the extracted factors. After the exclusion of items 1 and 12, only items 2, 4 and 9 produced cross loadings larger than or equal to 0.45. The variance among the items explained by the two-factor solution ranged between 57% for the product “water from a plastic bottle” and 71% for the product “bread Naturastar”. For the sake of concision, Table 5 presents the rotated factor solution and variance only for the data aggregated across all 8 products.

**Table 5:** Factor loadings and percent of variance explained for principle component extraction and varimax rotation on the aggregated data of the 12 bipolar items of the EFT.

Item		Factor 1	Factor 2	Scale
01: warm	cold	-	-	-
02: bright	dark	0.56	0.47	1
03: light	heavy	-	0.66	2
04: fresh	exhausted	0.51	0.68	2
05: energized	not energized	-	0.77	2
06: awake	tired	-	0.89	2
07: concentrated	not concentrated	-	0.76	2
08: relaxed	nervous	0.83	-	1
09: comforting	unwell	0.79	0.45	1
10: satisfied	unsatisfied	0.87	-	1
11: balance	unbalanced	0.90	-	1
12: long lasting	short	-	-	-
**Percent variance explained**		**39.51**	**35.97**	

Note: Loadings smaller than 0.45 are not depicted

According to the results of the factor analysis (Table 5), items 2, 8, 9, 10 and 11 were summarized in scale 1, while items 3, 4, 5, 6 and 7 were aggregated into scale 2.

Scale 1 can be interpreted as reflecting general (bodily) wellbeing, whereas the second scale can be interpreted as concentration or mindfulness. Item 1 (warm—cold) and item 12 (long—short lasting) remained separate. Low values (close to one) show a positive connotation, and high values (close to 5) show a negative connotation for scales 1 and 2 as well as for item 1. The values for item 12 were neutral; therefore, neither held a positive or negative connotation.

Furthermore, separate analyses of the reliability of each scale indicated very high internal consistency for each of the scales. Cronbach’s alpha, computed on the aggregated data of all 8 products, was 0.93 for scale 1 and 0.90 for scale 2. Thus, the present two-factor solution represents an adequate description of the EFT’s factor structure.

For the sake of brevity, the results of the conducted tests are summarized in tables, and only important results receive commentary. The main product effects and interactions are important in the following presentation of results.

### Results of the MDMQ ratings (test application)

In discussing significant MDMQ results, explanations are provided for the descriptive statistics. The single items were summarised in three bipolar scales (1^st^: good mood—bad mood, 2^nd^: alertness—fatigue and 3^rd^: ease—unease) (Table 2). High values (close to 5) held positive connotations, while values close to 1 had negative connotations.

#### Water

No significant effects were found on the MDMQ for water from a plastic compared to a glass bottle (Table 6).

**Table 6:** Analyses of variance from the MDMQ ratings of water, milk, bread and sugar samples, significant values (< 0.05) are in bold. (S1 Dataset and S1–S4 Files).

		Main effect: replication	Main effect: product	Interaction
**Water**	Scale 1	*p* = 0.83	*p* = 0.22	*p* = 0.57
	Scale 2	*p* = 0.18	*p* = 0.29	*p* = 0.96
	Scale 3	*p* = 0.30	*p* = 0.67	*p* = 0.84
**Milk**	Scale 1	***p* < 0.01**	***p* = 0.03**	*p* = 0.12
	Scale 2	***p* < 0.001**	*p* = 0.20	*p* = 0.29
	Scale 3	*p* = 0.41	*p* = 0.50	*p* = 0.22
**Bread**	Scale 1	*p* = 0.22	*p* = 0.94	*p* = 0.58
	Scale 2	*p* = 0.92	*p* = 0.09	*p* = 0.57
	Scale 3	*p* = 0.90	*p* = 0.87	*p* = 0.91
**Sugar**	Scale 1	*p* = 0.22	*p* = 0.30	*p* = 0.35
	Scale 2	*p* = 0.64	*p* = 0.66	*p* = 0.52
	Scale 3	*p* = 0.62	*p* = 0.81	*p* = 0.28

#### Milk

Scale 1 indicates some significant effects (Table 6). On scale 1, organic milk was rated consistently more positively [1^st^ mean 2.70 (SD 0.36); 2^nd^ mean 2.55 (SD 0.71)] than conventional milk [1^st^ mean 2.66 (SD 0.30); 2^nd^ mean 2.28 (SD 1.03)]. Both products received lower ratings on the second replication (2^nd^ mean).

#### Bread

There were no statistically significant differences for the bread pair (Table 6). Scale 2 shows a trend towards better ratings for Goldblume bread [1st mean 2.61 (SD 0.57); 2nd mean 2.63 (SD 0.80)] compared to Naturastar bread [1st mean 2.59 (SD 0.52); 2nd mean 2.58 (SD 0.80)].

#### Sugar

The analysis of variance shows no significant differences for the sugar pair (Table 6).

### Results of the EFT questionnaire rating (test application)

As outlined earlier, 10 of the 12 EFT items were summarized in terms of two scales. The remaining two items were analysed separately; therefore, discussing the scales is more meaningful than referring to single items. The significant results are discussed below and supplemented with observations on the descriptive statistics. A low mean value (close to 1) implies a positive connotation, while a high mean value (close to 5) indicates a negative connotation.

#### Water

Scale 1 and item 12 show significant effects in terms of the main effect of the product. The interaction is significant for item 12 (Table 7). For both replications on scale 1, water from a plastic bottle was rated more positively [1^st^ mean 2.13 (SD 0.64); 2^nd^ mean 2.13 (SD 0.67)] than water from a glass bottle [1^st^ mean 2.34 (SD 0.79); 2^nd^ mean 2.37 (SD 0.87)]. By trend, plastic [1^st^ mean 1.97 (SD 0.69); 2^nd^ mean 2.05 (SD 0.67)] was rated better than glass [1^st^ mean 2.10 (SD 0.68); 2^nd^ mean 2.21 (SD 0.77)] on scale 2. The effect of plastic was rated as longer lasting than glass on the first replication but similarly long lasting on the second replication.

**Table 7:** Analyses of variance from EFT ratings of water, milk, bread and sugar samples, significant values (< 0.05) are in bold. (S2 Dataset and S5–S8 Files).

		Main effect: replication	Main effect: product	Interaction
**Water**	Scale 1	*p* = 0.87	***p* = 0.01**	*p* = 0.85
	Scale 2	*p* = 0.14	*p* = 0.06	*p* = 0.84
	Item 1	*p* = 0.53	*p* = 0.15	*p* = 0.16
	Item 12	*p* = 0.43	***p* = 0.04**	***p* < 0.01**
**Milk**	Scale 1	*p* = 0.70	*p* = 0.49	*p* = 0.71
	Scale 2	*p* = 0.09	***p* = 0.02**	*p* = 0.81
	Item 1	*p* = 0.53	*p* = 0.23	***p* < 0.01**
	Item 12	*p* = 0.06	*p* = 0.37	*p* = 0.94
**Bread**	Scale 1	***p* = 0.02**	*p* = 0.30	*p* = 0.53
	Scale 2	***p* < 0.01**	*p* = 0.50	*p* = 0.44
	Item 1	*p* = 0.06	*p* = 0.83	*p* = 0.06
	Item 12	*p* = 0.80	*p* = 0.77	*p* = 0.43
**Sugar**	Scale 1	*p* = 0.80	*p* = 0.06	*p* = 0.58
	Scale 2	***p* < 0.05**	*p* = 0.09	*p* = 0.65
	Item 1	*p* = 0.16	*p* = 0.11	*p* = 0.52
	Item 12	*p* = 0.76	*p* = 0.83	*p* = 0.19

#### Milk

There were significant differences for the main product effect of scale 2 and a significant interaction for item 1 (Table 7). On scale 2, organic milk was rated more positively [1^st^ mean 1.90 (SD 0.69); 2^nd^ mean 2.01 (SD 0.66)] than conventional milk [1^st^ mean 2.12 (SD 0.80); 2^nd^ mean 2.20 (SD 0.77)]. The pattern for item 1 is inconsistent. Organic milk was rated more positively [mean 2.53 (SD 1.29)] than conventional milk [mean 3.00 (SD 1.40)] on the first replication, but this pattern flipped on the second replication [org. mean 2.75 (SD 1.35); conv. mean 2.61 (SD 1.22)].

#### Bread

For bread, no significant effects were found for the main product effect or interaction (Table 7).

#### Sugar

There were no statistically significant differences between sugar beet and sugar cane (Table 7), but scale 1 shows by trend that sugar cane [1^st^ mean 2.02 (SD 0.76); 2^nd^ mean 1.96 (SD 0.80)] is rated consistently more positively than sugar beet [1^st^ mean 2.17 (SD 0.78); 2^nd^ mean 2.20 (SD 0.87)] As well scale 2 shows by trend that sugar cane [1^st^ mean 2.04 (SD 0.83); 2^nd^ mean 1.86 (SD 0.68)] is rated more positively compared to sugar beet [1^st^ mean 2.17 (SD 0.76); 2^nd^ mean 2.05 (SD 0.82)].

### Results of the hedonic test rating (test application)

Significant results from the analysis of variance (Table 8) are discussed below, and descriptive statistics are complemented by observations. A high mean (close to 7) indicates excellent, while a low mean (close to 1) implies a bad evaluation of the attribute.

**Table 8:** Analyses of variance from hedonic ratings of water, milk, bread and sugar samples, significant values (< 0.05) are in bold. (S3 Dataset and S9–S12 Files).

		Main effect: Replication	Main effect: product	Interaction
**Water**	Appearance	***p* < 0.01**	*p* = 0.54	***p* = 0.01**
	Smell	*p* = 0.37	*p* = 0.26	*p* = 0.21
	Taste	*p* = 0.72	*p* = 0.35	*p* = 0.67
	Texture	*p* = 0.94	*p* = 0.72	*p* = 0.84
	General impression	*p* = 0.87	*p* = 0.46	*p* = 0.46
**Milk**	Appearance	***p* = 0.04**	***p* < 0.001**	*p* = 0.09
	Smell	***p* < 0.001**	*p* = 0.40	***p* < 0.001**
	Taste	***p* < 0.001**	***p* < 0.001**	***p* < 0.001**
	Texture	***p* < 0.001**	***p* = 0.03**	***p* < 0.001**
	General impression	***p* < 0.001**	***p* = 0.01**	***p* < 0.001**
**Bread**	Appearance	*p* = 0.89	***p* < 0.001**	***p* < 0.001**
	Smell	***p* < 0.01**	***p* < 0.01**	*p* = 0.88
	Taste	*p* < 0.87	*p* = 0.08	***p* = 0.04**
	Texture	*p* = 0.82	*p* = 0.22	*p* = 0.16
	General impression	*p* = 1.00	***p* < 0.01**	*p* = 0.29
**Sugar**	Appearance	*p* = 0.21	***p* < 0.001**	*p* = 0.50
	Smell	*p* = 0.10	***p* < 0.001**	*p* = 0.26
	Taste	*p* = 0 .11	***p* < 0.01**	*p* = 0.22
	Texture	*p* = 1.00	***p* = 0.03**	*p* = 0.55
	General impression	*p* = 0.92	***p* < 0.001**	*p* = 0.50

#### Water

No product differences were observed when comparing water from a glass to that of a plastic bottle (Table 8). The interaction for appearance showed a significant difference between the first and second dates, according to the analysis of variance (Table 8). Appearance was rated similarly in the first replication [glass: mean 5.63 (SD 1.15); plastic: mean 5.54 (SD 1.15)], but plastic was rated more positively than glass on the second replication [5.46 (SD 1.12)] [5.25 (SD 1.15)].

#### Milk

Significant product differences were found for all five liking items for the conventional milk (conv.) versus the organic milk (org.) (Table 8). The appearance of the conventional milk was rated more positively for both replications [1^st^ mean 5.59 (SD 1.19); 2^nd^ mean 5.25 (SD 1.05)] compared to the organic milk [1^st^ mean 4.95 (SD 1.24); 2^nd^ mean 4.92 (SD 1.15)]. The ratings for the smell varied between the two replications. The smell of conventional milk was rated more positively on the first replication [conv. mean 5.31, (SD 1.0); org. mean 5.05 (SD 0.98)], while the smell of organic milk was rated more positively on the second replication [conv. mean 4.52 (SD 1.28); org. mean 4.98 (SD 0.91)]. The same pattern was found with different mean and SD values for the taste, texture and general impression. If we consider taste, the statistics for first replication were [conv. mean 5.27 (SD 1.18); org. mean 5.11 (SD 1.06)]. At second replication, they were [conv. mean 3.87 (SD 1.44); org. mean 5.16 (SD 1.10)]. Next, considering texture: the first replication was [conv. mean 5.31 (SD 1.08); org. mean 5.23 (SD 1.05)], and the second replication was [conv. mean 4.53 (SD 1.18); org. mean 5.23 (SD 0.91)]. Finally, for the general impression, the first replication was [conv. mean 5.29 (SD 1.14); org. mean 5.05 (SD 0.97)], and the second replication was [conv. mean 4.13 (SD 1.34); org. mean 5.16 (SD 0.98)].

#### Bread

Significant differences were found for item appearance, smell and general impression when looking at the main product effect of the Naturstar bread (Nat.) versus the Goldblume bread (Gol.). The interaction shows significant results for taste and appearance (Table 8). The appearance was judged similarly on the first replication [Nat. mean 4.32 (SD 1.22); Gol. mean 4.33 (SD 1.22)], but for the second replication, the appearance of Naturastar was rated more positively [mean 4.57 (SD 1.15)] than Goldblume [mean 4.11 (SD 1.07)]. As for smell, Naturastar was rated more positively [1^st^ mean 4.92 (SD 1.06); 2^nd^ mean 4.63 (SD 1.09)] on both replications than Goldblume [1^st^ mean 4.71 (SD 1.09); 2^nd^ mean 4.45 (SD 0.9)]. The taste was rated similarly on the first replication [Nat. mean 4.16 (SD 1.07); Gol. mean 4.18 (SD 1.17)], but on the second replication, the taste of Naturastar [mean 4.34 (SD 1.10)] was rated more positively than Goldblume [mean 3.97 (SD 1.01)]. Regarding general impression, Naturastar [1^st^ mean 4.23 (SD 1.08); 2^nd^ mean 4.31 (SD 1.05)] was rated more positively than Goldblume [1^st^ mean 4.11 (SD 1.07); 2^nd^ mean 4.03 (SD 0.87)].

#### Sugar

The analysis of variance for sugar only showed significant differences for the main product effect (Table 8). Sugar from beet was consistently rated more positively than sugar cane for all five liking items. For appearance, the first replication was [beet mean 5.65 (SD 1.00); cane mean 4.33 (SD 1.3)], and the second replication was [beet mean 5.68 (SD 1.03); cane mean 4.48 (SD 1.22)]. For smell, the first replication was [beet mean 4.94 (SD 1.01); cane mean 4.05 (SD 1.26)], and the second replication was [beet mean 5.02 (SD 0.91); cane mean 4.32 (SD 1.23)]. For taste, the first replication was [beet mean 5.39 (SD 0.80); cane mean 4.85 (SD 1.33)], and the second replication was [beet mean 5.42 (SD 0.82); cane mean 5.10 (SD 1.07)]. For texture, the first replication was [beet mean 5.31 (SD 0.88); cane mean 4.92 (SD 1.23)], and the second replication was [beet mean 5.24 (SD 0.80); cane mean 4.98 (SD 1.12)]. Finally, for general impression, the first replication was [beet mean 5.40 (SD 0.69); cane mean 4.65 (1.15)], and the second replication was [beet mean 5.34 (SD 0.75); cane mean 4.69 (SD 1.08)]. appearance: first replication [beet mean 5.65 (SD 1.00); cane mean 4.33 (SD 1.3)] and second replication [beet mean 5.68 (SD 1.03); cane mean 4.48 (SD 1.22)], smell: first replication [beet mean 4.94 (SD 1.01); cane mean 4.05 (SD 1.26)] and second replication [beet mean 5.02 (SD 0.91); cane mean 4.32 (SD 1.23)], taste: first replication [beet mean 5.39 (SD 0.80); cane mean 4.85 (SD 1.33)] and second replication [beet mean 5.42 (SD 0.82); cane mean 5.10 (SD 1.07)], texture: first replication [beet mean 5.31 (SD 0.88); cane mean 4.92 (SD 1.23)] and second replication [beet mean 5.24 (SD 0.80); cane mean 4.98 (SD 1.12)] and general impression: first replication [beet mean 5.40 (SD 0.69); cane mean 4.65 (1.15)] and second replication [beet mean 5.34 (SD 0.75); cane mean 4.69 (SD 1.08)].

### Comparison of the MDMQ, EFT and hedonic test

**Fig 1:**
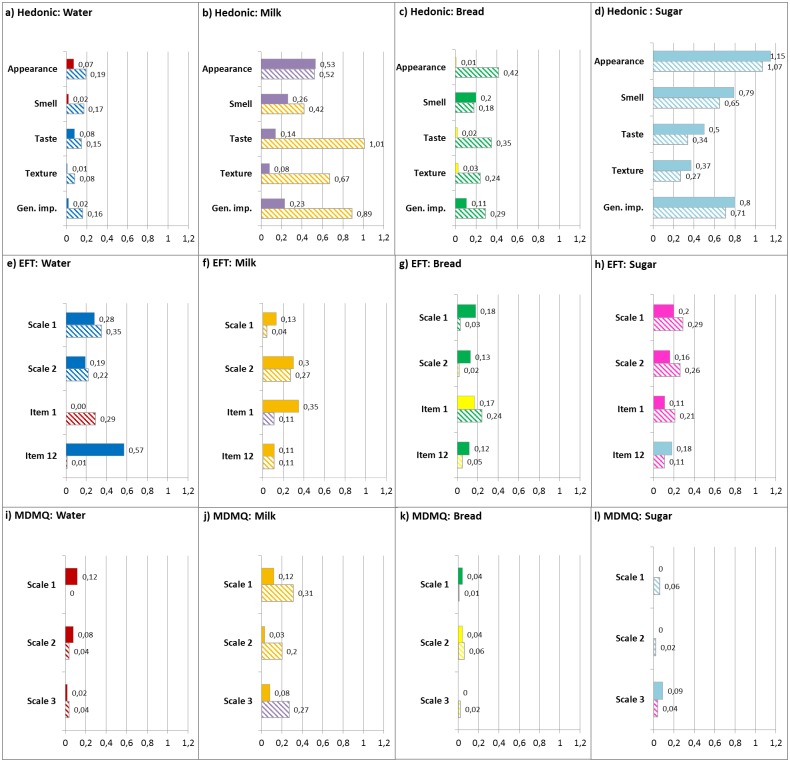
Overviews of Cohen’s d values. Overviews of Cohen’s d values (d = 0.2 small effect, d = 0.5 medium effect, d = 0.8 large effect) were completed for water, milk, bread and sugar for the three applied methods (MDMQ, EFT and hedonic tests). Colour represents the preference (positive connotation) for the products. Plain colours indicate the first occasion, and patterned colours indicate the second occasion.

With the exception of water, the hedonic test shows the highest effect size compared to the EFT and MDMQ (Fig 1). The effect size of the MDMQ is the smallest except for milk, for which the MDMQ and the EFT values are similar. The effect size of the EFT falls between the hedonic test and the MDMQ value with the mentioned exceptions (Fig 1).

Compared to the hedonic test, the EFT test for the water samples shows slightly larger differences to the majority of d-values above 0.2, whereas the d-values are all lower than 0.2. The MDMQ test revealed the smallest discrimination power for water; the d-values are very small. In the EFT, preference is shown for water from a plastic bottle; only item 1 shows a positive connotation for water from a glass bottle. (Fig 1a, 1d and 1i). Preference was not considered for d-values lower than 0.2, such as for the MDMQ and hedonic tests.

The milk samples were discriminated best by the hedonic test, but with a variation in replication. The d-values for replication one are mostly approximately 0.2, whereas the d-values for the second replication are between 0.4 and 1. The EFT and MDMQ test show similar d-values for the milk samples, with most of them lower than 0.3. In the hedonic test, the product ranking for the positive connotation switches from conventional milk on the first replication to organic milk on the second replication. Organic milk had a positive connotation in the EFT with one exception; the same is true for the MDMQ (Fig 1b, 1f and 1j).

For bread, the Cohen’s d-value for all methods is small. The d-values get progressively smaller in the hedonic test, followed by the EFT, and they are very small for the MDMQ. The majority of positive connotation is connected to Naturastar with two exceptions in the hedonic test and two in the EFT. The MDMQ values for bread are too low for comment (Fig 1c, 1g and 1k).

The hedonic test discriminates sugar samples with high or medium d-values, while d-values for the EFT are small or medium. The MDMQ test for sugar reveals very small d-values. The positive connotation falls to sugar beet in the hedonic test, whereas sugar cane is preferred in the EFT with one exception. The values for the MDMQ are too small to be considered (Fig 1d, 1h and 1l).

## Discussion

The explicit intention of the current study was to develop a test for food-induced emotions suitable for stable food and beverages. For validation, the EFT was compared to another emotion questionnaire (MDMQ). A hedonic test was conducted in parallel to assess liking based on the sensorial characteristics. The three guiding research questions were as follows: Is the power of discrimination of the EFT higher than that of the unspecific psychological test MDMQ? Do the results of the emotional impression (EFT) and sensorial-based liking (hedonic test) correspond? Does the product influence comparison of the three methods?

### Is the power of discrimination of the EFT higher than that of the MDMQ?

The product effect shown by the analysis of variance denotes a slightly higher discriminating power for the EFT for water and sugar (Table 7) compared to the MDMQ (Table 6). The product effect for milk and bread is similar for the MDMQ and EFT (Tables 6 and 7). The d-value of the EFT compared to the MDMQ is obviously higher, with the exception of milk, for which the d-values are similar (Fig 1). Nothing can be said about differences in preference due to the weak response of the MDMQ (Fig 1). It can be stated that the EFT has a slightly higher power of discrimination than the MDMQ. However, to prove the performance of the EFT, it could be compared to other questionnaires for food-induced emotions. The differences in discrimination between the EFT and MDMQ might be explained by the varying structure of the applied questionnaires (EFT and MDMQ). Another explanation for the higher discrimination power of the EFT is the effect of the body scan. As mentioned previously, the body scan is similar to the concept of mindfulness-based stress reduction, which was developed in a medicinal context [14,32]. Mindfulness stress reduction affects patients with chronic somatic diseases as well as healthy people. It has a small positive effect on a patient’s health status [36], and it impacts healthy people in terms of psychological variables, such as stress, depression, anxiety, distress and burnout [37].

If the positive effect found in the medicinal context is transferable to the higher discrimination power, the EFT remains open. Further research is needed to evaluate the potential influence of preparatory settings on the observation of food-induced emotions.

### Do the results of emotional impression (EFT) and sensorial-based liking (hedonic test) correspond?

The results of the EFT test and the hedonic test differ. The hedonic test shows more product effects for three of the four examined food pairs (Table 8). The d-values from the hedonic test are higher for all products, with the exception of water, where the d-value from the EFT is higher (Fig 1). The preference for water and bread is similar in both tests. Sugar shows an inverse preference while the preference is partly inverse for milk (Fig 1).

Few studies have been conducted comparing sensory perception, liking and emotional responses. Porcherot et al. [4] examined odour-induced emotions, discovering that measuring feelings improved the discrimination power of different products with similar liking scores. However, sensory and emotional parameters have not been studied in parallel. Jager et al. [7] compared the measurement of sensorial and emotional attributes in parallel over time using the example of dark chocolate. The emotional response occurred later and was generally harder to experience compared to sensorial parameters. Furthermore, a joint Canonical Variate Analysis plot (CVA plot) on the duration of dominance for sensory and emotional attributes revealed that the temporal evolution of most sensory and emotional attributes was related, but some attributes seemed to be independent [7]. Mojet [38] and Gutjar [39] showed that liking and emotion measurements are only partly associated. They discuss that emotion measurements can provide additional information compared to liking.

The temporal dynamic of perception might be one reason for the partly inverse results for the EFT and hedonic test. The current results also show that hedonic and emotional attributes are not always in accordance. Dürrschmid [19] noted that only a fraction of sensory perception finds its way into our consciousness, and therefore, only this fraction will be available in a classic standardised sensory test. The EFT focuses on emotion while the hedonic test concentrates on sensorial-based liking. A change in awareness might explain the different results on the EFT and hedonic test. Furthermore, such a change could explain why the preferences for a product can even be inverse, as is the case for sugar.

### Does the product influence comparison of the three methods?

The product partly influences the discrimination of the three applied tests. Three out of four products influence the comparison of the EFT and MDMQ (Fig 1). Two (sugar and water) out of four products indicate an influence due to the product when comparing the EFT and hedonic tests. Many studies investigating food emotion questionnaires focus only on one product [1,7,40]. Porcherot et al. [4] as well as King and Meiselman [12] also found product influence regarding acceptability and emotion measurement, highlighting the need to prove new methods with a wide range of food products.

The four products chosen for this study come from different product categories and are quite different. The EFT method has to be proven with more products to demonstrate its applicability to a wide range of products in the future.

The emotion questionnaires and hedonic test do not lead to the same results. Further research is needed to gain a better understanding of the relationship between sensorial perception, liking and emotional response. The current study applied a hedonic test based on sensorial liking rather than a descriptive sensory test due to its focus on consumers. Future studies should include sensory experts to obtain a better understanding of the relationship between sensory and emotional responses.

Köster [11] differentiates explicit and implicit emotion measurements. Explicit emotion measurements are an indirect method for the determination of emotions. In other words people have to express their feelings and document it—for example, with the help of a questionnaire. The implicit methods measure direct physiological or psychophysiological responses ([11]). Examples include the measurement of heart rate, skin conductance, face reading, eye tracking and behavioural tests [10,11,38].

However, some of these methods still have limitations: Leitch et al. [41] and Walsh et al. [42] showed that a better differentiation was reached by applying a hedonic test based on liking and an emotion term questionnaire, compared to facial expression. Eye tracking measures the reaction to the appearance of a product [11,43,44], rather than the emotional response.

The current study focused on explicit measurements; however, the combination with direct measurements is an interesting element for future study. Meiselman [10] states that the objective of the study should determine the choice of method.

## Supporting Information

S1 Dataset data_MDMQ_all products. (XLSX)

S2 Dataset data_EFT_:all products. (XLSX)

S3 Dataset data_hedonic sensory test_all products. (XLSX)

S1 File statistical analysis_MDMQ milk. (PDF)

S2 File statistical analysis_MDMQ water. (PDF)

S3 File statistical analysis_MDMQ bread. (PDF)

S4 File statistical analysis_MDMQ sugar. (PDF)

S5 File statistical analysis_EFT water. (PDF)

S6 File statistical analysis_EFT milk. (PDF)

S7 File statistical analysis_EFT bread. (PDF)

S8 File statistical analysis_EFT sugar. (PDF)

S9 File statistical analysis_hedonic sensory test water. (PDF)

S10 File statistical analysis_hedonic sensory test milk. (PDF)

S11 File statistical analysis_hedonic sensory test bread. (PDF)

S12 File statistical analysis_hedonic sensory test sugar. (PDF)
