# Supplementary material for: Development and Application of a Test for Food-Induced Emotions
Source: PLoS One. 2016 Nov 18;11(11):e0165991. doi: 10.1371/journal.pone.0165991 (PMC5115674; doi:10.1371/journal.pone.0165991)
Supplement: S1 File — (PDF) [file pone.0165991.s004.pdf]

```
GLM Item1.Söbbeke.11.12.12 Item1.Söbbeke.23.01.13 Item1.Landliebe.11.12.12 Item1.Landliebe.2
  /WSFACTOR=Produkttyp 2 Polynomial Messzeitpunkt 2 Polynomial
  /METHOD=SSTYPE(3)
  /EMMEANS=TABLES(Produkttyp)
  /PRINT=DESCRIPTIVE ETASQ
  /CRITERIA=ALPHA(.05)
  /WSDESIGN=Produkttyp Messzeitpunkt Produkttyp*Messzeitpunkt.
```

General Linear Model

Notes

Output Created		07-NOV-2013 12:03:15
Comments		
Input	Data	C:\Documents and Settings\Dennis Boywitt\My Documents\My Dropbox\Freiberufliche Tätigkeit\Forschungsring\Daten\MDBF_Gruppe3_Items.sav
	Active Dataset	DataSet3
	Filter	<none>
	Weight	<none>
	Split File	<none>
	N of Rows in Working Data File	70
Missing Value Handling	Definition of Missing	User-defined missing values are treated as missing.
	Cases Used	Statistics are based on all cases with valid data for all variables in the model.
Syntax		GLM Item1.Söbbeke.11.12.12 Item1.Söbbeke.23.01.13 Item1.Landliebe.11.12.12 Item1.Landliebe.23.01.13 /WSFACTOR=Produkttyp 2 Polynomial Messzeitpunkt 2 Polynomial /METHOD=SSTYPE(3) /EMMEANS=TABLES(Produkttyp) /PRINT=DESCRIPTIVE ETASQ /CRITERIA=ALPHA(.05) /WSDESIGN=Produkttyp Messzeitpunkt Produkttyp*Messzeitpunkt.

Notes

Resources	Processor Time	00:00:00,03
	Elapsed Time	00:00:00,03

```
[DataSet3] C:\Documents and Settings\Dennis Boywitt\My Documents\My Dropbo
x\Freiberufliche Tätigkeit\Forschungsring\Daten\MDBF_Gruppe3_Items.sav
```

Within-Subjects Factors

Measure: MEASURE_1

Produkttyp	Messzeitpunkt	Dependent Variable
1	1	Item1. Söbbeke. 11.12.12
	2	Item1. Söbbeke. 23.01.13
2	1	Item1. Landliebe. 11.12.12
	2	Item1. Landliebe. 23.01.13

Descriptive Statistics

	Mean	Std. Deviation	N
Item1.Söbbeke.11.12.12	2,70	,356	64
Item1.Söbbeke.23.01.13	2,55	,712	64
Item1.Landliebe.11.12.12	2,66	,303	64
Item1.Landliebe.23.01.13	2,28	1,026	64

Multivariate Tests[a]

Effect		Value	F	Hypothesis df	Error df
Produkttyp	Pillai's Trace	,077	5,271[b]	1,000	63,000
	Wilks' Lambda	,923	5,271[b]	1,000	63,000
	Hotelling's Trace	,084	5,271[b]	1,000	63,000
	Roy's Largest Root	,084	5,271[b]	1,000	63,000
Messzeitpunkt	Pillai's Trace	,107	7,542[b]	1,000	63,000
	Wilks' Lambda	,893	7,542[b]	1,000	63,000
	Hotelling's Trace	,120	7,542[b]	1,000	63,000
	Roy's Largest Root	,120	7,542[b]	1,000	63,000
Produkttyp * Messzeitpunkt	Pillai's Trace	,038	2,504[b]	1,000	63,000
	Wilks' Lambda	,962	2,504[b]	1,000	63,000
	Hotelling's Trace	,040	2,504[b]	1,000	63,000
	Roy's Largest Root	,040	2,504[b]	1,000	63,000

Multivariate Tests[a]

Effect		Sig.	Partial Eta Squared
Produkttyp	Pillai's Trace	,025	,077
	Wilks' Lambda	,025	,077
	Hotelling's Trace	,025	,077
	Roy's Largest Root	,025	,077
Messzeitpunkt	Pillai's Trace	,008	,107
	Wilks' Lambda	,008	,107
	Hotelling's Trace	,008	,107
	Roy's Largest Root	,008	,107
Produkttyp * Messzeitpunkt	Pillai's Trace	,119	,038
	Wilks' Lambda	,119	,038
	Hotelling's Trace	,119	,038
	Roy's Largest Root	,119	,038

a. Design: Intercept
Within Subjects Design: Produkttyp + Messzeitpunkt + Produkttyp * Messzeitpunkt

b. Exact statistic

Mauchly's Test of Sphericity[a]

Measure: MEASURE_1

Within Subjects Effect	Mauchly's W	Approx. Chi-Square	df	Sig.	Epsilon[b]
					Greenhouse-Geisser
Produkttyp	1,000	,000	0	.	1,000
Messzeitpunkt	1,000	,000	0	.	1,000
Produkttyp * Messzeitpunkt	1,000	,000	0	.	1,000

Mauchly's Test of Sphericity[a]

Measure: MEASURE_1

Within Subjects Effect	Epsilon[b]	
	Huynh-Feldt	Lower-bound
Produkttyp	1,000	1,000
Messzeitpunkt	1,000	1,000
Produkttyp * Messzeitpunkt	1,000	1,000

Tests the null hypothesis that the error covariance matrix of the orthonormalized transformed dependent variables is proportional to an identity matrix.

a. Design: Intercept
Within Subjects Design: Produkttyp + Messzeitpunkt + Produkttyp * Messzeitpunkt

b. May be used to adjust the degrees of freedom for the averaged tests of significance. Corrected tests are displayed in the Tests of Within-Subjects Effects table.

Tests of Within-Subjects Effects

Measure: MEASURE_1

Source		Type III Sum of Squares	df	Mean Square
Produkttyp	Sphericity Assumed	1,524	1	1,524
	Greenhouse-Geisser	1,524	1,000	1,524
	Huynh-Feldt	1,524	1,000	1,524
	Lower-bound	1,524	1,000	1,524
Error(Produkttyp)	Sphericity Assumed	18,211	63	,289
	Greenhouse-Geisser	18,211	63,000	,289
	Huynh-Feldt	18,211	63,000	,289
	Lower-bound	18,211	63,000	,289
Messzeitpunkt	Sphericity Assumed	4,582	1	4,582
	Greenhouse-Geisser	4,582	1,000	4,582
	Huynh-Feldt	4,582	1,000	4,582
	Lower-bound	4,582	1,000	4,582
Error(Messzeitpunkt)	Sphericity Assumed	38,277	63	,608
	Greenhouse-Geisser	38,277	63,000	,608
	Huynh-Feldt	38,277	63,000	,608
	Lower-bound	38,277	63,000	,608
Produkttyp * Messzeitpunkt	Sphericity Assumed	,850	1	,850
	Greenhouse-Geisser	,850	1,000	,850
	Huynh-Feldt	,850	1,000	,850
	Lower-bound	,850	1,000	,850
Error (Produkttyp*Messzeitpunkt)	Sphericity Assumed	21,385	63	,339
	Greenhouse-Geisser	21,385	63,000	,339
	Huynh-Feldt	21,385	63,000	,339
	Lower-bound	21,385	63,000	,339

Tests of Within-Subjects Effects

Measure: MEASURE_1

Source		F	Sig.	Partial Eta Squared
Produkttyp	Sphericity Assumed	5,271	,025	,077
	Greenhouse-Geisser	5,271	,025	,077
	Huynh-Feldt	5,271	,025	,077
	Lower-bound	5,271	,025	,077
Error(Produkttyp)	Sphericity Assumed			
	Greenhouse-Geisser			
	Huynh-Feldt			
	Lower-bound			
Messzeitpunkt	Sphericity Assumed	7,542	,008	,107
	Greenhouse-Geisser	7,542	,008	,107
	Huynh-Feldt	7,542	,008	,107
	Lower-bound	7,542	,008	,107
Error(Messzeitpunkt)	Sphericity Assumed			
	Greenhouse-Geisser			
	Huynh-Feldt			
	Lower-bound			
Produkttyp * Messzeitpunkt	Sphericity Assumed	2,504	,119	,038
	Greenhouse-Geisser	2,504	,119	,038
	Huynh-Feldt	2,504	,119	,038
	Lower-bound	2,504	,119	,038
Error (Produkttyp*Messzeitpunkt)	Sphericity Assumed			
	Greenhouse-Geisser			
	Huynh-Feldt			
	Lower-bound			

Tests of Within-Subjects Contrasts

Measure: MEASURE_1

Source	Produkttyp	Messzeitpunkt	Type III Sum of Squares	df	Mean Square
Produkttyp	Linear		1,524	1	1,524
Error(Produkttyp)	Linear		18,211	63	,289
Messzeitpunkt		Linear	4,582	1	4,582
Error(Messzeitpunkt)		Linear	38,277	63	,608
Produkttyp * Messzeitpunkt	Linear	Linear	,850	1	,850
Error (Produkttyp*Messzeitpunkt)	Linear	Linear	21,385	63	,339

Tests of Within-Subjects Contrasts

Measure: MEASURE_1

Source	Produkttyp	Messzeitpunkt	F	Sig.	Partial Eta Squared
Produkttyp	Linear		5,271	,025	,077
Error(Produkttyp)	Linear				
Messzeitpunkt		Linear	7,542	,008	,107
Error(Messzeitpunkt)		Linear			
Produkttyp * Messzeitpunkt	Linear	Linear	2,504	,119	,038
Error (Produkttyp*Messzeitpunkt)	Linear	Linear			

Tests of Between-Subjects Effects

Measure: MEASURE_1

Transformed Variable: Average

Source	Type III Sum of Squares	df	Mean Square	F	Sig.	Partial Eta Squared
Intercept	1664,385	1	1664,385	3075,021	,000	,980
Error	34,099	63	,541			

Estimated Marginal Means

Produkttyp

Measure: MEASURE_1

Produkttyp	Mean	Std. Error	95% Confidence Interval	
			Lower Bound	Upper Bound
1	2,627	,049	2,530	2,724
2	2,473	,064	2,345	2,601

```
GLM Item2.Söbbeke.11.12.12 Item2.Söbbeke.23.01.13 Item2.Landliebe.11.12.12 Item2.Landliebe.2
  /WSFACTOR=Produkttyp 2 Polynomial Messzeitpunkt 2 Polynomial
  /METHOD=SSTYPE(3)
  /EMMEANS=TABLES(Produkttyp)
  /PRINT=DESCRIPTIVE ETASQ
  /CRITERIA=ALPHA(.05)
  /WSDESIGN=Produkttyp Messzeitpunkt Produkttyp*Messzeitpunkt.
```

General Linear Model

Notes

Output Created		07-NOV-2013 12:10:17
Comments		
Input	Data	C:\Documents and Settings\Dennis Boywitt\My Documents\My Dropbox\Freiberufliche Tätigkeit\Forschungsring\Daten\MDBF_Gruppe3_Items.sav
	Active Dataset	DataSet3
	Filter	<none>
	Weight	<none>
	Split File	<none>
	N of Rows in Working Data File	70
Missing Value Handling	Definition of Missing	User-defined missing values are treated as missing.
	Cases Used	Statistics are based on all cases with valid data for all variables in the model.
Syntax		GLM Item2.Söbbeke.11.12.12 Item2.Söbbeke.23.01.13 Item2.Landliebe.11.12.12 Item2.Landliebe.23.01.13<br>/WSFACTOR=Produkttyp 2 Polynomial Messzeitpunkt 2 Polynomial<br>/METHOD=SSTYPE(3)<br>/EMMEANS=TABLES(Produkttyp)<br>/PRINT=DESCRIPTIVE ETASQ<br>/CRITERIA=ALPHA(.05)<br>/WSDESIGN=Produkttyp Messzeitpunkt Produkttyp*Messzeitpunkt.
Resources	Processor Time	00:00:00,02
	Elapsed Time	00:00:00,02

```
[DataSet3] C:\Documents and Settings\Dennis Boywitt\My Documents\My Dropbox\Freiberufliche Tätigkeit\Forschungsring\Daten\MDBF_Gruppe3_Items.sav
```

Within-Subjects Factors

Measure: MEASURE_1

Produkttyp	Messzeitpunkt	Dependent Variable
1	1	Item2.Söbbeke.11.12.12
	2	Item2.Söbbeke.23.01.13
2	1	Item2.Landliebe.11.12.12
	2	Item2.Landliebe.23.01.13

Descriptive Statistics

	Mean	Std. Deviation	N
Item2.Söbbeke.11.12.12	2,77	,397	64
Item2.Söbbeke.23.01.13	2,50	,729	64
Item2.Landliebe.11.12.12	2,76	,375	64
Item2.Landliebe.23.01.13	2,32	1,051	64

Multivariate Tests[a]

Effect		Value	F	Hypothesis df	Error df
Produkttyp	Pillai's Trace	,026	1,656[b]	1,000	63,000
	Wilks' Lambda	,974	1,656[b]	1,000	63,000
	Hotelling's Trace	,026	1,656[b]	1,000	63,000
	Roy's Largest Root	,026	1,656[b]	1,000	63,000
Messzeitpunkt	Pillai's Trace	,165	12,435[b]	1,000	63,000
	Wilks' Lambda	,835	12,435[b]	1,000	63,000
	Hotelling's Trace	,197	12,435[b]	1,000	63,000
	Roy's Largest Root	,197	12,435[b]	1,000	63,000
Produkttyp * Messzeitpunkt	Pillai's Trace	,017	1,121[b]	1,000	63,000
	Wilks' Lambda	,983	1,121[b]	1,000	63,000
	Hotelling's Trace	,018	1,121[b]	1,000	63,000
	Roy's Largest Root	,018	1,121[b]	1,000	63,000

Multivariate Tests[a]

Effect		Sig.	Partial Eta Squared
Produkttyp	Pillai's Trace	,203	,026
	Wilks' Lambda	,203	,026
	Hotelling's Trace	,203	,026
	Roy's Largest Root	,203	,026
Messzeitpunkt	Pillai's Trace	,001	,165
	Wilks' Lambda	,001	,165
	Hotelling's Trace	,001	,165
	Roy's Largest Root	,001	,165
Produkttyp * Messzeitpunkt	Pillai's Trace	,294	,017
	Wilks' Lambda	,294	,017
	Hotelling's Trace	,294	,017
	Roy's Largest Root	,294	,017

a. Design: Intercept
Within Subjects Design: Produkttyp + Messzeitpunkt + Produkttyp * Messzeitpunkt

b. Exact statistic

Mauchly's Test of Sphericity[a]

Measure: MEASURE_1

Within Subjects Effect	Mauchly's W	Approx. Chi-Square	df	Sig.	Epsilon[b]
					Greenhouse-Geisser
Produkttyp	1,000	,000	0	.	1,000
Messzeitpunkt	1,000	,000	0	.	1,000
Produkttyp * Messzeitpunkt	1,000	,000	0	.	1,000

Mauchly's Test of Sphericity[a]

Measure: MEASURE_1

Within Subjects Effect	Epsilon[b]	
	Huynh-Feldt	Lower-bound
Produkttyp	1,000	1,000
Messzeitpunkt	1,000	1,000
Produkttyp * Messzeitpunkt	1,000	1,000

Tests the null hypothesis that the error covariance matrix of the orthonormalized transformed dependent variables is proportional to an identity matrix.

a. Design: Intercept
Within Subjects Design: Produkttyp + Messzeitpunkt + Produkttyp * Messzeitpunkt

b. May be used to adjust the degrees of freedom for the averaged tests of significance. Corrected tests are displayed in the Tests of Within-Subjects Effects table.

Tests of Within-Subjects Effects

Measure: MEASURE_1

Source		Type III Sum of Squares	df	Mean Square
Produkttyp	Sphericity Assumed	,586	1	,586
	Greenhouse-Geisser	,586	1,000	,586
	Huynh-Feldt	,586	1,000	,586
	Lower-bound	,586	1,000	,586
Error(Produkttyp)	Sphericity Assumed	22,304	63	,354
	Greenhouse-Geisser	22,304	63,000	,354
	Huynh-Feldt	22,304	63,000	,354
	Lower-bound	22,304	63,000	,354
Messzeitpunkt	Sphericity Assumed	7,823	1	7,823
	Greenhouse-Geisser	7,823	1,000	7,823
	Huynh-Feldt	7,823	1,000	7,823
	Lower-bound	7,823	1,000	7,823
Error(Messzeitpunkt)	Sphericity Assumed	39,631	63	,629
	Greenhouse-Geisser	39,631	63,000	,629
	Huynh-Feldt	39,631	63,000	,629
	Lower-bound	39,631	63,000	,629
Produkttyp * Messzeitpunkt	Sphericity Assumed	,451	1	,451
	Greenhouse-Geisser	,451	1,000	,451
	Huynh-Feldt	,451	1,000	,451
	Lower-bound	,451	1,000	,451
Error (Produkttyp*Messzeitpunkt)	Sphericity Assumed	25,377	63	,403
	Greenhouse-Geisser	25,377	63,000	,403
	Huynh-Feldt	25,377	63,000	,403
	Lower-bound	25,377	63,000	,403

Tests of Within-Subjects Effects

Measure: MEASURE_1

Source		F	Sig.	Partial Eta Squared
Produkttyp	Sphericity Assumed	1,656	,203	,026
	Greenhouse-Geisser	1,656	,203	,026
	Huynh-Feldt	1,656	,203	,026
	Lower-bound	1,656	,203	,026
Error(Produkttyp)	Sphericity Assumed			
	Greenhouse-Geisser			
	Huynh-Feldt			
	Lower-bound			
Messzeitpunkt	Sphericity Assumed	12,435	,001	,165
	Greenhouse-Geisser	12,435	,001	,165
	Huynh-Feldt	12,435	,001	,165
	Lower-bound	12,435	,001	,165
Error(Messzeitpunkt)	Sphericity Assumed			
	Greenhouse-Geisser			
	Huynh-Feldt			
	Lower-bound			
Produkttyp * Messzeitpunkt	Sphericity Assumed	1,121	,294	,017
	Greenhouse-Geisser	1,121	,294	,017
	Huynh-Feldt	1,121	,294	,017
	Lower-bound	1,121	,294	,017
Error (Produkttyp*Messzeitpunkt)	Sphericity Assumed			
	Greenhouse-Geisser			
	Huynh-Feldt			
	Lower-bound			

Tests of Within-Subjects Contrasts

Measure: MEASURE_1

Source	Produkttyp	Messzeitpunkt	Type III Sum of Squares	df	Mean Square
Produkttyp	Linear		,586	1	,586
Error(Produkttyp)	Linear		22,304	63	,354
Messzeitpunkt		Linear	7,823	1	7,823
Error(Messzeitpunkt)		Linear	39,631	63	,629
Produkttyp * Messzeitpunkt	Linear	Linear	,451	1	,451
Error (Produkttyp*Messzeitpunkt)	Linear	Linear	25,377	63	,403

Tests of Within-Subjects Contrasts

Measure: MEASURE_1

Source	Produkttyp	Messzeitpunkt	F	Sig.	Partial Eta Squared
Produkttyp	Linear		1,656	,203	,026
Error(Produkttyp)	Linear				
Messzeitpunkt		Linear	12,435	,001	,165
Error(Messzeitpunkt)		Linear			
Produkttyp * Messzeitpunkt	Linear	Linear	1,121	,294	,017
Error (Produkttyp*Messzeitpunkt)	Linear	Linear			

Tests of Between-Subjects Effects

Measure: MEASURE_1

Transformed Variable: Average

Source	Type III Sum of Squares	df	Mean Square	F	Sig.	Partial Eta Squared
Intercept	1715,772	1	1715,772	3133,703	,000	,980
Error	34,494	63	,548			

Estimated Marginal Means

Produkttyp

Measure: MEASURE_1

Produkttyp	Mean	Std. Error	95% Confidence Interval	
			Lower Bound	Upper Bound
1	2,637	,049	2,538	2,735
2	2,541	,068	2,405	2,677

```
GLM Item3.Söbbeke.11.12.12 Item3.Söbbeke.23.01.13 Item3.Landliebe.11.12.12 Item3.Landliebe.2
  /WSFACTOR=Produkttyp 2 Polynomial Messzeitpunkt 2 Polynomial
  /METHOD=SSTYPE(3)
  /EMMEANS=TABLES(Produkttyp)
  /PRINT=DESCRIPTIVE ETASQ
  /CRITERIA=ALPHA(.05)
  /WSDESIGN=Produkttyp Messzeitpunkt Produkttyp*Messzeitpunkt.
```

General Linear Model

Notes

Output Created		07-NOV-2013 12:12:01
Comments		
Input	Data	C:\Documents and Settings\Dennis Boywitt\My Documents\My Dropbox\Freiberufliche Tätigkeit\Forschungsring\Daten\MDBF_Gruppe3_Items.sav
	Active Dataset	DataSet3
	Filter	<none>
	Weight	<none>
	Split File	<none>
	N of Rows in Working Data File	70
Missing Value Handling	Definition of Missing	User-defined missing values are treated as missing.
	Cases Used	Statistics are based on all cases with valid data for all variables in the model.
Syntax		GLM Item3.Söbbeke.11.12.12 Item3.Söbbeke.23.01.13 Item3.Landliebe.11.12.12 Item3.Landliebe.23.01.13 /WSFACTOR=Produkttyp 2 Polynomial Messzeitpunkt 2 Polynomial /METHOD=SSTYPE(3) /EMMEANS=TABLES(Produkttyp) /PRINT=DESCRIPTIVE ETASQ /CRITERIA=ALPHA(.05) /WSDESIGN=Produkttyp Messzeitpunkt Produkttyp*Messzeitpunkt.
Resources	Processor Time	00:00:00,03
	Elapsed Time	00:00:00,03

```
[DataSet3] C:\Documents and Settings\Dennis Boywitt\My Documents\My Dropbox\Freiberufliche Tätigkeit\Forschungsring\Daten\MDBF_Gruppe3_Items.sav
```

Within-Subjects Factors

Measure: MEASURE_1

Produkttyp	Messzeitpunkt	Dependent Variable
1	1	Item3.Söbbeke.11.12.12
	2	Item3.Söbbeke.23.01.13
2	1	Item3.Landliebe.11.12.12
	2	Item3.Landliebe.23.01.13

Descriptive Statistics

	Mean	Std. Deviation	N
Item3.Söbbeke.11.12.12	2,78	,357	52
Item3.Söbbeke.23.01.13	2,75	,419	52
Item3.Landliebe.11.12.12	2,75	,405	52
Item3.Landliebe.23.01.13	2,86	,412	52

Multivariate Tests[a]

Effect		Value	F	Hypothesis df	Error df
Produkttyp	Pillai's Trace	,009	,455[b]	1,000	51,000
	Wilks' Lambda	,991	,455[b]	1,000	51,000
	Hotelling's Trace	,009	,455[b]	1,000	51,000
	Roy's Largest Root	,009	,455[b]	1,000	51,000
Messzeitpunkt	Pillai's Trace	,013	,695[b]	1,000	51,000
	Wilks' Lambda	,987	,695[b]	1,000	51,000
	Hotelling's Trace	,014	,695[b]	1,000	51,000
	Roy's Largest Root	,014	,695[b]	1,000	51,000
Produkttyp * Messzeitpunkt	Pillai's Trace	,030	1,566[b]	1,000	51,000
	Wilks' Lambda	,970	1,566[b]	1,000	51,000
	Hotelling's Trace	,031	1,566[b]	1,000	51,000
	Roy's Largest Root	,031	1,566[b]	1,000	51,000

Multivariate Tests[a]

Effect		Sig.	Partial Eta Squared
Produkttyp	Pillai's Trace	,503	,009
	Wilks' Lambda	,503	,009
	Hotelling's Trace	,503	,009
	Roy's Largest Root	,503	,009
Messzeitpunkt	Pillai's Trace	,408	,013
	Wilks' Lambda	,408	,013
	Hotelling's Trace	,408	,013
	Roy's Largest Root	,408	,013
Produkttyp * Messzeitpunkt	Pillai's Trace	,216	,030
	Wilks' Lambda	,216	,030
	Hotelling's Trace	,216	,030
	Roy's Largest Root	,216	,030

a. Design: Intercept
Within Subjects Design: Produkttyp + Messzeitpunkt + Produkttyp * Messzeitpunkt

b. Exact statistic

Mauchly's Test of Sphericity[a]

Measure: MEASURE_1

Within Subjects Effect	Mauchly's W	Approx. Chi-Square	df	Sig.	Epsilon[b]
					Greenhouse-Geisser
Produkttyp	1,000	,000	0	.	1,000
Messzeitpunkt	1,000	,000	0	.	1,000
Produkttyp * Messzeitpunkt	1,000	,000	0	.	1,000

Mauchly's Test of Sphericity[a]

Measure: MEASURE_1

Within Subjects Effect	Epsilon[b]	
	Huynh-Feldt	Lower-bound
Produkttyp	1,000	1,000
Messzeitpunkt	1,000	1,000
Produkttyp * Messzeitpunkt	1,000	1,000

Tests the null hypothesis that the error covariance matrix of the orthonormalized transformed dependent variables is proportional to an identity matrix.

a. Design: Intercept
Within Subjects Design: Produkttyp + Messzeitpunkt + Produkttyp * Messzeitpunkt

b. May be used to adjust the degrees of freedom for the averaged tests of significance. Corrected tests are displayed in the Tests of Within-Subjects Effects table.

Tests of Within-Subjects Effects

Measure: MEASURE_1

Source		Type III Sum of Squares	df	Mean Square
Produkttyp	Sphericity Assumed	,059	1	,059
	Greenhouse-Geisser	,059	1,000	,059
	Huynh-Feldt	,059	1,000	,059
	Lower-bound	,059	1,000	,059
Error(Produkttyp)	Sphericity Assumed	6,597	51	,129
	Greenhouse-Geisser	6,597	51,000	,129
	Huynh-Feldt	6,597	51,000	,129
	Lower-bound	6,597	51,000	,129
Messzeitpunkt	Sphericity Assumed	,077	1	,077
	Greenhouse-Geisser	,077	1,000	,077
	Huynh-Feldt	,077	1,000	,077
	Lower-bound	,077	1,000	,077
Error(Messzeitpunkt)	Sphericity Assumed	5,642	51	,111
	Greenhouse-Geisser	5,642	51,000	,111
	Huynh-Feldt	5,642	51,000	,111
	Lower-bound	5,642	51,000	,111
Produkttyp * Messzeitpunkt	Sphericity Assumed	,236	1	,236
	Greenhouse-Geisser	,236	1,000	,236
	Huynh-Feldt	,236	1,000	,236
	Lower-bound	,236	1,000	,236
Error (Produkttyp*Messzeitpunkt)	Sphericity Assumed	7,671	51	,150
	Greenhouse-Geisser	7,671	51,000	,150
	Huynh-Feldt	7,671	51,000	,150
	Lower-bound	7,671	51,000	,150

Tests of Within-Subjects Effects

Measure: MEASURE_1

Source		F	Sig.	Partial Eta Squared
Produkttyp	Sphericity Assumed	,455	,503	,009
	Greenhouse-Geisser	,455	,503	,009
	Huynh-Feldt	,455	,503	,009
	Lower-bound	,455	,503	,009
Error(Produkttyp)	Sphericity Assumed			
	Greenhouse-Geisser			
	Huynh-Feldt			
	Lower-bound			
Messzeitpunkt	Sphericity Assumed	,695	,408	,013
	Greenhouse-Geisser	,695	,408	,013
	Huynh-Feldt	,695	,408	,013
	Lower-bound	,695	,408	,013
Error(Messzeitpunkt)	Sphericity Assumed			
	Greenhouse-Geisser			
	Huynh-Feldt			
	Lower-bound			
Produkttyp * Messzeitpunkt	Sphericity Assumed	1,566	,216	,030
	Greenhouse-Geisser	1,566	,216	,030
	Huynh-Feldt	1,566	,216	,030
	Lower-bound	1,566	,216	,030
Error (Produkttyp*Messzeitpunkt)	Sphericity Assumed			
	Greenhouse-Geisser			
	Huynh-Feldt			
	Lower-bound			

Tests of Within-Subjects Contrasts

Measure: MEASURE_1

Source	Produkttyp	Messzeitpunkt	Type III Sum of Squares	df	Mean Square
Produkttyp	Linear		,059	1	,059
Error(Produkttyp)	Linear		6,597	51	,129
Messzeitpunkt		Linear	,077	1	,077
Error(Messzeitpunkt)		Linear	5,642	51	,111
Produkttyp * Messzeitpunkt	Linear	Linear	,236	1	,236
Error (Produkttyp*Messzeitpunkt)	Linear	Linear	7,671	51	,150

Tests of Within-Subjects Contrasts

Measure: MEASURE_1

Source	Produkttyp	Messzeitpunkt	F	Sig.	Partial Eta Squared
Produkttyp	Linear		,455	,503	,009
Error(Produkttyp)	Linear				
Messzeitpunkt		Linear	,695	,408	,013
Error(Messzeitpunkt)		Linear			
Produkttyp * Messzeitpunkt	Linear	Linear	1,566	,216	,030
Error (Produkttyp*Messzeitpunkt)	Linear	Linear			

Tests of Between-Subjects Effects

Measure: MEASURE_1

Transformed Variable: Average

Source	Type III Sum of Squares	df	Mean Square	F	Sig.	Partial Eta Squared
Intercept	1614,520	1	1614,520	6548,832	,000	,992
Error	12,573	51	,247			

Estimated Marginal Means

Produkttyp

Measure: MEASURE_1

Produkttyp	Mean	Std. Error	95% Confidence Interval	
			Lower Bound	Upper Bound
1	2,769	,046	2,678	2,861
2	2,803	,039	2,724	2,882
