# Supplementary material for: Development and Application of a Test for Food-Induced Emotions
Source: PLoS One. 2016 Nov 18;11(11):e0165991. doi: 10.1371/journal.pone.0165991 (PMC5115674; doi:10.1371/journal.pone.0165991)
Supplement: S2 File — (PDF) [file pone.0165991.s005.pdf]

```
GLM Item1.AGlas.11.12.12 Item1.AGlas.23.01.13 Item1.APlastik.11.12.12 Item1.APlastik.23.01.1
   /WSFACTOR=Produkttyp 2 Polynomial Messzeitpunkt 2 Polynomial
   /METHOD=SSTYPE(3)
   /EMMEANS=TABLES(Produkttyp)
   /PRINT=DESCRIPTIVE ETASQ
   /CRITERIA=ALPHA(.05)
   /WSDESIGN=Produkttyp Messzeitpunkt Produkttyp*Messzeitpunkt.
```

General Linear Model

Notes

Output Created		07-NOV-2013 12:00:31
Comments		
Input	Data	C:\Documents and Settings\Dennis Boywitt\My Documents\My Dropbox\Freiberufliche Tätigkeit\Forschungsring\Daten\MDBF_Gruppe3_Items.sav
	Active Dataset	DataSet3
	Filter	<none>
	Weight	<none>
	Split File	<none>
	N of Rows in Working Data File	70
Missing Value Handling	Definition of Missing	User-defined missing values are treated as missing.
	Cases Used	Statistics are based on all cases with valid data for all variables in the model.
Syntax		GLM Item1.AGlas.11.12.12 Item1.AGlas.23.01.13 Item1.APlastik.11.12.12 Item1.APlastik.23.01.13 /WSFACTOR=Produkttyp 2 Polynomial Messzeitpunkt 2 Polynomial /METHOD=SSTYPE(3) /EMMEANS=TABLES(Produkttyp) /PRINT=DESCRIPTIVE ETASQ /CRITERIA=ALPHA(.05) /WSDESIGN=Produkttyp Messzeitpunkt ...

Notes

Resources	Processor Time	00:00:00,02
	Elapsed Time	00:00:00,03

```
[DataSet3] C:\Documents and Settings\Dennis Boywitt\My Documents\My Dropbo
x\Freiberufliche Tätigkeit\Forschungsring\Daten\MDBF_Gruppe3_Items.sav
```

Within-Subjects Factors

Measure: MEASURE_1

Produkttyp	Messzeitpunkt	Dependent Variable
1	1	Item1.AGlas.11.12.12
	2	Item1.AGlas.23.01.13
2	1	Item1.APlastik.11.12.12
	2	Item1.APlastik.23.01.13

Descriptive Statistics

	Mean	Std. Deviation	N
Item1.AGlas.11.12.12	2,57	,360	64
Item1.AGlas.23.01.13	2,53	,739	64
Item1.APlastik.11.12.12	2,52	,490	64
Item1.APlastik.23.01.13	2,51	,720	64

Multivariate Tests[a]

Effect		Value	F	Hypothesis df	Error df
Produkttyp	Pillai's Trace	,024	1,536[b]	1,000	63,000
	Wilks' Lambda	,976	1,536[b]	1,000	63,000
	Hotelling's Trace	,024	1,536[b]	1,000	63,000
	Roy's Largest Root	,024	1,536[b]	1,000	63,000
Messzeitpunkt	Pillai's Trace	,001	,046[b]	1,000	63,000
	Wilks' Lambda	,999	,046[b]	1,000	63,000
	Hotelling's Trace	,001	,046[b]	1,000	63,000
	Roy's Largest Root	,001	,046[b]	1,000	63,000
Produkttyp * Messzeitpunkt	Pillai's Trace	,005	,333[b]	1,000	63,000
	Wilks' Lambda	,995	,333[b]	1,000	63,000
	Hotelling's Trace	,005	,333[b]	1,000	63,000
	Roy's Largest Root	,005	,333[b]	1,000	63,000

Multivariate Tests[a]

Effect		Sig.	Partial Eta Squared
Produkttyp	Pillai's Trace	,220	,024
	Wilks' Lambda	,220	,024
	Hotelling's Trace	,220	,024
	Roy's Largest Root	,220	,024
Messzeitpunkt	Pillai's Trace	,830	,001
	Wilks' Lambda	,830	,001
	Hotelling's Trace	,830	,001
	Roy's Largest Root	,830	,001
Produkttyp * Messzeitpunkt	Pillai's Trace	,566	,005
	Wilks' Lambda	,566	,005
	Hotelling's Trace	,566	,005
	Roy's Largest Root	,566	,005

a. Design: Intercept
Within Subjects Design: Produkttyp + Messzeitpunkt + Produkttyp * Messzeitpunkt

b. Exact statistic

Mauchly's Test of Sphericity[a]

Measure: MEASURE_1

Within Subjects Effect	Mauchly's W	Approx. Chi-Square	df	Sig.	Epsilon[b]
					Greenhouse-Geisser
Produkttyp	1,000	,000	0	.	1,000
Messzeitpunkt	1,000	,000	0	.	1,000
Produkttyp * Messzeitpunkt	1,000	,000	0	.	1,000

Mauchly's Test of Sphericity[a]

Measure: MEASURE_1

Within Subjects Effect	Epsilon[b]	
	Huynh-Feldt	Lower-bound
Produkttyp	1,000	1,000
Messzeitpunkt	1,000	1,000
Produkttyp * Messzeitpunkt	1,000	1,000

Tests the null hypothesis that the error covariance matrix of the orthonormalized transformed dependent variables is proportional to an identity matrix.

a. Design: Intercept
Within Subjects Design: Produkttyp + Messzeitpunkt + Produkttyp * Messzeitpunkt

b. May be used to adjust the degrees of freedom for the averaged tests of significance. Corrected tests are displayed in the Tests of Within-Subjects Effects table.

Tests of Within-Subjects Effects

Measure: MEASURE_1

Source		Type III Sum of Squares	df	Mean Square
Produkttyp	Sphericity Assumed	,088	1	,088
	Greenhouse-Geisser	,088	1,000	,088
	Huynh-Feldt	,088	1,000	,088
	Lower-bound	,088	1,000	,088
Error(Produkttyp)	Sphericity Assumed	3,615	63	,057
	Greenhouse-Geisser	3,615	63,000	,057
	Huynh-Feldt	3,615	63,000	,057
	Lower-bound	3,615	63,000	,057
Messzeitpunkt	Sphericity Assumed	,030	1	,030
	Greenhouse-Geisser	,030	1,000	,030
	Huynh-Feldt	,030	1,000	,030
	Lower-bound	,030	1,000	,030
Error(Messzeitpunkt)	Sphericity Assumed	40,174	63	,638
	Greenhouse-Geisser	40,174	63,000	,638
	Huynh-Feldt	40,174	63,000	,638
	Lower-bound	40,174	63,000	,638
Produkttyp * Messzeitpunkt	Sphericity Assumed	,020	1	,020
	Greenhouse-Geisser	,020	1,000	,020
	Huynh-Feldt	,020	1,000	,020
	Lower-bound	,020	1,000	,020
Error (Produkttyp*Messzeitpunkt)	Sphericity Assumed	3,746	63	,059
	Greenhouse-Geisser	3,746	63,000	,059
	Huynh-Feldt	3,746	63,000	,059
	Lower-bound	3,746	63,000	,059

Tests of Within-Subjects Effects

Measure: MEASURE_1

Source		F	Sig.	Partial Eta Squared
Produkttyp	Sphericity Assumed	1,536	,220	,024
	Greenhouse-Geisser	1,536	,220	,024
	Huynh-Feldt	1,536	,220	,024
	Lower-bound	1,536	,220	,024
Error(Produkttyp)	Sphericity Assumed			
	Greenhouse-Geisser			
	Huynh-Feldt			
	Lower-bound			
Messzeitpunkt	Sphericity Assumed	,046	,830	,001
	Greenhouse-Geisser	,046	,830	,001
	Huynh-Feldt	,046	,830	,001
	Lower-bound	,046	,830	,001
Error(Messzeitpunkt)	Sphericity Assumed			
	Greenhouse-Geisser			
	Huynh-Feldt			
	Lower-bound			
Produkttyp * Messzeitpunkt	Sphericity Assumed	,333	,566	,005
	Greenhouse-Geisser	,333	,566	,005
	Huynh-Feldt	,333	,566	,005
	Lower-bound	,333	,566	,005
Error (Produkttyp*Messzeitpunkt)	Sphericity Assumed			
	Greenhouse-Geisser			
	Huynh-Feldt			
	Lower-bound			

Tests of Within-Subjects Contrasts

Measure: MEASURE_1

Source	Produkttyp	Messzeitpunkt	Type III Sum of Squares	df	Mean Square
Produkttyp	Linear		,088	1	,088
Error(Produkttyp)	Linear		3,615	63	,057
Messzeitpunkt		Linear	,030	1	,030
Error(Messzeitpunkt)		Linear	40,174	63	,638
Produkttyp * Messzeitpunkt	Linear	Linear	,020	1	,020
Error (Produkttyp*Messzeitpunkt)	Linear	Linear	3,746	63	,059

Tests of Within-Subjects Contrasts

Measure: MEASURE_1

Source	Produkttyp	Messzeitpunkt	F	Sig.	Partial Eta Squared
Produkttyp	Linear		1,536	,220	,024
Error(Produkttyp)	Linear				
Messzeitpunkt		Linear	,046	,830	,001
Error(Messzeitpunkt)		Linear			
Produkttyp * Messzeitpunkt	Linear	Linear	,333	,566	,005
Error (Produkttyp*Messzeitpunk t)	Linear	Linear			

Tests of Between-Subjects Effects

Measure: MEASURE_1

Transformed Variable: Average

Source	Type III Sum of Squares	df	Mean Square	F	Sig.	Partial Eta Squared
Intercept	1641,516	1	1641,516	2412,037	,000	,975
Error	42,875	63	,681			

Estimated Marginal Means

Produkttyp

Measure: MEASURE_1

Produkttyp	Mean	Std. Error	95% Confidence Interval	
			Lower Bound	Upper Bound
1	2,551	,047	2,457	2,644
2	2,514	,060	2,394	2,633

```
GLM Item2.AGlas.11.12.12 Item2.AGlas.23.01.13 Item2.APlastik.11.12.12 Item2.APlastik.23.01.1
   /WSFACTOR=Produkttyp 2 Polynomial Messzeitpunkt 2 Polynomial
   /METHOD=SSTYPE(3)
   /EMMEANS=TABLES(Produkttyp)
   /PRINT=DESCRIPTIVE ETASQ
   /CRITERIA=ALPHA(.05)
   /WSDESIGN=Produkttyp Messzeitpunkt Produkttyp*Messzeitpunkt.
```

General Linear Model

Notes

Output Created		07-NOV-2013 12:01:59
Comments		
Input	Data	C:\Documents and Settings\Dennis Boywitt\My Documents\My Dropbox\Freiberufliche Tätigkeit\Forschungsring\Daten\MDBF_Gruppe3_Items.sav
	Active Dataset	DataSet3
	Filter	<none>
	Weight	<none>
	Split File	<none>
	N of Rows in Working Data File	70
Missing Value Handling	Definition of Missing	User-defined missing values are treated as missing.
	Cases Used	Statistics are based on all cases with valid data for all variables in the model.
Syntax		GLM Item2.AGlas. 11.12.12 Item2.AGlas. 23.01.13 Item2.APlastik. 11.12.12 Item2.APlastik. 23.01.13<br><br>/WSFACTOR=Produkttyp 2 Polynomial Messzeitpunkt 2 Polynomial<br>/METHOD=SSTYPE(3)<br>/EMMEANS=TABLES (Produkttyp)<br>/PRINT=DESCRIPTIVE ETASQ<br>/CRITERIA=ALPHA(.05)<br>/WSDESIGN=Produkttyp Messzeitpunkt ...
Resources	Processor Time	00:00:00,03
	Elapsed Time	00:00:00,03

```
[DataSet3] C:\Documents and Settings\Dennis Boywitt\My Documents\My Dropbo
x\Freiberufliche Tätigkeit\Forschungsring\Daten\MDBF_Gruppe3_Items.sav
```

Within-Subjects Factors

Measure: MEASURE_1

Produkttyp	Messzeitpunkt	Dependent Variable
1	1	Item2.AGlas.11.12.12
	2	Item2.AGlas.23.01.13
2	1	Item2.APlastik.11.12.12
	2	Item2.APlastik.23.01.13

Descriptive Statistics

	Mean	Std. Deviation	N
Item2.AGlas.11.12.12	2,67	,416	64
Item2.AGlas.23.01.13	2,54	,755	64
Item2.APlastik.11.12.12	2,63	,531	64
Item2.APlastik.23.01.13	2,51	,737	64

Multivariate Tests[a]

Effect		Value	F	Hypothesis df	Error df
Produkttyp	Pillai's Trace	,018	1,134[b]	1,000	63,000
	Wilks' Lambda	,982	1,134[b]	1,000	63,000
	Hotelling's Trace	,018	1,134[b]	1,000	63,000
	Roy's Largest Root	,018	1,134[b]	1,000	63,000
Messzeitpunkt	Pillai's Trace	,029	1,849[b]	1,000	63,000
	Wilks' Lambda	,971	1,849[b]	1,000	63,000
	Hotelling's Trace	,029	1,849[b]	1,000	63,000
	Roy's Largest Root	,029	1,849[b]	1,000	63,000
Produkttyp * Messzeitpunkt	Pillai's Trace	,000	,002[b]	1,000	63,000
	Wilks' Lambda	1,000	,002[b]	1,000	63,000
	Hotelling's Trace	,000	,002[b]	1,000	63,000
	Roy's Largest Root	,000	,002[b]	1,000	63,000

Multivariate Tests[a]

Effect		Sig.	Partial Eta Squared
Produkttyp	Pillai's Trace	,291	,018
	Wilks' Lambda	,291	,018
	Hotelling's Trace	,291	,018
	Roy's Largest Root	,291	,018
Messzeitpunkt	Pillai's Trace	,179	,029
	Wilks' Lambda	,179	,029
	Hotelling's Trace	,179	,029
	Roy's Largest Root	,179	,029
Produkttyp * Messzeitpunkt	Pillai's Trace	,963	,000
	Wilks' Lambda	,963	,000
	Hotelling's Trace	,963	,000
	Roy's Largest Root	,963	,000

a. Design: Intercept
Within Subjects Design: Produkttyp + Messzeitpunkt + Produkttyp * Messzeitpunkt

b. Exact statistic

Mauchly's Test of Sphericity[a]

Measure: MEASURE_1

Within Subjects Effect	Mauchly's W	Approx. Chi-Square	df	Sig.	Epsilon[b]
					Greenhouse-Geisser
Produkttyp	1,000	,000	0	.	1,000
Messzeitpunkt	1,000	,000	0	.	1,000
Produkttyp * Messzeitpunkt	1,000	,000	0	.	1,000

Mauchly's Test of Sphericity[a]

Measure: MEASURE_1

Within Subjects Effect	Epsilon[b]	
	Huynh-Feldt	Lower-bound
Produkttyp	1,000	1,000
Messzeitpunkt	1,000	1,000
Produkttyp * Messzeitpunkt	1,000	1,000

Tests the null hypothesis that the error covariance matrix of the orthonormalized transformed dependent variables is proportional to an identity matrix.

a. Design: Intercept
Within Subjects Design: Produkttyp + Messzeitpunkt + Produkttyp * Messzeitpunkt

b. May be used to adjust the degrees of freedom for the averaged tests of significance. Corrected tests are displayed in the Tests of Within-Subjects Effects table.

Tests of Within-Subjects Effects

Measure: MEASURE_1

Source		Type III Sum of Squares	df	Mean Square
Produkttyp	Sphericity Assumed	,088	1	,088
	Greenhouse-Geisser	,088	1,000	,088
	Huynh-Feldt	,088	1,000	,088
	Lower-bound	,088	1,000	,088
Error(Produkttyp)	Sphericity Assumed	4,896	63	,078
	Greenhouse-Geisser	4,896	63,000	,078
	Huynh-Feldt	4,896	63,000	,078
	Lower-bound	4,896	63,000	,078
Messzeitpunkt	Sphericity Assumed	,969	1	,969
	Greenhouse-Geisser	,969	1,000	,969
	Huynh-Feldt	,969	1,000	,969
	Lower-bound	,969	1,000	,969
Error(Messzeitpunkt)	Sphericity Assumed	33,015	63	,524
	Greenhouse-Geisser	33,015	63,000	,524
	Huynh-Feldt	33,015	63,000	,524
	Lower-bound	33,015	63,000	,524
Produkttyp * Messzeitpunkt	Sphericity Assumed	,000	1	,000
	Greenhouse-Geisser	,000	1,000	,000
	Huynh-Feldt	,000	1,000	,000
	Lower-bound	,000	1,000	,000
Error (Produkttyp*Messzeitpunkt)	Sphericity Assumed	6,922	63	,110
	Greenhouse-Geisser	6,922	63,000	,110
	Huynh-Feldt	6,922	63,000	,110
	Lower-bound	6,922	63,000	,110

Tests of Within-Subjects Effects

Measure: MEASURE_1

Source		F	Sig.	Partial Eta Squared
Produkttyp	Sphericity Assumed	1,134	,291	,018
	Greenhouse-Geisser	1,134	,291	,018
	Huynh-Feldt	1,134	,291	,018
	Lower-bound	1,134	,291	,018
Error(Produkttyp)	Sphericity Assumed			
	Greenhouse-Geisser			
	Huynh-Feldt			
	Lower-bound			
Messzeitpunkt	Sphericity Assumed	1,849	,179	,029
	Greenhouse-Geisser	1,849	,179	,029
	Huynh-Feldt	1,849	,179	,029
	Lower-bound	1,849	,179	,029
Error(Messzeitpunkt)	Sphericity Assumed			
	Greenhouse-Geisser			
	Huynh-Feldt			
	Lower-bound			
Produkttyp * Messzeitpunkt	Sphericity Assumed	,002	,963	,000
	Greenhouse-Geisser	,002	,963	,000
	Huynh-Feldt	,002	,963	,000
	Lower-bound	,002	,963	,000
Error (Produkttyp*Messzeitpunkt)	Sphericity Assumed			
	Greenhouse-Geisser			
	Huynh-Feldt			
	Lower-bound			

Tests of Within-Subjects Contrasts

Measure: MEASURE_1

Source	Produkttyp	Messzeitpunkt	Type III Sum of Squares	df	Mean Square
Produkttyp	Linear		,088	1	,088
Error(Produkttyp)	Linear		4,896	63	,078
Messzeitpunkt		Linear	,969	1	,969
Error(Messzeitpunkt)		Linear	33,015	63	,524
Produkttyp * Messzeitpunkt	Linear	Linear	,000	1	,000
Error (Produkttyp*Messzeitpunkt)	Linear	Linear	6,922	63	,110

Tests of Within-Subjects Contrasts

Measure: MEASURE_1

Source	Produkttyp	Messzeitpunkt	F	Sig.	Partial Eta Squared
Produkttyp	Linear		1,134	,291	,018
Error(Produkttyp)	Linear				
Messzeitpunkt		Linear	1,849	,179	,029
Error(Messzeitpunkt)		Linear			
Produkttyp * Messzeitpunkt	Linear	Linear	,002	,963	,000
Error (Produkttyp*Messzeitpunk t)	Linear	Linear			

Tests of Between-Subjects Effects

Measure: MEASURE_1

Transformed Variable: Average

Source	Type III Sum of Squares	df	Mean Square	F	Sig.	Partial Eta Squared
Intercept	1713,184	1	1713,184	1999,157	,000	,969
Error	53,988	63	,857			

Estimated Marginal Means

Produkttyp

Measure: MEASURE_1

Produkttyp	Mean	Std. Error	95% Confidence Interval	
			Lower Bound	Upper Bound
1	2,605	,054	2,498	2,713
2	2,568	,067	2,435	2,701

```
GLM Item3.AGlas.11.12.12 Item3.AGlas.23.01.13 Item3.APlastik.11.12.12 Item3.APlastik.23.01.1
  /WSFACTOR=Produkttyp 2 Polynomial Messzeitpunkt 2 Polynomial
  /METHOD=SSTYPE(3)
  /EMMEANS=TABLES(Produkttyp)
  /PRINT=DESCRIPTIVE ETASQ
  /CRITERIA=ALPHA(.05)
  /WSDESIGN=Produkttyp Messzeitpunkt Produkttyp*Messzeitpunkt.
```

General Linear Model

Notes

Output Created		07-NOV-2013 12:02:31
Comments		
Input	Data	C:\Documents and Settings\Dennis Boywitt\My Documents\My Dropbox\Freiberufliche Tätigkeit\Forschungsring\Daten\MDBF_Gruppe3_Items.sav
	Active Dataset	DataSet3
	Filter	<none>
	Weight	<none>
	Split File	<none>
	N of Rows in Working Data File	70
Missing Value Handling	Definition of Missing	User-defined missing values are treated as missing.
	Cases Used	Statistics are based on all cases with valid data for all variables in the model.
Syntax		GLM Item3.AGlas.11.12.12 Item3.AGlas.23.01.13 Item3.APlastik.11.12.12 Item3.APlastik.23.01.13<br><br>/WSFACTOR=Produkttyp 2 Polynomial Messzeitpunkt 2 Polynomial<br>/METHOD=SSTYPE(3)<br>/EMMEANS=TABLES(Produkttyp)<br>/PRINT=DESCRIPTIVE ETASQ<br>/CRITERIA=ALPHA(.05)<br>/WSDESIGN=Produkttyp Messzeitpunkt ...
Resources	Processor Time	00:00:00,02
	Elapsed Time	00:00:00,03

```
[DataSet3] C:\Documents and Settings\Dennis Boywitt\My Documents\My Dropbo
x\Freiberufliche Tätigkeit\Forschungsring\Daten\MDBF_Gruppe3_Items.sav
```

Within-Subjects Factors

Measure: MEASURE_1

Produkttyp	Messzeitpunkt	Dependent Variable
1	1	Item3.AGlas.11.12.12
	2	Item3.AGlas.23.01.13
2	1	Item3.APlastik.11.12.12
	2	Item3.APlastik.23.01.13

Descriptive Statistics

	Mean	Std. Deviation	N
Item3.AGlas.11.12.12	2,74	,467	60
Item3.AGlas.23.01.13	2,82	,441	60
Item3.APlastik.11.12.12	2,73	,427	60
Item3.APlastik.23.01.13	2,80	,471	60

Multivariate Tests[a]

Effect		Value	F	Hypothesis df	Error df
Produkttyp	Pillai's Trace	,003	,187[b]	1,000	59,000
	Wilks' Lambda	,997	,187[b]	1,000	59,000
	Hotelling's Trace	,003	,187[b]	1,000	59,000
	Roy's Largest Root	,003	,187[b]	1,000	59,000
Messzeitpunkt	Pillai's Trace	,019	1,114[b]	1,000	59,000
	Wilks' Lambda	,981	1,114[b]	1,000	59,000
	Hotelling's Trace	,019	1,114[b]	1,000	59,000
	Roy's Largest Root	,019	1,114[b]	1,000	59,000
Produkttyp * Messzeitpunkt	Pillai's Trace	,001	,040[b]	1,000	59,000
	Wilks' Lambda	,999	,040[b]	1,000	59,000
	Hotelling's Trace	,001	,040[b]	1,000	59,000
	Roy's Largest Root	,001	,040[b]	1,000	59,000

Multivariate Tests[a]

Effect		Sig.	Partial Eta Squared
Produkttyp	Pillai's Trace	,667	,003
	Wilks' Lambda	,667	,003
	Hotelling's Trace	,667	,003
	Roy's Largest Root	,667	,003
Messzeitpunkt	Pillai's Trace	,295	,019
	Wilks' Lambda	,295	,019
	Hotelling's Trace	,295	,019
	Roy's Largest Root	,295	,019
Produkttyp * Messzeitpunkt	Pillai's Trace	,843	,001
	Wilks' Lambda	,843	,001
	Hotelling's Trace	,843	,001
	Roy's Largest Root	,843	,001

a. Design: Intercept
Within Subjects Design: Produkttyp + Messzeitpunkt + Produkttyp * Messzeitpunkt

b. Exact statistic

Mauchly's Test of Sphericity[a]

Measure: MEASURE_1

Within Subjects Effect	Mauchly's W	Approx. Chi-Square	df	Sig.	Epsilon[b] Greenhouse-Geisser
Produkttyp	1,000	,000	0	.	1,000
Messzeitpunkt	1,000	,000	0	.	1,000
Produkttyp * Messzeitpunkt	1,000	,000	0	.	1,000

Mauchly's Test of Sphericity[a]

Measure: MEASURE_1

Within Subjects Effect	Epsilon[b]	
	Huynh-Feldt	Lower-bound
Produkttyp	1,000	1,000
Messzeitpunkt	1,000	1,000
Produkttyp * Messzeitpunkt	1,000	1,000

Tests the null hypothesis that the error covariance matrix of the orthonormalized transformed dependent variables is proportional to an identity matrix.

a. Design: Intercept
Within Subjects Design: Produkttyp + Messzeitpunkt + Produkttyp * Messzeitpunkt

b. May be used to adjust the degrees of freedom for the averaged tests of significance. Corrected tests are displayed in the Tests of Within-Subjects Effects table.

Tests of Within-Subjects Effects

Measure: MEASURE_1

Source		Type III Sum of Squares	df	Mean Square
Produkttyp	Sphericity Assumed	,013	1	,013
	Greenhouse-Geisser	,013	1,000	,013
	Huynh-Feldt	,013	1,000	,013
	Lower-bound	,013	1,000	,013
Error(Produkttyp)	Sphericity Assumed	4,034	59	,068
	Greenhouse-Geisser	4,034	59,000	,068
	Huynh-Feldt	4,034	59,000	,068
	Lower-bound	4,034	59,000	,068
Messzeitpunkt	Sphericity Assumed	,284	1	,284
	Greenhouse-Geisser	,284	1,000	,284
	Huynh-Feldt	,284	1,000	,284
	Lower-bound	,284	1,000	,284
Error(Messzeitpunkt)	Sphericity Assumed	15,013	59	,254
	Greenhouse-Geisser	15,013	59,000	,254
	Huynh-Feldt	15,013	59,000	,254
	Lower-bound	15,013	59,000	,254
Produkttyp * Messzeitpunkt	Sphericity Assumed	,002	1	,002
	Greenhouse-Geisser	,002	1,000	,002
	Huynh-Feldt	,002	1,000	,002
	Lower-bound	,002	1,000	,002
Error (Produkttyp*Messzeitpunkt)	Sphericity Assumed	3,482	59	,059
	Greenhouse-Geisser	3,482	59,000	,059
	Huynh-Feldt	3,482	59,000	,059
	Lower-bound	3,482	59,000	,059

Tests of Within-Subjects Effects

Measure: MEASURE_1

Source		F	Sig.	Partial Eta Squared
Produkttyp	Sphericity Assumed	,187	,667	,003
	Greenhouse-Geisser	,187	,667	,003
	Huynh-Feldt	,187	,667	,003
	Lower-bound	,187	,667	,003
Error(Produkttyp)	Sphericity Assumed			
	Greenhouse-Geisser			
	Huynh-Feldt			
	Lower-bound			
Messzeitpunkt	Sphericity Assumed	1,114	,295	,019
	Greenhouse-Geisser	1,114	,295	,019
	Huynh-Feldt	1,114	,295	,019
	Lower-bound	1,114	,295	,019
Error(Messzeitpunkt)	Sphericity Assumed			
	Greenhouse-Geisser			
	Huynh-Feldt			
	Lower-bound			
Produkttyp * Messzeitpunkt	Sphericity Assumed	,040	,843	,001
	Greenhouse-Geisser	,040	,843	,001
	Huynh-Feldt	,040	,843	,001
	Lower-bound	,040	,843	,001
Error (Produkttyp*Messzeitpunkt)	Sphericity Assumed			
	Greenhouse-Geisser			
	Huynh-Feldt			
	Lower-bound			

Tests of Within-Subjects Contrasts

Measure: MEASURE_1

Source	Produkttyp	Messzeitpunkt	Type III Sum of Squares	df	Mean Square
Produkttyp	Linear		,013	1	,013
Error(Produkttyp)	Linear		4,034	59	,068
Messzeitpunkt		Linear	,284	1	,284
Error(Messzeitpunkt)		Linear	15,013	59	,254
Produkttyp * Messzeitpunkt	Linear	Linear	,002	1	,002
Error (Produkttyp*Messzeitpunkt)	Linear	Linear	3,482	59	,059

Tests of Within-Subjects Contrasts

Measure: MEASURE_1

Source	Produkttyp	Messzeitpunkt	F	Sig.	Partial Eta Squared
Produkttyp	Linear		,187	,667	,003
Error(Produkttyp)	Linear				
Messzeitpunkt		Linear	1,114	,295	,019
Error(Messzeitpunkt)		Linear			
Produkttyp * Messzeitpunkt	Linear	Linear	,040	,843	,001
Error (Produkttyp*Messzeitpunkt)	Linear	Linear			

Tests of Between-Subjects Effects

Measure: MEASURE_1

Transformed Variable: Average

Source	Type III Sum of Squares	df	Mean Square	F	Sig.	Partial Eta Squared
Intercept	1843,990	1	1843,990	4246,580	,000	,986
Error	25,620	59	,434			

Estimated Marginal Means

Produkttyp

Measure: MEASURE_1

Produkttyp	Mean	Std. Error	95% Confidence Interval	
			Lower Bound	Upper Bound
1	2,779	,044	2,691	2,867
2	2,765	,048	2,669	2,860
