# Supplementary material for: Development and Application of a Test for Food-Induced Emotions
Source: PLoS One. 2016 Nov 18;11(11):e0165991. doi: 10.1371/journal.pone.0165991 (PMC5115674; doi:10.1371/journal.pone.0165991)
Supplement: S3 File — (PDF) [file pone.0165991.s006.pdf]

```
GLM Item1.Naturastar.12.12.12 Item1.Naturastar.24.01.13 Item1.Goldblume.12.12.12 Item1.Goldb
  /WSFACTOR=Produkttyp 2 Polynomial Messzeitpunkt 2 Polynomial
  /METHOD=SSTYPE(3)
  /EMMEANS=TABLES(Produkttyp)
  /PRINT=DESCRIPTIVE ETASQ
  /CRITERIA=ALPHA(.05)
  /WSDESIGN=Produkttyp Messzeitpunkt Produkttyp*Messzeitpunkt.
```

General Linear Model

Notes

Output Created		07-NOV-2013 12:13:04
Comments		
Input	Data	C:\Documents and Settings\Dennis Boywitt\My Documents\My Dropbox\Freiberufliche Tätigkeit\Forschungsring\Daten\MDBF_Gruppe3_Items.sav
	Active Dataset	DataSet3
	Filter	<none>
	Weight	<none>
	Split File	<none>
	N of Rows in Working Data File	70
Missing Value Handling	Definition of Missing	User-defined missing values are treated as missing.
	Cases Used	Statistics are based on all cases with valid data for all variables in the model.
Syntax		GLM Item1.Naturastar.12.12.12 Item1.Naturastar.24.01.13 Item1.Goldblume.12.12.12 Item1.Goldblume.24.01.13 /WSFACTOR=Produkttyp 2 Polynomial Messzeitpunkt 2 Polynomial /METHOD=SSTYPE(3) /EMMEANS=TABLES(Produkttyp) /PRINT=DESCRIPTIVE ETASQ /CRITERIA=ALPHA(.05) /WSDESIGN=Produkttyp Messzeitpunkt Produkttyp*Messzeitpunkt.

Notes

Resources	Processor Time	00:00:00,03
	Elapsed Time	00:00:00,05

```
[DataSet3] C:\Documents and Settings\Dennis Boywitt\My Documents\My Dropbo
x\Freiberufliche Tätigkeit\Forschungsring\Daten\MDBF_Gruppe3_Items.sav
```

Within-Subjects Factors

Measure: MEASURE_1

Produkttyp	Messzeitpunkt	Dependent Variable
1	1	Item1. Naturastar. 12.12.12
	2	Item1. Naturastar. 24.01.13
2	1	Item1. Goldblume. 12.12.12
	2	Item1. Goldblume. 24.01.13

Descriptive Statistics

	Mean	Std. Deviation	N
Item1.Naturastar.12.12.12	2,58	,488	64
Item1.Naturastar.24.01.13	2,45	,781	64
Item1.Goldblume.12.12.12	2,56	,486	64
Item1.Goldblume.24.01.13	2,46	,787	64

Multivariate Tests[a]

Effect		Value	F	Hypothesis df	Error df
Produkttyp	Pillai's Trace	,000	,007[b]	1,000	63,000
	Wilks' Lambda	1,000	,007[b]	1,000	63,000
	Hotelling's Trace	,000	,007[b]	1,000	63,000
	Roy's Largest Root	,000	,007[b]	1,000	63,000
Messzeitpunkt	Pillai's Trace	,024	1,558[b]	1,000	63,000
	Wilks' Lambda	,976	1,558[b]	1,000	63,000
	Hotelling's Trace	,025	1,558[b]	1,000	63,000
	Roy's Largest Root	,025	1,558[b]	1,000	63,000
Produkttyp * Messzeitpunkt	Pillai's Trace	,005	,309[b]	1,000	63,000
	Wilks' Lambda	,995	,309[b]	1,000	63,000
	Hotelling's Trace	,005	,309[b]	1,000	63,000
	Roy's Largest Root	,005	,309[b]	1,000	63,000

Multivariate Tests[a]

Effect		Sig.	Partial Eta Squared
Produkttyp	Pillai's Trace	,935	,000
	Wilks' Lambda	,935	,000
	Hotelling's Trace	,935	,000
	Roy's Largest Root	,935	,000
Messzeitpunkt	Pillai's Trace	,217	,024
	Wilks' Lambda	,217	,024
	Hotelling's Trace	,217	,024
	Roy's Largest Root	,217	,024
Produkttyp * Messzeitpunkt	Pillai's Trace	,580	,005
	Wilks' Lambda	,580	,005
	Hotelling's Trace	,580	,005
	Roy's Largest Root	,580	,005

a. Design: Intercept
Within Subjects Design: Produkttyp + Messzeitpunkt + Produkttyp * Messzeitpunkt

b. Exact statistic

Mauchly's Test of Sphericity[a]

Measure: MEASURE_1

Within Subjects Effect	Mauchly's W	Approx. Chi-Square	df	Sig.	Epsilon[b] Greenhouse-Geisser
Produkttyp	1,000	,000	0	.	1,000
Messzeitpunkt	1,000	,000	0	.	1,000
Produkttyp * Messzeitpunkt	1,000	,000	0	.	1,000

Mauchly's Test of Sphericity[a]

Measure: MEASURE_1

Within Subjects Effect	Epsilon[b]	
	Huynh-Feldt	Lower-bound
Produkttyp	1,000	1,000
Messzeitpunkt	1,000	1,000
Produkttyp * Messzeitpunkt	1,000	1,000

Tests the null hypothesis that the error covariance matrix of the orthonormalized transformed dependent variables is proportional to an identity matrix.

a. Design: Intercept
Within Subjects Design: Produkttyp + Messzeitpunkt + Produkttyp * Messzeitpunkt

b. May be used to adjust the degrees of freedom for the averaged tests of significance. Corrected tests are displayed in the Tests of Within-Subjects Effects table.

Tests of Within-Subjects Effects

Measure: MEASURE_1

Source		Type III Sum of Squares	df	Mean Square
Produkttyp	Sphericity Assumed	,000	1	,000
	Greenhouse-Geisser	,000	1,000	,000
	Huynh-Feldt	,000	1,000	,000
	Lower-bound	,000	1,000	,000
Error(Produkttyp)	Sphericity Assumed	2,265	63	,036
	Greenhouse-Geisser	2,265	63,000	,036
	Huynh-Feldt	2,265	63,000	,036
	Lower-bound	2,265	63,000	,036
Messzeitpunkt	Sphericity Assumed	,908	1	,908
	Greenhouse-Geisser	,908	1,000	,908
	Huynh-Feldt	,908	1,000	,908
	Lower-bound	,908	1,000	,908
Error(Messzeitpunkt)	Sphericity Assumed	36,732	63	,583
	Greenhouse-Geisser	36,732	63,000	,583
	Huynh-Feldt	36,732	63,000	,583
	Lower-bound	36,732	63,000	,583
Produkttyp * Messzeitpunkt	Sphericity Assumed	,012	1	,012
	Greenhouse-Geisser	,012	1,000	,012
	Huynh-Feldt	,012	1,000	,012
	Lower-bound	,012	1,000	,012
Error (Produkttyp*Messzeitpunkt)	Sphericity Assumed	2,441	63	,039
	Greenhouse-Geisser	2,441	63,000	,039
	Huynh-Feldt	2,441	63,000	,039
	Lower-bound	2,441	63,000	,039

Tests of Within-Subjects Effects

Measure: MEASURE_1

Source		F	Sig.	Partial Eta Squared
Produkttyp	Sphericity Assumed	,007	,935	,000
	Greenhouse-Geisser	,007	,935	,000
	Huynh-Feldt	,007	,935	,000
	Lower-bound	,007	,935	,000
Error(Produkttyp)	Sphericity Assumed			
	Greenhouse-Geisser			
	Huynh-Feldt			
	Lower-bound			
Messzeitpunkt	Sphericity Assumed	1,558	,217	,024
	Greenhouse-Geisser	1,558	,217	,024
	Huynh-Feldt	1,558	,217	,024
	Lower-bound	1,558	,217	,024
Error(Messzeitpunkt)	Sphericity Assumed			
	Greenhouse-Geisser			
	Huynh-Feldt			
	Lower-bound			
Produkttyp * Messzeitpunkt	Sphericity Assumed	,309	,580	,005
	Greenhouse-Geisser	,309	,580	,005
	Huynh-Feldt	,309	,580	,005
	Lower-bound	,309	,580	,005
Error (Produkttyp*Messzeitpunkt)	Sphericity Assumed			
	Greenhouse-Geisser			
	Huynh-Feldt			
	Lower-bound			

Tests of Within-Subjects Contrasts

Measure: MEASURE_1

Source	Produkttyp	Messzeitpunkt	Type III Sum of Squares	df	Mean Square
Produkttyp	Linear		,000	1	,000
Error(Produkttyp)	Linear		2,265	63	,036
Messzeitpunkt		Linear	,908	1	,908
Error(Messzeitpunkt)		Linear	36,732	63	,583
Produkttyp * Messzeitpunkt	Linear	Linear	,012	1	,012
Error (Produkttyp*Messzeitpunkt)	Linear	Linear	2,441	63	,039

Tests of Within-Subjects Contrasts

Measure: MEASURE_1

Source	Produkttyp	Messzeitpunkt	F	Sig.	Partial Eta Squared
Produkttyp	Linear		,007	,935	,000
Error(Produkttyp)	Linear				
Messzeitpunkt		Linear	1,558	,217	,024
Error(Messzeitpunkt)		Linear			
Produkttyp * Messzeitpunkt	Linear	Linear	,309	,580	,005
Error (Produkttyp*Messzeitpunk t)	Linear	Linear			

Tests of Between-Subjects Effects

Measure: MEASURE_1

Transformed Variable: Average

Source	Type III Sum of Squares	df	Mean Square	F	Sig.	Partial Eta Squared
Intercept	1613,780	1	1613,780	1542,212	,000	,961
Error	65,924	63	1,046			

Estimated Marginal Means

Produkttyp

Measure: MEASURE_1

Produkttyp	Mean	Std. Error	95% Confidence Interval	
			Lower Bound	Upper Bound
1	2,512	,063	2,385	2,638
2	2,510	,067	2,376	2,643

```
GLM Item2.Naturastar.12.12.12 Item2.Naturastar.24.01.13 Item2.Goldblume.12.12.12 Item2.Goldb
  /WSFACTOR=Produkttyp 2 Polynomial Messzeitpunkt 2 Polynomial
  /METHOD=SSTYPE(3)
  /EMMEANS=TABLES(Produkttyp)
  /PRINT=DESCRIPTIVE ETASQ
  /CRITERIA=ALPHA(.05)
  /WSDESIGN=Produkttyp Messzeitpunkt Produkttyp*Messzeitpunkt.
```

General Linear Model

Notes

Output Created		07-NOV-2013 12:14:08
Comments		
Input	Data	C:\Documents and Settings\Dennis Boywitt\My Documents\My Dropbox\Freiberufliche Tätigkeit\Forschungsring\Daten\MDBF_Gruppe3_Items.sav
	Active Dataset	DataSet3
	Filter	<none>
	Weight	<none>
	Split File	<none>
	N of Rows in Working Data File	70
Missing Value Handling	Definition of Missing	User-defined missing values are treated as missing.
	Cases Used	Statistics are based on all cases with valid data for all variables in the model.
Syntax		GLM Item2.Naturastar.12.12.12 Item2.Naturastar.24.01.13 Item2.Goldblume.12.12.12 Item2.Goldblume.24.01.13 /WSFACTOR=Produkttyp 2 Polynomial Messzeitpunkt 2 Polynomial /METHOD=SSTYPE(3) /EMMEANS=TABLES(Produkttyp) /PRINT=DESCRIPTIVE ETASQ /CRITERIA=ALPHA(.05) /WSDESIGN=Produkttyp Messzeitpunkt Produkttyp*Messzeitpunkt.
Resources	Processor Time	00:00:00,03
	Elapsed Time	00:00:00,03

```
[DataSet3] C:\Documents and Settings\Dennis Boywitt\My Documents\My Dropbo
x\Freiberufliche Tätigkeit\Forschungsring\Daten\MDBF_Gruppe3_Items.sav
```

Within-Subjects Factors

Measure: MEASURE_1

Produkttyp	Messzeitpunkt	Dependent Variable
1	1	Item2. Naturastar. 12.12.12
	2	Item2. Naturastar. 24.01.13
2	1	Item2. Goldblume. 12.12.12
	2	Item2. Goldblume. 24.01.13

Descriptive Statistics

	Mean	Std. Deviation	N
Item2.Naturastar.12.12.12	2,59	,522	64
Item2.Naturastar.24.01.13	2,58	,798	64
Item2.Goldblume.12.12.12	2,61	,567	64
Item2.Goldblume.24.01.13	2,63	,803	64

Multivariate Tests[a]

Effect		Value	F	Hypothesis df	Error df
Produkttyp	Pillai's Trace	,046	3,028[b]	1,000	63,000
	Wilks' Lambda	,954	3,028[b]	1,000	63,000
	Hotelling's Trace	,048	3,028[b]	1,000	63,000
	Roy's Largest Root	,048	3,028[b]	1,000	63,000
Messzeitpunkt	Pillai's Trace	,000	,011[b]	1,000	63,000
	Wilks' Lambda	1,000	,011[b]	1,000	63,000
	Hotelling's Trace	,000	,011[b]	1,000	63,000
	Roy's Largest Root	,000	,011[b]	1,000	63,000
Produkttyp * Messzeitpunkt	Pillai's Trace	,005	,330[b]	1,000	63,000
	Wilks' Lambda	,995	,330[b]	1,000	63,000
	Hotelling's Trace	,005	,330[b]	1,000	63,000
	Roy's Largest Root	,005	,330[b]	1,000	63,000

Multivariate Tests[a]

Effect		Sig.	Partial Eta Squared
Produkttyp	Pillai's Trace	,087	,046
	Wilks' Lambda	,087	,046
	Hotelling's Trace	,087	,046
	Roy's Largest Root	,087	,046
Messzeitpunkt	Pillai's Trace	,918	,000
	Wilks' Lambda	,918	,000
	Hotelling's Trace	,918	,000
	Roy's Largest Root	,918	,000
Produkttyp * Messzeitpunkt	Pillai's Trace	,568	,005
	Wilks' Lambda	,568	,005
	Hotelling's Trace	,568	,005
	Roy's Largest Root	,568	,005

a. Design: Intercept
Within Subjects Design: Produkttyp + Messzeitpunkt + Produkttyp * Messzeitpunkt

b. Exact statistic

Mauchly's Test of Sphericity[a]

Measure: MEASURE_1

Within Subjects Effect	Mauchly's W	Approx. Chi-Square	df	Sig.	Epsilon[b]
					Greenhouse-Geisser
Produkttyp	1,000	,000	0	.	1,000
Messzeitpunkt	1,000	,000	0	.	1,000
Produkttyp * Messzeitpunkt	1,000	,000	0	.	1,000

Mauchly's Test of Sphericity[a]

Measure: MEASURE_1

Within Subjects Effect	Epsilon[b]	
	Huynh-Feldt	Lower-bound
Produkttyp	1,000	1,000
Messzeitpunkt	1,000	1,000
Produkttyp * Messzeitpunkt	1,000	1,000

Tests the null hypothesis that the error covariance matrix of the orthonormalized transformed dependent variables is proportional to an identity matrix.

a. Design: Intercept
Within Subjects Design: Produkttyp + Messzeitpunkt + Produkttyp * Messzeitpunkt

b. May be used to adjust the degrees of freedom for the averaged tests of significance. Corrected tests are displayed in the Tests of Within-Subjects Effects table.

Tests of Within-Subjects Effects

Measure: MEASURE_1

Source		Type III Sum of Squares	df	Mean Square
Produkttyp	Sphericity Assumed	,088	1	,088
	Greenhouse-Geisser	,088	1,000	,088
	Huynh-Feldt	,088	1,000	,088
	Lower-bound	,088	1,000	,088
Error(Produkttyp)	Sphericity Assumed	1,834	63	,029
	Greenhouse-Geisser	1,834	63,000	,029
	Huynh-Feldt	1,834	63,000	,029
	Lower-bound	1,834	63,000	,029
Messzeitpunkt	Sphericity Assumed	,006	1	,006
	Greenhouse-Geisser	,006	1,000	,006
	Huynh-Feldt	,006	1,000	,006
	Lower-bound	,006	1,000	,006
Error(Messzeitpunkt)	Sphericity Assumed	35,666	63	,566
	Greenhouse-Geisser	35,666	63,000	,566
	Huynh-Feldt	35,666	63,000	,566
	Lower-bound	35,666	63,000	,566
Produkttyp * Messzeitpunkt	Sphericity Assumed	,012	1	,012
	Greenhouse-Geisser	,012	1,000	,012
	Huynh-Feldt	,012	1,000	,012
	Lower-bound	,012	1,000	,012
Error (Produkttyp*Messzeitpunkt)	Sphericity Assumed	2,285	63	,036
	Greenhouse-Geisser	2,285	63,000	,036
	Huynh-Feldt	2,285	63,000	,036
	Lower-bound	2,285	63,000	,036

Tests of Within-Subjects Effects

Measure: MEASURE_1

Source		F	Sig.	Partial Eta Squared
Produkttyp	Sphericity Assumed	3,028	,087	,046
	Greenhouse-Geisser	3,028	,087	,046
	Huynh-Feldt	3,028	,087	,046
	Lower-bound	3,028	,087	,046
Error(Produkttyp)	Sphericity Assumed			
	Greenhouse-Geisser			
	Huynh-Feldt			
	Lower-bound			
Messzeitpunkt	Sphericity Assumed	,011	,918	,000
	Greenhouse-Geisser	,011	,918	,000
	Huynh-Feldt	,011	,918	,000
	Lower-bound	,011	,918	,000
Error(Messzeitpunkt)	Sphericity Assumed			
	Greenhouse-Geisser			
	Huynh-Feldt			
	Lower-bound			
Produkttyp * Messzeitpunkt	Sphericity Assumed	,330	,568	,005
	Greenhouse-Geisser	,330	,568	,005
	Huynh-Feldt	,330	,568	,005
	Lower-bound	,330	,568	,005
Error (Produkttyp*Messzeitpunkt)	Sphericity Assumed			
	Greenhouse-Geisser			
	Huynh-Feldt			
	Lower-bound			

Tests of Within-Subjects Contrasts

Measure: MEASURE_1

Source	Produkttyp	Messzeitpunkt	Type III Sum of Squares	df	Mean Square
Produkttyp	Linear		,088	1	,088
Error(Produkttyp)	Linear		1,834	63	,029
Messzeitpunkt		Linear	,006	1	,006
Error(Messzeitpunkt)		Linear	35,666	63	,566
Produkttyp * Messzeitpunkt	Linear	Linear	,012	1	,012
Error (Produkttyp*Messzeitpunkt)	Linear	Linear	2,285	63	,036

Tests of Within-Subjects Contrasts

Measure: MEASURE_1

Source	Produkttyp	Messzeitpunkt	F	Sig.	Partial Eta Squared
Produkttyp	Linear		3,028	,087	,046
Error(Produkttyp)	Linear				
Messzeitpunkt		Linear	,011	,918	,000
Error(Messzeitpunkt)		Linear			
Produkttyp * Messzeitpunkt	Linear	Linear	,330	,568	,005
Error (Produkttyp*Messzeitpunk t)	Linear	Linear			

Tests of Between-Subjects Effects

Measure: MEASURE_1

Transformed Variable: Average

Source	Type III Sum of Squares	df	Mean Square	F	Sig.	Partial Eta Squared
Intercept	1733,942	1	1733,942	1394,142	,000	,957
Error	78,355	63	1,244			

Estimated Marginal Means

Produkttyp

Measure: MEASURE_1

Produkttyp	Mean	Std. Error	95% Confidence Interval	
			Lower Bound	Upper Bound
1	2,584	,070	2,444	2,724
2	2,621	,071	2,479	2,763

```
GLM Item3.Naturastar.12.12.12 Item3.Naturastar.24.01.13 Item3.Goldblume.12.12.12 Item3.Goldb
  /WSFACTOR=Produkttyp 2 Polynomial Messzeitpunkt 2 Polynomial
  /METHOD=SSTYPE(3)
  /EMMEANS=TABLES(Produkttyp)
  /PRINT=DESCRIPTIVE ETASQ
  /CRITERIA=ALPHA(.05)
  /WSDESIGN=Produkttyp Messzeitpunkt Produkttyp*Messzeitpunkt.
```

General Linear Model

Notes

Output Created		07-NOV-2013 12:15:41
Comments		
Input	Data	C:\Documents and Settings\Dennis Boywitt\My Documents\My Dropbox\Freiberufliche Tätigkeit\Forschungsring\Daten\MDBF_Gruppe3_Items.sav
	Active Dataset	DataSet3
	Filter	<none>
	Weight	<none>
	Split File	<none>
	N of Rows in Working Data File	70
Missing Value Handling	Definition of Missing	User-defined missing values are treated as missing.
	Cases Used	Statistics are based on all cases with valid data for all variables in the model.
Syntax		GLM Item3.Naturastar.12.12.12 Item3.Naturastar.24.01.13 Item3.Goldblume.12.12.12 Item3.Goldblume.24.01.13 /WSFACTOR=Produkttyp 2 Polynomial Messzeitpunkt 2 Polynomial /METHOD=SSTYPE(3) /EMMEANS=TABLES(Produkttyp) /PRINT=DESCRIPTIVE ETASQ /CRITERIA=ALPHA(.05) /WSDESIGN=Produkttyp Messzeitpunkt Produkttyp*Messzeitpunkt.
Resources	Processor Time	00:00:00,03
	Elapsed Time	00:00:00,03

```
[DataSet3] C:\Documents and Settings\Dennis Boywitt\My Documents\My Dropbo
x\Freiberufliche Tätigkeit\Forschungsring\Daten\MDBF_Gruppe3_Items.sav
```

Within-Subjects Factors

Measure: MEASURE_1

Produkttyp	Messzeitpunkt	Dependent Variable
1	1	Item3. Naturastar. 12.12.12
	2	Item3. Naturastar. 24.01.13
2	1	Item3. Goldblume. 12.12.12
	2	Item3. Goldblume. 24.01.13

Descriptive Statistics

	Mean	Std. Deviation	N
Item3.Naturastar.12.12.12	2,75	,452	60
Item3.Naturastar.24.01.13	2,73	,453	60
Item3.Goldblume.12.12.12	2,75	,479	60
Item3.Goldblume.24.01.13	2,74	,469	60

Multivariate Tests[a]

Effect		Value	F	Hypothesis df	Error df
Produkttyp	Pillai's Trace	,000	,026[b]	1,000	59,000
	Wilks' Lambda	1,000	,026[b]	1,000	59,000
	Hotelling's Trace	,000	,026[b]	1,000	59,000
	Roy's Largest Root	,000	,026[b]	1,000	59,000
Messzeitpunkt	Pillai's Trace	,000	,016[b]	1,000	59,000
	Wilks' Lambda	1,000	,016[b]	1,000	59,000
	Hotelling's Trace	,000	,016[b]	1,000	59,000
	Roy's Largest Root	,000	,016[b]	1,000	59,000
Produkttyp * Messzeitpunkt	Pillai's Trace	,000	,012[b]	1,000	59,000
	Wilks' Lambda	1,000	,012[b]	1,000	59,000
	Hotelling's Trace	,000	,012[b]	1,000	59,000
	Roy's Largest Root	,000	,012[b]	1,000	59,000

Multivariate Tests[a]

Effect		Sig.	Partial Eta Squared
Produkttyp	Pillai's Trace	,874	,000
	Wilks' Lambda	,874	,000
	Hotelling's Trace	,874	,000
	Roy's Largest Root	,874	,000
Messzeitpunkt	Pillai's Trace	,901	,000
	Wilks' Lambda	,901	,000
	Hotelling's Trace	,901	,000
	Roy's Largest Root	,901	,000
Produkttyp * Messzeitpunkt	Pillai's Trace	,914	,000
	Wilks' Lambda	,914	,000
	Hotelling's Trace	,914	,000
	Roy's Largest Root	,914	,000

a. Design: Intercept
Within Subjects Design: Produkttyp + Messzeitpunkt + Produkttyp * Messzeitpunkt

b. Exact statistic

Mauchly's Test of Sphericity[a]

Measure: MEASURE_1

Within Subjects Effect	Mauchly's W	Approx. Chi-Square	df	Sig.	Epsilon[b]
					Greenhouse-Geisser
Produkttyp	1,000	,000	0	.	1,000
Messzeitpunkt	1,000	,000	0	.	1,000
Produkttyp * Messzeitpunkt	1,000	,000	0	.	1,000

Mauchly's Test of Sphericity[a]

Measure: MEASURE_1

Within Subjects Effect	Epsilon[b]	
	Huynh-Feldt	Lower-bound
Produkttyp	1,000	1,000
Messzeitpunkt	1,000	1,000
Produkttyp * Messzeitpunkt	1,000	1,000

Tests the null hypothesis that the error covariance matrix of the orthonormalized transformed dependent variables is proportional to an identity matrix.

a. Design: Intercept
Within Subjects Design: Produkttyp + Messzeitpunkt + Produkttyp * Messzeitpunkt

b. May be used to adjust the degrees of freedom for the averaged tests of significance. Corrected tests are displayed in the Tests of Within-Subjects Effects table.

Tests of Within-Subjects Effects

Measure: MEASURE_1

Source		Type III Sum of Squares	df	Mean Square
Produkttyp	Sphericity Assumed	,001	1	,001
	Greenhouse-Geisser	,001	1,000	,001
	Huynh-Feldt	,001	1,000	,001
	Lower-bound	,001	1,000	,001
Error(Produkttyp)	Sphericity Assumed	2,405	59	,041
	Greenhouse-Geisser	2,405	59,000	,041
	Huynh-Feldt	2,405	59,000	,041
	Lower-bound	2,405	59,000	,041
Messzeitpunkt	Sphericity Assumed	,004	1	,004
	Greenhouse-Geisser	,004	1,000	,004
	Huynh-Feldt	,004	1,000	,004
	Lower-bound	,004	1,000	,004
Error(Messzeitpunkt)	Sphericity Assumed	15,715	59	,266
	Greenhouse-Geisser	15,715	59,000	,266
	Huynh-Feldt	15,715	59,000	,266
	Lower-bound	15,715	59,000	,266
Produkttyp * Messzeitpunkt	Sphericity Assumed	,001	1	,001
	Greenhouse-Geisser	,001	1,000	,001
	Huynh-Feldt	,001	1,000	,001
	Lower-bound	,001	1,000	,001
Error (Produkttyp*Messzeitpunkt)	Sphericity Assumed	5,218	59	,088
	Greenhouse-Geisser	5,218	59,000	,088
	Huynh-Feldt	5,218	59,000	,088
	Lower-bound	5,218	59,000	,088

Tests of Within-Subjects Effects

Measure: MEASURE_1

Source		F	Sig.	Partial Eta Squared
Produkttyp	Sphericity Assumed	,026	,874	,000
	Greenhouse-Geisser	,026	,874	,000
	Huynh-Feldt	,026	,874	,000
	Lower-bound	,026	,874	,000
Error(Produkttyp)	Sphericity Assumed			
	Greenhouse-Geisser			
	Huynh-Feldt			
	Lower-bound			
Messzeitpunkt	Sphericity Assumed	,016	,901	,000
	Greenhouse-Geisser	,016	,901	,000
	Huynh-Feldt	,016	,901	,000
	Lower-bound	,016	,901	,000
Error(Messzeitpunkt)	Sphericity Assumed			
	Greenhouse-Geisser			
	Huynh-Feldt			
	Lower-bound			
Produkttyp * Messzeitpunkt	Sphericity Assumed	,012	,914	,000
	Greenhouse-Geisser	,012	,914	,000
	Huynh-Feldt	,012	,914	,000
	Lower-bound	,012	,914	,000
Error (Produkttyp*Messzeitpunkt)	Sphericity Assumed			
	Greenhouse-Geisser			
	Huynh-Feldt			
	Lower-bound			

Tests of Within-Subjects Contrasts

Measure: MEASURE_1

Source	Produkttyp	Messzeitpunkt	Type III Sum of Squares	df	Mean Square
Produkttyp	Linear		,001	1	,001
Error(Produkttyp)	Linear		2,405	59	,041
Messzeitpunkt		Linear	,004	1	,004
Error(Messzeitpunkt)		Linear	15,715	59	,266
Produkttyp * Messzeitpunkt	Linear	Linear	,001	1	,001
Error (Produkttyp*Messzeitpunkt)	Linear	Linear	5,218	59	,088

Tests of Within-Subjects Contrasts

Measure: MEASURE_1

Source	Produkttyp	Messzeitpunkt	F	Sig.	Partial Eta Squared
Produkttyp	Linear		,026	,874	,000
Error(Produkttyp)	Linear				
Messzeitpunkt		Linear	,016	,901	,000
Error(Messzeitpunkt)		Linear			
Produkttyp * Messzeitpunkt	Linear	Linear	,012	,914	,000
Error (Produkttyp*Messzeitpunkt)	Linear	Linear			

Tests of Between-Subjects Effects

Measure: MEASURE_1

Transformed Variable: Average

Source	Type III Sum of Squares	df	Mean Square	F	Sig.	Partial Eta Squared
Intercept	1804,017	1	1804,017	3886,039	,000	,985
Error	27,390	59	,464			

Estimated Marginal Means

Produkttyp

Measure: MEASURE_1

Produkttyp	Mean	Std. Error	95% Confidence Interval	
			Lower Bound	Upper Bound
1	2,740	,045	2,649	2,830
2	2,744	,047	2,651	2,837
