# Supplementary material for: Development and Application of a Test for Food-Induced Emotions
Source: PLoS One. 2016 Nov 18;11(11):e0165991. doi: 10.1371/journal.pone.0165991 (PMC5115674; doi:10.1371/journal.pone.0165991)
Supplement: S4 File — (PDF) [file pone.0165991.s007.pdf]

```
GLM Item1.Diamant.12.12.12 Item1.Diamant.24.01.13 Item1.Guarani.12.12.12 Item1.Guarani.24.01
  /WSFACTOR=Produkttyp 2 Polynomial Messzeitpunkt 2 Polynomial
  /METHOD=SSTYPE(3)
  /EMMEANS=TABLES(Produkttyp)
  /PRINT=DESCRIPTIVE ETASQ
  /CRITERIA=ALPHA(.05)
  /WSDESIGN=Produkttyp Messzeitpunkt Produkttyp*Messzeitpunkt.
```

General Linear Model

Notes

Output Created		07-NOV-2013 12:16:11
Comments		
Input	Data	C:\Documents and Settings\Dennis Boywitt\My Documents\My Dropbox\Freiberufliche Tätigkeit\Forschungsring\Daten\MDBF_Gruppe3_Items.sav
	Active Dataset	DataSet3
	Filter	<none>
	Weight	<none>
	Split File	<none>
	N of Rows in Working Data File	70
Missing Value Handling	Definition of Missing	User-defined missing values are treated as missing.
	Cases Used	Statistics are based on all cases with valid data for all variables in the model.
Syntax		GLM Item1.Diamant.12.12.12 Item1.Diamant.24.01.13 Item1.Guarani.12.12.12 Item1.Guarani.24.01.13<br>/WSFACTOR=Produkttyp 2 Polynomial Messzeitpunkt 2 Polynomial<br>/METHOD=SSTYPE(3)<br>/EMMEANS=TABLES(Produkttyp)<br>/PRINT=DESCRIPTIVE ETASQ<br>/CRITERIA=ALPHA(.05)<br>/WSDESIGN=Produkttyp Messzeitpunkt Produkttyp*Messzeitpunkt.

Notes

Resources	Processor Time	00:00:00,02
	Elapsed Time	00:00:00,03

```
[DataSet3] C:\Documents and Settings\Dennis Boywitt\My Documents\My Dropbo
x\Freiberufliche Tätigkeit\Forschungsring\Daten\MDBF_Gruppe3_Items.sav
```

Within-Subjects Factors

Measure: MEASURE_1

Produkttyp	Messzeitpunkt	Dependent Variable
1	1	Item1. Diamant. 12.12.12
	2	Item1. Diamant. 24.01.13
2	1	Item1. Guarani. 12.12.12
	2	Item1. Guarani. 24.01.13

Descriptive Statistics

	Mean	Std. Deviation	N
Item1.Diamant.12.12.12	2,69	,458	64
Item1.Diamant.24.01.13	2,59	,811	64
Item1.Guarani.12.12.12	2,69	,434	64
Item1.Guarani.24.01.13	2,54	,786	64

Multivariate Tests[a]

Effect		Value	F	Hypothesis df	Error df
Produkttyp	Pillai's Trace	,017	1,092[b]	1,000	63,000
	Wilks' Lambda	,983	1,092[b]	1,000	63,000
	Hotelling's Trace	,017	1,092[b]	1,000	63,000
	Roy's Largest Root	,017	1,092[b]	1,000	63,000
Messzeitpunkt	Pillai's Trace	,024	1,560[b]	1,000	63,000
	Wilks' Lambda	,976	1,560[b]	1,000	63,000
	Hotelling's Trace	,025	1,560[b]	1,000	63,000
	Roy's Largest Root	,025	1,560[b]	1,000	63,000
Produkttyp * Messzeitpunkt	Pillai's Trace	,014	,895[b]	1,000	63,000
	Wilks' Lambda	,986	,895[b]	1,000	63,000
	Hotelling's Trace	,014	,895[b]	1,000	63,000
	Roy's Largest Root	,014	,895[b]	1,000	63,000

Multivariate Tests[a]

Effect		Sig.	Partial Eta Squared
Produkttyp	Pillai's Trace	,300	,017
	Wilks' Lambda	,300	,017
	Hotelling's Trace	,300	,017
	Roy's Largest Root	,300	,017
Messzeitpunkt	Pillai's Trace	,216	,024
	Wilks' Lambda	,216	,024
	Hotelling's Trace	,216	,024
	Roy's Largest Root	,216	,024
Produkttyp * Messzeitpunkt	Pillai's Trace	,348	,014
	Wilks' Lambda	,348	,014
	Hotelling's Trace	,348	,014
	Roy's Largest Root	,348	,014

a. Design: Intercept
Within Subjects Design: Produkttyp + Messzeitpunkt + Produkttyp * Messzeitpunkt

b. Exact statistic

Mauchly's Test of Sphericity[a]

Measure: MEASURE_1

Within Subjects Effect	Mauchly's W	Approx. Chi-Square	df	Sig.	Epsilon[b]
					Greenhouse-Geisser
Produkttyp	1,000	,000	0	.	1,000
Messzeitpunkt	1,000	,000	0	.	1,000
Produkttyp * Messzeitpunkt	1,000	,000	0	.	1,000

Mauchly's Test of Sphericity[a]

Measure: MEASURE_1

Within Subjects Effect	Epsilon[b]	
	Huynh-Feldt	Lower-bound
Produkttyp	1,000	1,000
Messzeitpunkt	1,000	1,000
Produkttyp * Messzeitpunkt	1,000	1,000

Tests the null hypothesis that the error covariance matrix of the orthonormalized transformed dependent variables is proportional to an identity matrix.

a. Design: Intercept
Within Subjects Design: Produkttyp + Messzeitpunkt + Produkttyp * Messzeitpunkt

b. May be used to adjust the degrees of freedom for the averaged tests of significance. Corrected tests are displayed in the Tests of Within-Subjects Effects table.

Tests of Within-Subjects Effects

Measure: MEASURE_1

Source		Type III Sum of Squares	df	Mean Square
Produkttyp	Sphericity Assumed	,041	1	,041
	Greenhouse-Geisser	,041	1,000	,041
	Huynh-Feldt	,041	1,000	,041
	Lower-bound	,041	1,000	,041
Error(Produkttyp)	Sphericity Assumed	2,381	63	,038
	Greenhouse-Geisser	2,381	63,000	,038
	Huynh-Feldt	2,381	63,000	,038
	Lower-bound	2,381	63,000	,038
Messzeitpunkt	Sphericity Assumed	,969	1	,969
	Greenhouse-Geisser	,969	1,000	,969
	Huynh-Feldt	,969	1,000	,969
	Lower-bound	,969	1,000	,969
Error(Messzeitpunkt)	Sphericity Assumed	39,140	63	,621
	Greenhouse-Geisser	39,140	63,000	,621
	Huynh-Feldt	39,140	63,000	,621
	Lower-bound	39,140	63,000	,621
Produkttyp * Messzeitpunkt	Sphericity Assumed	,030	1	,030
	Greenhouse-Geisser	,030	1,000	,030
	Huynh-Feldt	,030	1,000	,030
	Lower-bound	,030	1,000	,030
Error (Produkttyp*Messzeitpunkt)	Sphericity Assumed	2,080	63	,033
	Greenhouse-Geisser	2,080	63,000	,033
	Huynh-Feldt	2,080	63,000	,033
	Lower-bound	2,080	63,000	,033

Tests of Within-Subjects Effects

Measure: MEASURE_1

Source		F	Sig.	Partial Eta Squared
Produkttyp	Sphericity Assumed	1,092	,300	,017
	Greenhouse-Geisser	1,092	,300	,017
	Huynh-Feldt	1,092	,300	,017
	Lower-bound	1,092	,300	,017
Error(Produkttyp)	Sphericity Assumed			
	Greenhouse-Geisser			
	Huynh-Feldt			
	Lower-bound			
Messzeitpunkt	Sphericity Assumed	1,560	,216	,024
	Greenhouse-Geisser	1,560	,216	,024
	Huynh-Feldt	1,560	,216	,024
	Lower-bound	1,560	,216	,024
Error(Messzeitpunkt)	Sphericity Assumed			
	Greenhouse-Geisser			
	Huynh-Feldt			
	Lower-bound			
Produkttyp * Messzeitpunkt	Sphericity Assumed	,895	,348	,014
	Greenhouse-Geisser	,895	,348	,014
	Huynh-Feldt	,895	,348	,014
	Lower-bound	,895	,348	,014
Error (Produkttyp*Messzeitpunkt)	Sphericity Assumed			
	Greenhouse-Geisser			
	Huynh-Feldt			
	Lower-bound			

Tests of Within-Subjects Contrasts

Measure: MEASURE_1

Source	Produkttyp	Messzeitpunkt	Type III Sum of Squares	df	Mean Square
Produkttyp	Linear		,041	1	,041
Error(Produkttyp)	Linear		2,381	63	,038
Messzeitpunkt		Linear	,969	1	,969
Error(Messzeitpunkt)		Linear	39,140	63	,621
Produkttyp * Messzeitpunkt	Linear	Linear	,030	1	,030
Error (Produkttyp*Messzeitpunkt)	Linear	Linear	2,080	63	,033

Tests of Within-Subjects Contrasts

Measure: MEASURE_1

Source	Produkttyp	Messzeitpunkt	F	Sig.	Partial Eta Squared
Produkttyp	Linear		1,092	,300	,017
Error(Produkttyp)	Linear				
Messzeitpunkt		Linear	1,560	,216	,024
Error(Messzeitpunkt)		Linear			
Produkttyp * Messzeitpunkt	Linear	Linear	,895	,348	,014
Error (Produkttyp*Messzeitpunkt)	Linear	Linear			

Tests of Between-Subjects Effects

Measure: MEASURE_1

Transformed Variable: Average

Source	Type III Sum of Squares	df	Mean Square	F	Sig.	Partial Eta Squared
Intercept	1767,940	1	1767,940	1800,603	,000	,966
Error	61,857	63	,982			

Estimated Marginal Means

Produkttyp

Measure: MEASURE_1

Produkttyp	Mean	Std. Error	95% Confidence Interval	
			Lower Bound	Upper Bound
1	2,641	,064	2,513	2,768
2	2,615	,062	2,491	2,740

```
GLM Item2.Diamant.12.12.12 Item2.Diamant.24.01.13 Item2.Guarani.12.12.12 Item2.Guarani.24.01
  /WSFACTOR=Produkttyp 2 Polynomial Messzeitpunkt 2 Polynomial
  /METHOD=SSTYPE(3)
  /EMMEANS=TABLES(Produkttyp)
  /PRINT=DESCRIPTIVE ETASQ
  /CRITERIA=ALPHA(.05)
  /WSDESIGN=Produkttyp Messzeitpunkt Produkttyp*Messzeitpunkt.
```

General Linear Model

Notes

Output Created		07-NOV-2013 12:20:01
Comments		
Input	Data	C:\Documents and Settings\Dennis Boywitt\My Documents\My Dropbox\Freiberufliche Tätigkeit\Forschungsring\Daten\MDBF_Gruppe3_Items.sav
	Active Dataset	DataSet3
	Filter	<none>
	Weight	<none>
	Split File	<none>
	N of Rows in Working Data File	70
Missing Value Handling	Definition of Missing	User-defined missing values are treated as missing.
	Cases Used	Statistics are based on all cases with valid data for all variables in the model.
Syntax		GLM Item2.Diamant. 12.12.12 Item2.Diamant. 24.01.13 Item2.Guarani. 12.12.12 Item2.Guarani. 24.01.13<br>/WSFACTOR=Produkttyp 2 Polynomial Messzeitpunkt 2 Polynomial<br>/METHOD=SSTYPE(3)<br>/EMMEANS=TABLES (Produkttyp)<br>/PRINT=DESCRIPTIVE ETASQ<br>/CRITERIA=ALPHA(.05)<br>/WSDESIGN=Produkttyp Messzeitpunkt Produkttyp*Messzeitpunkt.
Resources	Processor Time	00:00:00,02
	Elapsed Time	00:00:00,02

```
[DataSet3] C:\Documents and Settings\Dennis Boywitt\My Documents\My Dropbo
x\Freiberufliche Tätigkeit\Forschungsring\Daten\MDBF_Gruppe3_Items.sav
```

Within-Subjects Factors

Measure: MEASURE_1

Produkttyp	Messzeitpunkt	Dependent Variable
1	1	Item2. Diamant. 12.12.12
	2	Item2. Diamant. 24.01.13
2	1	Item2. Guarani. 12.12.12
	2	Item2. Guarani. 24.01.13

Descriptive Statistics

	Mean	Std. Deviation	N
Item2.Diamant.12.12.12	2,68	,539	64
Item2.Diamant.24.01.13	2,64	,809	64
Item2.Guarani.12.12.12	2,68	,598	64
Item2.Guarani.24.01.13	2,62	,804	64

Multivariate Tests[a]

Effect		Value	F	Hypothesis df	Error df
Produkttyp	Pillai's Trace	,003	,200[b]	1,000	63,000
	Wilks' Lambda	,997	,200[b]	1,000	63,000
	Hotelling's Trace	,003	,200[b]	1,000	63,000
	Roy's Largest Root	,003	,200[b]	1,000	63,000
Messzeitpunkt	Pillai's Trace	,004	,224[b]	1,000	63,000
	Wilks' Lambda	,996	,224[b]	1,000	63,000
	Hotelling's Trace	,004	,224[b]	1,000	63,000
	Roy's Largest Root	,004	,224[b]	1,000	63,000
Produkttyp * Messzeitpunkt	Pillai's Trace	,006	,412[b]	1,000	63,000
	Wilks' Lambda	,994	,412[b]	1,000	63,000
	Hotelling's Trace	,007	,412[b]	1,000	63,000
	Roy's Largest Root	,007	,412[b]	1,000	63,000

Multivariate Tests[a]

Effect		Sig.	Partial Eta Squared
Produkttyp	Pillai's Trace	,656	,003
	Wilks' Lambda	,656	,003
	Hotelling's Trace	,656	,003
	Roy's Largest Root	,656	,003
Messzeitpunkt	Pillai's Trace	,637	,004
	Wilks' Lambda	,637	,004
	Hotelling's Trace	,637	,004
	Roy's Largest Root	,637	,004
Produkttyp * Messzeitpunkt	Pillai's Trace	,523	,006
	Wilks' Lambda	,523	,006
	Hotelling's Trace	,523	,006
	Roy's Largest Root	,523	,006

a. Design: Intercept
Within Subjects Design: Produkttyp + Messzeitpunkt + Produkttyp * Messzeitpunkt

b. Exact statistic

Mauchly's Test of Sphericity[a]

Measure: MEASURE_1

Within Subjects Effect	Mauchly's W	Approx. Chi-Square	df	Sig.	Epsilon[b]
					Greenhouse-Geisser
Produkttyp	1,000	,000	0	.	1,000
Messzeitpunkt	1,000	,000	0	.	1,000
Produkttyp * Messzeitpunkt	1,000	,000	0	.	1,000

Mauchly's Test of Sphericity[a]

Measure: MEASURE_1

Within Subjects Effect	Epsilon[b]	
	Huynh-Feldt	Lower-bound
Produkttyp	1,000	1,000
Messzeitpunkt	1,000	1,000
Produkttyp * Messzeitpunkt	1,000	1,000

Tests the null hypothesis that the error covariance matrix of the orthonormalized transformed dependent variables is proportional to an identity matrix.

a. Design: Intercept
Within Subjects Design: Produkttyp + Messzeitpunkt + Produkttyp * Messzeitpunkt

b. May be used to adjust the degrees of freedom for the averaged tests of significance. Corrected tests are displayed in the Tests of Within-Subjects Effects table.

Tests of Within-Subjects Effects

Measure: MEASURE_1

Source		Type III Sum of Squares	df	Mean Square
Produkttyp	Sphericity Assumed	,009	1	,009
	Greenhouse-Geisser	,009	1,000	,009
	Huynh-Feldt	,009	1,000	,009
	Lower-bound	,009	1,000	,009
Error(Produkttyp)	Sphericity Assumed	2,772	63	,044
	Greenhouse-Geisser	2,772	63,000	,044
	Huynh-Feldt	2,772	63,000	,044
	Lower-bound	2,772	63,000	,044
Messzeitpunkt	Sphericity Assumed	,141	1	,141
	Greenhouse-Geisser	,141	1,000	,141
	Huynh-Feldt	,141	1,000	,141
	Lower-bound	,141	1,000	,141
Error(Messzeitpunkt)	Sphericity Assumed	39,516	63	,627
	Greenhouse-Geisser	39,516	63,000	,627
	Huynh-Feldt	39,516	63,000	,627
	Lower-bound	39,516	63,000	,627
Produkttyp * Messzeitpunkt	Sphericity Assumed	,016	1	,016
	Greenhouse-Geisser	,016	1,000	,016
	Huynh-Feldt	,016	1,000	,016
	Lower-bound	,016	1,000	,016
Error (Produkttyp*Messzeitpunkt)	Sphericity Assumed	2,391	63	,038
	Greenhouse-Geisser	2,391	63,000	,038
	Huynh-Feldt	2,391	63,000	,038
	Lower-bound	2,391	63,000	,038

Tests of Within-Subjects Effects

Measure: MEASURE_1

Source		F	Sig.	Partial Eta Squared
Produkttyp	Sphericity Assumed	,200	,656	,003
	Greenhouse-Geisser	,200	,656	,003
	Huynh-Feldt	,200	,656	,003
	Lower-bound	,200	,656	,003
Error(Produkttyp)	Sphericity Assumed			
	Greenhouse-Geisser			
	Huynh-Feldt			
	Lower-bound			
Messzeitpunkt	Sphericity Assumed	,224	,637	,004
	Greenhouse-Geisser	,224	,637	,004
	Huynh-Feldt	,224	,637	,004
	Lower-bound	,224	,637	,004
Error(Messzeitpunkt)	Sphericity Assumed			
	Greenhouse-Geisser			
	Huynh-Feldt			
	Lower-bound			
Produkttyp * Messzeitpunkt	Sphericity Assumed	,412	,523	,006
	Greenhouse-Geisser	,412	,523	,006
	Huynh-Feldt	,412	,523	,006
	Lower-bound	,412	,523	,006
Error (Produkttyp*Messzeitpunkt)	Sphericity Assumed			
	Greenhouse-Geisser			
	Huynh-Feldt			
	Lower-bound			

Tests of Within-Subjects Contrasts

Measure: MEASURE_1

Source	Produkttyp	Messzeitpunkt	Type III Sum of Squares	df	Mean Square
Produkttyp	Linear		,009	1	,009
Error(Produkttyp)	Linear		2,772	63	,044
Messzeitpunkt		Linear	,141	1	,141
Error(Messzeitpunkt)		Linear	39,516	63	,627
Produkttyp * Messzeitpunkt	Linear	Linear	,016	1	,016
Error (Produkttyp*Messzeitpunkt)	Linear	Linear	2,391	63	,038

Tests of Within-Subjects Contrasts

Measure: MEASURE_1

Source	Produkttyp	Messzeitpunkt	F	Sig.	Partial Eta Squared
Produkttyp	Linear		,200	,656	,003
Error(Produkttyp)	Linear				
Messzeitpunkt		Linear	,224	,637	,004
Error(Messzeitpunkt)		Linear			
Produkttyp * Messzeitpunkt	Linear	Linear	,412	,523	,006
Error (Produkttyp*Messzeitpunkt)	Linear	Linear			

Tests of Between-Subjects Effects

Measure: MEASURE_1

Transformed Variable: Average

Source	Type III Sum of Squares	df	Mean Square	F	Sig.	Partial Eta Squared
Intercept	1803,595	1	1803,595	1453,274	,000	,958
Error	78,187	63	1,241			

Estimated Marginal Means

Produkttyp

Measure: MEASURE_1

Produkttyp	Mean	Std. Error	95% Confidence Interval	
			Lower Bound	Upper Bound
1	2,660	,070	2,520	2,801
2	2,648	,071	2,506	2,791

```
GLM Item3.Diamant.12.12.12 Item3.Diamant.24.01.13 Item3.Guarani.12.12.12 Item3.Guarani.24.01
  /WSFACTOR=Produkttyp 2 Polynomial Messzeitpunkt 2 Polynomial
  /METHOD=SSTYPE(3)
  /EMMEANS=TABLES(Produkttyp)
  /PRINT=DESCRIPTIVE ETASQ
  /CRITERIA=ALPHA(.05)
  /WSDESIGN=Produkttyp Messzeitpunkt Produkttyp*Messzeitpunkt.
```

General Linear Model

Notes

Output Created		07-NOV-2013 12:20:43
Comments		
Input	Data	C:\Documents and Settings\Dennis Boywitt\My Documents\My Dropbox\Freiberufliche Tätigkeit\Forschungsring\Daten\MDBF_Gruppe3_Items.sav
	Active Dataset	DataSet3
	Filter	<none>
	Weight	<none>
	Split File	<none>
	N of Rows in Working Data File	70
Missing Value Handling	Definition of Missing	User-defined missing values are treated as missing.
	Cases Used	Statistics are based on all cases with valid data for all variables in the model.
Syntax		GLM Item3.Diamant.12.12.12 Item3.Diamant.24.01.13 Item3.Guarani.12.12.12 Item3.Guarani.24.01.13<br>/WSFACTOR=Produkttyp 2 Polynomial Messzeitpunkt 2 Polynomial<br>/METHOD=SSTYPE(3)<br>/EMMEANS=TABLES(Produkttyp)<br>/PRINT=DESCRIPTIVE ETASQ<br>/CRITERIA=ALPHA(.05)<br>/WSDESIGN=Produkttyp Messzeitpunkt Produkttyp*Messzeitpunkt.
Resources	Processor Time	00:00:00,03
	Elapsed Time	00:00:00,03

```
[DataSet3] C:\Documents and Settings\Dennis Boywitt\My Documents\My Dropbo
x\Freiberufliche Tätigkeit\Forschungsring\Daten\MDBF_Gruppe3_Items.sav
```

Within-Subjects Factors

Measure: MEASURE_1

Produkttyp	Messzeitpunkt	Dependent Variable
1	1	Item3. Diamant. 12.12.12
	2	Item3. Diamant. 24.01.13
2	1	Item3. Guarani. 12.12.12
	2	Item3. Guarani. 24.01.13

Descriptive Statistics

	Mean	Std. Deviation	N
Item3.Diamant.12.12.12	2,79	,451	60
Item3.Diamant.24.01.13	2,79	,522	60
Item3.Guarani.12.12.12	2,75	,461	60
Item3.Guarani.24.01.13	2,81	,484	60

Multivariate Tests[a]

Effect		Value	F	Hypothesis df	Error df
Produkttyp	Pillai's Trace	,001	,058[b]	1,000	59,000
	Wilks' Lambda	,999	,058[b]	1,000	59,000
	Hotelling's Trace	,001	,058[b]	1,000	59,000
	Roy's Largest Root	,001	,058[b]	1,000	59,000
Messzeitpunkt	Pillai's Trace	,004	,254[b]	1,000	59,000
	Wilks' Lambda	,996	,254[b]	1,000	59,000
	Hotelling's Trace	,004	,254[b]	1,000	59,000
	Roy's Largest Root	,004	,254[b]	1,000	59,000
Produkttyp * Messzeitpunkt	Pillai's Trace	,019	1,169[b]	1,000	59,000
	Wilks' Lambda	,981	1,169[b]	1,000	59,000
	Hotelling's Trace	,020	1,169[b]	1,000	59,000
	Roy's Largest Root	,020	1,169[b]	1,000	59,000

Multivariate Tests[a]

Effect		Sig.	Partial Eta Squared
Produkttyp	Pillai's Trace	,811	,001
	Wilks' Lambda	,811	,001
	Hotelling's Trace	,811	,001
	Roy's Largest Root	,811	,001
Messzeitpunkt	Pillai's Trace	,616	,004
	Wilks' Lambda	,616	,004
	Hotelling's Trace	,616	,004
	Roy's Largest Root	,616	,004
Produkttyp * Messzeitpunkt	Pillai's Trace	,284	,019
	Wilks' Lambda	,284	,019
	Hotelling's Trace	,284	,019
	Roy's Largest Root	,284	,019

a. Design: Intercept
Within Subjects Design: Produkttyp + Messzeitpunkt + Produkttyp * Messzeitpunkt

b. Exact statistic

Mauchly's Test of Sphericity[a]

Measure: MEASURE_1

Within Subjects Effect	Mauchly's W	Approx. Chi-Square	df	Sig.	Epsilon[b]
					Greenhouse-Geisser
Produkttyp	1,000	,000	0	.	1,000
Messzeitpunkt	1,000	,000	0	.	1,000
Produkttyp * Messzeitpunkt	1,000	,000	0	.	1,000

Mauchly's Test of Sphericity[a]

Measure: MEASURE_1

Within Subjects Effect	Epsilon[b]	
	Huynh-Feldt	Lower-bound
Produkttyp	1,000	1,000
Messzeitpunkt	1,000	1,000
Produkttyp * Messzeitpunkt	1,000	1,000

Tests the null hypothesis that the error covariance matrix of the orthonormalized transformed dependent variables is proportional to an identity matrix.

a. Design: Intercept
Within Subjects Design: Produkttyp + Messzeitpunkt + Produkttyp * Messzeitpunkt

b. May be used to adjust the degrees of freedom for the averaged tests of significance. Corrected tests are displayed in the Tests of Within-Subjects Effects table.

Tests of Within-Subjects Effects

Measure: MEASURE_1

Source		Type III Sum of Squares	df	Mean Square
Produkttyp	Sphericity Assumed	,002	1	,002
	Greenhouse-Geisser	,002	1,000	,002
	Huynh-Feldt	,002	1,000	,002
	Lower-bound	,002	1,000	,002
Error(Produkttyp)	Sphericity Assumed	2,388	59	,040
	Greenhouse-Geisser	2,388	59,000	,040
	Huynh-Feldt	2,388	59,000	,040
	Lower-bound	2,388	59,000	,040
Messzeitpunkt	Sphericity Assumed	,044	1	,044
	Greenhouse-Geisser	,044	1,000	,044
	Huynh-Feldt	,044	1,000	,044
	Lower-bound	,044	1,000	,044
Error(Messzeitpunkt)	Sphericity Assumed	10,222	59	,173
	Greenhouse-Geisser	10,222	59,000	,173
	Huynh-Feldt	10,222	59,000	,173
	Lower-bound	10,222	59,000	,173
Produkttyp * Messzeitpunkt	Sphericity Assumed	,059	1	,059
	Greenhouse-Geisser	,059	1,000	,059
	Huynh-Feldt	,059	1,000	,059
	Lower-bound	,059	1,000	,059
Error (Produkttyp*Messzeitpunkt)	Sphericity Assumed	2,957	59	,050
	Greenhouse-Geisser	2,957	59,000	,050
	Huynh-Feldt	2,957	59,000	,050
	Lower-bound	2,957	59,000	,050

Tests of Within-Subjects Effects

Measure: MEASURE_1

Source		F	Sig.	Partial Eta Squared
Produkttyp	Sphericity Assumed	,058	,811	,001
	Greenhouse-Geisser	,058	,811	,001
	Huynh-Feldt	,058	,811	,001
	Lower-bound	,058	,811	,001
Error(Produkttyp)	Sphericity Assumed			
	Greenhouse-Geisser			
	Huynh-Feldt			
	Lower-bound			
Messzeitpunkt	Sphericity Assumed	,254	,616	,004
	Greenhouse-Geisser	,254	,616	,004
	Huynh-Feldt	,254	,616	,004
	Lower-bound	,254	,616	,004
Error(Messzeitpunkt)	Sphericity Assumed			
	Greenhouse-Geisser			
	Huynh-Feldt			
	Lower-bound			
Produkttyp * Messzeitpunkt	Sphericity Assumed	1,169	,284	,019
	Greenhouse-Geisser	1,169	,284	,019
	Huynh-Feldt	1,169	,284	,019
	Lower-bound	1,169	,284	,019
Error (Produkttyp*Messzeitpunkt)	Sphericity Assumed			
	Greenhouse-Geisser			
	Huynh-Feldt			
	Lower-bound			

Tests of Within-Subjects Contrasts

Measure: MEASURE_1

Source	Produkttyp	Messzeitpunkt	Type III Sum of Squares	df	Mean Square
Produkttyp	Linear		,002	1	,002
Error(Produkttyp)	Linear		2,388	59	,040
Messzeitpunkt		Linear	,044	1	,044
Error(Messzeitpunkt)		Linear	10,222	59	,173
Produkttyp * Messzeitpunkt	Linear	Linear	,059	1	,059
Error (Produkttyp*Messzeitpunkt)	Linear	Linear	2,957	59	,050

Tests of Within-Subjects Contrasts

Measure: MEASURE_1

Source	Produkttyp	Messzeitpunkt	F	Sig.	Partial Eta Squared
Produkttyp	Linear		,058	,811	,001
Error(Produkttyp)	Linear				
Messzeitpunkt		Linear	,254	,616	,004
Error(Messzeitpunkt)		Linear			
Produkttyp * Messzeitpunkt	Linear	Linear	1,169	,284	,019
Error (Produkttyp*Messzeitpunkt)	Linear	Linear			

Tests of Between-Subjects Effects

Measure: MEASURE_1

Transformed Variable: Average

Source	Type III Sum of Squares	df	Mean Square	F	Sig.	Partial Eta Squared
Intercept	1863,444	1	1863,444	2822,920	,000	,980
Error	38,947	59	,660			

Estimated Marginal Means

Produkttyp

Measure: MEASURE_1

Produkttyp	Mean	Std. Error	95% Confidence Interval	
			Lower Bound	Upper Bound
1	2,790	,056	2,677	2,902
2	2,783	,052	2,679	2,887
