# Supplementary material for: Development and Application of a Test for Food-Induced Emotions
Source: PLoS One. 2016 Nov 18;11(11):e0165991. doi: 10.1371/journal.pone.0165991 (PMC5115674; doi:10.1371/journal.pone.0165991)
Supplement: S5 File — (PDF) [file pone.0165991.s008.pdf]

```
GLM Skala1_AGlas.05.12.12 Skala1_AGlas.16.01.13 Skala1_APlastik.05.12.12 Skala1_APlastik.16.
  /WSFACTOR=Produkt 2 Polynomial Messzeitpunkt 2 Polynomial
  /METHOD=SSTYPE(3)
  /EMMEANS=TABLES(Produkt)
  /PRINT=DESCRIPTIVE ETASQ
  /CRITERIA=ALPHA(.05)
  /WSDESIGN=Produkt Messzeitpunkt Produkt*Messzeitpunkt.
```

General Linear Model

Notes

Output Created		24-OCT-2013 17:54:41
Comments		
Input	Data	C:\Documents and Settings\Dennis Boywitt\My Documents\My Dropbox\Freiberufliche Tätigkeit\Forschungsring\Arbeitsordner Daten\Befindlichkeiten_Gruppe2_restructured.sav
	Active Dataset	DataSet2
	Filter	<none>
	Weight	<none>
	Split File	<none>
	N of Rows in Working Data File	62
Missing Value Handling	Definition of Missing	User-defined missing values are treated as missing.
	Cases Used	Statistics are based on all cases with valid data for all variables in the model.
Syntax		GLM Skala1_AGlas.05.12.12 Skala1_AGlas.16.01.13 Skala1_APlastik.05.12.12 Skala1_APlastik.16.01.13 /WSFACTOR=Produkt 2 Polynomial Messzeitpunkt 2 Polynomial /METHOD=SSTYPE(3) /EMMEANS=TABLES(Produkt) /PRINT=DESCRIPTIVE ETASQ /CRITERIA=ALPHA(.05) /WSDESIGN=Produkt Messzeitpunkt Produkt*Messzeitpunkt.

Notes

Resources	Processor Time	00:00:00,03
	Elapsed Time	00:00:00,06

```
[DataSet2] C:\Documents and Settings\Dennis Boywitt\My Documents\My Dropbo
x\Freiberufliche Tätigkeit\Forschungsring\Arbeitsordner Daten\Befindlichke
iten_Gruppe2_restructured.sav
```

Within-Subjects Factors

Measure: MEASURE_1

Produkt	Messzeitpunkt	Dependent Variable
1	1	Skala1_AGlas.05.12.12
	2	Skala1_AGlas.16.01.13
2	1	Skala1_APlastik.05.12.12
	2	Skala1_APlastik.16.01.13

Descriptive Statistics

	Mean	Std. Deviation	N
Skala1_AGlas.05.12.12	2,3441	,79438	59
Skala1_AGlas.16.01.13	2,3729	,86977	59
Skala1_APlastik.05.12.12	2,1364	,63659	59
Skala1_APlastik.16.01.13	2,1356	,67409	59

Multivariate Tests[a]

Effect		Value	F	Hypothesis df	Error df
Produkt	Pillai's Trace	,106	6,900[b]	1,000	58,000
	Wilks' Lambda	,894	6,900[b]	1,000	58,000
	Hotelling's Trace	,119	6,900[b]	1,000	58,000
	Roy's Largest Root	,119	6,900[b]	1,000	58,000
Messzeitpunkt	Pillai's Trace	,000	,028[b]	1,000	58,000
	Wilks' Lambda	1,000	,028[b]	1,000	58,000
	Hotelling's Trace	,000	,028[b]	1,000	58,000
	Roy's Largest Root	,000	,028[b]	1,000	58,000
Produkt * Messzeitpunkt	Pillai's Trace	,001	,037[b]	1,000	58,000
	Wilks' Lambda	,999	,037[b]	1,000	58,000
	Hotelling's Trace	,001	,037[b]	1,000	58,000
	Roy's Largest Root	,001	,037[b]	1,000	58,000

Multivariate Tests[a]

Effect		Sig.	Partial Eta Squared
Produkt	Pillai's Trace	,011	,106
	Wilks' Lambda	,011	,106
	Hotelling's Trace	,011	,106
	Roy's Largest Root	,011	,106
Messzeitpunkt	Pillai's Trace	,867	,000
	Wilks' Lambda	,867	,000
	Hotelling's Trace	,867	,000
	Roy's Largest Root	,867	,000
Produkt * Messzeitpunkt	Pillai's Trace	,849	,001
	Wilks' Lambda	,849	,001
	Hotelling's Trace	,849	,001
	Roy's Largest Root	,849	,001

a. Design: Intercept
Within Subjects Design: Produkt + Messzeitpunkt + Produkt * Messzeitpunkt

b. Exact statistic

Mauchly's Test of Sphericity[a]

Measure: MEASURE_1

Within Subjects Effect	Mauchly's W	Approx. Chi-Square	df	Sig.	Epsilon[b]
					Greenhouse-Geisser
Produkt	1,000	,000	0	.	1,000
Messzeitpunkt	1,000	,000	0	.	1,000
Produkt * Messzeitpunkt	1,000	,000	0	.	1,000

Mauchly's Test of Sphericity[a]

Measure: MEASURE_1

Within Subjects Effect	Epsilon[b]	
	Huynh-Feldt	Lower-bound
Produkt	1,000	1,000
Messzeitpunkt	1,000	1,000
Produkt * Messzeitpunkt	1,000	1,000

Tests the null hypothesis that the error covariance matrix of the orthonormalized transformed dependent variables is proportional to an identity matrix.

a. Design: Intercept
Within Subjects Design: Produkt + Messzeitpunkt + Produkt * Messzeitpunkt

b. May be used to adjust the degrees of freedom for the averaged tests of significance. Corrected tests are displayed in the Tests of Within-Subjects Effects table.

Tests of Within-Subjects Effects

Measure: MEASURE_1

Source		Type III Sum of Squares	df	Mean Square
Produkt	Sphericity Assumed	2,920	1	2,920
	Greenhouse-Geisser	2,920	1,000	2,920
	Huynh-Feldt	2,920	1,000	2,920
	Lower-bound	2,920	1,000	2,920
Error(Produkt)	Sphericity Assumed	24,542	58	,423
	Greenhouse-Geisser	24,542	58,000	,423
	Huynh-Feldt	24,542	58,000	,423
	Lower-bound	24,542	58,000	,423
Messzeitpunkt	Sphericity Assumed	,012	1	,012
	Greenhouse-Geisser	,012	1,000	,012
	Huynh-Feldt	,012	1,000	,012
	Lower-bound	,012	1,000	,012
Error(Messzeitpunkt)	Sphericity Assumed	23,610	58	,407
	Greenhouse-Geisser	23,610	58,000	,407
	Huynh-Feldt	23,610	58,000	,407
	Lower-bound	23,610	58,000	,407
Produkt * Messzeitpunkt	Sphericity Assumed	,013	1	,013
	Greenhouse-Geisser	,013	1,000	,013
	Huynh-Feldt	,013	1,000	,013
	Lower-bound	,013	1,000	,013
Error (Produkt*Messzeitpunkt)	Sphericity Assumed	20,539	58	,354
	Greenhouse-Geisser	20,539	58,000	,354
	Huynh-Feldt	20,539	58,000	,354
	Lower-bound	20,539	58,000	,354

Tests of Within-Subjects Effects

Measure: MEASURE_1

Source		F	Sig.	Partial Eta Squared
Produkt	Sphericity Assumed	6,900	,011	,106
	Greenhouse-Geisser	6,900	,011	,106
	Huynh-Feldt	6,900	,011	,106
	Lower-bound	6,900	,011	,106
Error(Produkt)	Sphericity Assumed			
	Greenhouse-Geisser			
	Huynh-Feldt			
	Lower-bound			
Messzeitpunkt	Sphericity Assumed	,028	,867	,000
	Greenhouse-Geisser	,028	,867	,000
	Huynh-Feldt	,028	,867	,000
	Lower-bound	,028	,867	,000
Error(Messzeitpunkt)	Sphericity Assumed			
	Greenhouse-Geisser			
	Huynh-Feldt			
	Lower-bound			
Produkt * Messzeitpunkt	Sphericity Assumed	,037	,849	,001
	Greenhouse-Geisser	,037	,849	,001
	Huynh-Feldt	,037	,849	,001
	Lower-bound	,037	,849	,001
Error (Produkt*Messzeitpunkt)	Sphericity Assumed			
	Greenhouse-Geisser			
	Huynh-Feldt			
	Lower-bound			

Tests of Within-Subjects Contrasts

Measure: MEASURE_1

Source	Produkt	Messzeitpunkt	Type III Sum of Squares	df	Mean Square
Produkt	Linear		2,920	1	2,920
Error(Produkt)	Linear		24,542	58	,423
Messzeitpunkt		Linear	,012	1	,012
Error(Messzeitpunkt)		Linear	23,610	58	,407
Produkt * Messzeitpunkt	Linear	Linear	,013	1	,013
Error (Produkt*Messzeitpunkt)	Linear	Linear	20,539	58	,354

Tests of Within-Subjects Contrasts

Measure: MEASURE_1

Source	Produkt	Messzeitpunkt	F	Sig.	Partial Eta Squared
Produkt	Linear		6,900	,011	,106
Error(Produkt)	Linear				
Messzeitpunkt		Linear	,028	,867	,000
Error(Messzeitpunkt)		Linear			
Produkt * Messzeitpunkt	Linear	Linear	,037	,849	,001
Error (Produkt*Messzeitpunkt)	Linear	Linear			

Tests of Between-Subjects Effects

Measure: MEASURE_1

Transformed Variable: Average

Source	Type III Sum of Squares	df	Mean Square	F	Sig.	Partial Eta Squared
Intercept	1191,827	1	1191,827	1121,354	,000	,951
Error	61,645	58	1,063			

Estimated Marginal Means

Produkt

Measure: MEASURE_1

Produkt	Mean	Std. Error	95% Confidence Interval	
			Lower Bound	Upper Bound
1	2,358	,088	2,182	2,535
2	2,136	,070	1,997	2,275

```
GLM Skala2_AGlas.05.12.12 Skala2_AGlas.16.01.13 Skala2_APlastik.05.12.12 Skala2_APlastik.16.
  /WSFACTOR=Produkt 2 Polynomial Messzeitpunkt 2 Polynomial
  /METHOD=SSTYPE(3)
  /EMMEANS=TABLES(Produkt)
  /PRINT=DESCRIPTIVE ETASQ
  /CRITERIA=ALPHA(.05)
  /WSDESIGN=Produkt Messzeitpunkt Produkt*Messzeitpunkt.
```

General Linear Model

Notes

Output Created		24-OCT-2013 18:01:02
Comments		
Input	Data	C:\Documents and Settings\Dennis Boywitt\My Documents\My Dropbox\Freiberufliche Tätigkeit\Forschungsring\Arbeitsordner Daten\Befindlichkeiten_Gruppe2_restructured.sav
	Active Dataset	DataSet2
	Filter	<none>
	Weight	<none>
	Split File	<none>
	N of Rows in Working Data File	62
Missing Value Handling	Definition of Missing	User-defined missing values are treated as missing.
	Cases Used	Statistics are based on all cases with valid data for all variables in the model.
Syntax		GLM Skala2_AGlas.05.12.12 Skala2_AGlas.16.01.13 Skala2_APlastik.05.12.12 Skala2_APlastik.16.01.13 /WSFACTOR=Produkt 2 Polynomial Messzeitpunkt 2 Polynomial /METHOD=SSTYPE(3) /EMMEANS=TABLES(Produkt) /PRINT=DESCRIPTIVE ETASQ /CRITERIA=ALPHA(.05) /WSDESIGN=Produkt Messzeitpunkt Produkt*Messzeitpunkt.
Resources	Processor Time	00:00:00,02
	Elapsed Time	00:00:00,02

```
[DataSet2] C:\Documents and Settings\Dennis Boywitt\My Documents\My Dropbox\Freiberufliche Tätigkeit\Forschungsring\Arbeitsordner Daten\Befindlichkeiten_Gruppe2_restructured.sav
```

Within-Subjects Factors

Measure: MEASURE_1

Produkt	Messzeitpunkt	Dependent Variable
1	1	Skala2_AGlas .05.12.12
	2	Skala2_AGlas .16.01.13
2	1	Skala2_APlas tik.05.12.12
	2	Skala2_APlas tik.16.01.13

Descriptive Statistics

	Mean	Std. Deviation	N
Skala2_AGlas.05.12.12	2,1017	,67810	59
Skala2_AGlas.16.01.13	2,2068	,77100	59
Skala2_APlastik.05.12.12	1,9695	,69462	59
Skala2_APlastik.16.01.13	2,0508	,66602	59

Multivariate Tests[a]

Effect		Value	F	Hypothesis df	Error df
Produkt	Pillai's Trace	,059	3,664[b]	1,000	58,000
	Wilks' Lambda	,941	3,664[b]	1,000	58,000
	Hotelling's Trace	,063	3,664[b]	1,000	58,000
	Roy's Largest Root	,063	3,664[b]	1,000	58,000
Messzeitpunkt	Pillai's Trace	,037	2,257[b]	1,000	58,000
	Wilks' Lambda	,963	2,257[b]	1,000	58,000
	Hotelling's Trace	,039	2,257[b]	1,000	58,000
	Roy's Largest Root	,039	2,257[b]	1,000	58,000
Produkt * Messzeitpunkt	Pillai's Trace	,001	,039[b]	1,000	58,000
	Wilks' Lambda	,999	,039[b]	1,000	58,000
	Hotelling's Trace	,001	,039[b]	1,000	58,000
	Roy's Largest Root	,001	,039[b]	1,000	58,000

Multivariate Tests[a]

Effect		Sig.	Partial Eta Squared
Produkt	Pillai's Trace	,061	,059
	Wilks' Lambda	,061	,059
	Hotelling's Trace	,061	,059
	Roy's Largest Root	,061	,059
Messzeitpunkt	Pillai's Trace	,138	,037
	Wilks' Lambda	,138	,037
	Hotelling's Trace	,138	,037
	Roy's Largest Root	,138	,037
Produkt * Messzeitpunkt	Pillai's Trace	,843	,001
	Wilks' Lambda	,843	,001
	Hotelling's Trace	,843	,001
	Roy's Largest Root	,843	,001

a. Design: Intercept
Within Subjects Design: Produkt + Messzeitpunkt + Produkt * Messzeitpunkt

b. Exact statistic

Mauchly's Test of Sphericity[a]

Measure: MEASURE_1

Within Subjects Effect	Mauchly's W	Approx. Chi-Square	df	Sig.	Epsilon[b]
					Greenhouse-Geisser
Produkt	1,000	,000	0	.	1,000
Messzeitpunkt	1,000	,000	0	.	1,000
Produkt * Messzeitpunkt	1,000	,000	0	.	1,000

Mauchly's Test of Sphericity[a]

Measure: MEASURE_1

Within Subjects Effect	Epsilon[b]	
	Huynh-Feldt	Lower-bound
Produkt	1,000	1,000
Messzeitpunkt	1,000	1,000
Produkt * Messzeitpunkt	1,000	1,000

Tests the null hypothesis that the error covariance matrix of the orthonormalized transformed dependent variables is proportional to an identity matrix.

a. Design: Intercept
Within Subjects Design: Produkt + Messzeitpunkt + Produkt * Messzeitpunkt

b. May be used to adjust the degrees of freedom for the averaged tests of significance. Corrected tests are displayed in the Tests of Within-Subjects Effects table.

Tests of Within-Subjects Effects

Measure: MEASURE_1

Source		Type III Sum of Squares	df	Mean Square
Produkt	Sphericity Assumed	1,225	1	1,225
	Greenhouse-Geisser	1,225	1,000	1,225
	Huynh-Feldt	1,225	1,000	1,225
	Lower-bound	1,225	1,000	1,225
Error(Produkt)	Sphericity Assumed	19,385	58	,334
	Greenhouse-Geisser	19,385	58,000	,334
	Huynh-Feldt	19,385	58,000	,334
	Lower-bound	19,385	58,000	,334
Messzeitpunkt	Sphericity Assumed	,513	1	,513
	Greenhouse-Geisser	,513	1,000	,513
	Huynh-Feldt	,513	1,000	,513
	Lower-bound	,513	1,000	,513
Error(Messzeitpunkt)	Sphericity Assumed	13,177	58	,227
	Greenhouse-Geisser	13,177	58,000	,227
	Huynh-Feldt	13,177	58,000	,227
	Lower-bound	13,177	58,000	,227
Produkt * Messzeitpunkt	Sphericity Assumed	,008	1	,008
	Greenhouse-Geisser	,008	1,000	,008
	Huynh-Feldt	,008	1,000	,008
	Lower-bound	,008	1,000	,008
Error (Produkt*Messzeitpunkt)	Sphericity Assumed	12,242	58	,211
	Greenhouse-Geisser	12,242	58,000	,211
	Huynh-Feldt	12,242	58,000	,211
	Lower-bound	12,242	58,000	,211

Tests of Within-Subjects Effects

Measure: MEASURE_1

Source		F	Sig.	Partial Eta Squared
Produkt	Sphericity Assumed	3,664	,061	,059
	Greenhouse-Geisser	3,664	,061	,059
	Huynh-Feldt	3,664	,061	,059
	Lower-bound	3,664	,061	,059
Error(Produkt)	Sphericity Assumed			
	Greenhouse-Geisser			
	Huynh-Feldt			
	Lower-bound			
Messzeitpunkt	Sphericity Assumed	2,257	,138	,037
	Greenhouse-Geisser	2,257	,138	,037
	Huynh-Feldt	2,257	,138	,037
	Lower-bound	2,257	,138	,037
Error(Messzeitpunkt)	Sphericity Assumed			
	Greenhouse-Geisser			
	Huynh-Feldt			
	Lower-bound			
Produkt * Messzeitpunkt	Sphericity Assumed	,039	,843	,001
	Greenhouse-Geisser	,039	,843	,001
	Huynh-Feldt	,039	,843	,001
	Lower-bound	,039	,843	,001
Error (Produkt*Messzeitpunkt)	Sphericity Assumed			
	Greenhouse-Geisser			
	Huynh-Feldt			
	Lower-bound			

Tests of Within-Subjects Contrasts

Measure: MEASURE_1

Source	Produkt	Messzeitpunkt	Type III Sum of Squares	df	Mean Square
Produkt	Linear		1,225	1	1,225
Error(Produkt)	Linear		19,385	58	,334
Messzeitpunkt		Linear	,513	1	,513
Error(Messzeitpunkt)		Linear	13,177	58	,227
Produkt * Messzeitpunkt	Linear	Linear	,008	1	,008
Error (Produkt*Messzeitpunkt)	Linear	Linear	12,242	58	,211

Tests of Within-Subjects Contrasts

Measure: MEASURE_1

Source	Produkt	Messzeitpunkt	F	Sig.	Partial Eta Squared
Produkt	Linear		3,664	,061	,059
Error(Produkt)	Linear				
Messzeitpunkt		Linear	2,257	,138	,037
Error(Messzeitpunkt)		Linear			
Produkt * Messzeitpunkt	Linear	Linear	,039	,843	,001
Error (Produkt*Messzeitpunkt)	Linear	Linear			

Tests of Between-Subjects Effects

Measure: MEASURE_1

Transformed Variable: Average

Source	Type III Sum of Squares	df	Mean Square	F	Sig.	Partial Eta Squared
Intercept	1023,195	1	1023,195	847,121	,000	,936
Error	70,055	58	1,208			

Estimated Marginal Means

Produkt

Measure: MEASURE_1

Produkt	Mean	Std. Error	95% Confidence Interval	
			Lower Bound	Upper Bound
1	2,154	,083	1,987	2,321
2	2,010	,078	1,854	2,167

```
 GLM Befindlichkeit1.AdelholzerGlas.05.12.12 Befindlichkeit1.AdelholzerGlas.16.01.13
Befindlichkeit1.AdelholzerPlastik.05.12.12 Befindlichkeit1.AdelholzerPlastik.16.01.13
 /WSFACTOR=Produkt 2 Polynomial Messzeitpunkt 2 Polynomial
  /METHOD=SSTYPE(3)
  /EMMEANS=TABLES(Produkt)
  /PRINT=DESCRIPTIVE ETASQ
  /CRITERIA=ALPHA(.05)
  /WSDESIGN=Produkt Messzeitpunkt Produkt*Messzeitpunkt.
```

General Linear Model

Notes

Output Created		24-OCT-2013 18:05:03
Comments		
Input	Data	C:\Documents and Settings\Dennis Boywitt\My Documents\My Dropbox\Freiberufliche Tätigkeit\Forschungsring\Arbeitsordner Daten\Befindlichkeiten_Gruppe2_restructured.sav
	Active Dataset	DataSet2
	Filter	<none>
	Weight	<none>
	Split File	<none>
	N of Rows in Working Data File	62
Missing Value Handling	Definition of Missing	User-defined missing values are treated as missing.
	Cases Used	Statistics are based on all cases with valid data for all variables in the model.
Syntax		GLM Befindlichkeit1. AdelholzerGlas.05.12.12 Befindlichkeit1. AdelholzerGlas.16.01.13 Befindlichkeit1. AdelholzerPlastik.05.12.12 Befindlichkeit1. AdelholzerPlastik.16.01.13 /WSFACTOR=Produkt 2 Polynomial Messzeitpunkt 2 Polynomial /METHOD=SSTYPE(3) /EMMEANS=TABLES (Produkt) /PRINT=DESCRIPTIVE ETASQ /CRITERIA=ALPHA(.05) /WSDESIGN=Produkt Messzeitpunkt Produkt*Messzeitpunkt.
Resources	Processor Time	00:00:00,00
	Elapsed Time	00:00:00,03

```
[DataSet2] C:\Documents and Settings\Dennis Boywitt\My Documents\My Dropbo
x\Freiberufliche Tätigkeit\Forschungsring\Arbeitsordner Daten\Befindlichke
iten_Gruppe2_restructured.sav
```

Within-Subjects Factors

Measure: MEASURE_1

Produkt	Messzeitpunkt	Dependent Variable
1	1	Befindlichkeit1.AdelholzerGlas.05.12.12
	2	Befindlichkeit1.AdelholzerGlas.16.01.13
2	1	Befindlichkeit1.AdelholzerPlastik.05.12.12
	2	Befindlichkeit1.AdelholzerPlastik.16.01.13

Descriptive Statistics

	Mean	Std. Deviation	N
Befindlichkeit1. AdelholzerGlas.05.12.12: Ich empfinde meinen Leib 1	3,05	1,195	59
Befindlichkeit1. AdelholzerGlas.16.01.13: Ich empfinde meinen Leib 1	2,78	1,247	59
Befindlichkeit1. AdelholzerPlastik. 05.12.12: Ich empfinde meinen Leib 1	3,05	1,195	59
Befindlichkeit1. AdelholzerPlastik. 16.01.13: Ich empfinde meinen Leib 1	3,14	1,238	59

Multivariate Tests[a]

Effect		Value	F	Hypothesis df	Error df
Produkt	Pillai's Trace	,036	2,151[b]	1,000	58,000
	Wilks' Lambda	,964	2,151[b]	1,000	58,000
	Hotelling's Trace	,037	2,151[b]	1,000	58,000
	Roy's Largest Root	,037	2,151[b]	1,000	58,000
Messzeitpunkt	Pillai's Trace	,007	,403[b]	1,000	58,000
	Wilks' Lambda	,993	,403[b]	1,000	58,000
	Hotelling's Trace	,007	,403[b]	1,000	58,000
	Roy's Largest Root	,007	,403[b]	1,000	58,000
Produkt * Messzeitpunkt	Pillai's Trace	,033	1,993[b]	1,000	58,000
	Wilks' Lambda	,967	1,993[b]	1,000	58,000
	Hotelling's Trace	,034	1,993[b]	1,000	58,000
	Roy's Largest Root	,034	1,993[b]	1,000	58,000

Multivariate Tests[a]

Effect		Sig.	Partial Eta Squared
Produkt	Pillai's Trace	,148	,036
	Wilks' Lambda	,148	,036
	Hotelling's Trace	,148	,036
	Roy's Largest Root	,148	,036
Messzeitpunkt	Pillai's Trace	,528	,007
	Wilks' Lambda	,528	,007
	Hotelling's Trace	,528	,007
	Roy's Largest Root	,528	,007
Produkt * Messzeitpunkt	Pillai's Trace	,163	,033
	Wilks' Lambda	,163	,033
	Hotelling's Trace	,163	,033
	Roy's Largest Root	,163	,033

a. Design: Intercept
Within Subjects Design: Produkt + Messzeitpunkt + Produkt * Messzeitpunkt

b. Exact statistic

Mauchly's Test of Sphericity[a]

Measure: MEASURE_1

Within Subjects Effect	Mauchly's W	Approx. Chi-Square	df	Sig.	Epsilon[b] Greenhouse-Geisser
Produkt	1,000	,000	0	.	1,000
Messzeitpunkt	1,000	,000	0	.	1,000
Produkt * Messzeitpunkt	1,000	,000	0	.	1,000

Mauchly's Test of Sphericity[a]

Measure: MEASURE_1

Within Subjects Effect	Epsilon[b]	
	Huynh-Feldt	Lower-bound
Produkt	1,000	1,000
Messzeitpunkt	1,000	1,000
Produkt * Messzeitpunkt	1,000	1,000

Tests the null hypothesis that the error covariance matrix of the orthonormalized transformed dependent variables is proportional to an identity matrix.

a. Design: Intercept
Within Subjects Design: Produkt + Messzeitpunkt + Produkt * Messzeitpunkt

b. May be used to adjust the degrees of freedom for the averaged tests of significance. Corrected tests are displayed in the Tests of Within-Subjects Effects table.

Tests of Within-Subjects Effects

Measure: MEASURE_1

Source		Type III Sum of Squares	df	Mean Square
Produkt	Sphericity Assumed	1,869	1	1,869
	Greenhouse-Geisser	1,869	1,000	1,869
	Huynh-Feldt	1,869	1,000	1,869
	Lower-bound	1,869	1,000	1,869
Error(Produkt)	Sphericity Assumed	50,381	58	,869
	Greenhouse-Geisser	50,381	58,000	,869
	Huynh-Feldt	50,381	58,000	,869
	Lower-bound	50,381	58,000	,869
Messzeitpunkt	Sphericity Assumed	,513	1	,513
	Greenhouse-Geisser	,513	1,000	,513
	Huynh-Feldt	,513	1,000	,513
	Lower-bound	,513	1,000	,513
Error(Messzeitpunkt)	Sphericity Assumed	73,737	58	1,271
	Greenhouse-Geisser	73,737	58,000	1,271
	Huynh-Feldt	73,737	58,000	1,271
	Lower-bound	73,737	58,000	1,271
Produkt * Messzeitpunkt	Sphericity Assumed	1,869	1	1,869
	Greenhouse-Geisser	1,869	1,000	1,869
	Huynh-Feldt	1,869	1,000	1,869
	Lower-bound	1,869	1,000	1,869
Error (Produkt*Messzeitpunkt)	Sphericity Assumed	54,381	58	,938
	Greenhouse-Geisser	54,381	58,000	,938
	Huynh-Feldt	54,381	58,000	,938
	Lower-bound	54,381	58,000	,938

Tests of Within-Subjects Effects

Measure: MEASURE_1

Source		F	Sig.	Partial Eta Squared
Produkt	Sphericity Assumed	2,151	,148	,036
	Greenhouse-Geisser	2,151	,148	,036
	Huynh-Feldt	2,151	,148	,036
	Lower-bound	2,151	,148	,036
Error(Produkt)	Sphericity Assumed			
	Greenhouse-Geisser			
	Huynh-Feldt			
	Lower-bound			
Messzeitpunkt	Sphericity Assumed	,403	,528	,007
	Greenhouse-Geisser	,403	,528	,007
	Huynh-Feldt	,403	,528	,007
	Lower-bound	,403	,528	,007
Error(Messzeitpunkt)	Sphericity Assumed			
	Greenhouse-Geisser			
	Huynh-Feldt			
	Lower-bound			
Produkt * Messzeitpunkt	Sphericity Assumed	1,993	,163	,033
	Greenhouse-Geisser	1,993	,163	,033
	Huynh-Feldt	1,993	,163	,033
	Lower-bound	1,993	,163	,033
Error (Produkt*Messzeitpunkt)	Sphericity Assumed			
	Greenhouse-Geisser			
	Huynh-Feldt			
	Lower-bound			

Tests of Within-Subjects Contrasts

Measure: MEASURE_1

Source	Produkt	Messzeitpunkt	Type III Sum of Squares	df	Mean Square
Produkt	Linear		1,869	1	1,869
Error(Produkt)	Linear		50,381	58	,869
Messzeitpunkt		Linear	,513	1	,513
Error(Messzeitpunkt)		Linear	73,737	58	1,271
Produkt * Messzeitpunkt	Linear	Linear	1,869	1	1,869
Error (Produkt*Messzeitpunkt)	Linear	Linear	54,381	58	,938

Tests of Within-Subjects Contrasts

Measure: MEASURE_1

Source	Produkt	Messzeitpunkt	F	Sig.	Partial Eta Squared
Produkt	Linear		2,151	,148	,036
Error(Produkt)	Linear				
Messzeitpunkt		Linear	,403	,528	,007
Error(Messzeitpunkt)		Linear			
Produkt * Messzeitpunkt	Linear	Linear	1,993	,163	,033
Error (Produkt*Messzeitpunkt)	Linear	Linear			

Tests of Between-Subjects Effects

Measure: MEASURE_1

Transformed Variable: Average

Source	Type III Sum of Squares	df	Mean Square	F	Sig.	Partial Eta Squared
Intercept	2130,004	1	2130,004	743,118	,000	,928
Error	166,246	58	2,866			

Estimated Marginal Means

Produkt

Measure: MEASURE_1

Produkt	Mean	Std. Error	95% Confidence Interval	
			Lower Bound	Upper Bound
1	2,915	,122	2,671	3,160
2	3,093	,129	2,834	3,352

```
 GLM Befindlichkeit12.AdelholzerGlas.05.12.12 Befindlichkeit12.AdelholzerGlas.16.01.13
Befindlichkeit12.AdelholzerPlastik.05.12.12 Befindlichkeit12.AdelholzerPlastik.16.01.13
 /WSFACTOR=Produkt 2 Polynomial Messzeitpunkt 2 Polynomial
  /METHOD=SSTYPE(3)
  /EMMEANS=TABLES(Produkt)
  /PRINT=DESCRIPTIVE ETASQ
  /CRITERIA=ALPHA(.05)
  /WSDESIGN=Produkt Messzeitpunkt Produkt*Messzeitpunkt.
```

General Linear Model

Notes

Output Created		24-OCT-2013 18:06:51
Comments		
Input	Data	C:\Documents and Settings\Dennis Boywitt\My Documents\My Dropbox\Freiberufliche Tätigkeit\Forschungsring\Arbeitsordner Daten\Befindlichkeiten_Gruppe2_restructured.sav
	Active Dataset	DataSet2
	Filter	<none>
	Weight	<none>
	Split File	<none>
	N of Rows in Working Data File	62
Missing Value Handling	Definition of Missing	User-defined missing values are treated as missing.
	Cases Used	Statistics are based on all cases with valid data for all variables in the model.
Syntax		GLM Befindlichkeit12.AdelholzerGlas.05.12.12 Befindlichkeit12.AdelholzerGlas.16.01.13 Befindlichkeit12.AdelholzerPlastik.05.12.12 Befindlichkeit12.AdelholzerPlastik.16.01.13 /WSFACTOR=Produkt 2 Polynomial Messzeitpunkt 2 Polynomial /METHOD=SSTYPE(3) /EMMEANS=TABLES(Produkt) /PRINT=DESCRIPTIVE ETASQ /CRITERIA=ALPHA(.05) /WSDESIGN=Produkt Messzeitpunkt Produkt*Messzeitpunkt.
Resources	Processor Time	00:00:00,03
	Elapsed Time	00:00:00,03

```
[DataSet2] C:\Documents and Settings\Dennis Boywitt\My Documents\My Dropbo
x\Freiberufliche Tätigkeit\Forschungsring\Arbeitsordner Daten\Befindlichke
iten_Gruppe2_restructured.sav
```

Within-Subjects Factors

Measure: MEASURE_1

Produkt	Messzeitpunkt	Dependent Variable
1	1	Befindlichkeit12. AdelholzerGlas.05.12.12
	2	Befindlichkeit12. AdelholzerGlas.16.01.13
2	1	Befindlichkeit12. AdelholzerPlastik.05.12.12
	2	Befindlichkeit12. AdelholzerPlastik.16.01.13

Descriptive Statistics

	Mean	Std. Deviation	N
Befindlichkeit12. AdelholzerGlas.05.12.12: Ich empfinde die Wirkung	3,03	,928	59
Befindlichkeit12. AdelholzerGlas.16.01.13: Ich empfinde die Wirkung	2,63	1,173	59
Befindlichkeit12. AdelholzerPlastik. 05.12.12: Ich empfinde die Wirkung	2,47	1,040	59
Befindlichkeit12. AdelholzerPlastik. 16.01.13: Ich empfinde die Wirkung	2,64	1,047	59

Multivariate Tests[a]

Effect		Value	F	Hypothesis df	Error df
Produkt	Pillai's Trace	,073	4,562[b]	1,000	58,000
	Wilks' Lambda	,927	4,562[b]	1,000	58,000
	Hotelling's Trace	,079	4,562[b]	1,000	58,000
	Roy's Largest Root	,079	4,562[b]	1,000	58,000
Messzeitpunkt	Pillai's Trace	,011	,628[b]	1,000	58,000
	Wilks' Lambda	,989	,628[b]	1,000	58,000
	Hotelling's Trace	,011	,628[b]	1,000	58,000
	Roy's Largest Root	,011	,628[b]	1,000	58,000
Produkt * Messzeitpunkt	Pillai's Trace	,156	10,680[b]	1,000	58,000
	Wilks' Lambda	,844	10,680[b]	1,000	58,000
	Hotelling's Trace	,184	10,680[b]	1,000	58,000
	Roy's Largest Root	,184	10,680[b]	1,000	58,000

Multivariate Tests[a]

Effect		Sig.	Partial Eta Squared
Produkt	Pillai's Trace	,037	,073
	Wilks' Lambda	,037	,073
	Hotelling's Trace	,037	,073
	Roy's Largest Root	,037	,073
Messzeitpunkt	Pillai's Trace	,431	,011
	Wilks' Lambda	,431	,011
	Hotelling's Trace	,431	,011
	Roy's Largest Root	,431	,011
Produkt * Messzeitpunkt	Pillai's Trace	,002	,156
	Wilks' Lambda	,002	,156
	Hotelling's Trace	,002	,156
	Roy's Largest Root	,002	,156

a. Design: Intercept
Within Subjects Design: Produkt + Messzeitpunkt + Produkt * Messzeitpunkt

b. Exact statistic

Mauchly's Test of Sphericity[a]

Measure: MEASURE_1

Within Subjects Effect	Mauchly's W	Approx. Chi-Square	df	Sig.	Epsilon[b]
					Greenhouse-Geisser
Produkt	1,000	,000	0	.	1,000
Messzeitpunkt	1,000	,000	0	.	1,000
Produkt * Messzeitpunkt	1,000	,000	0	.	1,000

Mauchly's Test of Sphericity[a]

Measure: MEASURE_1

Within Subjects Effect	Epsilon[b]	
	Huynh-Feldt	Lower-bound
Produkt	1,000	1,000
Messzeitpunkt	1,000	1,000
Produkt * Messzeitpunkt	1,000	1,000

Tests the null hypothesis that the error covariance matrix of the orthonormalized transformed dependent variables is proportional to an identity matrix.

a. Design: Intercept
Within Subjects Design: Produkt + Messzeitpunkt + Produkt * Messzeitpunkt

b. May be used to adjust the degrees of freedom for the averaged tests of significance. Corrected tests are displayed in the Tests of Within-Subjects Effects table.

Tests of Within-Subjects Effects

Measure: MEASURE_1

Source		Type III Sum of Squares	df	Mean Square
Produkt	Sphericity Assumed	4,339	1	4,339
	Greenhouse-Geisser	4,339	1,000	4,339
	Huynh-Feldt	4,339	1,000	4,339
	Lower-bound	4,339	1,000	4,339
Error(Produkt)	Sphericity Assumed	55,161	58	,951
	Greenhouse-Geisser	55,161	58,000	,951
	Huynh-Feldt	55,161	58,000	,951
	Lower-bound	55,161	58,000	,951
Messzeitpunkt	Sphericity Assumed	,831	1	,831
	Greenhouse-Geisser	,831	1,000	,831
	Huynh-Feldt	,831	1,000	,831
	Lower-bound	,831	1,000	,831
Error(Messzeitpunkt)	Sphericity Assumed	76,669	58	1,322
	Greenhouse-Geisser	76,669	58,000	1,322
	Huynh-Feldt	76,669	58,000	1,322
	Lower-bound	76,669	58,000	1,322
Produkt * Messzeitpunkt	Sphericity Assumed	4,898	1	4,898
	Greenhouse-Geisser	4,898	1,000	4,898
	Huynh-Feldt	4,898	1,000	4,898
	Lower-bound	4,898	1,000	4,898
Error (Produkt*Messzeitpunkt)	Sphericity Assumed	26,602	58	,459
	Greenhouse-Geisser	26,602	58,000	,459
	Huynh-Feldt	26,602	58,000	,459
	Lower-bound	26,602	58,000	,459

Tests of Within-Subjects Effects

Measure: MEASURE_1

Source		F	Sig.	Partial Eta Squared
Produkt	Sphericity Assumed	4,562	,037	,073
	Greenhouse-Geisser	4,562	,037	,073
	Huynh-Feldt	4,562	,037	,073
	Lower-bound	4,562	,037	,073
Error(Produkt)	Sphericity Assumed			
	Greenhouse-Geisser			
	Huynh-Feldt			
	Lower-bound			
Messzeitpunkt	Sphericity Assumed	,628	,431	,011
	Greenhouse-Geisser	,628	,431	,011
	Huynh-Feldt	,628	,431	,011
	Lower-bound	,628	,431	,011
Error(Messzeitpunkt)	Sphericity Assumed			
	Greenhouse-Geisser			
	Huynh-Feldt			
	Lower-bound			
Produkt * Messzeitpunkt	Sphericity Assumed	10,680	,002	,156
	Greenhouse-Geisser	10,680	,002	,156
	Huynh-Feldt	10,680	,002	,156
	Lower-bound	10,680	,002	,156
Error (Produkt*Messzeitpunkt)	Sphericity Assumed			
	Greenhouse-Geisser			
	Huynh-Feldt			
	Lower-bound			

Tests of Within-Subjects Contrasts

Measure: MEASURE_1

Source	Produkt	Messzeitpunkt	Type III Sum of Squares	df	Mean Square
Produkt	Linear		4,339	1	4,339
Error(Produkt)	Linear		55,161	58	,951
Messzeitpunkt		Linear	,831	1	,831
Error(Messzeitpunkt)		Linear	76,669	58	1,322
Produkt * Messzeitpunkt	Linear	Linear	4,898	1	4,898
Error (Produkt*Messzeitpunkt)	Linear	Linear	26,602	58	,459

Tests of Within-Subjects Contrasts

Measure: MEASURE_1

Source	Produkt	Messzeitpunkt	F	Sig.	Partial Eta Squared
Produkt	Linear		4,562	,037	,073
Error(Produkt)	Linear				
Messzeitpunkt		Linear	,628	,431	,011
Error(Messzeitpunkt)		Linear			
Produkt * Messzeitpunkt	Linear	Linear	10,680	,002	,156
Error (Produkt*Messzeitpunkt)	Linear	Linear			

Tests of Between-Subjects Effects

Measure: MEASURE_1

Transformed Variable: Average

Source	Type III Sum of Squares	df	Mean Square	F	Sig.	Partial Eta Squared
Intercept	1713,966	1	1713,966	1019,236	,000	,946
Error	97,534	58	1,682			

Estimated Marginal Means

Produkt

Measure: MEASURE_1

Produkt	Mean	Std. Error	95% Confidence Interval	
			Lower Bound	Upper Bound
1	2,831	,106	2,619	3,042
2	2,559	,105	2,348	2,770
