# Supplementary material for: Development and Application of a Test for Food-Induced Emotions
Source: PLoS One. 2016 Nov 18;11(11):e0165991. doi: 10.1371/journal.pone.0165991 (PMC5115674; doi:10.1371/journal.pone.0165991)
Supplement: S6 File — (PDF) [file pone.0165991.s009.pdf]

```
GLM Skala1_Söbbeke.05.12.12 Skala1_Söbbeke.16.01.13 Skala1_Landliebe.05.12.12 Skala1_Landlie
  /WSFACTOR=Produkt 2 Polynomial Messzeitpunkt 2 Polynomial
  /METHOD=SSTYPE(3)
  /EMMEANS=TABLES(Produkt)
  /PRINT=DESCRIPTIVE ETASQ
  /CRITERIA=ALPHA(.05)
  /WSDESIGN=Produkt Messzeitpunkt Produkt*Messzeitpunkt.
```

General Linear Model

Notes

Output Created		24-OCT-2013 18:13:44
Comments		
Input	Data	C:\Documents and Settings\Dennis Boywitt\My Documents\My Dropbox\Freiberufliche Tätigkeit\Forschungsring\Arbeitsordner Daten\Befindlichkeiten_Gruppe2_restructured.sav
	Active Dataset	DataSet2
	Filter	<none>
	Weight	<none>
	Split File	<none>
	N of Rows in Working Data File	62
Missing Value Handling	Definition of Missing	User-defined missing values are treated as missing.
	Cases Used	Statistics are based on all cases with valid data for all variables in the model.

Notes

Syntax		GLM Skala1_Söbbeke.05.12.12 Skala1_Söbbeke.16.01.13 Skala1_Landliebe.05.12.12 Skala1_Landliebe.16.01.13 /WSFACTOR=Produkt 2 Polynomial Messzeitpunkt 2 Polynomial /METHOD=SSTYPE(3) /EMMEANS=TABLES(Produkt) /PRINT=DESCRIPTIVE ETASQ /CRITERIA=ALPHA(.05) /WSDESIGN=Produkt Messzeitpunkt Produkt*Messzeitpunkt.
Resources	Processor Time	00:00:00,02
	Elapsed Time	00:00:00,02

```
[DataSet2] C:\Documents and Settings\Dennis Boywitt\My Documents\My Dropbo
x\Freiberufliche Tätigkeit\Forschungsring\Arbeitsordner Daten\Befindlichke
iten_Gruppe2_restructured.sav
```

Within-Subjects Factors

Measure: MEASURE_1

Produkt	Messzeitpunkt	Dependent Variable
1	1	Skala1_Söbbeke.05.12.12
	2	Skala1_Söbbeke.16.01.13
2	1	Skala1_Landliebe.05.12.12
	2	Skala1_Landliebe.16.01.13

Descriptive Statistics

	Mean	Std. Deviation	N
Skala1_Söbbeke.05.12.12	2,2746	,85573	59
Skala1_Söbbeke.16.01.13	2,3424	,97560	59
Skala1_Landliebe.05.12.12	2,3788	,90588	59
Skala1_Landliebe.16.01.13	2,3831	,89542	59

Multivariate Tests[a]

Effect		Value	F	Hypothesis df	Error df
Produkt	Pillai's Trace	,008	,483[b]	1,000	58,000
	Wilks' Lambda	,992	,483[b]	1,000	58,000
	Hotelling's Trace	,008	,483[b]	1,000	58,000
	Roy's Largest Root	,008	,483[b]	1,000	58,000
Messzeitpunkt	Pillai's Trace	,003	,151[b]	1,000	58,000
	Wilks' Lambda	,997	,151[b]	1,000	58,000
	Hotelling's Trace	,003	,151[b]	1,000	58,000
	Roy's Largest Root	,003	,151[b]	1,000	58,000
Produkt * Messzeitpunkt	Pillai's Trace	,002	,143[b]	1,000	58,000
	Wilks' Lambda	,998	,143[b]	1,000	58,000
	Hotelling's Trace	,002	,143[b]	1,000	58,000
	Roy's Largest Root	,002	,143[b]	1,000	58,000

Multivariate Tests[a]

Effect		Sig.	Partial Eta Squared
Produkt	Pillai's Trace	,490	,008
	Wilks' Lambda	,490	,008
	Hotelling's Trace	,490	,008
	Roy's Largest Root	,490	,008
Messzeitpunkt	Pillai's Trace	,699	,003
	Wilks' Lambda	,699	,003
	Hotelling's Trace	,699	,003
	Roy's Largest Root	,699	,003
Produkt * Messzeitpunkt	Pillai's Trace	,706	,002
	Wilks' Lambda	,706	,002
	Hotelling's Trace	,706	,002
	Roy's Largest Root	,706	,002

a. Design: Intercept
Within Subjects Design: Produkt + Messzeitpunkt + Produkt * Messzeitpunkt

b. Exact statistic

Mauchly's Test of Sphericity[a]

Measure: MEASURE_1

Within Subjects Effect	Mauchly's W	Approx. Chi-Square	df	Sig.	Epsilon[b] Greenhouse-Geisser
Produkt	1,000	,000	0	.	1,000
Messzeitpunkt	1,000	,000	0	.	1,000
Produkt * Messzeitpunkt	1,000	,000	0	.	1,000

Mauchly's Test of Sphericity[a]

Measure: MEASURE_1

Within Subjects Effect	Epsilon[b]	
	Huynh-Feldt	Lower-bound
Produkt	1,000	1,000
Messzeitpunkt	1,000	1,000
Produkt * Messzeitpunkt	1,000	1,000

Tests the null hypothesis that the error covariance matrix of the orthonormalized transformed dependent variables is proportional to an identity matrix.

a. Design: Intercept
Within Subjects Design: Produkt + Messzeitpunkt + Produkt * Messzeitpunkt

b. May be used to adjust the degrees of freedom for the averaged tests of significance. Corrected tests are displayed in the Tests of Within-Subjects Effects table.

Tests of Within-Subjects Effects

Measure: MEASURE_1

Source		Type III Sum of Squares	df	Mean Square
Produkt	Sphericity Assumed	,310	1	,310
	Greenhouse-Geisser	,310	1,000	,310
	Huynh-Feldt	,310	1,000	,310
	Lower-bound	,310	1,000	,310
Error(Produkt)	Sphericity Assumed	37,206	58	,641
	Greenhouse-Geisser	37,206	58,000	,641
	Huynh-Feldt	37,206	58,000	,641
	Lower-bound	37,206	58,000	,641
Messzeitpunkt	Sphericity Assumed	,077	1	,077
	Greenhouse-Geisser	,077	1,000	,077
	Huynh-Feldt	,077	1,000	,077
	Lower-bound	,077	1,000	,077
Error(Messzeitpunkt)	Sphericity Assumed	29,489	58	,508
	Greenhouse-Geisser	29,489	58,000	,508
	Huynh-Feldt	29,489	58,000	,508
	Lower-bound	29,489	58,000	,508
Produkt * Messzeitpunkt	Sphericity Assumed	,060	1	,060
	Greenhouse-Geisser	,060	1,000	,060
	Huynh-Feldt	,060	1,000	,060
	Lower-bound	,060	1,000	,060
Error (Produkt*Messzeitpunkt)	Sphericity Assumed	24,086	58	,415
	Greenhouse-Geisser	24,086	58,000	,415
	Huynh-Feldt	24,086	58,000	,415
	Lower-bound	24,086	58,000	,415

Tests of Within-Subjects Effects

Measure: MEASURE_1

Source		F	Sig.	Partial Eta Squared
Produkt	Sphericity Assumed	,483	,490	,008
	Greenhouse-Geisser	,483	,490	,008
	Huynh-Feldt	,483	,490	,008
	Lower-bound	,483	,490	,008
Error(Produkt)	Sphericity Assumed			
	Greenhouse-Geisser			
	Huynh-Feldt			
	Lower-bound			
Messzeitpunkt	Sphericity Assumed	,151	,699	,003
	Greenhouse-Geisser	,151	,699	,003
	Huynh-Feldt	,151	,699	,003
	Lower-bound	,151	,699	,003
Error(Messzeitpunkt)	Sphericity Assumed			
	Greenhouse-Geisser			
	Huynh-Feldt			
	Lower-bound			
Produkt * Messzeitpunkt	Sphericity Assumed	,143	,706	,002
	Greenhouse-Geisser	,143	,706	,002
	Huynh-Feldt	,143	,706	,002
	Lower-bound	,143	,706	,002
Error (Produkt*Messzeitpunkt)	Sphericity Assumed			
	Greenhouse-Geisser			
	Huynh-Feldt			
	Lower-bound			

Tests of Within-Subjects Contrasts

Measure: MEASURE_1

Source	Produkt	Messzeitpunkt	Type III Sum of Squares	df	Mean Square
Produkt	Linear		,310	1	,310
Error(Produkt)	Linear		37,206	58	,641
Messzeitpunkt		Linear	,077	1	,077
Error(Messzeitpunkt)		Linear	29,489	58	,508
Produkt * Messzeitpunkt	Linear	Linear	,060	1	,060
Error (Produkt*Messzeitpunkt)	Linear	Linear	24,086	58	,415

Tests of Within-Subjects Contrasts

Measure: MEASURE_1

Source	Produkt	Messzeitpunkt	F	Sig.	Partial Eta Squared
Produkt	Linear		,483	,490	,008
Error(Produkt)	Linear				
Messzeitpunkt		Linear	,151	,699	,003
Error(Messzeitpunkt)		Linear			
Produkt * Messzeitpunkt	Linear	Linear	,143	,706	,002
Error (Produkt*Messzeitpunkt)	Linear	Linear			

Tests of Between-Subjects Effects

Measure: MEASURE_1

Transformed Variable: Average

Source	Type III Sum of Squares	df	Mean Square	F	Sig.	Partial Eta Squared
Intercept	1297,442	1	1297,442	745,110	,000	,928
Error	100,994	58	1,741			

Estimated Marginal Means

Produkt

Measure: MEASURE_1

Produkt	Mean	Std. Error	95% Confidence Interval	
			Lower Bound	Upper Bound
1	2,308	,101	2,106	2,511
2	2,381	,100	2,181	2,581

```
GLM Skala2_Söbbeke.05.12.12 Skala2_Söbbeke.16.01.13 Skala2_Landliebe.05.12.12 Skala2_Landlie
  /WSFACTOR=Produkt 2 Polynomial Messzeitpunkt 2 Polynomial
  /METHOD=SSTYPE(3)
  /EMMEANS=TABLES(Produkt)
  /PRINT=DESCRIPTIVE ETASQ
  /CRITERIA=ALPHA(.05)
  /WSDESIGN=Produkt Messzeitpunkt Produkt*Messzeitpunkt.
```

General Linear Model

Notes

Output Created		24-OCT-2013 18:14:32
Comments		
Input	Data	C:\Documents and Settings\Dennis Boywitt\My Documents\My Dropbox\Freiberufliche Tätigkeit\Forschungsring\Arbeitsordner Daten\Befindlichkeiten_Gruppe2_restructured.sav
	Active Dataset	DataSet2
	Filter	<none>
	Weight	<none>
	Split File	<none>
	N of Rows in Working Data File	62
Missing Value Handling	Definition of Missing	User-defined missing values are treated as missing.
	Cases Used	Statistics are based on all cases with valid data for all variables in the model.
Syntax		GLM Skala2_Söbbeke. 05.12.12 Skala2_Söbbeke.16.01.13 Skala2_Landliebe. 05.12.12 Skala2_Landliebe. 16.01.13<br>/WSFACTOR=Produkt 2 Polynomial Messzeitpunkt 2 Polynomial<br>/METHOD=SSTYPE(3)<br>/EMMEANS=TABLES (Produkt)<br>/PRINT=DESCRIPTIVE ETASQ<br>/CRITERIA=ALPHA(.05)<br>/WSDESIGN=Produkt Messzeitpunkt Produkt*Messzeitpunkt.
Resources	Processor Time	00:00:00,03
	Elapsed Time	00:00:00,03

```
[DataSet2] C:\Documents and Settings\Dennis Boywitt\My Documents\My Dropbo
x\Freiberufliche Tätigkeit\Forschungsring\Arbeitsordner Daten\Befindlichke
iten_Gruppe2_restructured.sav
```

Within-Subjects Factors

Measure: MEASURE_1

Produkt	Messzeitpunkt	Dependent Variable
1	1	Skala2_Söbbeke.05.12.12
	2	Skala2_Söbbeke.16.01.13
2	1	Skala2_Landliebe.05.12.12
	2	Skala2_Landliebe.16.01.13

Descriptive Statistics

	Mean	Std. Deviation	N
Skala2_Söbbeke.05.12.12	1,9000	,68783	62
Skala2_Söbbeke.16.01.13	2,0097	,66226	62
Skala2_Landliebe.05.12.12	2,1210	,79818	62
Skala2_Landliebe.16.01.13	2,2000	,77163	62

Multivariate Tests[a]

Effect		Value	F	Hypothesis df	Error df
Produkt	Pillai's Trace	,085	5,632[b]	1,000	61,000
	Wilks' Lambda	,915	5,632[b]	1,000	61,000
	Hotelling's Trace	,092	5,632[b]	1,000	61,000
	Roy's Largest Root	,092	5,632[b]	1,000	61,000
Messzeitpunkt	Pillai's Trace	,045	2,885[b]	1,000	61,000
	Wilks' Lambda	,955	2,885[b]	1,000	61,000
	Hotelling's Trace	,047	2,885[b]	1,000	61,000
	Roy's Largest Root	,047	2,885[b]	1,000	61,000
Produkt * Messzeitpunkt	Pillai's Trace	,001	,058[b]	1,000	61,000
	Wilks' Lambda	,999	,058[b]	1,000	61,000
	Hotelling's Trace	,001	,058[b]	1,000	61,000
	Roy's Largest Root	,001	,058[b]	1,000	61,000

Multivariate Tests[a]

Effect		Sig.	Partial Eta Squared
Produkt	Pillai's Trace	,021	,085
	Wilks' Lambda	,021	,085
	Hotelling's Trace	,021	,085
	Roy's Largest Root	,021	,085
Messzeitpunkt	Pillai's Trace	,094	,045
	Wilks' Lambda	,094	,045
	Hotelling's Trace	,094	,045
	Roy's Largest Root	,094	,045
Produkt * Messzeitpunkt	Pillai's Trace	,811	,001
	Wilks' Lambda	,811	,001
	Hotelling's Trace	,811	,001
	Roy's Largest Root	,811	,001

a. Design: Intercept
Within Subjects Design: Produkt + Messzeitpunkt + Produkt * Messzeitpunkt

b. Exact statistic

Mauchly's Test of Sphericity[a]

Measure: MEASURE_1

Within Subjects Effect	Mauchly's W	Approx. Chi-Square	df	Sig.	Epsilon[b] Greenhouse-Geisser
Produkt	1,000	,000	0	.	1,000
Messzeitpunkt	1,000	,000	0	.	1,000
Produkt * Messzeitpunkt	1,000	,000	0	.	1,000

Mauchly's Test of Sphericity[a]

Measure: MEASURE_1

Within Subjects Effect	Epsilon[b]	
	Huynh-Feldt	Lower-bound
Produkt	1,000	1,000
Messzeitpunkt	1,000	1,000
Produkt * Messzeitpunkt	1,000	1,000

Tests the null hypothesis that the error covariance matrix of the orthonormalized transformed dependent variables is proportional to an identity matrix.

a. Design: Intercept
Within Subjects Design: Produkt + Messzeitpunkt + Produkt * Messzeitpunkt

b. May be used to adjust the degrees of freedom for the averaged tests of significance. Corrected tests are displayed in the Tests of Within-Subjects Effects table.

Tests of Within-Subjects Effects

Measure: MEASURE_1

Source		Type III Sum of Squares	df	Mean Square
Produkt	Sphericity Assumed	2,622	1	2,622
	Greenhouse-Geisser	2,622	1,000	2,622
	Huynh-Feldt	2,622	1,000	2,622
	Lower-bound	2,622	1,000	2,622
Error(Produkt)	Sphericity Assumed	28,401	61	,466
	Greenhouse-Geisser	28,401	61,000	,466
	Huynh-Feldt	28,401	61,000	,466
	Lower-bound	28,401	61,000	,466
Messzeitpunkt	Sphericity Assumed	,552	1	,552
	Greenhouse-Geisser	,552	1,000	,552
	Huynh-Feldt	,552	1,000	,552
	Lower-bound	,552	1,000	,552
Error(Messzeitpunkt)	Sphericity Assumed	11,671	61	,191
	Greenhouse-Geisser	11,671	61,000	,191
	Huynh-Feldt	11,671	61,000	,191
	Lower-bound	11,671	61,000	,191
Produkt * Messzeitpunkt	Sphericity Assumed	,015	1	,015
	Greenhouse-Geisser	,015	1,000	,015
	Huynh-Feldt	,015	1,000	,015
	Lower-bound	,015	1,000	,015
Error (Produkt*Messzeitpunkt)	Sphericity Assumed	15,328	61	,251
	Greenhouse-Geisser	15,328	61,000	,251
	Huynh-Feldt	15,328	61,000	,251
	Lower-bound	15,328	61,000	,251

Tests of Within-Subjects Effects

Measure: MEASURE_1

Source		F	Sig.	Partial Eta Squared
Produkt	Sphericity Assumed	5,632	,021	,085
	Greenhouse-Geisser	5,632	,021	,085
	Huynh-Feldt	5,632	,021	,085
	Lower-bound	5,632	,021	,085
Error(Produkt)	Sphericity Assumed			
	Greenhouse-Geisser			
	Huynh-Feldt			
	Lower-bound			
Messzeitpunkt	Sphericity Assumed	2,885	,094	,045
	Greenhouse-Geisser	2,885	,094	,045
	Huynh-Feldt	2,885	,094	,045
	Lower-bound	2,885	,094	,045
Error(Messzeitpunkt)	Sphericity Assumed			
	Greenhouse-Geisser			
	Huynh-Feldt			
	Lower-bound			
Produkt * Messzeitpunkt	Sphericity Assumed	,058	,811	,001
	Greenhouse-Geisser	,058	,811	,001
	Huynh-Feldt	,058	,811	,001
	Lower-bound	,058	,811	,001
Error (Produkt*Messzeitpunkt)	Sphericity Assumed			
	Greenhouse-Geisser			
	Huynh-Feldt			
	Lower-bound			

Tests of Within-Subjects Contrasts

Measure: MEASURE_1

Source	Produkt	Messzeitpunkt	Type III Sum of Squares	df	Mean Square
Produkt	Linear		2,622	1	2,622
Error(Produkt)	Linear		28,401	61	,466
Messzeitpunkt		Linear	,552	1	,552
Error(Messzeitpunkt)		Linear	11,671	61	,191
Produkt * Messzeitpunkt	Linear	Linear	,015	1	,015
Error (Produkt*Messzeitpunkt)	Linear	Linear	15,328	61	,251

Tests of Within-Subjects Contrasts

Measure: MEASURE_1

Source	Produkt	Messzeitpunkt	F	Sig.	Partial Eta Squared
Produkt	Linear		5,632	,021	,085
Error(Produkt)	Linear				
Messzeitpunkt		Linear	2,885	,094	,045
Error(Messzeitpunkt)		Linear			
Produkt * Messzeitpunkt	Linear	Linear	,058	,811	,001
Error (Produkt*Messzeitpunkt)	Linear	Linear			

Tests of Between-Subjects Effects

Measure: MEASURE_1

Transformed Variable: Average

Source	Type III Sum of Squares	df	Mean Square	F	Sig.	Partial Eta Squared
Intercept	1050,025	1	1050,025	849,513	,000	,933
Error	75,398	61	1,236			

Estimated Marginal Means

Produkt

Measure: MEASURE_1

Produkt	Mean	Std. Error	95% Confidence Interval	
			Lower Bound	Upper Bound
1	1,955	,074	1,807	2,103
2	2,160	,091	1,979	2,342

```
GLM Befindlichkeit1.Söbbeke.05.12.12 Befindlichkeit1.Söbbeke.16.01.13
Befindlichkeit1.Landliebe.05.12.12 Befindlichkeit1.Landliebe.16.01.13
 /WSFACTOR=Produkt 2 Polynomial Messzeitpunkt 2 Polynomial
 /METHOD=SSTYPE(3)
 /EMMEANS=TABLES(Produkt)
 /PRINT=DESCRIPTIVE ETASQ
 /CRITERIA=ALPHA(.05)
 /WSDESIGN=Produkt Messzeitpunkt Produkt*Messzeitpunkt.
```

General Linear Model

Notes

Output Created		24-OCT-2013 18:17:07
Comments		
Input	Data	C:\Documents and Settings\Dennis Boywitt\My Documents\My Dropbox\Freiberufliche Tätigkeit\Forschungsring\Arbeitsordner Daten\Befindlichkeiten_Gruppe2_restructured.sav
	Active Dataset	DataSet2
	Filter	<none>
	Weight	<none>
	Split File	<none>
	N of Rows in Working Data File	62
Missing Value Handling	Definition of Missing	User-defined missing values are treated as missing.
	Cases Used	Statistics are based on all cases with valid data for all variables in the model.
Syntax		GLM Befindlichkeit1.Söbbeke.05.12.12 Befindlichkeit1.Söbbeke.16.01.13<br>Befindlichkeit1.Landliebe.05.12.12 Befindlichkeit1.Landliebe.16.01.13<br>/WSFACTOR=Produkt 2 Polynomial Messzeitpunkt 2 Polynomial<br>/METHOD=SSTYPE(3)<br>/EMMEANS=TABLES(Produkt)<br>/PRINT=DESCRIPTIVE ETASQ<br>/CRITERIA=ALPHA(.05)<br>/WSDESIGN=Produkt Messzeitpunkt Produkt*Messzeitpunkt.
Resources	Processor Time	00:00:00,03
	Elapsed Time	00:00:00,03

[DataSet2] C:\Documents and Settings\Dennis Boywitt\My Documents\My Dropbox\Freiberufliche Tätigkeit\Forschungsring\Arbeitsordner Daten\Befindlichkeiten_Gruppe2_restructured.sav

Within-Subjects Factors

Measure: MEASURE_1

Produkt	Messzeitpunkt	Dependent Variable
1	1	Befindlichkeit1.Söbbeke.05.12.12
	2	Befindlichkeit1.Söbbeke.16.01.13
2	1	Befindlichkeit1.Landliebe.05.12.12
	2	Befindlichkeit1.Landliebe.16.01.13

Descriptive Statistics

	Mean	Std. Deviation	N
Befindlichkeit1.Söbbeke.05.12.12: Ich empfinde meinen Leib 1	2,53	1,291	59
Befindlichkeit1.Söbbeke.16.01.13: Ich empfinde meinen Leib 1	2,75	1,347	59
Befindlichkeit1.Landliebe.05.12.12: Ich empfinde meinen Leib 1	3,00	1,402	59
Befindlichkeit1.Landliebe.16.01.13: Ich empfinde meinen Leib 1	2,61	1,218	59

Multivariate Tests[a]

Effect		Value	F	Hypothesis df	Error df
Produkt	Pillai's Trace	,025	1,505[b]	1,000	58,000
	Wilks' Lambda	,975	1,505[b]	1,000	58,000
	Hotelling's Trace	,026	1,505[b]	1,000	58,000
	Roy's Largest Root	,026	1,505[b]	1,000	58,000
Messzeitpunkt	Pillai's Trace	,007	,406[b]	1,000	58,000
	Wilks' Lambda	,993	,406[b]	1,000	58,000
	Hotelling's Trace	,007	,406[b]	1,000	58,000
	Roy's Largest Root	,007	,406[b]	1,000	58,000
Produkt * Messzeitpunkt	Pillai's Trace	,117	7,673[b]	1,000	58,000
	Wilks' Lambda	,883	7,673[b]	1,000	58,000
	Hotelling's Trace	,132	7,673[b]	1,000	58,000
	Roy's Largest Root	,132	7,673[b]	1,000	58,000

Multivariate Tests[a]

Effect		Sig.	Partial Eta Squared
Produkt	Pillai's Trace	,225	,025
	Wilks' Lambda	,225	,025
	Hotelling's Trace	,225	,025
	Roy's Largest Root	,225	,025
Messzeitpunkt	Pillai's Trace	,527	,007
	Wilks' Lambda	,527	,007
	Hotelling's Trace	,527	,007
	Roy's Largest Root	,527	,007
Produkt * Messzeitpunkt	Pillai's Trace	,008	,117
	Wilks' Lambda	,008	,117
	Hotelling's Trace	,008	,117
	Roy's Largest Root	,008	,117

a. Design: Intercept
Within Subjects Design: Produkt + Messzeitpunkt + Produkt * Messzeitpunkt

b. Exact statistic

Mauchly's Test of Sphericity[a]

Measure: MEASURE_1

Within Subjects Effect	Mauchly's W	Approx. Chi-Square	df	Sig.	Epsilon[b]
					Greenhouse-Geisser
Produkt	1,000	,000	0	.	1,000
Messzeitpunkt	1,000	,000	0	.	1,000
Produkt * Messzeitpunkt	1,000	,000	0	.	1,000

Mauchly's Test of Sphericity[a]

Measure: MEASURE_1

Within Subjects Effect	Epsilon[b]	
	Huynh-Feldt	Lower-bound
Produkt	1,000	1,000
Messzeitpunkt	1,000	1,000
Produkt * Messzeitpunkt	1,000	1,000

Tests the null hypothesis that the error covariance matrix of the orthonormalized transformed dependent variables is proportional to an identity matrix.

a. Design: Intercept
Within Subjects Design: Produkt + Messzeitpunkt + Produkt * Messzeitpunkt

b. May be used to adjust the degrees of freedom for the averaged tests of significance. Corrected tests are displayed in the Tests of Within-Subjects Effects table.

Tests of Within-Subjects Effects

Measure: MEASURE_1

Source		Type III Sum of Squares	df	Mean Square
Produkt	Sphericity Assumed	1,695	1	1,695
	Greenhouse-Geisser	1,695	1,000	1,695
	Huynh-Feldt	1,695	1,000	1,695
	Lower-bound	1,695	1,000	1,695
Error(Produkt)	Sphericity Assumed	65,305	58	1,126
	Greenhouse-Geisser	65,305	58,000	1,126
	Huynh-Feldt	65,305	58,000	1,126
	Lower-bound	65,305	58,000	1,126
Messzeitpunkt	Sphericity Assumed	,424	1	,424
	Greenhouse-Geisser	,424	1,000	,424
	Huynh-Feldt	,424	1,000	,424
	Lower-bound	,424	1,000	,424
Error(Messzeitpunkt)	Sphericity Assumed	60,576	58	1,044
	Greenhouse-Geisser	60,576	58,000	1,044
	Huynh-Feldt	60,576	58,000	1,044
	Lower-bound	60,576	58,000	1,044
Produkt * Messzeitpunkt	Sphericity Assumed	5,492	1	5,492
	Greenhouse-Geisser	5,492	1,000	5,492
	Huynh-Feldt	5,492	1,000	5,492
	Lower-bound	5,492	1,000	5,492
Error (Produkt*Messzeitpunkt)	Sphericity Assumed	41,508	58	,716
	Greenhouse-Geisser	41,508	58,000	,716
	Huynh-Feldt	41,508	58,000	,716
	Lower-bound	41,508	58,000	,716

Tests of Within-Subjects Effects

Measure: MEASURE_1

Source		F	Sig.	Partial Eta Squared
Produkt	Sphericity Assumed	1,505	,225	,025
	Greenhouse-Geisser	1,505	,225	,025
	Huynh-Feldt	1,505	,225	,025
	Lower-bound	1,505	,225	,025
Error(Produkt)	Sphericity Assumed			
	Greenhouse-Geisser			
	Huynh-Feldt			
	Lower-bound			
Messzeitpunkt	Sphericity Assumed	,406	,527	,007
	Greenhouse-Geisser	,406	,527	,007
	Huynh-Feldt	,406	,527	,007
	Lower-bound	,406	,527	,007
Error(Messzeitpunkt)	Sphericity Assumed			
	Greenhouse-Geisser			
	Huynh-Feldt			
	Lower-bound			
Produkt * Messzeitpunkt	Sphericity Assumed	7,673	,008	,117
	Greenhouse-Geisser	7,673	,008	,117
	Huynh-Feldt	7,673	,008	,117
	Lower-bound	7,673	,008	,117
Error (Produkt*Messzeitpunkt)	Sphericity Assumed			
	Greenhouse-Geisser			
	Huynh-Feldt			
	Lower-bound			

Tests of Within-Subjects Contrasts

Measure: MEASURE_1

Source	Produkt	Messzeitpunkt	Type III Sum of Squares	df	Mean Square
Produkt	Linear		1,695	1	1,695
Error(Produkt)	Linear		65,305	58	1,126
Messzeitpunkt		Linear	,424	1	,424
Error(Messzeitpunkt)		Linear	60,576	58	1,044
Produkt * Messzeitpunkt	Linear	Linear	5,492	1	5,492
Error (Produkt*Messzeitpunkt)	Linear	Linear	41,508	58	,716

Tests of Within-Subjects Contrasts

Measure: MEASURE_1

Source	Produkt	Messzeitpunkt	F	Sig.	Partial Eta Squared
Produkt	Linear		1,505	,225	,025
Error(Produkt)	Linear				
Messzeitpunkt		Linear	,406	,527	,007
Error(Messzeitpunkt)		Linear			
Produkt * Messzeitpunkt	Linear	Linear	7,673	,008	,117
Error (Produkt*Messzeitpunkt)	Linear	Linear			

Tests of Between-Subjects Effects

Measure: MEASURE_1

Transformed Variable: Average

Source	Type III Sum of Squares	df	Mean Square	F	Sig.	Partial Eta Squared
Intercept	1746,458	1	1746,458	431,882	,000	,882
Error	234,542	58	4,044			

Estimated Marginal Means

Produkt

Measure: MEASURE_1

Produkt	Mean	Std. Error	95% Confidence Interval	
			Lower Bound	Upper Bound
1	2,636	,149	2,336	2,935
2	2,805	,147	2,512	3,098

```
GLM Befindlichkeit12.Söbbeke.05.12.12 Befindlichkeit12.Söbbeke.16.01.13
Befindlichkeit12.Landliebe.05.12.12 Befindlichkeit12.Landliebe.16.01.13
 /WSFACTOR=Produkt 2 Polynomial Messzeitpunkt 2 Polynomial
 /METHOD=SSTYPE(3)
 /EMMEANS=TABLES(Produkt)
 /PRINT=DESCRIPTIVE ETASQ
 /CRITERIA=ALPHA(.05)
 /WSDESIGN=Produkt Messzeitpunkt Produkt*Messzeitpunkt.
```

General Linear Model

Notes

Output Created		24-OCT-2013 18:19:43
Comments		
Input	Data	C:\Documents and Settings\Dennis Boywitt\My Documents\My Dropbox\Freiberufliche Tätigkeit\Forschungsring\Arbeitsordner Daten\Befindlichkeiten_Gruppe2_restructured.sav
	Active Dataset	DataSet2
	Filter	<none>
	Weight	<none>
	Split File	<none>
	N of Rows in Working Data File	62
Missing Value Handling	Definition of Missing	User-defined missing values are treated as missing.
	Cases Used	Statistics are based on all cases with valid data for all variables in the model.
Syntax		GLM Befindlichkeit12.Söbbeke.05.12.12 Befindlichkeit12.Söbbeke.16.01.13 Befindlichkeit12.Landliebe.05.12.12 Befindlichkeit12.Landliebe.16.01.13 /WSFACTOR=Produkt 2 Polynomial Messzeitpunkt 2 Polynomial /METHOD=SSTYPE(3) /EMMEANS=TABLES(Produkt) /PRINT=DESCRIPTIVE ETASQ /CRITERIA=ALPHA(.05) /WSDESIGN=Produkt Messzeitpunkt Produkt*Messzeitpunkt.
Resources	Processor Time	00:00:00,02
	Elapsed Time	00:00:00,02

```
[DataSet2] C:\Documents and Settings\Dennis Boywitt\My Documents\My Dropbo
x\Freiberufliche Tätigkeit\Forschungsring\Arbeitsordner Daten\Befindlichke
iten_Gruppe2_restructured.sav
```

Within-Subjects Factors

Measure: MEASURE_1

Produkt	Messzeitpunkt	Dependent Variable
1	1	Befindlichkeit12.Söbbeke.05.12.12
	2	Befindlichkeit12.Söbbeke.16.01.13
2	1	Befindlichkeit12.Landliebe.05.12.12
	2	Befindlichkeit12.Landliebe.16.01.13

Descriptive Statistics

	Mean	Std. Deviation	N
Befindlichkeit12.Söbbeke.05.12.12: Ich empfinde die Wirkung	2,14	,776	59
Befindlichkeit12.Söbbeke.16.01.13: Ich empfinde die Wirkung	2,37	1,128	59
Befindlichkeit12.Landliebe.05.12.12: Ich empfinde die Wirkung	2,24	1,056	59
Befindlichkeit12.Landliebe.16.01.13: Ich empfinde die Wirkung	2,49	1,006	59

Multivariate Tests[a]

Effect		Value	F	Hypothesis df	Error df
Produkt	Pillai's Trace	,014	,814[b]	1,000	58,000
	Wilks' Lambda	,986	,814[b]	1,000	58,000
	Hotelling's Trace	,014	,814[b]	1,000	58,000
	Roy's Largest Root	,014	,814[b]	1,000	58,000
Messzeitpunkt	Pillai's Trace	,062	3,814[b]	1,000	58,000
	Wilks' Lambda	,938	3,814[b]	1,000	58,000
	Hotelling's Trace	,066	3,814[b]	1,000	58,000
	Roy's Largest Root	,066	3,814[b]	1,000	58,000
Produkt * Messzeitpunkt	Pillai's Trace	,000	,006[b]	1,000	58,000
	Wilks' Lambda	1,000	,006[b]	1,000	58,000
	Hotelling's Trace	,000	,006[b]	1,000	58,000
	Roy's Largest Root	,000	,006[b]	1,000	58,000

Multivariate Tests[a]

Effect		Sig.	Partial Eta Squared
Produkt	Pillai's Trace	,371	,014
	Wilks' Lambda	,371	,014
	Hotelling's Trace	,371	,014
	Roy's Largest Root	,371	,014
Messzeitpunkt	Pillai's Trace	,056	,062
	Wilks' Lambda	,056	,062
	Hotelling's Trace	,056	,062
	Roy's Largest Root	,056	,062
Produkt * Messzeitpunkt	Pillai's Trace	,940	,000
	Wilks' Lambda	,940	,000
	Hotelling's Trace	,940	,000
	Roy's Largest Root	,940	,000

a. Design: Intercept
Within Subjects Design: Produkt + Messzeitpunkt + Produkt * Messzeitpunkt

b. Exact statistic

Mauchly's Test of Sphericity[a]

Measure: MEASURE_1

Within Subjects Effect	Mauchly's W	Approx. Chi-Square	df	Sig.	Epsilon[b]
					Greenhouse-Geisser
Produkt	1,000	,000	0	.	1,000
Messzeitpunkt	1,000	,000	0	.	1,000
Produkt * Messzeitpunkt	1,000	,000	0	.	1,000

Mauchly's Test of Sphericity[a]

Measure: MEASURE_1

Within Subjects Effect	Epsilon[b]	
	Huynh-Feldt	Lower-bound
Produkt	1,000	1,000
Messzeitpunkt	1,000	1,000
Produkt * Messzeitpunkt	1,000	1,000

Tests the null hypothesis that the error covariance matrix of the orthonormalized transformed dependent variables is proportional to an identity matrix.

a. Design: Intercept
Within Subjects Design: Produkt + Messzeitpunkt + Produkt * Messzeitpunkt

b. May be used to adjust the degrees of freedom for the averaged tests of significance. Corrected tests are displayed in the Tests of Within-Subjects Effects table.

Tests of Within-Subjects Effects

Measure: MEASURE_1

Source		Type III Sum of Squares	df	Mean Square
Produkt	Sphericity Assumed	,716	1	,716
	Greenhouse-Geisser	,716	1,000	,716
	Huynh-Feldt	,716	1,000	,716
	Lower-bound	,716	1,000	,716
Error(Produkt)	Sphericity Assumed	51,034	58	,880
	Greenhouse-Geisser	51,034	58,000	,880
	Huynh-Feldt	51,034	58,000	,880
	Lower-bound	51,034	58,000	,880
Messzeitpunkt	Sphericity Assumed	3,564	1	3,564
	Greenhouse-Geisser	3,564	1,000	3,564
	Huynh-Feldt	3,564	1,000	3,564
	Lower-bound	3,564	1,000	3,564
Error(Messzeitpunkt)	Sphericity Assumed	54,186	58	,934
	Greenhouse-Geisser	54,186	58,000	,934
	Huynh-Feldt	54,186	58,000	,934
	Lower-bound	54,186	58,000	,934
Produkt * Messzeitpunkt	Sphericity Assumed	,004	1	,004
	Greenhouse-Geisser	,004	1,000	,004
	Huynh-Feldt	,004	1,000	,004
	Lower-bound	,004	1,000	,004
Error (Produkt*Messzeitpunkt)	Sphericity Assumed	42,746	58	,737
	Greenhouse-Geisser	42,746	58,000	,737
	Huynh-Feldt	42,746	58,000	,737
	Lower-bound	42,746	58,000	,737

Tests of Within-Subjects Effects

Measure: MEASURE_1

Source		F	Sig.	Partial Eta Squared
Produkt	Sphericity Assumed	,814	,371	,014
	Greenhouse-Geisser	,814	,371	,014
	Huynh-Feldt	,814	,371	,014
	Lower-bound	,814	,371	,014
Error(Produkt)	Sphericity Assumed			
	Greenhouse-Geisser			
	Huynh-Feldt			
	Lower-bound			
Messzeitpunkt	Sphericity Assumed	3,814	,056	,062
	Greenhouse-Geisser	3,814	,056	,062
	Huynh-Feldt	3,814	,056	,062
	Lower-bound	3,814	,056	,062
Error(Messzeitpunkt)	Sphericity Assumed			
	Greenhouse-Geisser			
	Huynh-Feldt			
	Lower-bound			
Produkt * Messzeitpunkt	Sphericity Assumed	,006	,940	,000
	Greenhouse-Geisser	,006	,940	,000
	Huynh-Feldt	,006	,940	,000
	Lower-bound	,006	,940	,000
Error (Produkt*Messzeitpunkt)	Sphericity Assumed			
	Greenhouse-Geisser			
	Huynh-Feldt			
	Lower-bound			

Tests of Within-Subjects Contrasts

Measure: MEASURE_1

Source	Produkt	Messzeitpunkt	Type III Sum of Squares	df	Mean Square
Produkt	Linear		,716	1	,716
Error(Produkt)	Linear		51,034	58	,880
Messzeitpunkt		Linear	3,564	1	3,564
Error(Messzeitpunkt)		Linear	54,186	58	,934
Produkt * Messzeitpunkt	Linear	Linear	,004	1	,004
Error (Produkt*Messzeitpunkt)	Linear	Linear	42,746	58	,737

Tests of Within-Subjects Contrasts

Measure: MEASURE_1

Source	Produkt	Messzeitpunkt	F	Sig.	Partial Eta Squared
Produkt	Linear		,814	,371	,014
Error(Produkt)	Linear				
Messzeitpunkt		Linear	3,814	,056	,062
Error(Messzeitpunkt)		Linear			
Produkt * Messzeitpunkt	Linear	Linear	,006	,940	,000
Error (Produkt*Messzeitpunkt)	Linear	Linear			

Tests of Between-Subjects Effects

Measure: MEASURE_1

Transformed Variable: Average

Source	Type III Sum of Squares	df	Mean Square	F	Sig.	Partial Eta Squared
Intercept	1258,581	1	1258,581	867,270	,000	,937
Error	84,169	58	1,451			

Estimated Marginal Means

Produkt

Measure: MEASURE_1

Produkt	Mean	Std. Error	95% Confidence Interval	
			Lower Bound	Upper Bound
1	2,254	,093	2,068	2,441
2	2,364	,105	2,154	2,575
