# Supplementary material for: Development and Application of a Test for Food-Induced Emotions
Source: PLoS One. 2016 Nov 18;11(11):e0165991. doi: 10.1371/journal.pone.0165991 (PMC5115674; doi:10.1371/journal.pone.0165991)
Supplement: S7 File — (PDF) [file pone.0165991.s010.pdf]

```
GLM Skala1_Naturastar.06.12.12 Skala1_Naturastar.17.01.13 Skala1_Goldblume.06.12.12 Skala1_G
  /WSFACTOR=Produkt 2 Polynomial Messzeitpunkt 2 Polynomial
  /METHOD=SSTYPE(3)
  /EMMEANS=TABLES(Produkt)
  /PRINT=DESCRIPTIVE ETASQ
  /CRITERIA=ALPHA(.05)
  /WSDESIGN=Produkt Messzeitpunkt Produkt*Messzeitpunkt.
```

General Linear Model

Notes

Output Created		24-OCT-2013 18:22:53
Comments		
Input	Data	C:\Documents and Settings\Dennis Boywitt\My Documents\My Dropbox\Freiberufliche Tätigkeit\Forschungsring\Arbeitsordner Daten\Befindlichkeiten_Gruppe2_restructured.sav
	Active Dataset	DataSet2
	Filter	<none>
	Weight	<none>
	Split File	<none>
	N of Rows in Working Data File	62
Missing Value Handling	Definition of Missing	User-defined missing values are treated as missing.
	Cases Used	Statistics are based on all cases with valid data for all variables in the model.

Notes

Syntax		GLM Skala1_Naturastar.06.12.12 Skala1_Naturastar.17.01.13 Skala1_Goldblume.06.12.12 Skala1_Goldblume.17.01.13<br>/WSFACTOR=Produkt 2 Polynomial Messzeitpunkt 2 Polynomial<br>/METHOD=SSTYPE(3)<br>/EMMEANS=TABLES(Produkt)<br>/PRINT=DESCRIPTIVE ETASQ<br>/CRITERIA=ALPHA(.05)<br>/WSDESIGN=Produkt Messzeitpunkt Produkt*Messzeitpunkt.
Resources	Processor Time	00:00:00,03
	Elapsed Time	00:00:00,03

```
[DataSet2] C:\Documents and Settings\Dennis Boywitt\My Documents\My Dropbox\Freiberufliche Tätigkeit\Forschungsring\Arbeitsordner Daten\Befindlichkeiten_Gruppe2_restructured.sav
```

Within-Subjects Factors

Measure: MEASURE_1

Produkt	Messzeitpunkt	Dependent Variable
1	1	Skala1_Naturastar.06.12.12
	2	Skala1_Naturastar.17.01.13
2	1	Skala1_Goldblume.06.12.12
	2	Skala1_Goldblume.17.01.13

Descriptive Statistics

	Mean	Std. Deviation	N
Skala1_Naturastar. 06.12.12	2,3297	,85773	59
Skala1_Naturastar. 17.01.13	2,2068	,74553	59
Skala1_Goldblume. 06.12.12	2,4729	,75036	59
Skala1_Goldblume. 17.01.13	2,2271	,76063	59

Multivariate Tests[a]

Effect		Value	F	Hypothesis df	Error df
Produkt	Pillai's Trace	,018	1,080[b]	1,000	58,000
	Wilks' Lambda	,982	1,080[b]	1,000	58,000
	Hotelling's Trace	,019	1,080[b]	1,000	58,000
	Roy's Largest Root	,019	1,080[b]	1,000	58,000
Messzeitpunkt	Pillai's Trace	,091	5,805[b]	1,000	58,000
	Wilks' Lambda	,909	5,805[b]	1,000	58,000
	Hotelling's Trace	,100	5,805[b]	1,000	58,000
	Roy's Largest Root	,100	5,805[b]	1,000	58,000
Produkt * Messzeitpunkt	Pillai's Trace	,007	,403[b]	1,000	58,000
	Wilks' Lambda	,993	,403[b]	1,000	58,000
	Hotelling's Trace	,007	,403[b]	1,000	58,000
	Roy's Largest Root	,007	,403[b]	1,000	58,000

Multivariate Tests[a]

Effect		Sig.	Partial Eta Squared
Produkt	Pillai's Trace	,303	,018
	Wilks' Lambda	,303	,018
	Hotelling's Trace	,303	,018
	Roy's Largest Root	,303	,018
Messzeitpunkt	Pillai's Trace	,019	,091
	Wilks' Lambda	,019	,091
	Hotelling's Trace	,019	,091
	Roy's Largest Root	,019	,091
Produkt * Messzeitpunkt	Pillai's Trace	,528	,007
	Wilks' Lambda	,528	,007
	Hotelling's Trace	,528	,007
	Roy's Largest Root	,528	,007

a. Design: Intercept
Within Subjects Design: Produkt + Messzeitpunkt + Produkt * Messzeitpunkt

b. Exact statistic

Mauchly's Test of Sphericity[a]

Measure: MEASURE_1

Within Subjects Effect	Mauchly's W	Approx. Chi-Square	df	Sig.	Epsilon[b]
					Greenhouse-Geisser
Produkt	1,000	,000	0	.	1,000
Messzeitpunkt	1,000	,000	0	.	1,000
Produkt * Messzeitpunkt	1,000	,000	0	.	1,000

Mauchly's Test of Sphericity[a]

Measure: MEASURE_1

Within Subjects Effect	Epsilon[b]	
	Huynh-Feldt	Lower-bound
Produkt	1,000	1,000
Messzeitpunkt	1,000	1,000
Produkt * Messzeitpunkt	1,000	1,000

Tests the null hypothesis that the error covariance matrix of the orthonormalized transformed dependent variables is proportional to an identity matrix.

a. Design: Intercept
Within Subjects Design: Produkt + Messzeitpunkt + Produkt * Messzeitpunkt

b. May be used to adjust the degrees of freedom for the averaged tests of significance. Corrected tests are displayed in the Tests of Within-Subjects Effects table.

Tests of Within-Subjects Effects

Measure: MEASURE_1

Source		Type III Sum of Squares	df	Mean Square
Produkt	Sphericity Assumed	,395	1	,395
	Greenhouse-Geisser	,395	1,000	,395
	Huynh-Feldt	,395	1,000	,395
	Lower-bound	,395	1,000	,395
Error(Produkt)	Sphericity Assumed	21,199	58	,365
	Greenhouse-Geisser	21,199	58,000	,365
	Huynh-Feldt	21,199	58,000	,365
	Lower-bound	21,199	58,000	,365
Messzeitpunkt	Sphericity Assumed	2,005	1	2,005
	Greenhouse-Geisser	2,005	1,000	2,005
	Huynh-Feldt	2,005	1,000	2,005
	Lower-bound	2,005	1,000	2,005
Error(Messzeitpunkt)	Sphericity Assumed	20,029	58	,345
	Greenhouse-Geisser	20,029	58,000	,345
	Huynh-Feldt	20,029	58,000	,345
	Lower-bound	20,029	58,000	,345
Produkt * Messzeitpunkt	Sphericity Assumed	,223	1	,223
	Greenhouse-Geisser	,223	1,000	,223
	Huynh-Feldt	,223	1,000	,223
	Lower-bound	,223	1,000	,223
Error (Produkt*Messzeitpunkt)	Sphericity Assumed	32,020	58	,552
	Greenhouse-Geisser	32,020	58,000	,552
	Huynh-Feldt	32,020	58,000	,552
	Lower-bound	32,020	58,000	,552

Tests of Within-Subjects Effects

Measure: MEASURE_1

Source		F	Sig.	Partial Eta Squared
Produkt	Sphericity Assumed	1,080	,303	,018
	Greenhouse-Geisser	1,080	,303	,018
	Huynh-Feldt	1,080	,303	,018
	Lower-bound	1,080	,303	,018
Error(Produkt)	Sphericity Assumed			
	Greenhouse-Geisser			
	Huynh-Feldt			
	Lower-bound			
Messzeitpunkt	Sphericity Assumed	5,805	,019	,091
	Greenhouse-Geisser	5,805	,019	,091
	Huynh-Feldt	5,805	,019	,091
	Lower-bound	5,805	,019	,091
Error(Messzeitpunkt)	Sphericity Assumed			
	Greenhouse-Geisser			
	Huynh-Feldt			
	Lower-bound			
Produkt * Messzeitpunkt	Sphericity Assumed	,403	,528	,007
	Greenhouse-Geisser	,403	,528	,007
	Huynh-Feldt	,403	,528	,007
	Lower-bound	,403	,528	,007
Error (Produkt*Messzeitpunkt)	Sphericity Assumed			
	Greenhouse-Geisser			
	Huynh-Feldt			
	Lower-bound			

Tests of Within-Subjects Contrasts

Measure: MEASURE_1

Source	Produkt	Messzeitpunkt	Type III Sum of Squares	df	Mean Square
Produkt	Linear		,395	1	,395
Error(Produkt)	Linear		21,199	58	,365
Messzeitpunkt		Linear	2,005	1	2,005
Error(Messzeitpunkt)		Linear	20,029	58	,345
Produkt * Messzeitpunkt	Linear	Linear	,223	1	,223
Error (Produkt*Messzeitpunkt)	Linear	Linear	32,020	58	,552

Tests of Within-Subjects Contrasts

Measure: MEASURE_1

Source	Produkt	Messzeitpunkt	F	Sig.	Partial Eta Squared
Produkt	Linear		1,080	,303	,018
Error(Produkt)	Linear				
Messzeitpunkt		Linear	5,805	,019	,091
Error(Messzeitpunkt)		Linear			
Produkt * Messzeitpunkt	Linear	Linear	,403	,528	,007
Error (Produkt*Messzeitpunkt)	Linear	Linear			

Tests of Between-Subjects Effects

Measure: MEASURE_1

Transformed Variable: Average

Source	Type III Sum of Squares	df	Mean Square	F	Sig.	Partial Eta Squared
Intercept	1258,350	1	1258,350	1075,298	,000	,949
Error	67,874	58	1,170			

Estimated Marginal Means

Produkt

Measure: MEASURE_1

Produkt	Mean	Std. Error	95% Confidence Interval	
			Lower Bound	Upper Bound
1	2,268	,083	2,103	2,433
2	2,350	,079	2,192	2,508

```
GLM Skala2_Naturastar.06.12.12 Skala2_Naturastar.17.01.13 Skala2_Goldblume.06.12.12 Skala2_G
  /WSFACTOR=Produkt 2 Polynomial Messzeitpunkt 2 Polynomial
  /METHOD=SSTYPE(3)
  /EMMEANS=TABLES(Produkt)
  /PRINT=DESCRIPTIVE ETASQ
  /CRITERIA=ALPHA(.05)
  /WSDESIGN=Produkt Messzeitpunkt Produkt*Messzeitpunkt.
```

General Linear Model

Notes

Output Created		24-OCT-2013 18:25:12
Comments		
Input	Data	C:\Documents and Settings\Dennis Boywitt\My Documents\My Dropbox\Freiberufliche Tätigkeit\Forschungsring\Arbeitsordner Daten\Befindlichkeiten_Gruppe2_restructured.sav
	Active Dataset	DataSet2
	Filter	<none>
	Weight	<none>
	Split File	<none>
	N of Rows in Working Data File	62
Missing Value Handling	Definition of Missing	User-defined missing values are treated as missing.
	Cases Used	Statistics are based on all cases with valid data for all variables in the model.
Syntax		GLM Skala2_Naturastar.06.12.12 Skala2_Naturastar.17.01.13 Skala2_Goldblume.06.12.12 Skala2_Goldblume.17.01.13<br>/WSFACTOR=Produkt 2 Polynomial Messzeitpunkt 2 Polynomial<br>/METHOD=SSTYPE(3)<br>/EMMEANS=TABLES(Produkt)<br>/PRINT=DESCRIPTIVE ETASQ<br>/CRITERIA=ALPHA(.05)<br>/WSDESIGN=Produkt Messzeitpunkt Produkt*Messzeitpunkt.
Resources	Processor Time	00:00:00,02
	Elapsed Time	00:00:00,02

```
[DataSet2] C:\Documents and Settings\Dennis Boywitt\My Documents\My Dropbo
x\Freiberufliche Tätigkeit\Forschungsring\Arbeitsordner Daten\Befindlichke
iten_Gruppe2_restructured.sav
```

Within-Subjects Factors

Measure: MEASURE_1

Produkt	Messzeitpunkt	Dependent Variable
1	1	Skala2_Naturastar.06.12.12
	2	Skala2_Naturastar.17.01.13
2	1	Skala2_Goldblume.06.12.12
	2	Skala2_Goldblume.17.01.13

Descriptive Statistics

	Mean	Std. Deviation	N
Skala2_Naturastar.06.12.12	2,2466	,83206	59
Skala2_Naturastar.17.01.13	2,0746	,64956	59
Skala2_Goldblume.06.12.12	2,3627	,84321	59
Skala2_Goldblume.17.01.13	2,0610	,64247	59

Multivariate Tests[a]

Effect		Value	F	Hypothesis df	Error df
Produkt	Pillai's Trace	,008	,469[b]	1,000	58,000
	Wilks' Lambda	,992	,469[b]	1,000	58,000
	Hotelling's Trace	,008	,469[b]	1,000	58,000
	Roy's Largest Root	,008	,469[b]	1,000	58,000
Messzeitpunkt	Pillai's Trace	,145	9,823[b]	1,000	58,000
	Wilks' Lambda	,855	9,823[b]	1,000	58,000
	Hotelling's Trace	,169	9,823[b]	1,000	58,000
	Roy's Largest Root	,169	9,823[b]	1,000	58,000
Produkt * Messzeitpunkt	Pillai's Trace	,010	,614[b]	1,000	58,000
	Wilks' Lambda	,990	,614[b]	1,000	58,000
	Hotelling's Trace	,011	,614[b]	1,000	58,000
	Roy's Largest Root	,011	,614[b]	1,000	58,000

Multivariate Tests[a]

Effect		Sig.	Partial Eta Squared
Produkt	Pillai's Trace	,496	,008
	Wilks' Lambda	,496	,008
	Hotelling's Trace	,496	,008
	Roy's Largest Root	,496	,008
Messzeitpunkt	Pillai's Trace	,003	,145
	Wilks' Lambda	,003	,145
	Hotelling's Trace	,003	,145
	Roy's Largest Root	,003	,145
Produkt * Messzeitpunkt	Pillai's Trace	,437	,010
	Wilks' Lambda	,437	,010
	Hotelling's Trace	,437	,010
	Roy's Largest Root	,437	,010

a. Design: Intercept
Within Subjects Design: Produkt + Messzeitpunkt + Produkt * Messzeitpunkt

b. Exact statistic

Mauchly's Test of Sphericity[a]

Measure: MEASURE_1

Within Subjects Effect	Mauchly's W	Approx. Chi-Square	df	Sig.	Epsilon[b]
					Greenhouse-Geisser
Produkt	1,000	,000	0	.	1,000
Messzeitpunkt	1,000	,000	0	.	1,000
Produkt * Messzeitpunkt	1,000	,000	0	.	1,000

Mauchly's Test of Sphericity[a]

Measure: MEASURE_1

Within Subjects Effect	Epsilon[b]	
	Huynh-Feldt	Lower-bound
Produkt	1,000	1,000
Messzeitpunkt	1,000	1,000
Produkt * Messzeitpunkt	1,000	1,000

Tests the null hypothesis that the error covariance matrix of the orthonormalized transformed dependent variables is proportional to an identity matrix.

a. Design: Intercept
Within Subjects Design: Produkt + Messzeitpunkt + Produkt * Messzeitpunkt

b. May be used to adjust the degrees of freedom for the averaged tests of significance. Corrected tests are displayed in the Tests of Within-Subjects Effects table.

Tests of Within-Subjects Effects

Measure: MEASURE_1

Source		Type III Sum of Squares	df	Mean Square
Produkt	Sphericity Assumed	,155	1	,155
	Greenhouse-Geisser	,155	1,000	,155
	Huynh-Feldt	,155	1,000	,155
	Lower-bound	,155	1,000	,155
Error(Produkt)	Sphericity Assumed	19,171	58	,331
	Greenhouse-Geisser	19,171	58,000	,331
	Huynh-Feldt	19,171	58,000	,331
	Lower-bound	19,171	58,000	,331
Messzeitpunkt	Sphericity Assumed	3,310	1	3,310
	Greenhouse-Geisser	3,310	1,000	3,310
	Huynh-Feldt	3,310	1,000	3,310
	Lower-bound	3,310	1,000	3,310
Error(Messzeitpunkt)	Sphericity Assumed	19,545	58	,337
	Greenhouse-Geisser	19,545	58,000	,337
	Huynh-Feldt	19,545	58,000	,337
	Lower-bound	19,545	58,000	,337
Produkt * Messzeitpunkt	Sphericity Assumed	,248	1	,248
	Greenhouse-Geisser	,248	1,000	,248
	Huynh-Feldt	,248	1,000	,248
	Lower-bound	,248	1,000	,248
Error (Produkt*Messzeitpunkt)	Sphericity Assumed	23,438	58	,404
	Greenhouse-Geisser	23,438	58,000	,404
	Huynh-Feldt	23,438	58,000	,404
	Lower-bound	23,438	58,000	,404

Tests of Within-Subjects Effects

Measure: MEASURE_1

Source		F	Sig.	Partial Eta Squared
Produkt	Sphericity Assumed	,469	,496	,008
	Greenhouse-Geisser	,469	,496	,008
	Huynh-Feldt	,469	,496	,008
	Lower-bound	,469	,496	,008
Error(Produkt)	Sphericity Assumed			
	Greenhouse-Geisser			
	Huynh-Feldt			
	Lower-bound			
Messzeitpunkt	Sphericity Assumed	9,823	,003	,145
	Greenhouse-Geisser	9,823	,003	,145
	Huynh-Feldt	9,823	,003	,145
	Lower-bound	9,823	,003	,145
Error(Messzeitpunkt)	Sphericity Assumed			
	Greenhouse-Geisser			
	Huynh-Feldt			
	Lower-bound			
Produkt * Messzeitpunkt	Sphericity Assumed	,614	,437	,010
	Greenhouse-Geisser	,614	,437	,010
	Huynh-Feldt	,614	,437	,010
	Lower-bound	,614	,437	,010
Error (Produkt*Messzeitpunkt)	Sphericity Assumed			
	Greenhouse-Geisser			
	Huynh-Feldt			
	Lower-bound			

Tests of Within-Subjects Contrasts

Measure: MEASURE_1

Source	Produkt	Messzeitpunkt	Type III Sum of Squares	df	Mean Square
Produkt	Linear		,155	1	,155
Error(Produkt)	Linear		19,171	58	,331
Messzeitpunkt		Linear	3,310	1	3,310
Error(Messzeitpunkt)		Linear	19,545	58	,337
Produkt * Messzeitpunkt	Linear	Linear	,248	1	,248
Error (Produkt*Messzeitpunkt)	Linear	Linear	23,438	58	,404

Tests of Within-Subjects Contrasts

Measure: MEASURE_1

Source	Produkt	Messzeitpunkt	F	Sig.	Partial Eta Squared
Produkt	Linear		,469	,496	,008
Error(Produkt)	Linear				
Messzeitpunkt		Linear	9,823	,003	,145
Error(Messzeitpunkt)		Linear			
Produkt * Messzeitpunkt	Linear	Linear	,614	,437	,010
Error (Produkt*Messzeitpunkt)	Linear	Linear			

Tests of Between-Subjects Effects

Measure: MEASURE_1

Transformed Variable: Average

Source	Type III Sum of Squares	df	Mean Square	F	Sig.	Partial Eta Squared
Intercept	1127,985	1	1127,985	967,070	,000	,943
Error	67,651	58	1,166			

Estimated Marginal Means

Produkt

Measure: MEASURE_1

Produkt	Mean	Std. Error	95% Confidence Interval	
			Lower Bound	Upper Bound
1	2,161	,080	2,001	2,320
2	2,212	,079	2,053	2,371

```
 GLM Befindlichkeit1.Naturastar.06.12.12 Befindlichkeit1.Naturastar.17.01.13
Befindlichkeit1.Goldblume.06.12.12 Befindlichkeit1.Goldblume.17.01.13
  /WSFACTOR=Produkt 2 Polynomial Messzeitpunkt 2 Polynomial
  /METHOD=SSTYPE(3)
  /EMMEANS=TABLES(Produkt)
  /PRINT=DESCRIPTIVE ETASQ
  /CRITERIA=ALPHA(.05)
  /WSDESIGN=Produkt Messzeitpunkt Produkt*Messzeitpunkt.
```

General Linear Model

Notes

Output Created		24-OCT-2013 18:27:24
Comments		
Input	Data	C:\Documents and Settings\Dennis Boywitt\My Documents\My Dropbox\Freiberufliche Tätigkeit\Forschungsring\Arbeitsordner Daten\Befindlichkeiten_Gruppe2_restructured.sav
	Active Dataset	DataSet2
	Filter	<none>
	Weight	<none>
	Split File	<none>
	N of Rows in Working Data File	62
Missing Value Handling	Definition of Missing	User-defined missing values are treated as missing.
	Cases Used	Statistics are based on all cases with valid data for all variables in the model.
Syntax		GLM Befindlichkeit1.Naturastar.06.12.12 Befindlichkeit1.Naturastar.17.01.13 Befindlichkeit1.Goldblume.06.12.12 Befindlichkeit1.Goldblume.17.01.13<br>/WSFACTOR=Produkt 2 Polynomial Messzeitpunkt 2 Polynomial<br>/METHOD=SSTYPE(3)<br>/EMMEANS=TABLES(Produkt)<br>/PRINT=DESCRIPTIVE ETASQ<br>/CRITERIA=ALPHA(.05)<br>/WSDESIGN=Produkt Messzeitpunkt Produkt*Messzeitpunkt.
Resources	Processor Time	00:00:00,02
	Elapsed Time	00:00:00,02

```
[DataSet2] C:\Documents and Settings\Dennis Boywitt\My Documents\My Dropbox\Freiberufliche Tätigkeit\Forschungsring\Arbeitsordner Daten\Befindlichkeiten_Gruppe2_restructured.sav
```

Within-Subjects Factors

Measure: MEASURE_1

Produkt	Messzeitpunkt	Dependent Variable
1	1	Befindlichkeit1.Naturastar.06.12.12
	2	Befindlichkeit1.Naturastar.17.01.13
2	1	Befindlichkeit1.Goldblume.06.12.12
	2	Befindlichkeit1.Goldblume.17.01.13

Descriptive Statistics

	Mean	Std. Deviation	N
Befindlichkeit1.Naturastar.06.12.12: Ich empfinde meinen Leib 1	2,80	1,156	59
Befindlichkeit1.Naturastar.17.01.13: Ich empfinde meinen Leib 1	2,32	,973	59
Befindlichkeit1.Goldblume.06.12.12: Ich empfinde meinen Leib 1	2,61	1,130	59
Befindlichkeit1.Goldblume.17.01.13: Ich empfinde meinen Leib 1	2,56	1,055	59

Multivariate Tests[a]

Effect		Value	F	Hypothesis df	Error df
Produkt	Pillai's Trace	,001	,047[b]	1,000	58,000
	Wilks' Lambda	,999	,047[b]	1,000	58,000
	Hotelling's Trace	,001	,047[b]	1,000	58,000
	Roy's Largest Root	,001	,047[b]	1,000	58,000
Messzeitpunkt	Pillai's Trace	,062	3,829[b]	1,000	58,000
	Wilks' Lambda	,938	3,829[b]	1,000	58,000
	Hotelling's Trace	,066	3,829[b]	1,000	58,000
	Roy's Largest Root	,066	3,829[b]	1,000	58,000
Produkt * Messzeitpunkt	Pillai's Trace	,061	3,737[b]	1,000	58,000
	Wilks' Lambda	,939	3,737[b]	1,000	58,000
	Hotelling's Trace	,064	3,737[b]	1,000	58,000
	Roy's Largest Root	,064	3,737[b]	1,000	58,000

Multivariate Tests[a]

Effect		Sig.	Partial Eta Squared
Produkt	Pillai's Trace	,829	,001
	Wilks' Lambda	,829	,001
	Hotelling's Trace	,829	,001
	Roy's Largest Root	,829	,001
Messzeitpunkt	Pillai's Trace	,055	,062
	Wilks' Lambda	,055	,062
	Hotelling's Trace	,055	,062
	Roy's Largest Root	,055	,062
Produkt * Messzeitpunkt	Pillai's Trace	,058	,061
	Wilks' Lambda	,058	,061
	Hotelling's Trace	,058	,061
	Roy's Largest Root	,058	,061

a. Design: Intercept
Within Subjects Design: Produkt + Messzeitpunkt + Produkt * Messzeitpunkt

b. Exact statistic

Mauchly's Test of Sphericity[a]

Measure: MEASURE_1

Within Subjects Effect	Mauchly's W	Approx. Chi-Square	df	Sig.	Epsilon[b]
					Greenhouse-Geisser
Produkt	1,000	,000	0	.	1,000
Messzeitpunkt	1,000	,000	0	.	1,000
Produkt * Messzeitpunkt	1,000	,000	0	.	1,000

Mauchly's Test of Sphericity[a]

Measure: MEASURE_1

Within Subjects Effect	Epsilon[b]	
	Huynh-Feldt	Lower-bound
Produkt	1,000	1,000
Messzeitpunkt	1,000	1,000
Produkt * Messzeitpunkt	1,000	1,000

Tests the null hypothesis that the error covariance matrix of the orthonormalized transformed dependent variables is proportional to an identity matrix.

a. Design: Intercept
Within Subjects Design: Produkt + Messzeitpunkt + Produkt * Messzeitpunkt

b. May be used to adjust the degrees of freedom for the averaged tests of significance. Corrected tests are displayed in the Tests of Within-Subjects Effects table.

Tests of Within-Subjects Effects

Measure: MEASURE_1

Source		Type III Sum of Squares	df	Mean Square
Produkt	Sphericity Assumed	,038	1	,038
	Greenhouse-Geisser	,038	1,000	,038
	Huynh-Feldt	,038	1,000	,038
	Lower-bound	,038	1,000	,038
Error(Produkt)	Sphericity Assumed	46,712	58	,805
	Greenhouse-Geisser	46,712	58,000	,805
	Huynh-Feldt	46,712	58,000	,805
	Lower-bound	46,712	58,000	,805
Messzeitpunkt	Sphericity Assumed	4,072	1	4,072
	Greenhouse-Geisser	4,072	1,000	4,072
	Huynh-Feldt	4,072	1,000	4,072
	Lower-bound	4,072	1,000	4,072
Error(Messzeitpunkt)	Sphericity Assumed	61,678	58	1,063
	Greenhouse-Geisser	61,678	58,000	1,063
	Huynh-Feldt	61,678	58,000	1,063
	Lower-bound	61,678	58,000	1,063
Produkt * Messzeitpunkt	Sphericity Assumed	2,648	1	2,648
	Greenhouse-Geisser	2,648	1,000	2,648
	Huynh-Feldt	2,648	1,000	2,648
	Lower-bound	2,648	1,000	2,648
Error (Produkt*Messzeitpunkt)	Sphericity Assumed	41,102	58	,709
	Greenhouse-Geisser	41,102	58,000	,709
	Huynh-Feldt	41,102	58,000	,709
	Lower-bound	41,102	58,000	,709

Tests of Within-Subjects Effects

Measure: MEASURE_1

Source		F	Sig.	Partial Eta Squared
Produkt	Sphericity Assumed	,047	,829	,001
	Greenhouse-Geisser	,047	,829	,001
	Huynh-Feldt	,047	,829	,001
	Lower-bound	,047	,829	,001
Error(Produkt)	Sphericity Assumed			
	Greenhouse-Geisser			
	Huynh-Feldt			
	Lower-bound			
Messzeitpunkt	Sphericity Assumed	3,829	,055	,062
	Greenhouse-Geisser	3,829	,055	,062
	Huynh-Feldt	3,829	,055	,062
	Lower-bound	3,829	,055	,062
Error(Messzeitpunkt)	Sphericity Assumed			
	Greenhouse-Geisser			
	Huynh-Feldt			
	Lower-bound			
Produkt * Messzeitpunkt	Sphericity Assumed	3,737	,058	,061
	Greenhouse-Geisser	3,737	,058	,061
	Huynh-Feldt	3,737	,058	,061
	Lower-bound	3,737	,058	,061
Error (Produkt*Messzeitpunkt)	Sphericity Assumed			
	Greenhouse-Geisser			
	Huynh-Feldt			
	Lower-bound			

Tests of Within-Subjects Contrasts

Measure: MEASURE_1

Source	Produkt	Messzeitpunkt	Type III Sum of Squares	df	Mean Square
Produkt	Linear		,038	1	,038
Error(Produkt)	Linear		46,712	58	,805
Messzeitpunkt		Linear	4,072	1	4,072
Error(Messzeitpunkt)		Linear	61,678	58	1,063
Produkt * Messzeitpunkt	Linear	Linear	2,648	1	2,648
Error (Produkt*Messzeitpunkt)	Linear	Linear	41,102	58	,709

Tests of Within-Subjects Contrasts

Measure: MEASURE_1

Source	Produkt	Messzeitpunkt	F	Sig.	Partial Eta Squared
Produkt	Linear		,047	,829	,001
Error(Produkt)	Linear				
Messzeitpunkt		Linear	3,829	,055	,062
Error(Messzeitpunkt)		Linear			
Produkt * Messzeitpunkt	Linear	Linear	3,737	,058	,061
Error (Produkt*Messzeitpunkt)	Linear	Linear			

Tests of Between-Subjects Effects

Measure: MEASURE_1

Transformed Variable: Average

Source	Type III Sum of Squares	df	Mean Square	F	Sig.	Partial Eta Squared
Intercept	1561,225	1	1561,225	745,120	,000	,928
Error	121,525	58	2,095			

Estimated Marginal Means

Produkt

Measure: MEASURE_1

Produkt	Mean	Std. Error	95% Confidence Interval	
			Lower Bound	Upper Bound
1	2,559	,113	2,334	2,785
2	2,585	,109	2,367	2,803

```
 GLM Befindlichkeit12.Naturastar.06.12.12 Befindlichkeit12.Naturastar.17.01.13
Befindlichkeit12.Goldblume.06.12.12 Befindlichkeit12.Goldblume.17.01.13
  /WSFACTOR=Produkt 2 Polynomial Messzeitpunkt 2 Polynomial
  /METHOD=SSTYPE(3)
  /EMMEANS=TABLES(Produkt)
  /PRINT=DESCRIPTIVE ETASQ
  /CRITERIA=ALPHA(.05)
  /WSDESIGN=Produkt Messzeitpunkt Produkt*Messzeitpunkt.
```

General Linear Model

Notes

Output Created		24-OCT-2013 18:31:07
Comments		
Input	Data	C:\Documents and Settings\Dennis Boywitt\My Documents\My Dropbox\Freiberufliche Tätigkeit\Forschungsring\Arbeitsordner Daten\Befindlichkeiten_Gruppe2_restructured.sav
	Active Dataset	DataSet2
	Filter	<none>
	Weight	<none>
	Split File	<none>
	N of Rows in Working Data File	62
Missing Value Handling	Definition of Missing	User-defined missing values are treated as missing.
	Cases Used	Statistics are based on all cases with valid data for all variables in the model.
Syntax		GLM Befindlichkeit12. Naturastar.06.12.12 Befindlichkeit12. Naturastar.17.01.13 Befindlichkeit12. Goldblume.06.12.12 Befindlichkeit12. Goldblume.17.01.13 /WSFACTOR=Produkt 2 Polynomial Messzeitpunkt 2 Polynomial /METHOD=SSTYPE(3) /EMMEANS=TABLES (Produkt) /PRINT=DESCRIPTIVE ETASQ /CRITERIA=ALPHA(.05) /WSDESIGN=Produkt Messzeitpunkt Produkt*Messzeitpunkt.
Resources	Processor Time	00:00:00,02
	Elapsed Time	00:00:00,02

```
[DataSet2] C:\Documents and Settings\Dennis Boywitt\My Documents\My Dropbox\Freiberufliche Tätigkeit\Forschungsring\Arbeitsordner Daten\Befindlichkeiten_Gruppe2_restructured.sav
```

Within-Subjects Factors

Measure: MEASURE_1

Produkt	Messzeitpunkt	Dependent Variable
1	1	Befindlichkeit12.Naturastar.06.12.12
	2	Befindlichkeit12.Naturastar.17.01.13
2	1	Befindlichkeit12.Goldblume.06.12.12
	2	Befindlichkeit12.Goldblume.17.01.13

Descriptive Statistics

	Mean	Std. Deviation	N
Befindlichkeit12. Naturastar.06.12.12: Ich empfinde die Wirkung	2,49	1,023	59
Befindlichkeit12. Naturastar.17.01.13: Ich empfinde die Wirkung	2,54	,953	59
Befindlichkeit12. Goldblume.06.12.12: Ich empfinde die Wirkung	2,61	,965	59
Befindlichkeit12. Goldblume.17.01.13: Ich empfinde die Wirkung	2,49	,954	59

Multivariate Tests[a]

Effect		Value	F	Hypothesis df	Error df
Produkt	Pillai's Trace	,002	,088[b]	1,000	58,000
	Wilks' Lambda	,998	,088[b]	1,000	58,000
	Hotelling's Trace	,002	,088[b]	1,000	58,000
	Roy's Largest Root	,002	,088[b]	1,000	58,000
Messzeitpunkt	Pillai's Trace	,001	,065[b]	1,000	58,000
	Wilks' Lambda	,999	,065[b]	1,000	58,000
	Hotelling's Trace	,001	,065[b]	1,000	58,000
	Roy's Largest Root	,001	,065[b]	1,000	58,000
Produkt * Messzeitpunkt	Pillai's Trace	,011	,629[b]	1,000	58,000
	Wilks' Lambda	,989	,629[b]	1,000	58,000
	Hotelling's Trace	,011	,629[b]	1,000	58,000
	Roy's Largest Root	,011	,629[b]	1,000	58,000

Multivariate Tests[a]

Effect		Sig.	Partial Eta Squared
Produkt	Pillai's Trace	,767	,002
	Wilks' Lambda	,767	,002
	Hotelling's Trace	,767	,002
	Roy's Largest Root	,767	,002
Messzeitpunkt	Pillai's Trace	,800	,001
	Wilks' Lambda	,800	,001
	Hotelling's Trace	,800	,001
	Roy's Largest Root	,800	,001
Produkt * Messzeitpunkt	Pillai's Trace	,431	,011
	Wilks' Lambda	,431	,011
	Hotelling's Trace	,431	,011
	Roy's Largest Root	,431	,011

a. Design: Intercept
Within Subjects Design: Produkt + Messzeitpunkt + Produkt * Messzeitpunkt

b. Exact statistic

Mauchly's Test of Sphericity[a]

Measure: MEASURE_1

Within Subjects Effect	Mauchly's W	Approx. Chi-Square	df	Sig.	Epsilon[b] Greenhouse-Geisser
Produkt	1,000	,000	0	.	1,000
Messzeitpunkt	1,000	,000	0	.	1,000
Produkt * Messzeitpunkt	1,000	,000	0	.	1,000

Mauchly's Test of Sphericity[a]

Measure: MEASURE_1

Within Subjects Effect	Epsilon[b]	
	Huynh-Feldt	Lower-bound
Produkt	1,000	1,000
Messzeitpunkt	1,000	1,000
Produkt * Messzeitpunkt	1,000	1,000

Tests the null hypothesis that the error covariance matrix of the orthonormalized transformed dependent variables is proportional to an identity matrix.

a. Design: Intercept
Within Subjects Design: Produkt + Messzeitpunkt + Produkt * Messzeitpunkt

b. May be used to adjust the degrees of freedom for the averaged tests of significance. Corrected tests are displayed in the Tests of Within-Subjects Effects table.

Tests of Within-Subjects Effects

Measure: MEASURE_1

Source		Type III Sum of Squares	df	Mean Square
Produkt	Sphericity Assumed	,068	1	,068
	Greenhouse-Geisser	,068	1,000	,068
	Huynh-Feldt	,068	1,000	,068
	Lower-bound	,068	1,000	,068
Error(Produkt)	Sphericity Assumed	44,432	58	,766
	Greenhouse-Geisser	44,432	58,000	,766
	Huynh-Feldt	44,432	58,000	,766
	Lower-bound	44,432	58,000	,766
Messzeitpunkt	Sphericity Assumed	,068	1	,068
	Greenhouse-Geisser	,068	1,000	,068
	Huynh-Feldt	,068	1,000	,068
	Lower-bound	,068	1,000	,068
Error(Messzeitpunkt)	Sphericity Assumed	60,432	58	1,042
	Greenhouse-Geisser	60,432	58,000	1,042
	Huynh-Feldt	60,432	58,000	1,042
	Lower-bound	60,432	58,000	1,042
Produkt * Messzeitpunkt	Sphericity Assumed	,424	1	,424
	Greenhouse-Geisser	,424	1,000	,424
	Huynh-Feldt	,424	1,000	,424
	Lower-bound	,424	1,000	,424
Error (Produkt*Messzeitpunkt)	Sphericity Assumed	39,076	58	,674
	Greenhouse-Geisser	39,076	58,000	,674
	Huynh-Feldt	39,076	58,000	,674
	Lower-bound	39,076	58,000	,674

Tests of Within-Subjects Effects

Measure: MEASURE_1

Source		F	Sig.	Partial Eta Squared
Produkt	Sphericity Assumed	,088	,767	,002
	Greenhouse-Geisser	,088	,767	,002
	Huynh-Feldt	,088	,767	,002
	Lower-bound	,088	,767	,002
Error(Produkt)	Sphericity Assumed			
	Greenhouse-Geisser			
	Huynh-Feldt			
	Lower-bound			
Messzeitpunkt	Sphericity Assumed	,065	,800	,001
	Greenhouse-Geisser	,065	,800	,001
	Huynh-Feldt	,065	,800	,001
	Lower-bound	,065	,800	,001
Error(Messzeitpunkt)	Sphericity Assumed			
	Greenhouse-Geisser			
	Huynh-Feldt			
	Lower-bound			
Produkt * Messzeitpunkt	Sphericity Assumed	,629	,431	,011
	Greenhouse-Geisser	,629	,431	,011
	Huynh-Feldt	,629	,431	,011
	Lower-bound	,629	,431	,011
Error (Produkt*Messzeitpunkt)	Sphericity Assumed			
	Greenhouse-Geisser			
	Huynh-Feldt			
	Lower-bound			

Tests of Within-Subjects Contrasts

Measure: MEASURE_1

Source	Produkt	Messzeitpunkt	Type III Sum of Squares	df	Mean Square
Produkt	Linear		,068	1	,068
Error(Produkt)	Linear		44,432	58	,766
Messzeitpunkt		Linear	,068	1	,068
Error(Messzeitpunkt)		Linear	60,432	58	1,042
Produkt * Messzeitpunkt	Linear	Linear	,424	1	,424
Error (Produkt*Messzeitpunkt)	Linear	Linear	39,076	58	,674

Tests of Within-Subjects Contrasts

Measure: MEASURE_1

Source	Produkt	Messzeitpunkt	F	Sig.	Partial Eta Squared
Produkt	Linear		,088	,767	,002
Error(Produkt)	Linear				
Messzeitpunkt		Linear	,065	,800	,001
Error(Messzeitpunkt)		Linear			
Produkt * Messzeitpunkt	Linear	Linear	,629	,431	,011
Error (Produkt*Messzeitpunkt)	Linear	Linear			

Tests of Between-Subjects Effects

Measure: MEASURE_1

Transformed Variable: Average

Source	Type III Sum of Squares	df	Mean Square	F	Sig.	Partial Eta Squared
Intercept	1515,271	1	1515,271	1152,920	,000	,952
Error	76,229	58	1,314			

Estimated Marginal Means

Produkt

Measure: MEASURE_1

Produkt	Mean	Std. Error	95% Confidence Interval	
			Lower Bound	Upper Bound
1	2,517	,094	2,330	2,704
2	2,551	,094	2,362	2,739
