# Supplementary material for: Development and Application of a Test for Food-Induced Emotions
Source: PLoS One. 2016 Nov 18;11(11):e0165991. doi: 10.1371/journal.pone.0165991 (PMC5115674; doi:10.1371/journal.pone.0165991)
Supplement: S8 File — (PDF) [file pone.0165991.s011.pdf]

```
GLM Skala1_Diamant.06.12.12 Skala1_Diamant.17.01.13 Skala1_Guarani.06.12.12 Skala1_Guarani.1
  /WSFACTOR=Produkt 2 Polynomial Messzeitpunkt 2 Polynomial
  /METHOD=SSTYPE(3)
  /EMMEANS=TABLES(Produkt)
  /PRINT=DESCRIPTIVE ETASQ
  /CRITERIA=ALPHA(.05)
  /WSDESIGN=Produkt Messzeitpunkt Produkt*Messzeitpunkt.
```

General Linear Model

Notes

Output Created		29-OCT-2013 12:18:00
Comments		
Input	Data	C:\Documents and Settings\Dennis Boywitt\My Documents\My Dropbox\Freiberufliche Tätigkeit\Forschungsring\Arbeitsordner Daten\Befindlichkeiten_Gruppe2_restructured.sav
	Active Dataset	DataSet2
	Filter	<none>
	Weight	<none>
	Split File	<none>
	N of Rows in Working Data File	62
Missing Value Handling	Definition of Missing	User-defined missing values are treated as missing.
	Cases Used	Statistics are based on all cases with valid data for all variables in the model.
Syntax		GLM Skala1_Diamant.06.12.12 Skala1_Diamant.17.01.13 Skala1_Guarani.06.12.12 Skala1_Guarani.17.01.13 /WSFACTOR=Produkt 2 Polynomial Messzeitpunkt 2 Polynomial /METHOD=SSTYPE(3) /EMMEANS=TABLES(Produkt) /PRINT=DESCRIPTIVE ETASQ /CRITERIA=ALPHA(.05) /WSDESIGN=Produkt Messzeitpunkt Produkt*Messzeitpunkt.

Notes

Resources	Processor Time	00:00:00,03
	Elapsed Time	00:00:00,05

```
[DataSet2] C:\Documents and Settings\Dennis Boywitt\My Documents\My Dropbo
x\Freiberufliche Tätigkeit\Forschungsring\Arbeitsordner Daten\Befindlichke
iten_Gruppe2_restructured.sav
```

Within-Subjects Factors

Measure: MEASURE_1

Produkt	Messzeitpunkt	Dependent Variable
1	1	Skala1_Diamant.06.12.12
	2	Skala1_Diamant.17.01.13
2	1	Skala1_Guarani.06.12.12
	2	Skala1_Guarani.17.01.13

Descriptive Statistics

	Mean	Std. Deviation	N
Skala1_Diamant.06.12.12	2,1729	,77767	59
Skala1_Diamant.17.01.13	2,1966	,87454	59
Skala1_Guarani.06.12.12	2,0237	,76301	59
Skala1_Guarani.17.01.13	1,9593	,79635	59

Multivariate Tests[a]

Effect		Value	F	Hypothesis df	Error df
Produkt	Pillai's Trace	,061	3,785[b]	1,000	58,000
	Wilks' Lambda	,939	3,785[b]	1,000	58,000
	Hotelling's Trace	,065	3,785[b]	1,000	58,000
	Roy's Largest Root	,065	3,785[b]	1,000	58,000
Messzeitpunkt	Pillai's Trace	,001	,065[b]	1,000	58,000
	Wilks' Lambda	,999	,065[b]	1,000	58,000
	Hotelling's Trace	,001	,065[b]	1,000	58,000
	Roy's Largest Root	,001	,065[b]	1,000	58,000
Produkt * Messzeitpunkt	Pillai's Trace	,005	,317[b]	1,000	58,000
	Wilks' Lambda	,995	,317[b]	1,000	58,000
	Hotelling's Trace	,005	,317[b]	1,000	58,000
	Roy's Largest Root	,005	,317[b]	1,000	58,000

Multivariate Tests[a]

Effect		Sig.	Partial Eta Squared
Produkt	Pillai's Trace	,057	,061
	Wilks' Lambda	,057	,061
	Hotelling's Trace	,057	,061
	Roy's Largest Root	,057	,061
Messzeitpunkt	Pillai's Trace	,799	,001
	Wilks' Lambda	,799	,001
	Hotelling's Trace	,799	,001
	Roy's Largest Root	,799	,001
Produkt * Messzeitpunkt	Pillai's Trace	,576	,005
	Wilks' Lambda	,576	,005
	Hotelling's Trace	,576	,005
	Roy's Largest Root	,576	,005

a. Design: Intercept
Within Subjects Design: Produkt + Messzeitpunkt + Produkt * Messzeitpunkt

b. Exact statistic

Mauchly's Test of Sphericity[a]

Measure: MEASURE_1

Within Subjects Effect	Mauchly's W	Approx. Chi-Square	df	Sig.	Epsilon[b]
					Greenhouse-Geisser
Produkt	1,000	,000	0	.	1,000
Messzeitpunkt	1,000	,000	0	.	1,000
Produkt * Messzeitpunkt	1,000	,000	0	.	1,000

Mauchly's Test of Sphericity[a]

Measure: MEASURE_1

Within Subjects Effect	Epsilon[b]	
	Huynh-Feldt	Lower-bound
Produkt	1,000	1,000
Messzeitpunkt	1,000	1,000
Produkt * Messzeitpunkt	1,000	1,000

Tests the null hypothesis that the error covariance matrix of the orthonormalized transformed dependent variables is proportional to an identity matrix.

a. Design: Intercept
Within Subjects Design: Produkt + Messzeitpunkt + Produkt * Messzeitpunkt

b. May be used to adjust the degrees of freedom for the averaged tests of significance. Corrected tests are displayed in the Tests of Within-Subjects Effects table.

Tests of Within-Subjects Effects

Measure: MEASURE_1

Source		Type III Sum of Squares	df	Mean Square
Produkt	Sphericity Assumed	2,203	1	2,203
	Greenhouse-Geisser	2,203	1,000	2,203
	Huynh-Feldt	2,203	1,000	2,203
	Lower-bound	2,203	1,000	2,203
Error(Produkt)	Sphericity Assumed	33,757	58	,582
	Greenhouse-Geisser	33,757	58,000	,582
	Huynh-Feldt	33,757	58,000	,582
	Lower-bound	33,757	58,000	,582
Messzeitpunkt	Sphericity Assumed	,024	1	,024
	Greenhouse-Geisser	,024	1,000	,024
	Huynh-Feldt	,024	1,000	,024
	Lower-bound	,024	1,000	,024
Error(Messzeitpunkt)	Sphericity Assumed	21,616	58	,373
	Greenhouse-Geisser	21,616	58,000	,373
	Huynh-Feldt	21,616	58,000	,373
	Lower-bound	21,616	58,000	,373
Produkt * Messzeitpunkt	Sphericity Assumed	,115	1	,115
	Greenhouse-Geisser	,115	1,000	,115
	Huynh-Feldt	,115	1,000	,115
	Lower-bound	,115	1,000	,115
Error (Produkt*Messzeitpunkt)	Sphericity Assumed	20,965	58	,361
	Greenhouse-Geisser	20,965	58,000	,361
	Huynh-Feldt	20,965	58,000	,361
	Lower-bound	20,965	58,000	,361

Tests of Within-Subjects Effects

Measure: MEASURE_1

Source		F	Sig.	Partial Eta Squared
Produkt	Sphericity Assumed	3,785	,057	,061
	Greenhouse-Geisser	3,785	,057	,061
	Huynh-Feldt	3,785	,057	,061
	Lower-bound	3,785	,057	,061
Error(Produkt)	Sphericity Assumed			
	Greenhouse-Geisser			
	Huynh-Feldt			
	Lower-bound			
Messzeitpunkt	Sphericity Assumed	,065	,799	,001
	Greenhouse-Geisser	,065	,799	,001
	Huynh-Feldt	,065	,799	,001
	Lower-bound	,065	,799	,001
Error(Messzeitpunkt)	Sphericity Assumed			
	Greenhouse-Geisser			
	Huynh-Feldt			
	Lower-bound			
Produkt * Messzeitpunkt	Sphericity Assumed	,317	,576	,005
	Greenhouse-Geisser	,317	,576	,005
	Huynh-Feldt	,317	,576	,005
	Lower-bound	,317	,576	,005
Error (Produkt*Messzeitpunkt)	Sphericity Assumed			
	Greenhouse-Geisser			
	Huynh-Feldt			
	Lower-bound			

Tests of Within-Subjects Contrasts

Measure: MEASURE_1

Source	Produkt	Messzeitpunkt	Type III Sum of Squares	df	Mean Square
Produkt	Linear		2,203	1	2,203
Error(Produkt)	Linear		33,757	58	,582
Messzeitpunkt		Linear	,024	1	,024
Error(Messzeitpunkt)		Linear	21,616	58	,373
Produkt * Messzeitpunkt	Linear	Linear	,115	1	,115
Error (Produkt*Messzeitpunkt)	Linear	Linear	20,965	58	,361

Tests of Within-Subjects Contrasts

Measure: MEASURE_1

Source	Produkt	Messzeitpunkt	F	Sig.	Partial Eta Squared
Produkt	Linear		3,785	,057	,061
Error(Produkt)	Linear				
Messzeitpunkt		Linear	,065	,799	,001
Error(Messzeitpunkt)		Linear			
Produkt * Messzeitpunkt	Linear	Linear	,317	,576	,005
Error (Produkt*Messzeitpunkt)	Linear	Linear			

Tests of Between-Subjects Effects

Measure: MEASURE_1

Transformed Variable: Average

Source	Type III Sum of Squares	df	Mean Square	F	Sig.	Partial Eta Squared
Intercept	1029,033	1	1029,033	810,408	,000	,933
Error	73,647	58	1,270			

Estimated Marginal Means

Produkt

Measure: MEASURE_1

Produkt	Mean	Std. Error	95% Confidence Interval	
			Lower Bound	Upper Bound
1	2,185	,088	2,009	2,361
2	1,992	,089	1,813	2,170

```
GLM Skala2_Diamant.06.12.12 Skala2_Diamant.17.01.13 Skala2_Guarani.06.12.12 Skala2_Guarani.1
  /WSFACTOR=Produkt 2 Polynomial Messzeitpunkt 2 Polynomial
  /METHOD=SSTYPE(3)
  /EMMEANS=TABLES(Produkt)
  /PRINT=DESCRIPTIVE ETASQ
  /CRITERIA=ALPHA(.05)
  /WSDESIGN=Produkt Messzeitpunkt Produkt*Messzeitpunkt.
```

General Linear Model

Notes

Output Created		29-OCT-2013 12:19:40
Comments		
Input	Data	C:\Documents and Settings\Dennis Boywitt\My Documents\My Dropbox\Freiberufliche Tätigkeit\Forschungsring\Arbeitsordner Daten\Befindlichkeiten_Gruppe2_restructured.sav
	Active Dataset	DataSet2
	Filter	<none>
	Weight	<none>
	Split File	<none>
	N of Rows in Working Data File	62
Missing Value Handling	Definition of Missing	User-defined missing values are treated as missing.
	Cases Used	Statistics are based on all cases with valid data for all variables in the model.
Syntax		GLM Skala2_Diamant. 06.12.12 Skala2_Diamant. 17.01.13 Skala2_Guarani. 06.12.12 Skala2_Guarani. 17.01.13<br>/WSFACTOR=Produkt 2 Polynomial Messzeitpunkt 2 Polynomial<br>/METHOD=SSTYPE(3)<br>/EMMEANS=TABLES (Produkt)<br>/PRINT=DESCRIPTIVE ETASQ<br>/CRITERIA=ALPHA(.05)<br>/WSDESIGN=Produkt Messzeitpunkt Produkt*Messzeitpunkt.
Resources	Processor Time	00:00:00,03
	Elapsed Time	00:00:00,03

```
[DataSet2] C:\Documents and Settings\Dennis Boywitt\My Documents\My Dropbox\Freiberufliche Tätigkeit\Forschungsring\Arbeitsordner Daten\Befindlichkeiten_Gruppe2_restructured.sav
```

Within-Subjects Factors

Measure: MEASURE_1

Produkt	Messzeitpunkt	Dependent Variable
1	1	Skala2_Diamant.06.12.12
	2	Skala2_Diamant.17.01.13
2	1	Skala2_Guarani.06.12.12
	2	Skala2_Guarani.17.01.13

Descriptive Statistics

	Mean	Std. Deviation	N
Skala2_Diamant.06.12.12	2,1729	,76244	59
Skala2_Diamant.17.01.13	2,0475	,81481	59
Skala2_Guarani.06.12.12	2,0373	,83334	59
Skala2_Guarani.17.01.13	1,8576	,67879	59

Multivariate Tests[a]

Effect		Value	F	Hypothesis df	Error df
Produkt	Pillai's Trace	,049	2,980[b]	1,000	58,000
	Wilks' Lambda	,951	2,980[b]	1,000	58,000
	Hotelling's Trace	,051	2,980[b]	1,000	58,000
	Roy's Largest Root	,051	2,980[b]	1,000	58,000
Messzeitpunkt	Pillai's Trace	,066	4,116[b]	1,000	58,000
	Wilks' Lambda	,934	4,116[b]	1,000	58,000
	Hotelling's Trace	,071	4,116[b]	1,000	58,000
	Roy's Largest Root	,071	4,116[b]	1,000	58,000
Produkt * Messzeitpunkt	Pillai's Trace	,004	,206[b]	1,000	58,000
	Wilks' Lambda	,996	,206[b]	1,000	58,000
	Hotelling's Trace	,004	,206[b]	1,000	58,000
	Roy's Largest Root	,004	,206[b]	1,000	58,000

Multivariate Tests[a]

Effect		Sig.	Partial Eta Squared
Produkt	Pillai's Trace	,090	,049
	Wilks' Lambda	,090	,049
	Hotelling's Trace	,090	,049
	Roy's Largest Root	,090	,049
Messzeitpunkt	Pillai's Trace	,047	,066
	Wilks' Lambda	,047	,066
	Hotelling's Trace	,047	,066
	Roy's Largest Root	,047	,066
Produkt * Messzeitpunkt	Pillai's Trace	,652	,004
	Wilks' Lambda	,652	,004
	Hotelling's Trace	,652	,004
	Roy's Largest Root	,652	,004

a. Design: Intercept
Within Subjects Design: Produkt + Messzeitpunkt + Produkt * Messzeitpunkt

b. Exact statistic

Mauchly's Test of Sphericity[a]

Measure: MEASURE_1

Within Subjects Effect	Mauchly's W	Approx. Chi-Square	df	Sig.	Epsilon[b]
					Greenhouse-Geisser
Produkt	1,000	,000	0	.	1,000
Messzeitpunkt	1,000	,000	0	.	1,000
Produkt * Messzeitpunkt	1,000	,000	0	.	1,000

Mauchly's Test of Sphericity[a]

Measure: MEASURE_1

Within Subjects Effect	Epsilon[b]	
	Huynh-Feldt	Lower-bound
Produkt	1,000	1,000
Messzeitpunkt	1,000	1,000
Produkt * Messzeitpunkt	1,000	1,000

Tests the null hypothesis that the error covariance matrix of the orthonormalized transformed dependent variables is proportional to an identity matrix.

a. Design: Intercept
Within Subjects Design: Produkt + Messzeitpunkt + Produkt * Messzeitpunkt

b. May be used to adjust the degrees of freedom for the averaged tests of significance. Corrected tests are displayed in the Tests of Within-Subjects Effects table.

Tests of Within-Subjects Effects

Measure: MEASURE_1

Source		Type III Sum of Squares	df	Mean Square
Produkt	Sphericity Assumed	1,562	1	1,562
	Greenhouse-Geisser	1,562	1,000	1,562
	Huynh-Feldt	1,562	1,000	1,562
	Lower-bound	1,562	1,000	1,562
Error(Produkt)	Sphericity Assumed	30,398	58	,524
	Greenhouse-Geisser	30,398	58,000	,524
	Huynh-Feldt	30,398	58,000	,524
	Lower-bound	30,398	58,000	,524
Messzeitpunkt	Sphericity Assumed	1,373	1	1,373
	Greenhouse-Geisser	1,373	1,000	1,373
	Huynh-Feldt	1,373	1,000	1,373
	Lower-bound	1,373	1,000	1,373
Error(Messzeitpunkt)	Sphericity Assumed	19,347	58	,334
	Greenhouse-Geisser	19,347	58,000	,334
	Huynh-Feldt	19,347	58,000	,334
	Lower-bound	19,347	58,000	,334
Produkt * Messzeitpunkt	Sphericity Assumed	,043	1	,043
	Greenhouse-Geisser	,043	1,000	,043
	Huynh-Feldt	,043	1,000	,043
	Lower-bound	,043	1,000	,043
Error (Produkt*Messzeitpunkt)	Sphericity Assumed	12,237	58	,211
	Greenhouse-Geisser	12,237	58,000	,211
	Huynh-Feldt	12,237	58,000	,211
	Lower-bound	12,237	58,000	,211

Tests of Within-Subjects Effects

Measure: MEASURE_1

Source		F	Sig.	Partial Eta Squared
Produkt	Sphericity Assumed	2,980	,090	,049
	Greenhouse-Geisser	2,980	,090	,049
	Huynh-Feldt	2,980	,090	,049
	Lower-bound	2,980	,090	,049
Error(Produkt)	Sphericity Assumed			
	Greenhouse-Geisser			
	Huynh-Feldt			
	Lower-bound			
Messzeitpunkt	Sphericity Assumed	4,116	,047	,066
	Greenhouse-Geisser	4,116	,047	,066
	Huynh-Feldt	4,116	,047	,066
	Lower-bound	4,116	,047	,066
Error(Messzeitpunkt)	Sphericity Assumed			
	Greenhouse-Geisser			
	Huynh-Feldt			
	Lower-bound			
Produkt * Messzeitpunkt	Sphericity Assumed	,206	,652	,004
	Greenhouse-Geisser	,206	,652	,004
	Huynh-Feldt	,206	,652	,004
	Lower-bound	,206	,652	,004
Error (Produkt*Messzeitpunkt)	Sphericity Assumed			
	Greenhouse-Geisser			
	Huynh-Feldt			
	Lower-bound			

Tests of Within-Subjects Contrasts

Measure: MEASURE_1

Source	Produkt	Messzeitpunkt	Type III Sum of Squares	df	Mean Square
Produkt	Linear		1,562	1	1,562
Error(Produkt)	Linear		30,398	58	,524
Messzeitpunkt		Linear	1,373	1	1,373
Error(Messzeitpunkt)		Linear	19,347	58	,334
Produkt * Messzeitpunkt	Linear	Linear	,043	1	,043
Error (Produkt*Messzeitpunkt)	Linear	Linear	12,237	58	,211

Tests of Within-Subjects Contrasts

Measure: MEASURE_1

Source	Produkt	Messzeitpunkt	F	Sig.	Partial Eta Squared
Produkt	Linear		2,980	,090	,049
Error(Produkt)	Linear				
Messzeitpunkt		Linear	4,116	,047	,066
Error(Messzeitpunkt)		Linear			
Produkt * Messzeitpunkt	Linear	Linear	,206	,652	,004
Error (Produkt*Messzeitpunkt)	Linear	Linear			

Tests of Between-Subjects Effects

Measure: MEASURE_1

Transformed Variable: Average

Source	Type III Sum of Squares	df	Mean Square	F	Sig.	Partial Eta Squared
Intercept	971,396	1	971,396	729,389	,000	,926
Error	77,244	58	1,332			

Estimated Marginal Means

Produkt

Measure: MEASURE_1

Produkt	Mean	Std. Error	95% Confidence Interval	
			Lower Bound	Upper Bound
1	2,110	,086	1,938	2,282
2	1,947	,091	1,765	2,130

```
 GLM Befindlichkeit1.Diamant.06.12.12 Befindlichkeit1.Diamant.17.01.13
Befindlichkeit1.Guarani.06.12.12 Befindlichkeit1.Guarani.17.01.13
  /WSFACTOR=Produkt 2 Polynomial Messzeitpunkt 2 Polynomial
  /METHOD=SSTYPE(3)
  /EMMEANS=TABLES(Produkt)
  /PRINT=DESCRIPTIVE ETASQ
  /CRITERIA=ALPHA(.05)
  /WSDESIGN=Produkt Messzeitpunkt Produkt*Messzeitpunkt.
```

General Linear Model

Notes

Output Created		29-OCT-2013 12:22:42
Comments		
Input	Data	C:\Documents and Settings\Dennis Boywitt\My Documents\My Dropbox\Freiberufliche Tätigkeit\Forschungsring\Arbeitsordner Daten\Befindlichkeiten_Gruppe2_restructured.sav
	Active Dataset	DataSet2
	Filter	<none>
	Weight	<none>
	Split File	<none>
	N of Rows in Working Data File	62
Missing Value Handling	Definition of Missing	User-defined missing values are treated as missing.
	Cases Used	Statistics are based on all cases with valid data for all variables in the model.
Syntax		GLM Befindlichkeit1.Diamant.06.12.12 Befindlichkeit1.Diamant.17.01.13 Befindlichkeit1.Guarani.06.12.12 Befindlichkeit1.Guarani.17.01.13 /WSFACTOR=Produkt 2 Polynomial Messzeitpunkt 2 Polynomial /METHOD=SSTYPE(3) /EMMEANS=TABLES(Produkt) /PRINT=DESCRIPTIVE ETASQ /CRITERIA=ALPHA(.05) /WSDESIGN=Produkt Messzeitpunkt Produkt*Messzeitpunkt.
Resources	Processor Time	00:00:00,03
	Elapsed Time	00:00:00,03

```
[DataSet2] C:\Documents and Settings\Dennis Boywitt\My Documents\My Dropbox\Freiberufliche Tätigkeit\Forschungsring\Arbeitsordner Daten\Befindlichkeiten_Gruppe2_restructured.sav
```

Within-Subjects Factors

Measure: MEASURE_1

Produkt	Messzeitpunkt	Dependent Variable
1	1	Befindlichkeit1 .Diamant. 06.12.12
	2	Befindlichkeit1 .Diamant. 17.01.13
2	1	Befindlichkeit1 .Guarani. 06.12.12
	2	Befindlichkeit1 .Guarani. 17.01.13

Descriptive Statistics

	Mean	Std. Deviation	N
Befindlichkeit1.Diamant. 06.12.12: Ich empfinde meinen Leib 1	2,12	,930	59
Befindlichkeit1.Diamant. 17.01.13: Ich empfinde meinen Leib 1	2,32	1,121	59
Befindlichkeit1.Guarani. 06.12.12: Ich empfinde meinen Leib 1	2,02	,956	59
Befindlichkeit1.Guarani. 17.01.13: Ich empfinde meinen Leib 1	2,10	1,012	59

Multivariate Tests[a]

Effect		Value	F	Hypothesis df	Error df
Produkt	Pillai's Trace	,044	2,671[b]	1,000	58,000
	Wilks' Lambda	,956	2,671[b]	1,000	58,000
	Hotelling's Trace	,046	2,671[b]	1,000	58,000
	Roy's Largest Root	,046	2,671[b]	1,000	58,000
Messzeitpunkt	Pillai's Trace	,033	1,999[b]	1,000	58,000
	Wilks' Lambda	,967	1,999[b]	1,000	58,000
	Hotelling's Trace	,034	1,999[b]	1,000	58,000
	Roy's Largest Root	,034	1,999[b]	1,000	58,000
Produkt * Messzeitpunkt	Pillai's Trace	,007	,422[b]	1,000	58,000
	Wilks' Lambda	,993	,422[b]	1,000	58,000
	Hotelling's Trace	,007	,422[b]	1,000	58,000
	Roy's Largest Root	,007	,422[b]	1,000	58,000

Multivariate Tests[a]

Effect		Sig.	Partial Eta Squared
Produkt	Pillai's Trace	,108	,044
	Wilks' Lambda	,108	,044
	Hotelling's Trace	,108	,044
	Roy's Largest Root	,108	,044
Messzeitpunkt	Pillai's Trace	,163	,033
	Wilks' Lambda	,163	,033
	Hotelling's Trace	,163	,033
	Roy's Largest Root	,163	,033
Produkt * Messzeitpunkt	Pillai's Trace	,519	,007
	Wilks' Lambda	,519	,007
	Hotelling's Trace	,519	,007
	Roy's Largest Root	,519	,007

a. Design: Intercept
Within Subjects Design: Produkt + Messzeitpunkt + Produkt * Messzeitpunkt

b. Exact statistic

Mauchly's Test of Sphericity[a]

Measure: MEASURE_1

Within Subjects Effect	Mauchly's W	Approx. Chi-Square	df	Sig.	Epsilon[b] Greenhouse-Geisser	Epsilon[b] Huynh-Feldt	Epsilon[b] Lower-bound
Produkt	1,000	,000	0	.	1,000	1,000	1,000
Messzeitpunkt	1,000	,000	0	.	1,000	1,000	1,000
Produkt * Messzeitpunkt	1,000	,000	0	.	1,000	1,000	1,000

Tests the null hypothesis that the error covariance matrix of the orthonormalized transformed dependent variables is proportional to an identity matrix.

a. Design: Intercept
Within Subjects Design: Produkt + Messzeitpunkt + Produkt * Messzeitpunkt

b. May be used to adjust the degrees of freedom for the averaged tests of significance. Corrected tests are displayed in the Tests of Within-Subjects Effects table.

Tests of Within-Subjects Effects

Measure: MEASURE_1

Source		Type III Sum of Squares	df	Mean Square
Produkt	Sphericity Assumed	1,530	1	1,530
	Greenhouse-Geisser	1,530	1,000	1,530
	Huynh-Feldt	1,530	1,000	1,530
	Lower-bound	1,530	1,000	1,530
Error(Produkt)	Sphericity Assumed	33,220	58	,573
	Greenhouse-Geisser	33,220	58,000	,573
	Huynh-Feldt	33,220	58,000	,573
	Lower-bound	33,220	58,000	,573
Messzeitpunkt	Sphericity Assumed	1,225	1	1,225
	Greenhouse-Geisser	1,225	1,000	1,225
	Huynh-Feldt	1,225	1,000	1,225
	Lower-bound	1,225	1,000	1,225
Error(Messzeitpunkt)	Sphericity Assumed	35,525	58	,613
	Greenhouse-Geisser	35,525	58,000	,613
	Huynh-Feldt	35,525	58,000	,613
	Lower-bound	35,525	58,000	,613
Produkt * Messzeitpunkt	Sphericity Assumed	,208	1	,208
	Greenhouse-Geisser	,208	1,000	,208
	Huynh-Feldt	,208	1,000	,208
	Lower-bound	,208	1,000	,208
Error (Produkt*Messzeitpunkt)	Sphericity Assumed	28,542	58	,492
	Greenhouse-Geisser	28,542	58,000	,492
	Huynh-Feldt	28,542	58,000	,492
	Lower-bound	28,542	58,000	,492

Tests of Within-Subjects Effects

Measure: MEASURE_1

Source		F	Sig.	Partial Eta Squared
Produkt	Sphericity Assumed	2,671	,108	,044
	Greenhouse-Geisser	2,671	,108	,044
	Huynh-Feldt	2,671	,108	,044
	Lower-bound	2,671	,108	,044
Error(Produkt)	Sphericity Assumed			
	Greenhouse-Geisser			
	Huynh-Feldt			
	Lower-bound			
Messzeitpunkt	Sphericity Assumed	1,999	,163	,033
	Greenhouse-Geisser	1,999	,163	,033
	Huynh-Feldt	1,999	,163	,033
	Lower-bound	1,999	,163	,033
Error(Messzeitpunkt)	Sphericity Assumed			
	Greenhouse-Geisser			
	Huynh-Feldt			
	Lower-bound			
Produkt * Messzeitpunkt	Sphericity Assumed	,422	,519	,007
	Greenhouse-Geisser	,422	,519	,007
	Huynh-Feldt	,422	,519	,007
	Lower-bound	,422	,519	,007
Error (Produkt*Messzeitpunkt)	Sphericity Assumed			
	Greenhouse-Geisser			
	Huynh-Feldt			
	Lower-bound			

Tests of Within-Subjects Contrasts

Measure: MEASURE_1

Source	Produkt	Messzeitpunkt	Type III Sum of Squares	df	Mean Square
Produkt	Linear		1,530	1	1,530
Error(Produkt)	Linear		33,220	58	,573
Messzeitpunkt		Linear	1,225	1	1,225
Error(Messzeitpunkt)		Linear	35,525	58	,613
Produkt * Messzeitpunkt	Linear	Linear	,208	1	,208
Error (Produkt*Messzeitpunkt)	Linear	Linear	28,542	58	,492

Tests of Within-Subjects Contrasts

Measure: MEASURE_1

Source	Produkt	Messzeitpunkt	F	Sig.	Partial Eta Squared
Produkt	Linear		2,671	,108	,044
Error(Produkt)	Linear				
Messzeitpunkt		Linear	1,999	,163	,033
Error(Messzeitpunkt)		Linear			
Produkt * Messzeitpunkt	Linear	Linear	,422	,519	,007
Error (Produkt*Messzeitpunkt)	Linear	Linear			

Tests of Between-Subjects Effects

Measure: MEASURE_1

Transformed Variable: Average

Source	Type III Sum of Squares	df	Mean Square	F	Sig.	Partial Eta Squared
Intercept	1080,614	1	1080,614	453,725	,000	,887
Error	138,136	58	2,382			

Estimated Marginal Means

Produkt

Measure: MEASURE_1

Produkt	Mean	Std. Error	95% Confidence Interval	
			Lower Bound	Upper Bound
1	2,220	,114	1,992	2,449
2	2,059	,110	1,840	2,279

```
 GLM Befindlichkeit12.Diamant.06.12.12 Befindlichkeit12.Diamant.17.01.13
Befindlichkeit12.Guarani.06.12.12 Befindlichkeit12.Guarani.17.01.13
  /WSFACTOR=Produkt 2 Polynomial Messzeitpunkt 2 Polynomial
  /METHOD=SSTYPE(3)
  /EMMEANS=TABLES(Produkt)
  /PRINT=DESCRIPTIVE ETASQ
  /CRITERIA=ALPHA(.05)
  /WSDESIGN=Produkt Messzeitpunkt Produkt*Messzeitpunkt.
```

General Linear Model

Notes

Output Created		29-OCT-2013 12:24:56
Comments		
Input	Data	C:\Documents and Settings\Dennis Boywitt\My Documents\My Dropbox\Freiberufliche Tätigkeit\Forschungsring\Arbeitsordner Daten\Befindlichkeiten_Gruppe2_restructured.sav
	Active Dataset	DataSet2
	Filter	<none>
	Weight	<none>
	Split File	<none>
	N of Rows in Working Data File	62
Missing Value Handling	Definition of Missing	User-defined missing values are treated as missing.
	Cases Used	Statistics are based on all cases with valid data for all variables in the model.
Syntax		GLM Befindlichkeit12.Diamant.06.12.12 Befindlichkeit12.Diamant.17.01.13 Befindlichkeit12.Guarani.06.12.12 Befindlichkeit12.Guarani.17.01.13<br>/WSFACTOR=Produkt 2 Polynomial Messzeitpunkt 2 Polynomial<br>/METHOD=SSTYPE(3)<br>/EMMEANS=TABLES(Produkt)<br>/PRINT=DESCRIPTIVE ETASQ<br>/CRITERIA=ALPHA(.05)<br>/WSDESIGN=Produkt Messzeitpunkt Produkt*Messzeitpunkt.
Resources	Processor Time	00:00:00,03
	Elapsed Time	00:00:00,03

```
[DataSet2] C:\Documents and Settings\Dennis Boywitt\My Documents\My Dropbo
x\Freiberufliche Tätigkeit\Forschungsring\Arbeitsordner Daten\Befindlichke
iten_Gruppe2_restructured.sav
```

Within-Subjects Factors

Measure: MEASURE_1

Produkt	Messzeitpunkt	Dependent Variable
1	1	Befindlichkeit12.Diamant.06.12.12
	2	Befindlichkeit12.Diamant.17.01.13
2	1	Befindlichkeit12.Guarani.06.12.12
	2	Befindlichkeit12.Guarani.17.01.13

Descriptive Statistics

	Mean	Std. Deviation	N
Befindlichkeit12.Diamant.06.12.12: Ich empfinde die Wirkung	2,48	,960	58
Befindlichkeit12.Diamant.17.01.13: Ich empfinde die Wirkung	2,67	1,145	58
Befindlichkeit12.Guarani.06.12.12: Ich empfinde die Wirkung	2,66	1,052	58
Befindlichkeit12.Guarani.17.01.13: Ich empfinde die Wirkung	2,55	1,142	58

Multivariate Tests[a]

Effect		Value	F	Hypothesis df	Error df
Produkt	Pillai's Trace	,001	,047[b]	1,000	57,000
	Wilks' Lambda	,999	,047[b]	1,000	57,000
	Hotelling's Trace	,001	,047[b]	1,000	57,000
	Roy's Largest Root	,001	,047[b]	1,000	57,000
Messzeitpunkt	Pillai's Trace	,002	,094[b]	1,000	57,000
	Wilks' Lambda	,998	,094[b]	1,000	57,000
	Hotelling's Trace	,002	,094[b]	1,000	57,000
	Roy's Largest Root	,002	,094[b]	1,000	57,000
Produkt * Messzeitpunkt	Pillai's Trace	,030	1,753[b]	1,000	57,000
	Wilks' Lambda	,970	1,753[b]	1,000	57,000
	Hotelling's Trace	,031	1,753[b]	1,000	57,000
	Roy's Largest Root	,031	1,753[b]	1,000	57,000

Multivariate Tests[a]

Effect		Sig.	Partial Eta Squared
Produkt	Pillai's Trace	,829	,001
	Wilks' Lambda	,829	,001
	Hotelling's Trace	,829	,001
	Roy's Largest Root	,829	,001
Messzeitpunkt	Pillai's Trace	,761	,002
	Wilks' Lambda	,761	,002
	Hotelling's Trace	,761	,002
	Roy's Largest Root	,761	,002
Produkt * Messzeitpunkt	Pillai's Trace	,191	,030
	Wilks' Lambda	,191	,030
	Hotelling's Trace	,191	,030
	Roy's Largest Root	,191	,030

a. Design: Intercept
Within Subjects Design: Produkt + Messzeitpunkt + Produkt * Messzeitpunkt

b. Exact statistic

Mauchly's Test of Sphericity[a]

Measure: MEASURE_1

Within Subjects Effect	Mauchly's W	Approx. Chi-Square	df	Sig.	Epsilon[b] Greenhouse-Geisser
Produkt	1,000	,000	0	.	1,000
Messzeitpunkt	1,000	,000	0	.	1,000
Produkt * Messzeitpunkt	1,000	,000	0	.	1,000

Mauchly's Test of Sphericity[a]

Measure: MEASURE_1

Within Subjects Effect	Epsilon[b]	
	Huynh-Feldt	Lower-bound
Produkt	1,000	1,000
Messzeitpunkt	1,000	1,000
Produkt * Messzeitpunkt	1,000	1,000

Tests the null hypothesis that the error covariance matrix of the orthonormalized transformed dependent variables is proportional to an identity matrix.

a. Design: Intercept
Within Subjects Design: Produkt + Messzeitpunkt + Produkt * Messzeitpunkt

b. May be used to adjust the degrees of freedom for the averaged tests of significance. Corrected tests are displayed in the Tests of Within-Subjects Effects table.

Tests of Within-Subjects Effects

Measure: MEASURE_1

Source		Type III Sum of Squares	df	Mean Square
Produkt	Sphericity Assumed	,039	1	,039
	Greenhouse-Geisser	,039	1,000	,039
	Huynh-Feldt	,039	1,000	,039
	Lower-bound	,039	1,000	,039
Error(Produkt)	Sphericity Assumed	46,711	57	,819
	Greenhouse-Geisser	46,711	57,000	,819
	Huynh-Feldt	46,711	57,000	,819
	Lower-bound	46,711	57,000	,819
Messzeitpunkt	Sphericity Assumed	,108	1	,108
	Greenhouse-Geisser	,108	1,000	,108
	Huynh-Feldt	,108	1,000	,108
	Lower-bound	,108	1,000	,108
Error(Messzeitpunkt)	Sphericity Assumed	65,642	57	1,152
	Greenhouse-Geisser	65,642	57,000	1,152
	Huynh-Feldt	65,642	57,000	1,152
	Lower-bound	65,642	57,000	1,152
Produkt * Messzeitpunkt	Sphericity Assumed	1,246	1	1,246
	Greenhouse-Geisser	1,246	1,000	1,246
	Huynh-Feldt	1,246	1,000	1,246
	Lower-bound	1,246	1,000	1,246
Error (Produkt*Messzeitpunkt)	Sphericity Assumed	40,504	57	,711
	Greenhouse-Geisser	40,504	57,000	,711
	Huynh-Feldt	40,504	57,000	,711
	Lower-bound	40,504	57,000	,711

Tests of Within-Subjects Effects

Measure: MEASURE_1

Source		F	Sig.	Partial Eta Squared
Produkt	Sphericity Assumed	,047	,829	,001
	Greenhouse-Geisser	,047	,829	,001
	Huynh-Feldt	,047	,829	,001
	Lower-bound	,047	,829	,001
Error(Produkt)	Sphericity Assumed			
	Greenhouse-Geisser			
	Huynh-Feldt			
	Lower-bound			
Messzeitpunkt	Sphericity Assumed	,094	,761	,002
	Greenhouse-Geisser	,094	,761	,002
	Huynh-Feldt	,094	,761	,002
	Lower-bound	,094	,761	,002
Error(Messzeitpunkt)	Sphericity Assumed			
	Greenhouse-Geisser			
	Huynh-Feldt			
	Lower-bound			
Produkt * Messzeitpunkt	Sphericity Assumed	1,753	,191	,030
	Greenhouse-Geisser	1,753	,191	,030
	Huynh-Feldt	1,753	,191	,030
	Lower-bound	1,753	,191	,030
Error (Produkt*Messzeitpunkt)	Sphericity Assumed			
	Greenhouse-Geisser			
	Huynh-Feldt			
	Lower-bound			

Tests of Within-Subjects Contrasts

Measure: MEASURE_1

Source	Produkt	Messzeitpunkt	Type III Sum of Squares	df	Mean Square
Produkt	Linear		,039	1	,039
Error(Produkt)	Linear		46,711	57	,819
Messzeitpunkt		Linear	,108	1	,108
Error(Messzeitpunkt)		Linear	65,642	57	1,152
Produkt * Messzeitpunkt	Linear	Linear	1,246	1	1,246
Error (Produkt*Messzeitpunkt)	Linear	Linear	40,504	57	,711

Tests of Within-Subjects Contrasts

Measure: MEASURE_1

Source	Produkt	Messzeitpunkt	F	Sig.	Partial Eta Squared
Produkt	Linear		,047	,829	,001
Error(Produkt)	Linear				
Messzeitpunkt		Linear	,094	,761	,002
Error(Messzeitpunkt)		Linear			
Produkt * Messzeitpunkt	Linear	Linear	1,753	,191	,030
Error (Produkt*Messzeitpunkt)	Linear	Linear			

Tests of Between-Subjects Effects

Measure: MEASURE_1

Transformed Variable: Average

Source	Type III Sum of Squares	df	Mean Square	F	Sig.	Partial Eta Squared
Intercept	1556,901	1	1556,901	793,420	,000	,933
Error	111,849	57	1,962			

Estimated Marginal Means

Produkt

Measure: MEASURE_1

Produkt	Mean	Std. Error	95% Confidence Interval	
			Lower Bound	Upper Bound
1	2,578	,115	2,348	2,807
2	2,603	,104	2,395	2,812

```
 DATASET ACTIVATE DataSet2.
SAVE OUTFILE='C:\Documents and Settings\Dennis Boywitt\My Documents\My Dropbox\Freiberuflich
    'Tätigkeit\Forschungsring\Arbeitsordner Daten\Befindlichkeiten_Gruppe2_restructured.sav'
 /COMPRESSED.
```
