# Supplementary material for: Development and Application of a Test for Food-Induced Emotions
Source: PLoS One. 2016 Nov 18;11(11):e0165991. doi: 10.1371/journal.pone.0165991 (PMC5115674; doi:10.1371/journal.pone.0165991)
Supplement: S9 File — (PDF) [file pone.0165991.s012.pdf]

```
GLM Sensorik1.AdelholzerGlas27.11.12 Sensorik1AdelholzerGlas09.01.13
    Sensorik1Adelholzerplastik27.11.12 Sensorik1AdelholzerPlastik09.01.13
  /WSFACTOR=Produkttyp 2 Polynomial Meeszeitpunkt 2 Polynomial
  /METHOD=SSTYPE(3)
  /EMMEANS=TABLES(Produkttyp)
  /PRINT=DESCRIPTIVE ETASQ
  /CRITERIA=ALPHA(.05)
  /WSDESIGN=Produkttyp Meeszeitpunkt Produkttyp*Meeszeitpunkt.
```

General Linear Model

Notes

Output Created		11-NOV-2013 14:57:10
Comments		
Input	Data	C:\Documents and Settings\Dennis Boywitt\My Documents\My Dropbox\Freiberufliche Tätigkeit\Forschungsring\Daten\Sensorik_Gruppe_1_restructured.sav
	Active Dataset	DataSet2
	Filter	<none>
	Weight	<none>
	Split File	<none>
	N of Rows in Working Data File	65
Missing Value Handling	Definition of Missing	User-defined missing values are treated as missing.
	Cases Used	Statistics are based on all cases with valid data for all variables in the model.

Notes

Syntax		GLM Sensorik1.<br>AdelholzerGlas27.11.12<br>Sensorik1AdelholzerGlas09.01.13<br><br>Sensorik1Adelholzerplastik27.11.12<br>Sensorik1AdelholzerPlastik09.01.13<br>/WSFACTOR=Produkttyp 2 Polynomial Meeszeitpunkt 2 Polynomial<br>/METHOD=SSTYPE(3)<br>/EMMEANS=TABLES(Produkttyp)<br>/PRINT=DESCRIPTIVE ETASQ<br>/CRITERIA=ALPHA(.05)<br>/WSDESIGN=Produkttyp Meeszeitpunkt Produkttyp*Meeszeitpunkt.
Resources	Processor Time	00:00:00,02
	Elapsed Time	00:00:00,03

```
[DataSet2] C:\Documents and Settings\Dennis Boywitt\My Documents\My Dropbox\Freiberufliche Tätigkeit\Forschungsring\Daten\Sensorik_Gruppe_1_restructured.sav
```

Within-Subjects Factors

Measure: MEASURE_1

Produkttyp	Meeszeitpunkt	Dependent Variable
1	1	Sensorik1.AdelholzerGlas27.11.12
	2	Sensorik1AdelholzerGlas09.01.13
2	1	Sensorik1Adelholzerplastik27.11.12
	2	Sensorik1AdelholzerPlastik09.01.13

Descriptive Statistics

	Mean	Std. Deviation	N
Sensorik1. AdelholzerGlas27.11.12	5,63	1,248	63
Sensorik1AdelholzerGlas09.01.13	5,25	1,150	63
Sensorik1Adelholzerplastik27.11.12	5,54	1,229	63
Sensorik1AdelholzerPlastik09.01.13	5,46	1,105	63

Multivariate Tests[a]

Effect		Value	F	Hypothesis df	Error df
Produkttyp	Pillai's Trace	,006	,388[b]	1,000	62,000
	Wilks' Lambda	,994	,388[b]	1,000	62,000
	Hotelling's Trace	,006	,388[b]	1,000	62,000
	Roy's Largest Root	,006	,388[b]	1,000	62,000
Meeszeitpunkt	Pillai's Trace	,118	8,306[b]	1,000	62,000
	Wilks' Lambda	,882	8,306[b]	1,000	62,000
	Hotelling's Trace	,134	8,306[b]	1,000	62,000
	Roy's Largest Root	,134	8,306[b]	1,000	62,000
Produkttyp * Meeszeitpunkt	Pillai's Trace	,094	6,428[b]	1,000	62,000
	Wilks' Lambda	,906	6,428[b]	1,000	62,000
	Hotelling's Trace	,104	6,428[b]	1,000	62,000
	Roy's Largest Root	,104	6,428[b]	1,000	62,000

Multivariate Tests[a]

Effect		Sig.	Partial Eta Squared
Produkttyp	Pillai's Trace	,536	,006
	Wilks' Lambda	,536	,006
	Hotelling's Trace	,536	,006
	Roy's Largest Root	,536	,006
Meeszeitpunkt	Pillai's Trace	,005	,118
	Wilks' Lambda	,005	,118
	Hotelling's Trace	,005	,118
	Roy's Largest Root	,005	,118
Produkttyp * Meeszeitpunkt	Pillai's Trace	,014	,094
	Wilks' Lambda	,014	,094
	Hotelling's Trace	,014	,094
	Roy's Largest Root	,014	,094

a. Design: Intercept
Within Subjects Design: Produkttyp + Meeszeitpunkt + Produkttyp * Meeszeitpunkt

b. Exact statistic

Mauchly's Test of Sphericity[a]

Measure: MEASURE_1

Within Subjects Effect	Mauchly's W	Approx. Chi-Square	df	Sig.	Epsilon[b]
					Greenhouse-Geisser
Produkttyp	1,000	,000	0	.	1,000
Meeszeitpunkt	1,000	,000	0	.	1,000
Produkttyp * Meeszeitpunkt	1,000	,000	0	.	1,000

Mauchly's Test of Sphericity[a]

Measure: MEASURE_1

Within Subjects Effect	Epsilon[b]	
	Huynh-Feldt	Lower-bound
Produkttyp	1,000	1,000
Meeszeitpunkt	1,000	1,000
Produkttyp * Meeszeitpunkt	1,000	1,000

Tests the null hypothesis that the error covariance matrix of the orthonormalized transformed dependent variables is proportional to an identity matrix.

a. Design: Intercept
Within Subjects Design: Produkttyp + Meeszeitpunkt + Produkttyp * Meeszeitpunkt

b. May be used to adjust the degrees of freedom for the averaged tests of significance. Corrected tests are displayed in the Tests of Within-Subjects Effects table.

Tests of Within-Subjects Effects

Measure: MEASURE_1

Source		Type III Sum of Squares	df	Mean Square
Produkttyp	Sphericity Assumed	,194	1	,194
	Greenhouse-Geisser	,194	1,000	,194
	Huynh-Feldt	,194	1,000	,194
	Lower-bound	,194	1,000	,194
Error(Produkttyp)	Sphericity Assumed	31,056	62	,501
	Greenhouse-Geisser	31,056	62,000	,501
	Huynh-Feldt	31,056	62,000	,501
	Lower-bound	31,056	62,000	,501
Meeszeitpunkt	Sphericity Assumed	3,337	1	3,337
	Greenhouse-Geisser	3,337	1,000	3,337
	Huynh-Feldt	3,337	1,000	3,337
	Lower-bound	3,337	1,000	3,337
Error(Meeszeitpunkt)	Sphericity Assumed	24,913	62	,402
	Greenhouse-Geisser	24,913	62,000	,402
	Huynh-Feldt	24,913	62,000	,402
	Lower-bound	24,913	62,000	,402
Produkttyp * Meeszeitpunkt	Sphericity Assumed	1,433	1	1,433
	Greenhouse-Geisser	1,433	1,000	1,433
	Huynh-Feldt	1,433	1,000	1,433
	Lower-bound	1,433	1,000	1,433
Error (Produkttyp*Meeszeitpunkt)	Sphericity Assumed	13,817	62	,223
	Greenhouse-Geisser	13,817	62,000	,223
	Huynh-Feldt	13,817	62,000	,223
	Lower-bound	13,817	62,000	,223

Tests of Within-Subjects Effects

Measure: MEASURE_1

Source		F	Sig.	Partial Eta Squared
Produkttyp	Sphericity Assumed	,388	,536	,006
	Greenhouse-Geisser	,388	,536	,006
	Huynh-Feldt	,388	,536	,006
	Lower-bound	,388	,536	,006
Error(Produkttyp)	Sphericity Assumed			
	Greenhouse-Geisser			
	Huynh-Feldt			
	Lower-bound			
Meeszeitpunkt	Sphericity Assumed	8,306	,005	,118
	Greenhouse-Geisser	8,306	,005	,118
	Huynh-Feldt	8,306	,005	,118
	Lower-bound	8,306	,005	,118
Error(Meeszeitpunkt)	Sphericity Assumed			
	Greenhouse-Geisser			
	Huynh-Feldt			
	Lower-bound			
Produkttyp * Meeszeitpunkt	Sphericity Assumed	6,428	,014	,094
	Greenhouse-Geisser	6,428	,014	,094
	Huynh-Feldt	6,428	,014	,094
	Lower-bound	6,428	,014	,094
Error (Produkttyp*Meeszeitpunkt)	Sphericity Assumed			
	Greenhouse-Geisser			
	Huynh-Feldt			
	Lower-bound			

Tests of Within-Subjects Contrasts

Measure: MEASURE_1

Source	Produkttyp	Meeszeitpunkt	Type III Sum of Squares	df	Mean Square
Produkttyp	Linear		,194	1	,194
Error(Produkttyp)	Linear		31,056	62	,501
Meeszeitpunkt		Linear	3,337	1	3,337
Error(Meeszeitpunkt)		Linear	24,913	62	,402
Produkttyp * Meeszeitpunkt	Linear	Linear	1,433	1	1,433
Error (Produkttyp*Meeszeitpunkt)	Linear	Linear	13,817	62	,223

Tests of Within-Subjects Contrasts

Measure: MEASURE_1

Source	Produkttyp	Meeszeitpunkt	F	Sig.	Partial Eta Squared
Produkttyp	Linear		,388	,536	,006
Error(Produkttyp)	Linear				
Meeszeitpunkt		Linear	8,306	,005	,118
Error(Meeszeitpunkt)		Linear			
Produkttyp * Meeszeitpunkt	Linear	Linear	6,428	,014	,094
Error (Produkttyp*Meeszeitpunkt)	Linear	Linear			

Tests of Between-Subjects Effects

Measure: MEASURE_1

Transformed Variable: Average

Source	Type III Sum of Squares	df	Mean Square	F	Sig.	Partial Eta Squared
Intercept	7546,194	1	7546,194	1682,628	,000	,964
Error	278,056	62	4,485			

Estimated Marginal Means

Produkttyp

Measure: MEASURE_1

Produkttyp	Mean	Std. Error	95% Confidence Interval	
			Lower Bound	Upper Bound
1	5,444	,142	5,160	5,729
2	5,500	,139	5,222	5,778

```
GLM Sensorik2AdelholzerGlas27.11.12 Sensorik2AdelholzerGlas09.01.13
   Sensorik2Adelholzerplastik27.11.12 Sensorik2AdelholzerPlastik09.01.13
  /WSFACTOR=Produkttyp 2 Polynomial Meeszeitpunkt 2 Polynomial
  /METHOD=SSTYPE(3)
  /EMMEANS=TABLES(Produkttyp)
  /PRINT=DESCRIPTIVE ETASQ
  /CRITERIA=ALPHA(.05)
  /WSDESIGN=Produkttyp Meeszeitpunkt Produkttyp*Meeszeitpunkt.
```

General Linear Model

Notes

Output Created		11-NOV-2013 15:01:27
Comments		
Input	Data	C:\Documents and Settings\Dennis Boywitt\My Documents\My Dropbox\Freiberufliche Tätigkeit\Forschungsring\Daten\Sensorik_Gruppe_1_restructured.sav
	Active Dataset	DataSet2
	Filter	<none>
	Weight	<none>
	Split File	<none>
	N of Rows in Working Data File	65
Missing Value Handling	Definition of Missing	User-defined missing values are treated as missing.
	Cases Used	Statistics are based on all cases with valid data for all variables in the model.
Syntax		GLM Sensorik2AdelholzerGlas27.11.12 Sensorik2AdelholzerGlas09.01.13<br><br>Sensorik2Adelholzerplastik27.11.12 Sensorik2AdelholzerPlastik09.01.13<br>/WSFACTOR=Produkttyp 2 Polynomial Meeszeitpunkt 2 Polynomial<br>/METHOD=SSTYPE(3)<br>/EMMEANS=TABLES(Produkttyp)<br>/PRINT=DESCRIPTIVE ETASQ<br>/CRITERIA=ALPHA(.05)<br>/WSDESIGN=Produkttyp Meeszeitpunkt Produkttyp*Meeszeitpunkt.
Resources	Processor Time	00:00:00,02
	Elapsed Time	00:00:00,02

```
[DataSet2] C:\Documents and Settings\Dennis Boywitt\My Documents\My Dropbox\Freiberufliche Tätigkeit\Forschungsring\Daten\Sensorik_Gruppe_1_restructured.sav
```

Within-Subjects Factors

Measure: MEASURE_1

Produkttyp	Meeszeitpunkt	Dependent Variable
1	1	Sensorik2AdelholzerGlas27.11.12
	2	Sensorik2AdelholzerGlas09.01.13
2	1	Sensorik2Adelholzerplastik27.11.12
	2	Sensorik2AdelholzerPlastik09.01.13

Descriptive Statistics

	Mean	Std. Deviation	N
Sensorik2AdelholzerGlas27.11.12	5,39	,981	62
Sensorik2AdelholzerGlas09.01.13	5,21	,926	62
Sensorik2Adelholzerplastik27.11.12	5,37	1,028	62
Sensorik2AdelholzerPlastik09.01.13	5,37	,945	62

Multivariate Tests[a]

Effect		Value	F	Hypothesis df	Error df
Produkttyp	Pillai's Trace	,021	1,292[b]	1,000	61,000
	Wilks' Lambda	,979	1,292[b]	1,000	61,000
	Hotelling's Trace	,021	1,292[b]	1,000	61,000
	Roy's Largest Root	,021	1,292[b]	1,000	61,000
Meeszeitpunkt	Pillai's Trace	,013	,821[b]	1,000	61,000
	Wilks' Lambda	,987	,821[b]	1,000	61,000
	Hotelling's Trace	,013	,821[b]	1,000	61,000
	Roy's Largest Root	,013	,821[b]	1,000	61,000
Produkttyp * Meeszeitpunkt	Pillai's Trace	,026	1,630[b]	1,000	61,000
	Wilks' Lambda	,974	1,630[b]	1,000	61,000
	Hotelling's Trace	,027	1,630[b]	1,000	61,000
	Roy's Largest Root	,027	1,630[b]	1,000	61,000

Multivariate Tests[a]

Effect		Sig.	Partial Eta Squared
Produkttyp	Pillai's Trace	,260	,021
	Wilks' Lambda	,260	,021
	Hotelling's Trace	,260	,021
	Roy's Largest Root	,260	,021
Meeszeitpunkt	Pillai's Trace	,369	,013
	Wilks' Lambda	,369	,013
	Hotelling's Trace	,369	,013
	Roy's Largest Root	,369	,013
Produkttyp * Meeszeitpunkt	Pillai's Trace	,207	,026
	Wilks' Lambda	,207	,026
	Hotelling's Trace	,207	,026
	Roy's Largest Root	,207	,026

a. Design: Intercept
Within Subjects Design: Produkttyp + Meeszeitpunkt + Produkttyp * Meeszeitpunkt

b. Exact statistic

Mauchly's Test of Sphericity[a]

Measure: MEASURE_1

Within Subjects Effect	Mauchly's W	Approx. Chi-Square	df	Sig.	Epsilon[b]
					Greenhouse-Geisser
Produkttyp	1,000	,000	0	.	1,000
Meeszeitpunkt	1,000	,000	0	.	1,000
Produkttyp * Meeszeitpunkt	1,000	,000	0	.	1,000

Mauchly's Test of Sphericity[a]

Measure: MEASURE_1

Within Subjects Effect	Epsilon[b]	
	Huynh-Feldt	Lower-bound
Produkttyp	1,000	1,000
Meeszeitpunkt	1,000	1,000
Produkttyp * Meeszeitpunkt	1,000	1,000

Tests the null hypothesis that the error covariance matrix of the orthonormalized transformed dependent variables is proportional to an identity matrix.

a. Design: Intercept
Within Subjects Design: Produkttyp + Meeszeitpunkt + Produkttyp * Meeszeitpunkt

b. May be used to adjust the degrees of freedom for the averaged tests of significance. Corrected tests are displayed in the Tests of Within-Subjects Effects table.

Tests of Within-Subjects Effects

Measure: MEASURE_1

Source		Type III Sum of Squares	df	Mean Square
Produkttyp	Sphericity Assumed	,327	1	,327
	Greenhouse-Geisser	,327	1,000	,327
	Huynh-Feldt	,327	1,000	,327
	Lower-bound	,327	1,000	,327
Error(Produkttyp)	Sphericity Assumed	15,423	61	,253
	Greenhouse-Geisser	15,423	61,000	,253
	Huynh-Feldt	15,423	61,000	,253
	Lower-bound	15,423	61,000	,253
Meeszeitpunkt	Sphericity Assumed	,488	1	,488
	Greenhouse-Geisser	,488	1,000	,488
	Huynh-Feldt	,488	1,000	,488
	Lower-bound	,488	1,000	,488
Error(Meeszeitpunkt)	Sphericity Assumed	36,262	61	,594
	Greenhouse-Geisser	36,262	61,000	,594
	Huynh-Feldt	36,262	61,000	,594
	Lower-bound	36,262	61,000	,594
Produkttyp * Meeszeitpunkt	Sphericity Assumed	,488	1	,488
	Greenhouse-Geisser	,488	1,000	,488
	Huynh-Feldt	,488	1,000	,488
	Lower-bound	,488	1,000	,488
Error (Produkttyp*Meeszeitpunkt)	Sphericity Assumed	18,262	61	,299
	Greenhouse-Geisser	18,262	61,000	,299
	Huynh-Feldt	18,262	61,000	,299
	Lower-bound	18,262	61,000	,299

Tests of Within-Subjects Effects

Measure: MEASURE_1

Source		F	Sig.	Partial Eta Squared
Produkttyp	Sphericity Assumed	1,292	,260	,021
	Greenhouse-Geisser	1,292	,260	,021
	Huynh-Feldt	1,292	,260	,021
	Lower-bound	1,292	,260	,021
Error(Produkttyp)	Sphericity Assumed			
	Greenhouse-Geisser			
	Huynh-Feldt			
	Lower-bound			
Meeszeitpunkt	Sphericity Assumed	,821	,369	,013
	Greenhouse-Geisser	,821	,369	,013
	Huynh-Feldt	,821	,369	,013
	Lower-bound	,821	,369	,013
Error(Meeszeitpunkt)	Sphericity Assumed			
	Greenhouse-Geisser			
	Huynh-Feldt			
	Lower-bound			
Produkttyp * Meeszeitpunkt	Sphericity Assumed	1,630	,207	,026
	Greenhouse-Geisser	1,630	,207	,026
	Huynh-Feldt	1,630	,207	,026
	Lower-bound	1,630	,207	,026
Error (Produkttyp*Meeszeitpunkt)	Sphericity Assumed			
	Greenhouse-Geisser			
	Huynh-Feldt			
	Lower-bound			

Tests of Within-Subjects Contrasts

Measure: MEASURE_1

Source	Produkttyp	Meeszeitpunkt	Type III Sum of Squares	df	Mean Square
Produkttyp	Linear		,327	1	,327
Error(Produkttyp)	Linear		15,423	61	,253
Meeszeitpunkt		Linear	,488	1	,488
Error(Meeszeitpunkt)		Linear	36,262	61	,594
Produkttyp * Meeszeitpunkt	Linear	Linear	,488	1	,488
Error (Produkttyp*Meeszeitpunkt)	Linear	Linear	18,262	61	,299

Tests of Within-Subjects Contrasts

Measure: MEASURE_1

Source	Produkttyp	Meeszeitpunkt	F	Sig.	Partial Eta Squared
Produkttyp	Linear		1,292	,260	,021
Error(Produkttyp)	Linear				
Meeszeitpunkt		Linear	,821	,369	,013
Error(Meeszeitpunkt)		Linear			
Produkttyp * Meeszeitpunkt	Linear	Linear	1,630	,207	,026
Error (Produkttyp*Meeszeitpunkt)	Linear	Linear			

Tests of Between-Subjects Effects

Measure: MEASURE_1

Transformed Variable: Average

Source	Type III Sum of Squares	df	Mean Square	F	Sig.	Partial Eta Squared
Intercept	7057,778	1	7057,778	2691,253	,000	,978
Error	159,972	61	2,622			

Estimated Marginal Means

Produkttyp

Measure: MEASURE_1

Produkttyp	Mean	Std. Error	95% Confidence Interval	
			Lower Bound	Upper Bound
1	5,298	,106	5,087	5,510
2	5,371	,110	5,152	5,590

```
GLM Sensorik3AdelholzerGlas27.11.12 Sensorik3AdelholzerGlas09.01.13
   Sensorik3Adelholzerplastik27.11.12 Sensorik3AdelholzerPlastik09.01.13
 /WSFACTOR=Produkttyp 2 Polynomial Meeszeitpunkt 2 Polynomial
 /METHOD=SSTYPE(3)
 /EMMEANS=TABLES(Produkttyp)
 /PRINT=DESCRIPTIVE ETASQ
 /CRITERIA=ALPHA(.05)
 /WSDESIGN=Produkttyp Meeszeitpunkt Produkttyp*Meeszeitpunkt.
```

General Linear Model

Notes

Output Created		11-NOV-2013 15:02:24
Comments		
Input	Data	C:\Documents and Settings\Dennis Boywitt\My Documents\My Dropbox\Freiberufliche Tätigkeit\Forschungsring\Daten\Sensorik_Gruppe_1_restructured.sav
	Active Dataset	DataSet2
	Filter	<none>
	Weight	<none>
	Split File	<none>
	N of Rows in Working Data File	65
Missing Value Handling	Definition of Missing	User-defined missing values are treated as missing.
	Cases Used	Statistics are based on all cases with valid data for all variables in the model.
Syntax		GLM Sensorik3AdelholzerGlas27.11.12 Sensorik3AdelholzerGlas09.01.13<br><br>Sensorik3Adelholzerplastik27.11.12 Sensorik3AdelholzerPlastik09.01.13<br>/WSFACTOR=Produkttyp 2 Polynomial Meeszeitpunkt 2 Polynomial<br>/METHOD=SSTYPE(3)<br>/EMMEANS=TABLES(Produkttyp)<br>/PRINT=DESCRIPTIVE ETASQ<br>/CRITERIA=ALPHA(.05)<br>/WSDESIGN=Produkttyp Meeszeitpunkt Produkttyp*Meeszeitpunkt.
Resources	Processor Time	00:00:00,02
	Elapsed Time	00:00:00,05

```
[DataSet2] C:\Documents and Settings\Dennis Boywitt\My Documents\My Dropbo
x\Freiberufliche Tätigkeit\Forschungsring\Daten\Sensorik_Gruppe_1_restruct
ured.sav
```

Within-Subjects Factors

Measure: MEASURE_1

Produkttyp	Meeszeitpunkt	Dependent Variable
1	1	Sensorik3AdelholzerGlas27.11.12
	2	Sensorik3AdelholzerGlas09.01.13
2	1	Sensorik3Adelholzerplastik27.11.12
	2	Sensorik3AdelholzerPlastik09.01.13

Descriptive Statistics

	Mean	Std. Deviation	N
Sensorik3AdelholzerGlas27.11.12	5,08	,980	62
Sensorik3AdelholzerGlas09.01.13	5,00	1,086	62
Sensorik3Adelholzerplastik27.11.12	5,16	1,059	62
Sensorik3AdelholzerPlastik09.01.13	5,16	,995	62

Multivariate Tests[a]

Effect		Value	F	Hypothesis df	Error df
Produkttyp	Pillai's Trace	,014	,895[b]	1,000	61,000
	Wilks' Lambda	,986	,895[b]	1,000	61,000
	Hotelling's Trace	,015	,895[b]	1,000	61,000
	Roy's Largest Root	,015	,895[b]	1,000	61,000
Meeszeitpunkt	Pillai's Trace	,002	,132[b]	1,000	61,000
	Wilks' Lambda	,998	,132[b]	1,000	61,000
	Hotelling's Trace	,002	,132[b]	1,000	61,000
	Roy's Largest Root	,002	,132[b]	1,000	61,000
Produkttyp * Meeszeitpunkt	Pillai's Trace	,003	,183[b]	1,000	61,000
	Wilks' Lambda	,997	,183[b]	1,000	61,000
	Hotelling's Trace	,003	,183[b]	1,000	61,000
	Roy's Largest Root	,003	,183[b]	1,000	61,000

Multivariate Tests[a]

Effect		Sig.	Partial Eta Squared
Produkttyp	Pillai's Trace	,348	,014
	Wilks' Lambda	,348	,014
	Hotelling's Trace	,348	,014
	Roy's Largest Root	,348	,014
Meeszeitpunkt	Pillai's Trace	,718	,002
	Wilks' Lambda	,718	,002
	Hotelling's Trace	,718	,002
	Roy's Largest Root	,718	,002
Produkttyp * Meeszeitpunkt	Pillai's Trace	,671	,003
	Wilks' Lambda	,671	,003
	Hotelling's Trace	,671	,003
	Roy's Largest Root	,671	,003

a. Design: Intercept
Within Subjects Design: Produkttyp + Meeszeitpunkt + Produkttyp * Meeszeitpunkt

b. Exact statistic

Mauchly's Test of Sphericity[a]

Measure: MEASURE_1

Within Subjects Effect	Mauchly's W	Approx. Chi-Square	df	Sig.	Epsilon[b]
					Greenhouse-Geisser
Produkttyp	1,000	,000	0	.	1,000
Meeszeitpunkt	1,000	,000	0	.	1,000
Produkttyp * Meeszeitpunkt	1,000	,000	0	.	1,000

Mauchly's Test of Sphericity[a]

Measure: MEASURE_1

Within Subjects Effect	Epsilon[b]	
	Huynh-Feldt	Lower-bound
Produkttyp	1,000	1,000
Meeszeitpunkt	1,000	1,000
Produkttyp * Meeszeitpunkt	1,000	1,000

Tests the null hypothesis that the error covariance matrix of the orthonormalized transformed dependent variables is proportional to an identity matrix.

a. Design: Intercept
Within Subjects Design: Produkttyp + Meeszeitpunkt + Produkttyp * Meeszeitpunkt

b. May be used to adjust the degrees of freedom for the averaged tests of significance. Corrected tests are displayed in the Tests of Within-Subjects Effects table.

Tests of Within-Subjects Effects

Measure: MEASURE_1

Source		Type III Sum of Squares	df	Mean Square
Produkttyp	Sphericity Assumed	,907	1	,907
	Greenhouse-Geisser	,907	1,000	,907
	Huynh-Feldt	,907	1,000	,907
	Lower-bound	,907	1,000	,907
Error(Produkttyp)	Sphericity Assumed	61,843	61	1,014
	Greenhouse-Geisser	61,843	61,000	1,014
	Huynh-Feldt	61,843	61,000	1,014
	Lower-bound	61,843	61,000	1,014
Meeszeitpunkt	Sphericity Assumed	,101	1	,101
	Greenhouse-Geisser	,101	1,000	,101
	Huynh-Feldt	,101	1,000	,101
	Lower-bound	,101	1,000	,101
Error(Meeszeitpunkt)	Sphericity Assumed	46,649	61	,765
	Greenhouse-Geisser	46,649	61,000	,765
	Huynh-Feldt	46,649	61,000	,765
	Lower-bound	46,649	61,000	,765
Produkttyp * Meeszeitpunkt	Sphericity Assumed	,101	1	,101
	Greenhouse-Geisser	,101	1,000	,101
	Huynh-Feldt	,101	1,000	,101
	Lower-bound	,101	1,000	,101
Error (Produkttyp*Meeszeitpunkt)	Sphericity Assumed	33,649	61	,552
	Greenhouse-Geisser	33,649	61,000	,552
	Huynh-Feldt	33,649	61,000	,552
	Lower-bound	33,649	61,000	,552

Tests of Within-Subjects Effects

Measure: MEASURE_1

Source		F	Sig.	Partial Eta Squared
Produkttyp	Sphericity Assumed	,895	,348	,014
	Greenhouse-Geisser	,895	,348	,014
	Huynh-Feldt	,895	,348	,014
	Lower-bound	,895	,348	,014
Error(Produkttyp)	Sphericity Assumed			
	Greenhouse-Geisser			
	Huynh-Feldt			
	Lower-bound			
Meeszeitpunkt	Sphericity Assumed	,132	,718	,002
	Greenhouse-Geisser	,132	,718	,002
	Huynh-Feldt	,132	,718	,002
	Lower-bound	,132	,718	,002
Error(Meeszeitpunkt)	Sphericity Assumed			
	Greenhouse-Geisser			
	Huynh-Feldt			
	Lower-bound			
Produkttyp * Meeszeitpunkt	Sphericity Assumed	,183	,671	,003
	Greenhouse-Geisser	,183	,671	,003
	Huynh-Feldt	,183	,671	,003
	Lower-bound	,183	,671	,003
Error (Produkttyp*Meeszeitpunkt)	Sphericity Assumed			
	Greenhouse-Geisser			
	Huynh-Feldt			
	Lower-bound			

Tests of Within-Subjects Contrasts

Measure: MEASURE_1

Source	Produkttyp	Meeszeitpunkt	Type III Sum of Squares	df	Mean Square
Produkttyp	Linear		,907	1	,907
Error(Produkttyp)	Linear		61,843	61	1,014
Meeszeitpunkt		Linear	,101	1	,101
Error(Meeszeitpunkt)		Linear	46,649	61	,765
Produkttyp * Meeszeitpunkt	Linear	Linear	,101	1	,101
Error (Produkttyp*Meeszeitpunkt)	Linear	Linear	33,649	61	,552

Tests of Within-Subjects Contrasts

Measure: MEASURE_1

Source	Produkttyp	Meeszeitpunkt	F	Sig.	Partial Eta Squared
Produkttyp	Linear		,895	,348	,014
Error(Produkttyp)	Linear				
Meeszeitpunkt		Linear	,132	,718	,002
Error(Meeszeitpunkt)		Linear			
Produkttyp * Meeszeitpunkt	Linear	Linear	,183	,671	,003
Error (Produkttyp*Meeszeitpunkt)	Linear	Linear			

Tests of Between-Subjects Effects

Measure: MEASURE_1

Transformed Variable: Average

Source	Type III Sum of Squares	df	Mean Square	F	Sig.	Partial Eta Squared
Intercept	6452,520	1	6452,520	3357,539	,000	,982
Error	117,230	61	1,922			

Estimated Marginal Means

Produkttyp

Measure: MEASURE_1

Produkttyp	Mean	Std. Error	95% Confidence Interval	
			Lower Bound	Upper Bound
1	5,040	,100	4,839	5,241
2	5,161	,117	4,928	5,394

```
GLM Sensorik4AdelholzerGlas27.11.12 Sensorik4AdelholzerGlas09.01.13
   Sensorik4Adelholzerplastik27.11.12 Sensorik4AdelholzerPlastik09.01.13
  /WSFACTOR=Produkttyp 2 Polynomial Meeszeitpunkt 2 Polynomial
  /METHOD=SSTYPE(3)
  /EMMEANS=TABLES(Produkttyp)
  /PRINT=DESCRIPTIVE ETASQ
  /CRITERIA=ALPHA(.05)
  /WSDESIGN=Produkttyp Meeszeitpunkt Produkttyp*Meeszeitpunkt.
```

General Linear Model

Notes

Output Created		11-NOV-2013 15:31:37
Comments		
Input	Data	C:\Documents and Settings\Dennis Boywitt\My Documents\My Dropbox\Freiberufliche Tätigkeit\Forschungsring\Daten\Sensorik_Gruppe_1_restructured.sav
	Active Dataset	DataSet2
	Filter	<none>
	Weight	<none>
	Split File	<none>
	N of Rows in Working Data File	65
Missing Value Handling	Definition of Missing	User-defined missing values are treated as missing.
	Cases Used	Statistics are based on all cases with valid data for all variables in the model.
Syntax		GLM Sensorik4AdelholzerGlas27.11.12 Sensorik4AdelholzerGlas09.01.13<br><br>Sensorik4Adelholzerplastik27.11.12 Sensorik4AdelholzerPlastik09.01.13<br>/WSFACTOR=Produkttyp 2 Polynomial Meeszeitpunkt 2 Polynomial<br>/METHOD=SSTYPE(3)<br>/EMMEANS=TABLES (Produkttyp)<br>/PRINT=DESCRIPTIVE ETASQ<br>/CRITERIA=ALPHA(.05)<br>/WSDESIGN=Produkttyp Meeszeitpunkt Produkttyp*Meeszeitpunkt.
Resources	Processor Time	00:00:00,05
	Elapsed Time	00:00:00,06

```
[DataSet2] C:\Documents and Settings\Dennis Boywitt\My Documents\My Dropbox\Freiberufliche Tätigkeit\Forschungsring\Daten\Sensorik_Gruppe_1_restructured.sav
```

Within-Subjects Factors

Measure: MEASURE_1

Produkttyp	Meeszeitpunkt	Dependent Variable
1	1	Sensorik4AdelholzerGlas27.11.12
	2	Sensorik4AdelholzerGlas09.01.13
2	1	Sensorik4Adelholzerplastik27.11.12
	2	Sensorik4AdelholzerPlastik09.01.13

Descriptive Statistics

	Mean	Std. Deviation	N
Sensorik4AdelholzerGlas27.11.12	5,10	1,082	62
Sensorik4AdelholzerGlas09.01.13	5,08	,946	62
Sensorik4Adelholzerplastik27.11.12	5,11	1,088	62
Sensorik4AdelholzerPlastik09.01.13	5,15	,921	62

Multivariate Tests[a]

Effect		Value	F	Hypothesis df	Error df
Produkttyp	Pillai's Trace	,002	,126[b]	1,000	61,000
	Wilks' Lambda	,998	,126[b]	1,000	61,000
	Hotelling's Trace	,002	,126[b]	1,000	61,000
	Roy's Largest Root	,002	,126[b]	1,000	61,000
Meeszeitpunkt	Pillai's Trace	,000	,006[b]	1,000	61,000
	Wilks' Lambda	1,000	,006[b]	1,000	61,000
	Hotelling's Trace	,000	,006[b]	1,000	61,000
	Roy's Largest Root	,000	,006[b]	1,000	61,000
Produkttyp * Meeszeitpunkt	Pillai's Trace	,001	,042[b]	1,000	61,000
	Wilks' Lambda	,999	,042[b]	1,000	61,000
	Hotelling's Trace	,001	,042[b]	1,000	61,000
	Roy's Largest Root	,001	,042[b]	1,000	61,000

Multivariate Tests[a]

Effect		Sig.	Partial Eta Squared
Produkttyp	Pillai's Trace	,723	,002
	Wilks' Lambda	,723	,002
	Hotelling's Trace	,723	,002
	Roy's Largest Root	,723	,002
Meeszeitpunkt	Pillai's Trace	,940	,000
	Wilks' Lambda	,940	,000
	Hotelling's Trace	,940	,000
	Roy's Largest Root	,940	,000
Produkttyp * Meeszeitpunkt	Pillai's Trace	,838	,001
	Wilks' Lambda	,838	,001
	Hotelling's Trace	,838	,001
	Roy's Largest Root	,838	,001

a. Design: Intercept
Within Subjects Design: Produkttyp + Meeszeitpunkt + Produkttyp * Meeszeitpunkt

b. Exact statistic

Mauchly's Test of Sphericity[a]

Measure: MEASURE_1

Within Subjects Effect	Mauchly's W	Approx. Chi-Square	df	Sig.	Epsilon[b]
					Greenhouse-Geisser
Produkttyp	1,000	,000	0	.	1,000
Meeszeitpunkt	1,000	,000	0	.	1,000
Produkttyp * Meeszeitpunkt	1,000	,000	0	.	1,000

Mauchly's Test of Sphericity[a]

Measure: MEASURE_1

Within Subjects Effect	Epsilon[b]	
	Huynh-Feldt	Lower-bound
Produkttyp	1,000	1,000
Meeszeitpunkt	1,000	1,000
Produkttyp * Meeszeitpunkt	1,000	1,000

Tests the null hypothesis that the error covariance matrix of the orthonormalized transformed dependent variables is proportional to an identity matrix.

a. Design: Intercept
Within Subjects Design: Produkttyp + Meeszeitpunkt + Produkttyp * Meeszeitpunkt

b. May be used to adjust the degrees of freedom for the averaged tests of significance. Corrected tests are displayed in the Tests of Within-Subjects Effects table.

Tests of Within-Subjects Effects

Measure: MEASURE_1

Source		Type III Sum of Squares	df	Mean Square
Produkttyp	Sphericity Assumed	,101	1	,101
	Greenhouse-Geisser	,101	1,000	,101
	Huynh-Feldt	,101	1,000	,101
	Lower-bound	,101	1,000	,101
Error(Produkttyp)	Sphericity Assumed	48,649	61	,798
	Greenhouse-Geisser	48,649	61,000	,798
	Huynh-Feldt	48,649	61,000	,798
	Lower-bound	48,649	61,000	,798
Meeszeitpunkt	Sphericity Assumed	,004	1	,004
	Greenhouse-Geisser	,004	1,000	,004
	Huynh-Feldt	,004	1,000	,004
	Lower-bound	,004	1,000	,004
Error(Meeszeitpunkt)	Sphericity Assumed	43,746	61	,717
	Greenhouse-Geisser	43,746	61,000	,717
	Huynh-Feldt	43,746	61,000	,717
	Lower-bound	43,746	61,000	,717
Produkttyp * Meeszeitpunkt	Sphericity Assumed	,036	1	,036
	Greenhouse-Geisser	,036	1,000	,036
	Huynh-Feldt	,036	1,000	,036
	Lower-bound	,036	1,000	,036
Error (Produkttyp*Meeszeitpunkt)	Sphericity Assumed	52,714	61	,864
	Greenhouse-Geisser	52,714	61,000	,864
	Huynh-Feldt	52,714	61,000	,864
	Lower-bound	52,714	61,000	,864

Tests of Within-Subjects Effects

Measure: MEASURE_1

Source		F	Sig.	Partial Eta Squared
Produkttyp	Sphericity Assumed	,126	,723	,002
	Greenhouse-Geisser	,126	,723	,002
	Huynh-Feldt	,126	,723	,002
	Lower-bound	,126	,723	,002
Error(Produkttyp)	Sphericity Assumed			
	Greenhouse-Geisser			
	Huynh-Feldt			
	Lower-bound			
Meeszeitpunkt	Sphericity Assumed	,006	,940	,000
	Greenhouse-Geisser	,006	,940	,000
	Huynh-Feldt	,006	,940	,000
	Lower-bound	,006	,940	,000
Error(Meeszeitpunkt)	Sphericity Assumed			
	Greenhouse-Geisser			
	Huynh-Feldt			
	Lower-bound			
Produkttyp * Meeszeitpunkt	Sphericity Assumed	,042	,838	,001
	Greenhouse-Geisser	,042	,838	,001
	Huynh-Feldt	,042	,838	,001
	Lower-bound	,042	,838	,001
Error (Produkttyp*Meeszeitpunkt)	Sphericity Assumed			
	Greenhouse-Geisser			
	Huynh-Feldt			
	Lower-bound			

Tests of Within-Subjects Contrasts

Measure: MEASURE_1

Source	Produkttyp	Meeszeitpunkt	Type III Sum of Squares	df	Mean Square
Produkttyp	Linear		,101	1	,101
Error(Produkttyp)	Linear		48,649	61	,798
Meeszeitpunkt		Linear	,004	1	,004
Error(Meeszeitpunkt)		Linear	43,746	61	,717
Produkttyp * Meeszeitpunkt	Linear	Linear	,036	1	,036
Error (Produkttyp*Meeszeitpunkt)	Linear	Linear	52,714	61	,864

Tests of Within-Subjects Contrasts

Measure: MEASURE_1

Source	Produkttyp	Meeszeitpunkt	F	Sig.	Partial Eta Squared
Produkttyp	Linear		,126	,723	,002
Error(Produkttyp)	Linear				
Meeszeitpunkt		Linear	,006	,940	,000
Error(Meeszeitpunkt)		Linear			
Produkttyp * Meeszeitpunkt	Linear	Linear	,042	,838	,001
Error (Produkttyp*Meeszeitpunkt)	Linear	Linear			

Tests of Between-Subjects Effects

Measure: MEASURE_1

Transformed Variable: Average

Source	Type III Sum of Squares	df	Mean Square	F	Sig.	Partial Eta Squared
Intercept	6472,940	1	6472,940	3767,269	,000	,984
Error	104,810	61	1,718			

Estimated Marginal Means

Produkttyp

Measure: MEASURE_1

Produkttyp	Mean	Std. Error	95% Confidence Interval	
			Lower Bound	Upper Bound
1	5,089	,095	4,898	5,279
2	5,129	,106	4,917	5,341

```
GLM Sensorik5AdelholzerGlas27.11.12 Sensorik5AdelholzerGlas09.01.13
   Sensorik5Adelholzerplastik27.11.12 Sensorik5AdelholzerPlastik09.01.13
  /WSFACTOR=Produkttyp 2 Polynomial Meeszeitpunkt 2 Polynomial
  /METHOD=SSTYPE(3)
  /EMMEANS=TABLES(Produkttyp)
  /PRINT=DESCRIPTIVE ETASQ
  /CRITERIA=ALPHA(.05)
  /WSDESIGN=Produkttyp Meeszeitpunkt Produkttyp*Meeszeitpunkt.
```

General Linear Model

Notes

Output Created		11-NOV-2013 15:32:19
Comments		
Input	Data	C:\Documents and Settings\Dennis Boywitt\My Documents\My Dropbox\Freiberufliche Tätigkeit\Forschungsring\Daten\Sensorik_Gruppe_1_restructured.sav
	Active Dataset	DataSet2
	Filter	<none>
	Weight	<none>
	Split File	<none>
	N of Rows in Working Data File	65
Missing Value Handling	Definition of Missing	User-defined missing values are treated as missing.
	Cases Used	Statistics are based on all cases with valid data for all variables in the model.
Syntax		GLM Sensorik5AdelholzerGlas27.11.12 Sensorik5AdelholzerGlas09.01.13<br><br>Sensorik5Adelholzerplastik27.11.12 Sensorik5AdelholzerPlastik09.01.13<br>/WSFACTOR=Produkttyp 2 Polynomial Meeszeitpunkt 2 Polynomial<br>/METHOD=SSTYPE(3)<br>/EMMEANS=TABLES(Produkttyp)<br>/PRINT=DESCRIPTIVE ETASQ<br>/CRITERIA=ALPHA(.05)<br>/WSDESIGN=Produkttyp Meeszeitpunkt Produkttyp*Meeszeitpunkt.
Resources	Processor Time	00:00:00,03
	Elapsed Time	00:00:00,13

[DataSet2] C:\Documents and Settings\Dennis Boywitt\My Documents\My Dropbox\Freiberufliche Tätigkeit\Forschungsring\Daten\Sensorik_Gruppe_1_restructured.sav

Within-Subjects Factors

Measure: MEASURE_1

Produkttyp	Meeszeitpunkt	Dependent Variable
1	1	Sensorik5AdelholzerGlas27.11.12
	2	Sensorik5AdelholzerGlas09.01.13
2	1	Sensorik5Adelholzerplastik27.11.12
	2	Sensorik5AdelholzerPlastik09.01.13

Descriptive Statistics

	Mean	Std. Deviation	N
Sensorik5AdelholzerGlas27.11.12	5,08	,911	62
Sensorik5AdelholzerGlas09.01.13	5,03	,975	62
Sensorik5Adelholzerplastik27.11.12	5,10	1,020	62
Sensorik5AdelholzerPlastik09.01.13	5,18	,878	62

Multivariate Tests[a]

Effect		Value	F	Hypothesis df	Error df
Produkttyp	Pillai's Trace	,009	,564[b]	1,000	61,000
	Wilks' Lambda	,991	,564[b]	1,000	61,000
	Hotelling's Trace	,009	,564[b]	1,000	61,000
	Roy's Largest Root	,009	,564[b]	1,000	61,000
Meeszeitpunkt	Pillai's Trace	,000	,026[b]	1,000	61,000
	Wilks' Lambda	1,000	,026[b]	1,000	61,000
	Hotelling's Trace	,000	,026[b]	1,000	61,000
	Roy's Largest Root	,000	,026[b]	1,000	61,000
Produkttyp * Meeszeitpunkt	Pillai's Trace	,009	,548[b]	1,000	61,000
	Wilks' Lambda	,991	,548[b]	1,000	61,000
	Hotelling's Trace	,009	,548[b]	1,000	61,000
	Roy's Largest Root	,009	,548[b]	1,000	61,000

Multivariate Tests[a]

Effect		Sig.	Partial Eta Squared
Produkttyp	Pillai's Trace	,455	,009
	Wilks' Lambda	,455	,009
	Hotelling's Trace	,455	,009
	Roy's Largest Root	,455	,009
Meeszeitpunkt	Pillai's Trace	,873	,000
	Wilks' Lambda	,873	,000
	Hotelling's Trace	,873	,000
	Roy's Largest Root	,873	,000
Produkttyp * Meeszeitpunkt	Pillai's Trace	,462	,009
	Wilks' Lambda	,462	,009
	Hotelling's Trace	,462	,009
	Roy's Largest Root	,462	,009

a. Design: Intercept
Within Subjects Design: Produkttyp + Meeszeitpunkt + Produkttyp * Meeszeitpunkt

b. Exact statistic

Mauchly's Test of Sphericity[a]

Measure: MEASURE_1

Within Subjects Effect	Mauchly's W	Approx. Chi-Square	df	Sig.	Epsilon[b]
					Greenhouse-Geisser
Produkttyp	1,000	,000	0	.	1,000
Meeszeitpunkt	1,000	,000	0	.	1,000
Produkttyp * Meeszeitpunkt	1,000	,000	0	.	1,000

Mauchly's Test of Sphericity[a]

Measure: MEASURE_1

Within Subjects Effect	Epsilon[b]	
	Huynh-Feldt	Lower-bound
Produkttyp	1,000	1,000
Meeszeitpunkt	1,000	1,000
Produkttyp * Meeszeitpunkt	1,000	1,000

Tests the null hypothesis that the error covariance matrix of the orthonormalized transformed dependent variables is proportional to an identity matrix.

a. Design: Intercept
Within Subjects Design: Produkttyp + Meeszeitpunkt + Produkttyp * Meeszeitpunkt

b. May be used to adjust the degrees of freedom for the averaged tests of significance. Corrected tests are displayed in the Tests of Within-Subjects Effects table.

Tests of Within-Subjects Effects

Measure: MEASURE_1

Source		Type III Sum of Squares	df	Mean Square
Produkttyp	Sphericity Assumed	,403	1	,403
	Greenhouse-Geisser	,403	1,000	,403
	Huynh-Feldt	,403	1,000	,403
	Lower-bound	,403	1,000	,403
Error(Produkttyp)	Sphericity Assumed	43,597	61	,715
	Greenhouse-Geisser	43,597	61,000	,715
	Huynh-Feldt	43,597	61,000	,715
	Lower-bound	43,597	61,000	,715
Meeszeitpunkt	Sphericity Assumed	,016	1	,016
	Greenhouse-Geisser	,016	1,000	,016
	Huynh-Feldt	,016	1,000	,016
	Lower-bound	,016	1,000	,016
Error(Meeszeitpunkt)	Sphericity Assumed	37,984	61	,623
	Greenhouse-Geisser	37,984	61,000	,623
	Huynh-Feldt	37,984	61,000	,623
	Lower-bound	37,984	61,000	,623
Produkttyp * Meeszeitpunkt	Sphericity Assumed	,258	1	,258
	Greenhouse-Geisser	,258	1,000	,258
	Huynh-Feldt	,258	1,000	,258
	Lower-bound	,258	1,000	,258
Error (Produkttyp*Meeszeitpunkt)	Sphericity Assumed	28,742	61	,471
	Greenhouse-Geisser	28,742	61,000	,471
	Huynh-Feldt	28,742	61,000	,471
	Lower-bound	28,742	61,000	,471

Tests of Within-Subjects Effects

Measure: MEASURE_1

Source		F	Sig.	Partial Eta Squared
Produkttyp	Sphericity Assumed	,564	,455	,009
	Greenhouse-Geisser	,564	,455	,009
	Huynh-Feldt	,564	,455	,009
	Lower-bound	,564	,455	,009
Error(Produkttyp)	Sphericity Assumed			
	Greenhouse-Geisser			
	Huynh-Feldt			
	Lower-bound			
Meeszeitpunkt	Sphericity Assumed	,026	,873	,000
	Greenhouse-Geisser	,026	,873	,000
	Huynh-Feldt	,026	,873	,000
	Lower-bound	,026	,873	,000
Error(Meeszeitpunkt)	Sphericity Assumed			
	Greenhouse-Geisser			
	Huynh-Feldt			
	Lower-bound			
Produkttyp * Meeszeitpunkt	Sphericity Assumed	,548	,462	,009
	Greenhouse-Geisser	,548	,462	,009
	Huynh-Feldt	,548	,462	,009
	Lower-bound	,548	,462	,009
Error (Produkttyp*Meeszeitpunkt)	Sphericity Assumed			
	Greenhouse-Geisser			
	Huynh-Feldt			
	Lower-bound			

Tests of Within-Subjects Contrasts

Measure: MEASURE_1

Source	Produkttyp	Meeszeitpunkt	Type III Sum of Squares	df	Mean Square
Produkttyp	Linear		,403	1	,403
Error(Produkttyp)	Linear		43,597	61	,715
Meeszeitpunkt		Linear	,016	1	,016
Error(Meeszeitpunkt)		Linear	37,984	61	,623
Produkttyp * Meeszeitpunkt	Linear	Linear	,258	1	,258
Error (Produkttyp*Meeszeitpunkt)	Linear	Linear	28,742	61	,471

Tests of Within-Subjects Contrasts

Measure: MEASURE_1

Source	Produkttyp	Meeszeitpunkt	F	Sig.	Partial Eta Squared
Produkttyp	Linear		,564	,455	,009
Error(Produkttyp)	Linear				
Meeszeitpunkt		Linear	,026	,873	,000
Error(Meeszeitpunkt)		Linear			
Produkttyp * Meeszeitpunkt	Linear	Linear	,548	,462	,009
Error (Produkttyp*Meeszeitpunkt)	Linear	Linear			

Tests of Between-Subjects Effects

Measure: MEASURE_1

Transformed Variable: Average

Source	Type III Sum of Squares	df	Mean Square	F	Sig.	Partial Eta Squared
Intercept	6442,323	1	6442,323	3616,038	,000	,983
Error	108,677	61	1,782			

Estimated Marginal Means

Produkttyp

Measure: MEASURE_1

Produkttyp	Mean	Std. Error	95% Confidence Interval	
			Lower Bound	Upper Bound
1	5,056	,093	4,870	5,243
2	5,137	,107	4,924	5,351
