# Supplementary material for: Development and Application of a Test for Food-Induced Emotions
Source: PLoS One. 2016 Nov 18;11(11):e0165991. doi: 10.1371/journal.pone.0165991 (PMC5115674; doi:10.1371/journal.pone.0165991)
Supplement: S10 File — (PDF) [file pone.0165991.s013.pdf]

```
 ************************
*Milch

*** Verlgeich Söbbeke vs. Landliebe am 27.11.12 & am 09.01.13 -> Sensorik 1

GLM Sensorik1Söbbeke27.11.12 Sensorik1Söbbeke09.01.13
    Sensorik1Landliebe27.11.12 Sensorik1Landliebe09.01.13
  /WSFACTOR=Produkttyp 2 Polynomial Meeszeitpunkt 2 Polynomial
  /METHOD=SSTYPE(3)
  /EMMEANS=TABLES(Produkttyp)
  /PRINT=DESCRIPTIVE ETASQ
  /CRITERIA=ALPHA(.05)
  /WSDESIGN=Produkttyp Meeszeitpunkt Produkttyp*Meeszeitpunkt.
```

General Linear Model

Notes

Output Created		11-NOV-2013 15:33:05
Comments		
Input	Data	C:\Documents and Settings\Dennis Boywitt\My Documents\My Dropbox\Freiberufliche Tätigkeit\Forschungsring\Daten\Sensorik_Gruppe_1_restructured.sav
	Active Dataset	DataSet2
	Filter	<none>
	Weight	<none>
	Split File	<none>
	N of Rows in Working Data File	65
Missing Value Handling	Definition of Missing	User-defined missing values are treated as missing.
	Cases Used	Statistics are based on all cases with valid data for all variables in the model.

Notes

Syntax		GLM Sensorik1Söbbeke27.11.12 Sensorik1Söbbeke09.01.13 Sensorik1Landliebe27.11.12 Sensorik1Landliebe09.01.13<br>/WSFACTOR=Produkttyp 2 Polynomial Meeszeitpunkt 2 Polynomial<br>/METHOD=SSTYPE(3)<br>/EMMEANS=TABLES (Produkttyp)<br>/PRINT=DESCRIPTIVE ETASQ<br>/CRITERIA=ALPHA(.05)<br>/WSDESIGN=Produkttyp Meeszeitpunkt Produkttyp*Meeszeitpunkt.
Resources	Processor Time	00:00:00,03
	Elapsed Time	00:00:00,05

```
[DataSet2] C:\Documents and Settings\Dennis Boywitt\My Documents\My Dropbo
x\Freiberufliche Tätigkeit\Forschungsring\Daten\Sensorik_Gruppe_1_restruct
ured.sav
```

Within-Subjects Factors

Measure: MEASURE_1

Produkttyp	Meeszeitpunkt	Dependent Variable
1	1	Sensorik1Söbbeke27.11.12
	2	Sensorik1Söbbeke09.01.13
2	1	Sensorik1Landliebe27.11.12
	2	Sensorik1Landliebe09.01.13

Descriptive Statistics

	Mean	Std. Deviation	N
Sensorik1Söbbeke27.11.12	4,95	1,237	63
Sensorik1Söbbeke09.01.13	4,92	1,154	63
Sensorik1Landliebe27.11.12	5,59	1,186	63
Sensorik1Landliebe09.01.13	5,25	1,047	63

Multivariate Tests[a]

Effect		Value	F	Hypothesis df	Error df
Produkttyp	Pillai's Trace	,245	20,128[b]	1,000	62,000
	Wilks' Lambda	,755	20,128[b]	1,000	62,000
	Hotelling's Trace	,325	20,128[b]	1,000	62,000
	Roy's Largest Root	,325	20,128[b]	1,000	62,000
Meeszeitpunkt	Pillai's Trace	,069	4,623[b]	1,000	62,000
	Wilks' Lambda	,931	4,623[b]	1,000	62,000
	Hotelling's Trace	,075	4,623[b]	1,000	62,000
	Roy's Largest Root	,075	4,623[b]	1,000	62,000
Produkttyp * Meeszeitpunkt	Pillai's Trace	,046	2,979[b]	1,000	62,000
	Wilks' Lambda	,954	2,979[b]	1,000	62,000
	Hotelling's Trace	,048	2,979[b]	1,000	62,000
	Roy's Largest Root	,048	2,979[b]	1,000	62,000

Multivariate Tests[a]

Effect		Sig.	Partial Eta Squared
Produkttyp	Pillai's Trace	,000	,245
	Wilks' Lambda	,000	,245
	Hotelling's Trace	,000	,245
	Roy's Largest Root	,000	,245
Meeszeitpunkt	Pillai's Trace	,035	,069
	Wilks' Lambda	,035	,069
	Hotelling's Trace	,035	,069
	Roy's Largest Root	,035	,069
Produkttyp * Meeszeitpunkt	Pillai's Trace	,089	,046
	Wilks' Lambda	,089	,046
	Hotelling's Trace	,089	,046
	Roy's Largest Root	,089	,046

a. Design: Intercept
Within Subjects Design: Produkttyp + Meeszeitpunkt + Produkttyp * Meeszeitpunkt

b. Exact statistic

Mauchly's Test of Sphericity[a]

Measure: MEASURE_1

Within Subjects Effect	Mauchly's W	Approx. Chi-Square	df	Sig.	Epsilon[b] Greenhouse-Geisser
Produkttyp	1,000	,000	0	.	1,000
Meeszeitpunkt	1,000	,000	0	.	1,000
Produkttyp * Meeszeitpunkt	1,000	,000	0	.	1,000

Mauchly's Test of Sphericity[a]

Measure: MEASURE_1

	Epsilon[b]	
Within Subjects Effect	Huynh-Feldt	Lower-bound
Produkttyp	1,000	1,000
Meeszeitpunkt	1,000	1,000
Produkttyp * Meeszeitpunkt	1,000	1,000

Tests the null hypothesis that the error covariance matrix of the orthonormalized transformed dependent variables is proportional to an identity matrix.

a. Design: Intercept
Within Subjects Design: Produkttyp + Meeszeitpunkt + Produkttyp * Meeszeitpunkt

b. May be used to adjust the degrees of freedom for the averaged tests of significance. Corrected tests are displayed in the Tests of Within-Subjects Effects table.

Tests of Within-Subjects Effects

Measure: MEASURE_1

Source		Type III Sum of Squares	df	Mean Square
Produkttyp	Sphericity Assumed	14,766	1	14,766
	Greenhouse-Geisser	14,766	1,000	14,766
	Huynh-Feldt	14,766	1,000	14,766
	Lower-bound	14,766	1,000	14,766
Error(Produkttyp)	Sphericity Assumed	45,484	62	,734
	Greenhouse-Geisser	45,484	62,000	,734
	Huynh-Feldt	45,484	62,000	,734
	Lower-bound	45,484	62,000	,734
Meeszeitpunkt	Sphericity Assumed	2,099	1	2,099
	Greenhouse-Geisser	2,099	1,000	2,099
	Huynh-Feldt	2,099	1,000	2,099
	Lower-bound	2,099	1,000	2,099
Error(Meeszeitpunkt)	Sphericity Assumed	28,151	62	,454
	Greenhouse-Geisser	28,151	62,000	,454
	Huynh-Feldt	28,151	62,000	,454
	Lower-bound	28,151	62,000	,454
Produkttyp * Meeszeitpunkt	Sphericity Assumed	1,433	1	1,433
	Greenhouse-Geisser	1,433	1,000	1,433
	Huynh-Feldt	1,433	1,000	1,433
	Lower-bound	1,433	1,000	1,433
Error (Produkttyp*Meeszeitpunkt)	Sphericity Assumed	29,817	62	,481
	Greenhouse-Geisser	29,817	62,000	,481
	Huynh-Feldt	29,817	62,000	,481
	Lower-bound	29,817	62,000	,481

Tests of Within-Subjects Effects

Measure: MEASURE_1

Source		F	Sig.	Partial Eta Squared
Produkttyp	Sphericity Assumed	20,128	,000	,245
	Greenhouse-Geisser	20,128	,000	,245
	Huynh-Feldt	20,128	,000	,245
	Lower-bound	20,128	,000	,245
Error(Produkttyp)	Sphericity Assumed			
	Greenhouse-Geisser			
	Huynh-Feldt			
	Lower-bound			
Meeszeitpunkt	Sphericity Assumed	4,623	,035	,069
	Greenhouse-Geisser	4,623	,035	,069
	Huynh-Feldt	4,623	,035	,069
	Lower-bound	4,623	,035	,069
Error(Meeszeitpunkt)	Sphericity Assumed			
	Greenhouse-Geisser			
	Huynh-Feldt			
	Lower-bound			
Produkttyp * Meeszeitpunkt	Sphericity Assumed	2,979	,089	,046
	Greenhouse-Geisser	2,979	,089	,046
	Huynh-Feldt	2,979	,089	,046
	Lower-bound	2,979	,089	,046
Error (Produkttyp*Meeszeitpunkt)	Sphericity Assumed			
	Greenhouse-Geisser			
	Huynh-Feldt			
	Lower-bound			

Tests of Within-Subjects Contrasts

Measure: MEASURE_1

Source	Produkttyp	Meeszeitpunkt	Type III Sum of Squares	df	Mean Square
Produkttyp	Linear		14,766	1	14,766
Error(Produkttyp)	Linear		45,484	62	,734
Meeszeitpunkt		Linear	2,099	1	2,099
Error(Meeszeitpunkt)		Linear	28,151	62	,454
Produkttyp * Meeszeitpunkt	Linear	Linear	1,433	1	1,433
Error (Produkttyp*Meeszeitpunkt)	Linear	Linear	29,817	62	,481

Tests of Within-Subjects Contrasts

Measure: MEASURE_1

Source	Produkttyp	Meeszeitpunkt	F	Sig.	Partial Eta Squared
Produkttyp	Linear		20,128	,000	,245
Error(Produkttyp)	Linear				
Meeszeitpunkt		Linear	4,623	,035	,069
Error(Meeszeitpunkt)		Linear			
Produkttyp * Meeszeitpunkt	Linear	Linear	2,979	,089	,046
Error (Produkttyp*Meeszeitpunkt)	Linear	Linear			

Tests of Between-Subjects Effects

Measure: MEASURE_1

Transformed Variable: Average

Source	Type III Sum of Squares	df	Mean Square	F	Sig.	Partial Eta Squared
Intercept	6758,036	1	6758,036	1827,976	,000	,967
Error	229,214	62	3,697			

Estimated Marginal Means

Produkttyp

Measure: MEASURE_1

Produkttyp	Mean	Std. Error	95% Confidence Interval	
			Lower Bound	Upper Bound
1	4,937	,135	4,667	5,206
2	5,421	,130	5,161	5,681

```
GLM Sensorik2Söbbeke27.11.12 Sensorik2Söbbeke09.01.13
   Sensorik2Landliebe27.11.12 Sensorik2Landliebe09.01.13
 /WSFACTOR=Produkttyp 2 Polynomial Meeszeitpunkt 2 Polynomial
 /METHOD=SSTYPE(3)
 /EMMEANS=TABLES(Produkttyp)
 /PRINT=DESCRIPTIVE ETASQ
 /CRITERIA=ALPHA(.05)
 /WSDESIGN=Produkttyp Meeszeitpunkt Produkttyp*Meeszeitpunkt.
```

General Linear Model

Notes

Output Created		11-NOV-2013 15:36:18
Comments		
Input	Data	C:\Documents and Settings\Dennis Boywitt\My Documents\My Dropbox\Freiberufliche Tätigkeit\Forschungsring\Daten\Sensorik_Gruppe_1_restructured.sav
	Active Dataset	DataSet2
	Filter	<none>
	Weight	<none>
	Split File	<none>
	N of Rows in Working Data File	65
Missing Value Handling	Definition of Missing	User-defined missing values are treated as missing.
	Cases Used	Statistics are based on all cases with valid data for all variables in the model.
Syntax		GLM Sensorik2Söbbeke27.11.12 Sensorik2Söbbeke09.01.13 Sensorik2Landliebe27.11.12 Sensorik2Landliebe09.01.13 /WSFACTOR=Produkttyp 2 Polynomial Meeszeitpunkt 2 Polynomial /METHOD=SSTYPE(3) /EMMEANS=TABLES(Produkttyp) /PRINT=DESCRIPTIVE ETASQ /CRITERIA=ALPHA(.05) /WSDESIGN=Produkttyp Meeszeitpunkt Produkttyp*Meeszeitpunkt.
Resources	Processor Time	00:00:00,02
	Elapsed Time	00:00:00,03

```
[DataSet2] C:\Documents and Settings\Dennis Boywitt\My Documents\My Dropbox\Freiberufliche Tätigkeit\Forschungsring\Daten\Sensorik_Gruppe_1_restructured.sav
```

Within-Subjects Factors

Measure: MEASURE_1

Produkttyp	Meeszeitpunkt	Dependent Variable
1	1	Sensorik2Söbbeke27.11.12
	2	Sensorik2Söbbeke09.01.13
2	1	Sensorik2Landliebe27.11.12
	2	Sensorik2Landliebe09.01.13

Descriptive Statistics

	Mean	Std. Deviation	N
Sensorik2Söbbeke27.11.12	5,05	,982	62
Sensorik2Söbbeke09.01.13	4,98	,914	62
Sensorik2Landliebe27.11.12	5,31	1,001	62
Sensorik2Landliebe09.01.13	4,52	1,277	62

Multivariate Tests[a]

Effect		Value	F	Hypothesis df	Error df
Produkttyp	Pillai's Trace	,012	,728[b]	1,000	61,000
	Wilks' Lambda	,988	,728[b]	1,000	61,000
	Hotelling's Trace	,012	,728[b]	1,000	61,000
	Roy's Largest Root	,012	,728[b]	1,000	61,000
Meeszeitpunkt	Pillai's Trace	,237	18,969[b]	1,000	61,000
	Wilks' Lambda	,763	18,969[b]	1,000	61,000
	Hotelling's Trace	,311	18,969[b]	1,000	61,000
	Roy's Largest Root	,311	18,969[b]	1,000	61,000
Produkttyp * Meeszeitpunkt	Pillai's Trace	,211	16,285[b]	1,000	61,000
	Wilks' Lambda	,789	16,285[b]	1,000	61,000
	Hotelling's Trace	,267	16,285[b]	1,000	61,000
	Roy's Largest Root	,267	16,285[b]	1,000	61,000

Multivariate Tests[a]

Effect		Sig.	Partial Eta Squared
Produkttyp	Pillai's Trace	,397	,012
	Wilks' Lambda	,397	,012
	Hotelling's Trace	,397	,012
	Roy's Largest Root	,397	,012
Meeszeitpunkt	Pillai's Trace	,000	,237
	Wilks' Lambda	,000	,237
	Hotelling's Trace	,000	,237
	Roy's Largest Root	,000	,237
Produkttyp * Meeszeitpunkt	Pillai's Trace	,000	,211
	Wilks' Lambda	,000	,211
	Hotelling's Trace	,000	,211
	Roy's Largest Root	,000	,211

a. Design: Intercept
Within Subjects Design: Produkttyp + Meeszeitpunkt + Produkttyp * Meeszeitpunkt

b. Exact statistic

Mauchly's Test of Sphericity[a]

Measure: MEASURE_1

Within Subjects Effect	Mauchly's W	Approx. Chi-Square	df	Sig.	Epsilon[b]
					Greenhouse-Geisser
Produkttyp	1,000	,000	0	.	1,000
Meeszeitpunkt	1,000	,000	0	.	1,000
Produkttyp * Meeszeitpunkt	1,000	,000	0	.	1,000

Mauchly's Test of Sphericity[a]

Measure: MEASURE_1

Within Subjects Effect	Epsilon[b]	
	Huynh-Feldt	Lower-bound
Produkttyp	1,000	1,000
Meeszeitpunkt	1,000	1,000
Produkttyp * Meeszeitpunkt	1,000	1,000

Tests the null hypothesis that the error covariance matrix of the orthonormalized transformed dependent variables is proportional to an identity matrix.

a. Design: Intercept
Within Subjects Design: Produkttyp + Meeszeitpunkt + Produkttyp * Meeszeitpunkt

b. May be used to adjust the degrees of freedom for the averaged tests of significance. Corrected tests are displayed in the Tests of Within-Subjects Effects table.

Tests of Within-Subjects Effects

Measure: MEASURE_1

Source		Type III Sum of Squares	df	Mean Square
Produkttyp	Sphericity Assumed	,681	1	,681
	Greenhouse-Geisser	,681	1,000	,681
	Huynh-Feldt	,681	1,000	,681
	Lower-bound	,681	1,000	,681
Error(Produkttyp)	Sphericity Assumed	57,069	61	,936
	Greenhouse-Geisser	57,069	61,000	,936
	Huynh-Feldt	57,069	61,000	,936
	Lower-bound	57,069	61,000	,936
Meeszeitpunkt	Sphericity Assumed	11,327	1	11,327
	Greenhouse-Geisser	11,327	1,000	11,327
	Huynh-Feldt	11,327	1,000	11,327
	Lower-bound	11,327	1,000	11,327
Error(Meeszeitpunkt)	Sphericity Assumed	36,423	61	,597
	Greenhouse-Geisser	36,423	61,000	,597
	Huynh-Feldt	36,423	61,000	,597
	Lower-bound	36,423	61,000	,597
Produkttyp * Meeszeitpunkt	Sphericity Assumed	8,165	1	8,165
	Greenhouse-Geisser	8,165	1,000	8,165
	Huynh-Feldt	8,165	1,000	8,165
	Lower-bound	8,165	1,000	8,165
Error (Produkttyp*Meeszeitpunkt)	Sphericity Assumed	30,585	61	,501
	Greenhouse-Geisser	30,585	61,000	,501
	Huynh-Feldt	30,585	61,000	,501
	Lower-bound	30,585	61,000	,501

Tests of Within-Subjects Effects

Measure: MEASURE_1

Source		F	Sig.	Partial Eta Squared
Produkttyp	Sphericity Assumed	,728	,397	,012
	Greenhouse-Geisser	,728	,397	,012
	Huynh-Feldt	,728	,397	,012
	Lower-bound	,728	,397	,012
Error(Produkttyp)	Sphericity Assumed			
	Greenhouse-Geisser			
	Huynh-Feldt			
	Lower-bound			
Meeszeitpunkt	Sphericity Assumed	18,969	,000	,237
	Greenhouse-Geisser	18,969	,000	,237
	Huynh-Feldt	18,969	,000	,237
	Lower-bound	18,969	,000	,237
Error(Meeszeitpunkt)	Sphericity Assumed			
	Greenhouse-Geisser			
	Huynh-Feldt			
	Lower-bound			
Produkttyp * Meeszeitpunkt	Sphericity Assumed	16,285	,000	,211
	Greenhouse-Geisser	16,285	,000	,211
	Huynh-Feldt	16,285	,000	,211
	Lower-bound	16,285	,000	,211
Error (Produkttyp*Meeszeitpunkt)	Sphericity Assumed			
	Greenhouse-Geisser			
	Huynh-Feldt			
	Lower-bound			

Tests of Within-Subjects Contrasts

Measure: MEASURE_1

Source	Produkttyp	Meeszeitpunkt	Type III Sum of Squares	df	Mean Square
Produkttyp	Linear		,681	1	,681
Error(Produkttyp)	Linear		57,069	61	,936
Meeszeitpunkt		Linear	11,327	1	11,327
Error(Meeszeitpunkt)		Linear	36,423	61	,597
Produkttyp * Meeszeitpunkt	Linear	Linear	8,165	1	8,165
Error (Produkttyp*Meeszeitpunkt)	Linear	Linear	30,585	61	,501

Tests of Within-Subjects Contrasts

Measure: MEASURE_1

Source	Produkttyp	Meeszeitpunkt	F	Sig.	Partial Eta Squared
Produkttyp	Linear		,728	,397	,012
Error(Produkttyp)	Linear				
Meeszeitpunkt		Linear	18,969	,000	,237
Error(Meeszeitpunkt)		Linear			
Produkttyp * Meeszeitpunkt	Linear	Linear	16,285	,000	,211
Error (Produkttyp*Meeszeitpunkt)	Linear	Linear			

Tests of Between-Subjects Effects

Measure: MEASURE_1

Transformed Variable: Average

Source	Type III Sum of Squares	df	Mean Square	F	Sig.	Partial Eta Squared
Intercept	6110,327	1	6110,327	2545,563	,000	,977
Error	146,423	61	2,400			

Estimated Marginal Means

Produkttyp

Measure: MEASURE_1

Produkttyp	Mean	Std. Error	95% Confidence Interval	
			Lower Bound	Upper Bound
1	5,016	,104	4,808	5,224
2	4,911	,127	4,658	5,165

```
GLM Sensorik3Söbbeke27.11.12 Sensorik3Söbbeke09.01.13
   Sensorik3Landliebe27.11.12 Sensorik3Landliebe09.01.13
 /WSFACTOR=Produkttyp 2 Polynomial Meeszeitpunkt 2 Polynomial
 /METHOD=SSTYPE(3)
 /EMMEANS=TABLES(Produkttyp)
 /PRINT=DESCRIPTIVE ETASQ
 /CRITERIA=ALPHA(.05)
 /WSDESIGN=Produkttyp Meeszeitpunkt Produkttyp*Meeszeitpunkt.
```

General Linear Model

Notes

Output Created		11-NOV-2013 15:38:37
Comments		
Input	Data	C:\Documents and Settings\Dennis Boywitt\My Documents\My Dropbox\Freiberufliche Tätigkeit\Forschungsring\Daten\Sensorik_Gruppe_1_restructured.sav
	Active Dataset	DataSet2
	Filter	<none>
	Weight	<none>
	Split File	<none>
	N of Rows in Working Data File	65
Missing Value Handling	Definition of Missing	User-defined missing values are treated as missing.
	Cases Used	Statistics are based on all cases with valid data for all variables in the model.
Syntax		GLM Sensorik3Söbbeke27.11.12 Sensorik3Söbbeke09.01.13 Sensorik3Landliebe27.11.12 Sensorik3Landliebe09.01.13 /WSFACTOR=Produkttyp 2 Polynomial Meeszeitpunkt 2 Polynomial /METHOD=SSTYPE(3) /EMMEANS=TABLES(Produkttyp) /PRINT=DESCRIPTIVE ETASQ /CRITERIA=ALPHA(.05) /WSDESIGN=Produkttyp Meeszeitpunkt Produkttyp*Meeszeitpunkt.
Resources	Processor Time	00:00:00,02
	Elapsed Time	00:00:00,02

```
[DataSet2] C:\Documents and Settings\Dennis Boywitt\My Documents\My Dropbox\Freiberufliche Tätigkeit\Forschungsring\Daten\Sensorik_Gruppe_1_restructured.sav
```

Within-Subjects Factors

Measure: MEASURE_1

Produkttyp	Meeszeitpunkt	Dependent Variable
1	1	Sensorik3Söbbeke27.11.12
	2	Sensorik3Söbbeke09.01.13
2	1	Sensorik3Landliebe27.11.12
	2	Sensorik3Landliebe09.01.13

Descriptive Statistics

	Mean	Std. Deviation	N
Sensorik3Söbbeke27.11.12	5,11	1,057	62
Sensorik3Söbbeke09.01.13	5,16	1,104	62
Sensorik3Landliebe27.11.12	5,27	1,176	62
Sensorik3Landliebe09.01.13	3,87	1,443	62

Multivariate Tests[a]

Effect		Value	F	Hypothesis df	Error df
Produkttyp	Pillai's Trace	,163	11,846[b]	1,000	61,000
	Wilks' Lambda	,837	11,846[b]	1,000	61,000
	Hotelling's Trace	,194	11,846[b]	1,000	61,000
	Roy's Largest Root	,194	11,846[b]	1,000	61,000
Meeszeitpunkt	Pillai's Trace	,349	32,716[b]	1,000	61,000
	Wilks' Lambda	,651	32,716[b]	1,000	61,000
	Hotelling's Trace	,536	32,716[b]	1,000	61,000
	Roy's Largest Root	,536	32,716[b]	1,000	61,000
Produkttyp * Meeszeitpunkt	Pillai's Trace	,298	25,929[b]	1,000	61,000
	Wilks' Lambda	,702	25,929[b]	1,000	61,000
	Hotelling's Trace	,425	25,929[b]	1,000	61,000
	Roy's Largest Root	,425	25,929[b]	1,000	61,000

Multivariate Tests[a]

Effect		Sig.	Partial Eta Squared
Produkttyp	Pillai's Trace	,001	,163
	Wilks' Lambda	,001	,163
	Hotelling's Trace	,001	,163
	Roy's Largest Root	,001	,163
Meeszeitpunkt	Pillai's Trace	,000	,349
	Wilks' Lambda	,000	,349
	Hotelling's Trace	,000	,349
	Roy's Largest Root	,000	,349
Produkttyp * Meeszeitpunkt	Pillai's Trace	,000	,298
	Wilks' Lambda	,000	,298
	Hotelling's Trace	,000	,298
	Roy's Largest Root	,000	,298

a. Design: Intercept
Within Subjects Design: Produkttyp + Meeszeitpunkt + Produkttyp * Meeszeitpunkt

b. Exact statistic

Mauchly's Test of Sphericity[a]

Measure: MEASURE_1

Within Subjects Effect	Mauchly's W	Approx. Chi-Square	df	Sig.	Epsilon[b]
					Greenhouse-Geisser
Produkttyp	1,000	,000	0	.	1,000
Meeszeitpunkt	1,000	,000	0	.	1,000
Produkttyp * Meeszeitpunkt	1,000	,000	0	.	1,000

Mauchly's Test of Sphericity[a]

Measure: MEASURE_1

Within Subjects Effect	Epsilon[b]	
	Huynh-Feldt	Lower-bound
Produkttyp	1,000	1,000
Meeszeitpunkt	1,000	1,000
Produkttyp * Meeszeitpunkt	1,000	1,000

Tests the null hypothesis that the error covariance matrix of the orthonormalized transformed dependent variables is proportional to an identity matrix.

a. Design: Intercept
Within Subjects Design: Produkttyp + Meeszeitpunkt + Produkttyp * Meeszeitpunkt

b. May be used to adjust the degrees of freedom for the averaged tests of significance. Corrected tests are displayed in the Tests of Within-Subjects Effects table.

Tests of Within-Subjects Effects

Measure: MEASURE_1

Source		Type III Sum of Squares	df	Mean Square
Produkttyp	Sphericity Assumed	19,758	1	19,758
	Greenhouse-Geisser	19,758	1,000	19,758
	Huynh-Feldt	19,758	1,000	19,758
	Lower-bound	19,758	1,000	19,758
Error(Produkttyp)	Sphericity Assumed	101,742	61	1,668
	Greenhouse-Geisser	101,742	61,000	1,668
	Huynh-Feldt	101,742	61,000	1,668
	Lower-bound	101,742	61,000	1,668
Meeszeitpunkt	Sphericity Assumed	28,452	1	28,452
	Greenhouse-Geisser	28,452	1,000	28,452
	Huynh-Feldt	28,452	1,000	28,452
	Lower-bound	28,452	1,000	28,452
Error(Meeszeitpunkt)	Sphericity Assumed	53,048	61	,870
	Greenhouse-Geisser	53,048	61,000	,870
	Huynh-Feldt	53,048	61,000	,870
	Lower-bound	53,048	61,000	,870
Produkttyp * Meeszeitpunkt	Sphericity Assumed	32,661	1	32,661
	Greenhouse-Geisser	32,661	1,000	32,661
	Huynh-Feldt	32,661	1,000	32,661
	Lower-bound	32,661	1,000	32,661
Error (Produkttyp*Meeszeitpunkt)	Sphericity Assumed	76,839	61	1,260
	Greenhouse-Geisser	76,839	61,000	1,260
	Huynh-Feldt	76,839	61,000	1,260
	Lower-bound	76,839	61,000	1,260

Tests of Within-Subjects Effects

Measure: MEASURE_1

Source		F	Sig.	Partial Eta Squared
Produkttyp	Sphericity Assumed	11,846	,001	,163
	Greenhouse-Geisser	11,846	,001	,163
	Huynh-Feldt	11,846	,001	,163
	Lower-bound	11,846	,001	,163
Error(Produkttyp)	Sphericity Assumed			
	Greenhouse-Geisser			
	Huynh-Feldt			
	Lower-bound			
Meeszeitpunkt	Sphericity Assumed	32,716	,000	,349
	Greenhouse-Geisser	32,716	,000	,349
	Huynh-Feldt	32,716	,000	,349
	Lower-bound	32,716	,000	,349
Error(Meeszeitpunkt)	Sphericity Assumed			
	Greenhouse-Geisser			
	Huynh-Feldt			
	Lower-bound			
Produkttyp * Meeszeitpunkt	Sphericity Assumed	25,929	,000	,298
	Greenhouse-Geisser	25,929	,000	,298
	Huynh-Feldt	25,929	,000	,298
	Lower-bound	25,929	,000	,298
Error (Produkttyp*Meeszeitpunkt)	Sphericity Assumed			
	Greenhouse-Geisser			
	Huynh-Feldt			
	Lower-bound			

Tests of Within-Subjects Contrasts

Measure: MEASURE_1

Source	Produkttyp	Meeszeitpunkt	Type III Sum of Squares	df	Mean Square
Produkttyp	Linear		19,758	1	19,758
Error(Produkttyp)	Linear		101,742	61	1,668
Meeszeitpunkt		Linear	28,452	1	28,452
Error(Meeszeitpunkt)		Linear	53,048	61	,870
Produkttyp * Meeszeitpunkt	Linear	Linear	32,661	1	32,661
Error (Produkttyp*Meeszeitpunkt)	Linear	Linear	76,839	61	1,260

Tests of Within-Subjects Contrasts

Measure: MEASURE_1

Source	Produkttyp	Meeszeitpunkt	F	Sig.	Partial Eta Squared
Produkttyp	Linear		11,846	,001	,163
Error(Produkttyp)	Linear				
Meeszeitpunkt		Linear	32,716	,000	,349
Error(Meeszeitpunkt)		Linear			
Produkttyp * Meeszeitpunkt	Linear	Linear	25,929	,000	,298
Error (Produkttyp*Meeszeitpunkt)	Linear	Linear			

Tests of Between-Subjects Effects

Measure: MEASURE_1

Transformed Variable: Average

Source	Type III Sum of Squares	df	Mean Square	F	Sig.	Partial Eta Squared
Intercept	5845,226	1	5845,226	2916,059	,000	,980
Error	122,274	61	2,004			

Estimated Marginal Means

Produkttyp

Measure: MEASURE_1

Produkttyp	Mean	Std. Error	95% Confidence Interval	
			Lower Bound	Upper Bound
1	5,137	,113	4,910	5,364
2	4,573	,129	4,314	4,832

```
GLM Sensorik4Söbbeke27.11.12 Sensorik4Söbbeke09.01.13
   Sensorik4Landliebe27.11.12 Sensorik4Landliebe09.01.13
  /WSFACTOR=Produkttyp 2 Polynomial Meeszeitpunkt 2 Polynomial
  /METHOD=SSTYPE(3)
  /EMMEANS=TABLES(Produkttyp)
  /PRINT=DESCRIPTIVE ETASQ
  /CRITERIA=ALPHA(.05)
  /WSDESIGN=Produkttyp Meeszeitpunkt Produkttyp*Meeszeitpunkt.
```

General Linear Model

Notes

Output Created		11-NOV-2013 15:40:56
Comments		
Input	Data	C:\Documents and Settings\Dennis Boywitt\My Documents\My Dropbox\Freiberufliche Tätigkeit\Forschungsring\Daten\Sensorik_Gruppe_1_restructured.sav
	Active Dataset	DataSet2
	Filter	<none>
	Weight	<none>
	Split File	<none>
	N of Rows in Working Data File	65
Missing Value Handling	Definition of Missing	User-defined missing values are treated as missing.
	Cases Used	Statistics are based on all cases with valid data for all variables in the model.
Syntax		GLM Sensorik4Söbbeke27.11.12 Sensorik4Söbbeke09.01.13 Sensorik4Landliebe27.11.12 Sensorik4Landliebe09.01.13 /WSFACTOR=Produkttyp 2 Polynomial Meeszeitpunkt 2 Polynomial /METHOD=SSTYPE(3) /EMMEANS=TABLES(Produkttyp) /PRINT=DESCRIPTIVE ETASQ /CRITERIA=ALPHA(.05) /WSDESIGN=Produkttyp Meeszeitpunkt Produkttyp*Meeszeitpunkt.
Resources	Processor Time	00:00:00,02
	Elapsed Time	00:00:00,03

```
[DataSet2] C:\Documents and Settings\Dennis Boywitt\My Documents\My Dropbox\Freiberufliche Tätigkeit\Forschungsring\Daten\Sensorik_Gruppe_1_restructured.sav
```

Within-Subjects Factors

Measure: MEASURE_1

Produkttyp	Meeszeitpunkt	Dependent Variable
1	1	Sensorik4Söbbeke27.11.12
	2	Sensorik4Söbbeke09.01.13
2	1	Sensorik4Landliebe27.11.12
	2	Sensorik4Landliebe09.01.13

Descriptive Statistics

	Mean	Std. Deviation	N
Sensorik4Söbbeke27.11.12	5,23	1,047	62
Sensorik4Söbbeke09.01.13	5,23	,913	62
Sensorik4Landliebe27.11.12	5,31	1,080	62
Sensorik4Landliebe09.01.13	4,53	1,183	62

Multivariate Tests[a]

Effect		Value	F	Hypothesis df	Error df
Produkttyp	Pillai's Trace	,073	4,821[b]	1,000	61,000
	Wilks' Lambda	,927	4,821[b]	1,000	61,000
	Hotelling's Trace	,079	4,821[b]	1,000	61,000
	Roy's Largest Root	,079	4,821[b]	1,000	61,000
Meeszeitpunkt	Pillai's Trace	,229	18,158[b]	1,000	61,000
	Wilks' Lambda	,771	18,158[b]	1,000	61,000
	Hotelling's Trace	,298	18,158[b]	1,000	61,000
	Roy's Largest Root	,298	18,158[b]	1,000	61,000
Produkttyp * Meeszeitpunkt	Pillai's Trace	,170	12,535[b]	1,000	61,000
	Wilks' Lambda	,830	12,535[b]	1,000	61,000
	Hotelling's Trace	,205	12,535[b]	1,000	61,000
	Roy's Largest Root	,205	12,535[b]	1,000	61,000

Multivariate Tests[a]

Effect		Sig.	Partial Eta Squared
Produkttyp	Pillai's Trace	,032	,073
	Wilks' Lambda	,032	,073
	Hotelling's Trace	,032	,073
	Roy's Largest Root	,032	,073
Meeszeitpunkt	Pillai's Trace	,000	,229
	Wilks' Lambda	,000	,229
	Hotelling's Trace	,000	,229
	Roy's Largest Root	,000	,229
Produkttyp * Meeszeitpunkt	Pillai's Trace	,001	,170
	Wilks' Lambda	,001	,170
	Hotelling's Trace	,001	,170
	Roy's Largest Root	,001	,170

a. Design: Intercept
Within Subjects Design: Produkttyp + Meeszeitpunkt + Produkttyp * Meeszeitpunkt

b. Exact statistic

Mauchly's Test of Sphericity[a]

Measure: MEASURE_1

Within Subjects Effect	Mauchly's W	Approx. Chi-Square	df	Sig.	Epsilon[b]
					Greenhouse-Geisser
Produkttyp	1,000	,000	0	.	1,000
Meeszeitpunkt	1,000	,000	0	.	1,000
Produkttyp * Meeszeitpunkt	1,000	,000	0	.	1,000

Mauchly's Test of Sphericity[a]

Measure: MEASURE_1

Within Subjects Effect	Epsilon[b]	
	Huynh-Feldt	Lower-bound
Produkttyp	1,000	1,000
Meeszeitpunkt	1,000	1,000
Produkttyp * Meeszeitpunkt	1,000	1,000

Tests the null hypothesis that the error covariance matrix of the orthonormalized transformed dependent variables is proportional to an identity matrix.

a. Design: Intercept
Within Subjects Design: Produkttyp + Meeszeitpunkt + Produkttyp * Meeszeitpunkt

b. May be used to adjust the degrees of freedom for the averaged tests of significance. Corrected tests are displayed in the Tests of Within-Subjects Effects table.

Tests of Within-Subjects Effects

Measure: MEASURE_1

Source		Type III Sum of Squares	df	Mean Square
Produkttyp	Sphericity Assumed	5,823	1	5,823
	Greenhouse-Geisser	5,823	1,000	5,823
	Huynh-Feldt	5,823	1,000	5,823
	Lower-bound	5,823	1,000	5,823
Error(Produkttyp)	Sphericity Assumed	73,677	61	1,208
	Greenhouse-Geisser	73,677	61,000	1,208
	Huynh-Feldt	73,677	61,000	1,208
	Lower-bound	73,677	61,000	1,208
Meeszeitpunkt	Sphericity Assumed	9,290	1	9,290
	Greenhouse-Geisser	9,290	1,000	9,290
	Huynh-Feldt	9,290	1,000	9,290
	Lower-bound	9,290	1,000	9,290
Error(Meeszeitpunkt)	Sphericity Assumed	31,210	61	,512
	Greenhouse-Geisser	31,210	61,000	,512
	Huynh-Feldt	31,210	61,000	,512
	Lower-bound	31,210	61,000	,512
Produkttyp * Meeszeitpunkt	Sphericity Assumed	9,290	1	9,290
	Greenhouse-Geisser	9,290	1,000	9,290
	Huynh-Feldt	9,290	1,000	9,290
	Lower-bound	9,290	1,000	9,290
Error (Produkttyp*Meeszeitpunkt)	Sphericity Assumed	45,210	61	,741
	Greenhouse-Geisser	45,210	61,000	,741
	Huynh-Feldt	45,210	61,000	,741
	Lower-bound	45,210	61,000	,741

Tests of Within-Subjects Effects

Measure: MEASURE_1

Source		F	Sig.	Partial Eta Squared
Produkttyp	Sphericity Assumed	4,821	,032	,073
	Greenhouse-Geisser	4,821	,032	,073
	Huynh-Feldt	4,821	,032	,073
	Lower-bound	4,821	,032	,073
Error(Produkttyp)	Sphericity Assumed			
	Greenhouse-Geisser			
	Huynh-Feldt			
	Lower-bound			
Meeszeitpunkt	Sphericity Assumed	18,158	,000	,229
	Greenhouse-Geisser	18,158	,000	,229
	Huynh-Feldt	18,158	,000	,229
	Lower-bound	18,158	,000	,229
Error(Meeszeitpunkt)	Sphericity Assumed			
	Greenhouse-Geisser			
	Huynh-Feldt			
	Lower-bound			
Produkttyp * Meeszeitpunkt	Sphericity Assumed	12,535	,001	,170
	Greenhouse-Geisser	12,535	,001	,170
	Huynh-Feldt	12,535	,001	,170
	Lower-bound	12,535	,001	,170
Error (Produkttyp*Meeszeitpunkt)	Sphericity Assumed			
	Greenhouse-Geisser			
	Huynh-Feldt			
	Lower-bound			

Tests of Within-Subjects Contrasts

Measure: MEASURE_1

Source	Produkttyp	Meeszeitpunkt	Type III Sum of Squares	df	Mean Square
Produkttyp	Linear		5,823	1	5,823
Error(Produkttyp)	Linear		73,677	61	1,208
Meeszeitpunkt		Linear	9,290	1	9,290
Error(Meeszeitpunkt)		Linear	31,210	61	,512
Produkttyp * Meeszeitpunkt	Linear	Linear	9,290	1	9,290
Error (Produkttyp*Meeszeitpunkt)	Linear	Linear	45,210	61	,741

Tests of Within-Subjects Contrasts

Measure: MEASURE_1

Source	Produkttyp	Meeszeitpunkt	F	Sig.	Partial Eta Squared
Produkttyp	Linear		4,821	,032	,073
Error(Produkttyp)	Linear				
Meeszeitpunkt		Linear	18,158	,000	,229
Error(Meeszeitpunkt)		Linear			
Produkttyp * Meeszeitpunkt	Linear	Linear	12,535	,001	,170
Error (Produkttyp*Meeszeitpunkt)	Linear	Linear			

Tests of Between-Subjects Effects

Measure: MEASURE_1

Transformed Variable: Average

Source	Type III Sum of Squares	df	Mean Square	F	Sig.	Partial Eta Squared
Intercept	6381,306	1	6381,306	3134,299	,000	,981
Error	124,194	61	2,036			

Estimated Marginal Means

Produkttyp

Measure: MEASURE_1

Produkttyp	Mean	Std. Error	95% Confidence Interval	
			Lower Bound	Upper Bound
1	5,226	,100	5,026	5,426
2	4,919	,127	4,665	5,174

```
GLM Sensorik5Söbbeke27.11.12 Sensorik5Söbbeke09.01.13
   Sensorik5Landliebe27.11.12 Sensorik5Landliebe09.01.13
  /WSFACTOR=Produkttyp 2 Polynomial Meeszeitpunkt 2 Polynomial
  /METHOD=SSTYPE(3)
  /EMMEANS=TABLES(Produkttyp)
  /PRINT=DESCRIPTIVE ETASQ
  /CRITERIA=ALPHA(.05)
  /WSDESIGN=Produkttyp Meeszeitpunkt Produkttyp*Meeszeitpunkt.
```

General Linear Model

Notes

Output Created		11-NOV-2013 15:43:33
Comments		
Input	Data	C:\Documents and Settings\Dennis Boywitt\My Documents\My Dropbox\Freiberufliche Tätigkeit\Forschungsring\Daten\Sensorik_Gruppe_1_restructured.sav
	Active Dataset	DataSet2
	Filter	<none>
	Weight	<none>
	Split File	<none>
	N of Rows in Working Data File	65
Missing Value Handling	Definition of Missing	User-defined missing values are treated as missing.
	Cases Used	Statistics are based on all cases with valid data for all variables in the model.
Syntax		GLM Sensorik5Söbbeke27.11.12 Sensorik5Söbbeke09.01.13 Sensorik5Landliebe27.11.12 Sensorik5Landliebe09.01.13<br>/WSFACTOR=Produkttyp 2 Polynomial Meeszeitpunkt 2 Polynomial<br>/METHOD=SSTYPE(3)<br>/EMMEANS=TABLES(Produkttyp)<br>/PRINT=DESCRIPTIVE ETASQ<br>/CRITERIA=ALPHA(.05)<br>/WSDESIGN=Produkttyp Meeszeitpunkt Produkttyp*Meeszeitpunkt.
Resources	Processor Time	00:00:00,03
	Elapsed Time	00:00:00,03

```
[DataSet2] C:\Documents and Settings\Dennis Boywitt\My Documents\My Dropbo
x\Freiberufliche Tätigkeit\Forschungsring\Daten\Sensorik_Gruppe_1_restruct
ured.sav
```

Within-Subjects Factors

Measure: MEASURE_1

Produkttyp	Meeszeitpunkt	Dependent Variable
1	1	Sensorik5Söbbeke27.11.12
	2	Sensorik5Söbbeke09.01.13
2	1	Sensorik5Landliebe27.11.12
	2	Sensorik5Landliebe09.01.13

Descriptive Statistics

	Mean	Std. Deviation	N
Sensorik5Söbbeke27.11.12	5,05	,965	62
Sensorik5Söbbeke09.01.13	5,16	,978	62
Sensorik5Landliebe27.11.12	5,29	1,136	62
Sensorik5Landliebe09.01.13	4,13	1,337	62

Multivariate Tests[a]

Effect		Value	F	Hypothesis df	Error df
Produkttyp	Pillai's Trace	,103	6,983[b]	1,000	61,000
	Wilks' Lambda	,897	6,983[b]	1,000	61,000
	Hotelling's Trace	,114	6,983[b]	1,000	61,000
	Roy's Largest Root	,114	6,983[b]	1,000	61,000
Meeszeitpunkt	Pillai's Trace	,288	24,618[b]	1,000	61,000
	Wilks' Lambda	,712	24,618[b]	1,000	61,000
	Hotelling's Trace	,404	24,618[b]	1,000	61,000
	Roy's Largest Root	,404	24,618[b]	1,000	61,000
Produkttyp * Meeszeitpunkt	Pillai's Trace	,310	27,371[b]	1,000	61,000
	Wilks' Lambda	,690	27,371[b]	1,000	61,000
	Hotelling's Trace	,449	27,371[b]	1,000	61,000
	Roy's Largest Root	,449	27,371[b]	1,000	61,000

Multivariate Tests[a]

Effect		Sig.	Partial Eta Squared
Produkttyp	Pillai's Trace	,010	,103
	Wilks' Lambda	,010	,103
	Hotelling's Trace	,010	,103
	Roy's Largest Root	,010	,103
Meeszeitpunkt	Pillai's Trace	,000	,288
	Wilks' Lambda	,000	,288
	Hotelling's Trace	,000	,288
	Roy's Largest Root	,000	,288
Produkttyp * Meeszeitpunkt	Pillai's Trace	,000	,310
	Wilks' Lambda	,000	,310
	Hotelling's Trace	,000	,310
	Roy's Largest Root	,000	,310

a. Design: Intercept
Within Subjects Design: Produkttyp + Meeszeitpunkt + Produkttyp * Meeszeitpunkt

b. Exact statistic

Mauchly's Test of Sphericity[a]

Measure: MEASURE_1

Within Subjects Effect	Mauchly's W	Approx. Chi-Square	df	Sig.	Epsilon[b]
					Greenhouse-Geisser
Produkttyp	1,000	,000	0	.	1,000
Meeszeitpunkt	1,000	,000	0	.	1,000
Produkttyp * Meeszeitpunkt	1,000	,000	0	.	1,000

Mauchly's Test of Sphericity[a]

Measure: MEASURE_1

Within Subjects Effect	Epsilon[b]	
	Huynh-Feldt	Lower-bound
Produkttyp	1,000	1,000
Meeszeitpunkt	1,000	1,000
Produkttyp * Meeszeitpunkt	1,000	1,000

Tests the null hypothesis that the error covariance matrix of the orthonormalized transformed dependent variables is proportional to an identity matrix.

a. Design: Intercept
Within Subjects Design: Produkttyp + Meeszeitpunkt + Produkttyp * Meeszeitpunkt

b. May be used to adjust the degrees of freedom for the averaged tests of significance. Corrected tests are displayed in the Tests of Within-Subjects Effects table.

Tests of Within-Subjects Effects

Measure: MEASURE_1

Source		Type III Sum of Squares	df	Mean Square
Produkttyp	Sphericity Assumed	9,681	1	9,681
	Greenhouse-Geisser	9,681	1,000	9,681
	Huynh-Feldt	9,681	1,000	9,681
	Lower-bound	9,681	1,000	9,681
Error(Produkttyp)	Sphericity Assumed	84,569	61	1,386
	Greenhouse-Geisser	84,569	61,000	1,386
	Huynh-Feldt	84,569	61,000	1,386
	Lower-bound	84,569	61,000	1,386
Meeszeitpunkt	Sphericity Assumed	17,036	1	17,036
	Greenhouse-Geisser	17,036	1,000	17,036
	Huynh-Feldt	17,036	1,000	17,036
	Lower-bound	17,036	1,000	17,036
Error(Meeszeitpunkt)	Sphericity Assumed	42,214	61	,692
	Greenhouse-Geisser	42,214	61,000	,692
	Huynh-Feldt	42,214	61,000	,692
	Lower-bound	42,214	61,000	,692
Produkttyp * Meeszeitpunkt	Sphericity Assumed	25,165	1	25,165
	Greenhouse-Geisser	25,165	1,000	25,165
	Huynh-Feldt	25,165	1,000	25,165
	Lower-bound	25,165	1,000	25,165
Error (Produkttyp*Meeszeitpunkt)	Sphericity Assumed	56,085	61	,919
	Greenhouse-Geisser	56,085	61,000	,919
	Huynh-Feldt	56,085	61,000	,919
	Lower-bound	56,085	61,000	,919

Tests of Within-Subjects Effects

Measure: MEASURE_1

Source		F	Sig.	Partial Eta Squared
Produkttyp	Sphericity Assumed	6,983	,010	,103
	Greenhouse-Geisser	6,983	,010	,103
	Huynh-Feldt	6,983	,010	,103
	Lower-bound	6,983	,010	,103
Error(Produkttyp)	Sphericity Assumed			
	Greenhouse-Geisser			
	Huynh-Feldt			
	Lower-bound			
Meeszeitpunkt	Sphericity Assumed	24,618	,000	,288
	Greenhouse-Geisser	24,618	,000	,288
	Huynh-Feldt	24,618	,000	,288
	Lower-bound	24,618	,000	,288
Error(Meeszeitpunkt)	Sphericity Assumed			
	Greenhouse-Geisser			
	Huynh-Feldt			
	Lower-bound			
Produkttyp * Meeszeitpunkt	Sphericity Assumed	27,371	,000	,310
	Greenhouse-Geisser	27,371	,000	,310
	Huynh-Feldt	27,371	,000	,310
	Lower-bound	27,371	,000	,310
Error (Produkttyp*Meeszeitpunkt)	Sphericity Assumed			
	Greenhouse-Geisser			
	Huynh-Feldt			
	Lower-bound			

Tests of Within-Subjects Contrasts

Measure: MEASURE_1

Source	Produkttyp	Meeszeitpunkt	Type III Sum of Squares	df	Mean Square
Produkttyp	Linear		9,681	1	9,681
Error(Produkttyp)	Linear		84,569	61	1,386
Meeszeitpunkt		Linear	17,036	1	17,036
Error(Meeszeitpunkt)		Linear	42,214	61	,692
Produkttyp * Meeszeitpunkt	Linear	Linear	25,165	1	25,165
Error (Produkttyp*Meeszeitpunkt)	Linear	Linear	56,085	61	,919

Tests of Within-Subjects Contrasts

Measure: MEASURE_1

Source	Produkttyp	Meeszeitpunkt	F	Sig.	Partial Eta Squared
Produkttyp	Linear		6,983	,010	,103
Error(Produkttyp)	Linear				
Meeszeitpunkt		Linear	24,618	,000	,288
Error(Meeszeitpunkt)		Linear			
Produkttyp * Meeszeitpunkt	Linear	Linear	27,371	,000	,310
Error (Produkttyp*Meeszeitpunk t)	Linear	Linear			

Tests of Between-Subjects Effects

Measure: MEASURE_1

Transformed Variable: Average

Source	Type III Sum of Squares	df	Mean Square	F	Sig.	Partial Eta Squared
Intercept	5972,133	1	5972,133	3032,879	,000	,980
Error	120,117	61	1,969			

Estimated Marginal Means

Produkttyp

Measure: MEASURE_1

Produkttyp	Mean	Std. Error	95% Confidence Interval	
			Lower Bound	Upper Bound
1	5,105	,104	4,896	5,313
2	4,710	,127	4,455	4,964
