# Supplementary material for: Development and Application of a Test for Food-Induced Emotions
Source: PLoS One. 2016 Nov 18;11(11):e0165991. doi: 10.1371/journal.pone.0165991 (PMC5115674; doi:10.1371/journal.pone.0165991)
Supplement: S11 File — (PDF) [file pone.0165991.s014.pdf]

```
GLM Sensorik1Naturastar28.11.12 Sensorik1Naturastar10.01.13
   Sensorik1Goldblume27.11.12 Sensorik1Goldblume10.01.13
  /WSFACTOR=Produkttyp 2 Polynomial Meeszeitpunkt 2 Polynomial
  /METHOD=SSTYPE(3)
  /EMMEANS=TABLES(Produkttyp)
  /PRINT=DESCRIPTIVE ETASQ
  /CRITERIA=ALPHA(.05)
  /WSDESIGN=Produkttyp Meeszeitpunkt Produkttyp*Meeszeitpunkt.
```

General Linear Model

Notes

Output Created		11-NOV-2013 15:59:48
Comments		
Input	Data	C:\Documents and Settings\Dennis Boywitt\My Documents\My Dropbox\Freiberufliche Tätigkeit\Forschungsring\Daten\Sensorik_Gruppe_1_restructured.sav
	Active Dataset	DataSet2
	Filter	<none>
	Weight	<none>
	Split File	<none>
	N of Rows in Working Data File	65
Missing Value Handling	Definition of Missing	User-defined missing values are treated as missing.
	Cases Used	Statistics are based on all cases with valid data for all variables in the model.

Notes

Syntax		GLM<br>Sensorik1Naturastar28.<br>11.12<br>Sensorik1Naturastar10.<br>01.13<br>Sensorik1Goldblume27.<br>11.12<br>Sensorik1Goldblume10.<br>01.13<br>/WSFACTOR=Produkttyp<br>2 Polynomial<br>Meeszeitpunkt 2<br>Polynomial<br>/METHOD=SSTYPE(3)<br>/EMMEANS=TABLES<br>(Produkttyp)<br>/PRINT=DESCRIPTIVE<br>ETASQ<br>/CRITERIA=ALPHA(.05)<br>/WSDESIGN=Produkttyp<br>Meeszeitpunkt<br>Produkttyp*Meeszeitpunkt.
Resources	Processor Time	00:00:00,02
	Elapsed Time	00:00:00,03

[DataSet2] C:\Documents and Settings\Dennis Boywitt\My Documents\My Dropbox\Freiberufliche Tätigkeit\Forschungsring\Daten\Sensorik_Gruppe_1_restructured.sav

Within-Subjects Factors

Measure: MEASURE_1

Produkttyp	Meeszeitpunkt	Dependent Variable
1	1	Sensorik1Naturastar28.11.12
	2	Sensorik1Naturastar10.01.13
2	1	Sensorik1Goldblume27.11.12
	2	Sensorik1Goldblume10.01.13

Descriptive Statistics

	Mean	Std. Deviation	N
Sensorik1Naturastar28.11.12	4,32	1,216	63
Sensorik1Naturastar10.01.13	4,57	1,146	63
Sensorik1Goldblume27.11.12	4,33	1,218	63
Sensorik1Goldblume10.01.13	4,11	1,064	63

Multivariate Tests[a]

Effect		Value	F	Hypothesis df	Error df
Produkttyp	Pillai's Trace	,173	12,955[b]	1,000	62,000
	Wilks' Lambda	,827	12,955[b]	1,000	62,000
	Hotelling's Trace	,209	12,955[b]	1,000	62,000
	Roy's Largest Root	,209	12,955[b]	1,000	62,000
Meeszeitpunkt	Pillai's Trace	,000	,019[b]	1,000	62,000
	Wilks' Lambda	1,000	,019[b]	1,000	62,000
	Hotelling's Trace	,000	,019[b]	1,000	62,000
	Roy's Largest Root	,000	,019[b]	1,000	62,000
Produkttyp * Meeszeitpunkt	Pillai's Trace	,198	15,347[b]	1,000	62,000
	Wilks' Lambda	,802	15,347[b]	1,000	62,000
	Hotelling's Trace	,248	15,347[b]	1,000	62,000
	Roy's Largest Root	,248	15,347[b]	1,000	62,000

Multivariate Tests[a]

Effect		Sig.	Partial Eta Squared
Produkttyp	Pillai's Trace	,001	,173
	Wilks' Lambda	,001	,173
	Hotelling's Trace	,001	,173
	Roy's Largest Root	,001	,173
Meeszeitpunkt	Pillai's Trace	,892	,000
	Wilks' Lambda	,892	,000
	Hotelling's Trace	,892	,000
	Roy's Largest Root	,892	,000
Produkttyp * Meeszeitpunkt	Pillai's Trace	,000	,198
	Wilks' Lambda	,000	,198
	Hotelling's Trace	,000	,198
	Roy's Largest Root	,000	,198

a. Design: Intercept
Within Subjects Design: Produkttyp + Meeszeitpunkt + Produkttyp * Meeszeitpunkt

b. Exact statistic

Mauchly's Test of Sphericity[a]

Measure: MEASURE_1

Within Subjects Effect	Mauchly's W	Approx. Chi-Square	df	Sig.	Epsilon[b] Greenhouse-Geisser
Produkttyp	1,000	,000	0	.	1,000
Meeszeitpunkt	1,000	,000	0	.	1,000
Produkttyp * Meeszeitpunkt	1,000	,000	0	.	1,000

Mauchly's Test of Sphericity[a]

Measure: MEASURE_1

Within Subjects Effect	Epsilon[b]	
	Huynh-Feldt	Lower-bound
Produkttyp	1,000	1,000
Meeszeitpunkt	1,000	1,000
Produkttyp * Meeszeitpunkt	1,000	1,000

Tests the null hypothesis that the error covariance matrix of the orthonormalized transformed dependent variables is proportional to an identity matrix.

a. Design: Intercept
Within Subjects Design: Produkttyp + Meeszeitpunkt + Produkttyp * Meeszeitpunkt

b. May be used to adjust the degrees of freedom for the averaged tests of significance. Corrected tests are displayed in the Tests of Within-Subjects Effects table.

Tests of Within-Subjects Effects

Measure: MEASURE_1

Source		Type III Sum of Squares	df	Mean Square
Produkttyp	Sphericity Assumed	3,111	1	3,111
	Greenhouse-Geisser	3,111	1,000	3,111
	Huynh-Feldt	3,111	1,000	3,111
	Lower-bound	3,111	1,000	3,111
Error(Produkttyp)	Sphericity Assumed	14,889	62	,240
	Greenhouse-Geisser	14,889	62,000	,240
	Huynh-Feldt	14,889	62,000	,240
	Lower-bound	14,889	62,000	,240
Meeszeitpunkt	Sphericity Assumed	,016	1	,016
	Greenhouse-Geisser	,016	1,000	,016
	Huynh-Feldt	,016	1,000	,016
	Lower-bound	,016	1,000	,016
Error(Meeszeitpunkt)	Sphericity Assumed	52,984	62	,855
	Greenhouse-Geisser	52,984	62,000	,855
	Huynh-Feldt	52,984	62,000	,855
	Lower-bound	52,984	62,000	,855
Produkttyp * Meeszeitpunkt	Sphericity Assumed	3,571	1	3,571
	Greenhouse-Geisser	3,571	1,000	3,571
	Huynh-Feldt	3,571	1,000	3,571
	Lower-bound	3,571	1,000	3,571
Error (Produkttyp*Meeszeitpunkt)	Sphericity Assumed	14,429	62	,233
	Greenhouse-Geisser	14,429	62,000	,233
	Huynh-Feldt	14,429	62,000	,233
	Lower-bound	14,429	62,000	,233

Tests of Within-Subjects Effects

Measure: MEASURE_1

Source		F	Sig.	Partial Eta Squared
Produkttyp	Sphericity Assumed	12,955	,001	,173
	Greenhouse-Geisser	12,955	,001	,173
	Huynh-Feldt	12,955	,001	,173
	Lower-bound	12,955	,001	,173
Error(Produkttyp)	Sphericity Assumed			
	Greenhouse-Geisser			
	Huynh-Feldt			
	Lower-bound			
Meeszeitpunkt	Sphericity Assumed	,019	,892	,000
	Greenhouse-Geisser	,019	,892	,000
	Huynh-Feldt	,019	,892	,000
	Lower-bound	,019	,892	,000
Error(Meeszeitpunkt)	Sphericity Assumed			
	Greenhouse-Geisser			
	Huynh-Feldt			
	Lower-bound			
Produkttyp * Meeszeitpunkt	Sphericity Assumed	15,347	,000	,198
	Greenhouse-Geisser	15,347	,000	,198
	Huynh-Feldt	15,347	,000	,198
	Lower-bound	15,347	,000	,198
Error (Produkttyp*Meeszeitpunkt)	Sphericity Assumed			
	Greenhouse-Geisser			
	Huynh-Feldt			
	Lower-bound			

Tests of Within-Subjects Contrasts

Measure: MEASURE_1

Source	Produkttyp	Meeszeitpunkt	Type III Sum of Squares	df	Mean Square
Produkttyp	Linear		3,111	1	3,111
Error(Produkttyp)	Linear		14,889	62	,240
Meeszeitpunkt		Linear	,016	1	,016
Error(Meeszeitpunkt)		Linear	52,984	62	,855
Produkttyp * Meeszeitpunkt	Linear	Linear	3,571	1	3,571
Error (Produkttyp*Meeszeitpunkt)	Linear	Linear	14,429	62	,233

Tests of Within-Subjects Contrasts

Measure: MEASURE_1

Source	Produkttyp	Meeszeitpunkt	F	Sig.	Partial Eta Squared
Produkttyp	Linear		12,955	,001	,173
Error(Produkttyp)	Linear				
Meeszeitpunkt		Linear	,019	,892	,000
Error(Meeszeitpunkt)		Linear			
Produkttyp * Meeszeitpunkt	Linear	Linear	15,347	,000	,198
Error (Produkttyp*Meeszeitpunkt)	Linear	Linear			

Tests of Between-Subjects Effects

Measure: MEASURE_1

Transformed Variable: Average

Source	Type III Sum of Squares	df	Mean Square	F	Sig.	Partial Eta Squared
Intercept	4732,000	1	4732,000	1159,621	,000	,949
Error	253,000	62	4,081			

Estimated Marginal Means

Produkttyp

Measure: MEASURE_1

Produkttyp	Mean	Std. Error	95% Confidence Interval	
			Lower Bound	Upper Bound
1	4,444	,133	4,178	4,711
2	4,222	,128	3,966	4,479

```
GLM Sensorik2Naturastar28.11.12 Sensorik2Naturastar10.01.13
   Sensorik2Goldblume27.11.12 Sensorik2Goldblume10.01.13
 /WSFACTOR=Produkttyp 2 Polynomial Meeszeitpunkt 2 Polynomial
 /METHOD=SSTYPE(3)
 /EMMEANS=TABLES(Produkttyp)
 /PRINT=DESCRIPTIVE ETASQ
 /CRITERIA=ALPHA(.05)
 /WSDESIGN=Produkttyp Meeszeitpunkt Produkttyp*Meeszeitpunkt.
```

General Linear Model

Notes

Output Created		11-NOV-2013 16:01:57
Comments		
Input	Data	C:\Documents and Settings\Dennis Boywitt\My Documents\My Dropbox\Freiberufliche Tätigkeit\Forschungsring\Daten\Sensorik_Gruppe_1_restructured.sav
	Active Dataset	DataSet2
	Filter	<none>
	Weight	<none>
	Split File	<none>
	N of Rows in Working Data File	65
Missing Value Handling	Definition of Missing	User-defined missing values are treated as missing.
	Cases Used	Statistics are based on all cases with valid data for all variables in the model.
Syntax		GLM Sensorik2Naturastar28.11.12 Sensorik2Naturastar10.01.13 Sensorik2Goldblume27.11.12 Sensorik2Goldblume10.01.13<br>/WSFACTOR=Produkttyp 2 Polynomial Meeszeitpunkt 2 Polynomial<br>/METHOD=SSTYPE(3)<br>/EMMEANS=TABLES(Produkttyp)<br>/PRINT=DESCRIPTIVE ETASQ<br>/CRITERIA=ALPHA(.05)<br>/WSDESIGN=Produkttyp Meeszeitpunkt Produkttyp*Meeszeitpunkt.
Resources	Processor Time	00:00:00,02
	Elapsed Time	00:00:00,05

```
[DataSet2] C:\Documents and Settings\Dennis Boywitt\My Documents\My Dropbox\Freiberufliche Tätigkeit\Forschungsring\Daten\Sensorik_Gruppe_1_restructured.sav
```

Within-Subjects Factors

Measure: MEASURE_1

Produkttyp	Meeszeitpunkt	Dependent Variable
1	1	Sensorik2Naturastar28.11.12
	2	Sensorik2Naturastar10.01.13
2	1	Sensorik2Goldblume27.11.12
	2	Sensorik2Goldblume10.01.13

Descriptive Statistics

	Mean	Std. Deviation	N
Sensorik2Naturastar28.11.12	4,92	1,060	62
Sensorik2Naturastar10.01.13	4,63	1,090	62
Sensorik2Goldblume27.11.12	4,71	1,092	62
Sensorik2Goldblume10.01.13	4,45	,899	62

Multivariate Tests[a]

Effect		Value	F	Hypothesis df	Error df
Produkttyp	Pillai's Trace	,126	8,758[b]	1,000	61,000
	Wilks' Lambda	,874	8,758[b]	1,000	61,000
	Hotelling's Trace	,144	8,758[b]	1,000	61,000
	Roy's Largest Root	,144	8,758[b]	1,000	61,000
Meeszeitpunkt	Pillai's Trace	,128	8,931[b]	1,000	61,000
	Wilks' Lambda	,872	8,931[b]	1,000	61,000
	Hotelling's Trace	,146	8,931[b]	1,000	61,000
	Roy's Largest Root	,146	8,931[b]	1,000	61,000
Produkttyp * Meeszeitpunkt	Pillai's Trace	,000	,025[b]	1,000	61,000
	Wilks' Lambda	1,000	,025[b]	1,000	61,000
	Hotelling's Trace	,000	,025[b]	1,000	61,000
	Roy's Largest Root	,000	,025[b]	1,000	61,000

Multivariate Tests[a]

Effect		Sig.	Partial Eta Squared
Produkttyp	Pillai's Trace	,004	,126
	Wilks' Lambda	,004	,126
	Hotelling's Trace	,004	,126
	Roy's Largest Root	,004	,126
Meeszeitpunkt	Pillai's Trace	,004	,128
	Wilks' Lambda	,004	,128
	Hotelling's Trace	,004	,128
	Roy's Largest Root	,004	,128
Produkttyp * Meeszeitpunkt	Pillai's Trace	,875	,000
	Wilks' Lambda	,875	,000
	Hotelling's Trace	,875	,000
	Roy's Largest Root	,875	,000

a. Design: Intercept
Within Subjects Design: Produkttyp + Meeszeitpunkt + Produkttyp * Meeszeitpunkt

b. Exact statistic

Mauchly's Test of Sphericity[a]

Measure: MEASURE_1

Within Subjects Effect	Mauchly's W	Approx. Chi-Square	df	Sig.	Epsilon[b]
					Greenhouse-Geisser
Produkttyp	1,000	,000	0	.	1,000
Meeszeitpunkt	1,000	,000	0	.	1,000
Produkttyp * Meeszeitpunkt	1,000	,000	0	.	1,000

Mauchly's Test of Sphericity[a]

Measure: MEASURE_1

Within Subjects Effect	Epsilon[b]	
	Huynh-Feldt	Lower-bound
Produkttyp	1,000	1,000
Meeszeitpunkt	1,000	1,000
Produkttyp * Meeszeitpunkt	1,000	1,000

Tests the null hypothesis that the error covariance matrix of the orthonormalized transformed dependent variables is proportional to an identity matrix.

a. Design: Intercept
Within Subjects Design: Produkttyp + Meeszeitpunkt + Produkttyp * Meeszeitpunkt

b. May be used to adjust the degrees of freedom for the averaged tests of significance. Corrected tests are displayed in the Tests of Within-Subjects Effects table.

Tests of Within-Subjects Effects

Measure: MEASURE_1

Source		Type III Sum of Squares	df	Mean Square
Produkttyp	Sphericity Assumed	2,323	1	2,323
	Greenhouse-Geisser	2,323	1,000	2,323
	Huynh-Feldt	2,323	1,000	2,323
	Lower-bound	2,323	1,000	2,323
Error(Produkttyp)	Sphericity Assumed	16,177	61	,265
	Greenhouse-Geisser	16,177	61,000	,265
	Huynh-Feldt	16,177	61,000	,265
	Lower-bound	16,177	61,000	,265
Meeszeitpunkt	Sphericity Assumed	4,661	1	4,661
	Greenhouse-Geisser	4,661	1,000	4,661
	Huynh-Feldt	4,661	1,000	4,661
	Lower-bound	4,661	1,000	4,661
Error(Meeszeitpunkt)	Sphericity Assumed	31,839	61	,522
	Greenhouse-Geisser	31,839	61,000	,522
	Huynh-Feldt	31,839	61,000	,522
	Lower-bound	31,839	61,000	,522
Produkttyp * Meeszeitpunkt	Sphericity Assumed	,016	1	,016
	Greenhouse-Geisser	,016	1,000	,016
	Huynh-Feldt	,016	1,000	,016
	Lower-bound	,016	1,000	,016
Error (Produkttyp*Meeszeitpunkt)	Sphericity Assumed	39,484	61	,647
	Greenhouse-Geisser	39,484	61,000	,647
	Huynh-Feldt	39,484	61,000	,647
	Lower-bound	39,484	61,000	,647

Tests of Within-Subjects Effects

Measure: MEASURE_1

Source		F	Sig.	Partial Eta Squared
Produkttyp	Sphericity Assumed	8,758	,004	,126
	Greenhouse-Geisser	8,758	,004	,126
	Huynh-Feldt	8,758	,004	,126
	Lower-bound	8,758	,004	,126
Error(Produkttyp)	Sphericity Assumed			
	Greenhouse-Geisser			
	Huynh-Feldt			
	Lower-bound			
Meeszeitpunkt	Sphericity Assumed	8,931	,004	,128
	Greenhouse-Geisser	8,931	,004	,128
	Huynh-Feldt	8,931	,004	,128
	Lower-bound	8,931	,004	,128
Error(Meeszeitpunkt)	Sphericity Assumed			
	Greenhouse-Geisser			
	Huynh-Feldt			
	Lower-bound			
Produkttyp * Meeszeitpunkt	Sphericity Assumed	,025	,875	,000
	Greenhouse-Geisser	,025	,875	,000
	Huynh-Feldt	,025	,875	,000
	Lower-bound	,025	,875	,000
Error (Produkttyp*Meeszeitpunkt)	Sphericity Assumed			
	Greenhouse-Geisser			
	Huynh-Feldt			
	Lower-bound			

Tests of Within-Subjects Contrasts

Measure: MEASURE_1

Source	Produkttyp	Meeszeitpunkt	Type III Sum of Squares	df	Mean Square
Produkttyp	Linear		2,323	1	2,323
Error(Produkttyp)	Linear		16,177	61	,265
Meeszeitpunkt		Linear	4,661	1	4,661
Error(Meeszeitpunkt)		Linear	31,839	61	,522
Produkttyp * Meeszeitpunkt	Linear	Linear	,016	1	,016
Error (Produkttyp*Meeszeitpunkt)	Linear	Linear	39,484	61	,647

Tests of Within-Subjects Contrasts

Measure: MEASURE_1

Source	Produkttyp	Meeszeitpunkt	F	Sig.	Partial Eta Squared
Produkttyp	Linear		8,758	,004	,126
Error(Produkttyp)	Linear				
Meeszeitpunkt		Linear	8,931	,004	,128
Error(Meeszeitpunkt)		Linear			
Produkttyp * Meeszeitpunkt	Linear	Linear	,025	,875	,000
Error (Produkttyp*Meeszeitpunkt)	Linear	Linear			

Tests of Between-Subjects Effects

Measure: MEASURE_1

Transformed Variable: Average

Source	Type III Sum of Squares	df	Mean Square	F	Sig.	Partial Eta Squared
Intercept	5425,806	1	5425,806	1883,815	,000	,969
Error	175,694	61	2,880			

Estimated Marginal Means

Produkttyp

Measure: MEASURE_1

Produkttyp	Mean	Std. Error	95% Confidence Interval	
			Lower Bound	Upper Bound
1	4,774	,118	4,538	5,011
2	4,581	,107	4,367	4,794

```
 *** Verlgeich Naturastar vs. Goldblume am 28.11.12 & am 10.01.13 -> Sensorik 3

GLM Sensorik3Naturastar28.11.12 Sensorik3Naturastar10.01.13
    Sensorik3Goldblume27.11.12 Sensorik3Goldblume10.01.13
  /WSFACTOR=Produkttyp 2 Polynomial Meeszeitpunkt 2 Polynomial
  /METHOD=SSTYPE(3)
  /EMMEANS=TABLES(Produkttyp)
  /PRINT=DESCRIPTIVE ETASQ
  /CRITERIA=ALPHA(.05)
  /WSDESIGN=Produkttyp Meeszeitpunkt Produkttyp*Meeszeitpunkt.
```

General Linear Model

Notes

Output Created		11-NOV-2013 16:07:29
Comments		
Input	Data	C:\Documents and Settings\Dennis Boywitt\My Documents\My Dropbox\Freiberufliche Tätigkeit\Forschungsring\Daten\Sensorik_Gruppe_1_restructured.sav
	Active Dataset	DataSet2
	Filter	<none>
	Weight	<none>
	Split File	<none>
	N of Rows in Working Data File	65
Missing Value Handling	Definition of Missing	User-defined missing values are treated as missing.
	Cases Used	Statistics are based on all cases with valid data for all variables in the model.
Syntax		GLM Sensorik3Naturastar28.11.12 Sensorik3Naturastar10.01.13 Sensorik3Goldblume27.11.12 Sensorik3Goldblume10.01.13<br>/WSFACTOR=Produkttyp 2 Polynomial Meeszeitpunkt 2 Polynomial<br>/METHOD=SSTYPE(3)<br>/EMMEANS=TABLES(Produkttyp)<br>/PRINT=DESCRIPTIVE ETASQ<br>/CRITERIA=ALPHA(.05)<br>/WSDESIGN=Produkttyp Meeszeitpunkt Produkttyp*Meeszeitpunkt.
Resources	Processor Time	00:00:00,03
	Elapsed Time	00:00:00,03

```
[DataSet2] C:\Documents and Settings\Dennis Boywitt\My Documents\My Dropbox\Freiberufliche Tätigkeit\Forschungsring\Daten\Sensorik_Gruppe_1_restructured.sav
```

Within-Subjects Factors

Measure: MEASURE_1

Produkttyp	Meeszeitpunkt	Dependent Variable
1	1	Sensorik3Naturastar28.11.12
	2	Sensorik3Naturastar10.01.13
2	1	Sensorik3Goldblume27.11.12
	2	Sensorik3Goldblume10.01.13

Descriptive Statistics

	Mean	Std. Deviation	N
Sensorik3Naturastar28.11.12	4,16	1,074	62
Sensorik3Naturastar10.01.13	4,34	1,101	62
Sensorik3Goldblume27.11.12	4,18	1,167	62
Sensorik3Goldblume10.01.13	3,97	1,008	62

Multivariate Tests[a]

Effect		Value	F	Hypothesis df	Error df
Produkttyp	Pillai's Trace	,051	3,257[b]	1,000	61,000
	Wilks' Lambda	,949	3,257[b]	1,000	61,000
	Hotelling's Trace	,053	3,257[b]	1,000	61,000
	Roy's Largest Root	,053	3,257[b]	1,000	61,000
Meeszeitpunkt	Pillai's Trace	,000	,028[b]	1,000	61,000
	Wilks' Lambda	1,000	,028[b]	1,000	61,000
	Hotelling's Trace	,000	,028[b]	1,000	61,000
	Roy's Largest Root	,000	,028[b]	1,000	61,000
Produkttyp * Meeszeitpunkt	Pillai's Trace	,069	4,544[b]	1,000	61,000
	Wilks' Lambda	,931	4,544[b]	1,000	61,000
	Hotelling's Trace	,074	4,544[b]	1,000	61,000
	Roy's Largest Root	,074	4,544[b]	1,000	61,000

Multivariate Tests[a]

Effect		Sig.	Partial Eta Squared
Produkttyp	Pillai's Trace	,076	,051
	Wilks' Lambda	,076	,051
	Hotelling's Trace	,076	,051
	Roy's Largest Root	,076	,051
Meeszeitpunkt	Pillai's Trace	,868	,000
	Wilks' Lambda	,868	,000
	Hotelling's Trace	,868	,000
	Roy's Largest Root	,868	,000
Produkttyp * Meeszeitpunkt	Pillai's Trace	,037	,069
	Wilks' Lambda	,037	,069
	Hotelling's Trace	,037	,069
	Roy's Largest Root	,037	,069

a. Design: Intercept
Within Subjects Design: Produkttyp + Meeszeitpunkt + Produkttyp * Meeszeitpunkt

b. Exact statistic

Mauchly's Test of Sphericity[a]

Measure: MEASURE_1

Within Subjects Effect	Mauchly's W	Approx. Chi-Square	df	Sig.	Epsilon[b]
					Greenhouse-Geisser
Produkttyp	1,000	,000	0	.	1,000
Meeszeitpunkt	1,000	,000	0	.	1,000
Produkttyp * Meeszeitpunkt	1,000	,000	0	.	1,000

Mauchly's Test of Sphericity[a]

Measure: MEASURE_1

Within Subjects Effect	Epsilon[b]	
	Huynh-Feldt	Lower-bound
Produkttyp	1,000	1,000
Meeszeitpunkt	1,000	1,000
Produkttyp * Meeszeitpunkt	1,000	1,000

Tests the null hypothesis that the error covariance matrix of the orthonormalized transformed dependent variables is proportional to an identity matrix.

a. Design: Intercept
Within Subjects Design: Produkttyp + Meeszeitpunkt + Produkttyp * Meeszeitpunkt

b. May be used to adjust the degrees of freedom for the averaged tests of significance. Corrected tests are displayed in the Tests of Within-Subjects Effects table.

Tests of Within-Subjects Effects

Measure: MEASURE_1

Source		Type III Sum of Squares	df	Mean Square
Produkttyp	Sphericity Assumed	1,952	1	1,952
	Greenhouse-Geisser	1,952	1,000	1,952
	Huynh-Feldt	1,952	1,000	1,952
	Lower-bound	1,952	1,000	1,952
Error(Produkttyp)	Sphericity Assumed	36,548	61	,599
	Greenhouse-Geisser	36,548	61,000	,599
	Huynh-Feldt	36,548	61,000	,599
	Lower-bound	36,548	61,000	,599
Meeszeitpunkt	Sphericity Assumed	,016	1	,016
	Greenhouse-Geisser	,016	1,000	,016
	Huynh-Feldt	,016	1,000	,016
	Lower-bound	,016	1,000	,016
Error(Meeszeitpunkt)	Sphericity Assumed	35,484	61	,582
	Greenhouse-Geisser	35,484	61,000	,582
	Huynh-Feldt	35,484	61,000	,582
	Lower-bound	35,484	61,000	,582
Produkttyp * Meeszeitpunkt	Sphericity Assumed	2,323	1	2,323
	Greenhouse-Geisser	2,323	1,000	2,323
	Huynh-Feldt	2,323	1,000	2,323
	Lower-bound	2,323	1,000	2,323
Error (Produkttyp*Meeszeitpunkt)	Sphericity Assumed	31,177	61	,511
	Greenhouse-Geisser	31,177	61,000	,511
	Huynh-Feldt	31,177	61,000	,511
	Lower-bound	31,177	61,000	,511

Tests of Within-Subjects Effects

Measure: MEASURE_1

Source		F	Sig.	Partial Eta Squared
Produkttyp	Sphericity Assumed	3,257	,076	,051
	Greenhouse-Geisser	3,257	,076	,051
	Huynh-Feldt	3,257	,076	,051
	Lower-bound	3,257	,076	,051
Error(Produkttyp)	Sphericity Assumed			
	Greenhouse-Geisser			
	Huynh-Feldt			
	Lower-bound			
Meeszeitpunkt	Sphericity Assumed	,028	,868	,000
	Greenhouse-Geisser	,028	,868	,000
	Huynh-Feldt	,028	,868	,000
	Lower-bound	,028	,868	,000
Error(Meeszeitpunkt)	Sphericity Assumed			
	Greenhouse-Geisser			
	Huynh-Feldt			
	Lower-bound			
Produkttyp * Meeszeitpunkt	Sphericity Assumed	4,544	,037	,069
	Greenhouse-Geisser	4,544	,037	,069
	Huynh-Feldt	4,544	,037	,069
	Lower-bound	4,544	,037	,069
Error (Produkttyp*Meeszeitpunkt)	Sphericity Assumed			
	Greenhouse-Geisser			
	Huynh-Feldt			
	Lower-bound			

Tests of Within-Subjects Contrasts

Measure: MEASURE_1

Source	Produkttyp	Meeszeitpunkt	Type III Sum of Squares	df	Mean Square
Produkttyp	Linear		1,952	1	1,952
Error(Produkttyp)	Linear		36,548	61	,599
Meeszeitpunkt		Linear	,016	1	,016
Error(Meeszeitpunkt)		Linear	35,484	61	,582
Produkttyp * Meeszeitpunkt	Linear	Linear	2,323	1	2,323
Error (Produkttyp*Meeszeitpunkt)	Linear	Linear	31,177	61	,511

Tests of Within-Subjects Contrasts

Measure: MEASURE_1

Source	Produkttyp	Meeszeitpunkt	F	Sig.	Partial Eta Squared
Produkttyp	Linear		3,257	,076	,051
Error(Produkttyp)	Linear				
Meeszeitpunkt		Linear	,028	,868	,000
Error(Meeszeitpunkt)		Linear			
Produkttyp * Meeszeitpunkt	Linear	Linear	4,544	,037	,069
Error (Produkttyp*Meeszeitpunkt)	Linear	Linear			

Tests of Between-Subjects Effects

Measure: MEASURE_1

Transformed Variable: Average

Source	Type III Sum of Squares	df	Mean Square	F	Sig.	Partial Eta Squared
Intercept	4294,452	1	4294,452	1408,029	,000	,958
Error	186,048	61	3,050			

Estimated Marginal Means

Produkttyp

Measure: MEASURE_1

Produkttyp	Mean	Std. Error	95% Confidence Interval	
			Lower Bound	Upper Bound
1	4,250	,124	4,003	4,497
2	4,073	,119	3,835	4,310

```
*** Verlgeich Naturastar vs. Goldblume am 28.11.12 & am 10.01.13 -> Sensorik 4

GLM Sensorik4Naturastar28.11.12 Sensorik4Naturastar10.01.13
    Sensorik4Goldblume27.11.12 Sensorik4Goldblume10.01.13
  /WSFACTOR=Produkttyp 2 Polynomial Meeszeitpunkt 2 Polynomial
  /METHOD=SSTYPE(3)
  /EMMEANS=TABLES(Produkttyp)
  /PRINT=DESCRIPTIVE ETASQ
  /CRITERIA=ALPHA(.05)
  /WSDESIGN=Produkttyp Meeszeitpunkt Produkttyp*Meeszeitpunkt.
```

General Linear Model

Notes

Output Created		11-NOV-2013 16:08:50
Comments		
Input	Data	C:\Documents and Settings\Dennis Boywitt\My Documents\My Dropbox\Freiberufliche Tätigkeit\Forschungsring\Daten\Sensorik_Gruppe_1_restructured.sav
	Active Dataset	DataSet2
	Filter	<none>
	Weight	<none>
	Split File	<none>
	N of Rows in Working Data File	65
Missing Value Handling	Definition of Missing	User-defined missing values are treated as missing.
	Cases Used	Statistics are based on all cases with valid data for all variables in the model.
Syntax		GLM Sensorik4Naturastar28.11.12 Sensorik4Naturastar10.01.13 Sensorik4Goldblume27.11.12 Sensorik4Goldblume10.01.13 /WSFACTOR=Produkttyp 2 Polynomial Meeszeitpunkt 2 Polynomial /METHOD=SSTYPE(3) /EMMEANS=TABLES(Produkttyp) /PRINT=DESCRIPTIVE ETASQ /CRITERIA=ALPHA(.05) /WSDESIGN=Produkttyp Meeszeitpunkt Produkttyp*Meeszeitpunkt.
Resources	Processor Time	00:00:00,03
	Elapsed Time	00:00:00,03

```
[DataSet2] C:\Documents and Settings\Dennis Boywitt\My Documents\My Dropbox\Freiberufliche Tätigkeit\Forschungsring\Daten\Sensorik_Gruppe_1_restructured.sav
```

Within-Subjects Factors

Measure: MEASURE_1

Produkttyp	Meeszeitpunkt	Dependent Variable
1	1	Sensorik4Naturastar28.11.12
	2	Sensorik4Naturastar10.01.13
2	1	Sensorik4Goldblume27.11.12
	2	Sensorik4Goldblume10.01.13

Descriptive Statistics

	Mean	Std. Deviation	N
Sensorik4Naturastar28.11.12	4,10	1,155	62
Sensorik4Naturastar10.01.13	4,26	1,085	62
Sensorik4Goldblume27.11.12	4,13	1,180	62
Sensorik4Goldblume10.01.13	4,02	,949	62

Multivariate Tests[a]

Effect		Value	F	Hypothesis df	Error df
Produkttyp	Pillai's Trace	,024	1,508[b]	1,000	61,000
	Wilks' Lambda	,976	1,508[b]	1,000	61,000
	Hotelling's Trace	,025	1,508[b]	1,000	61,000
	Roy's Largest Root	,025	1,508[b]	1,000	61,000
Meeszeitpunkt	Pillai's Trace	,001	,054[b]	1,000	61,000
	Wilks' Lambda	,999	,054[b]	1,000	61,000
	Hotelling's Trace	,001	,054[b]	1,000	61,000
	Roy's Largest Root	,001	,054[b]	1,000	61,000
Produkttyp * Meeszeitpunkt	Pillai's Trace	,032	2,026[b]	1,000	61,000
	Wilks' Lambda	,968	2,026[b]	1,000	61,000
	Hotelling's Trace	,033	2,026[b]	1,000	61,000
	Roy's Largest Root	,033	2,026[b]	1,000	61,000

Multivariate Tests[a]

Effect		Sig.	Partial Eta Squared
Produkttyp	Pillai's Trace	,224	,024
	Wilks' Lambda	,224	,024
	Hotelling's Trace	,224	,024
	Roy's Largest Root	,224	,024
Meeszeitpunkt	Pillai's Trace	,817	,001
	Wilks' Lambda	,817	,001
	Hotelling's Trace	,817	,001
	Roy's Largest Root	,817	,001
Produkttyp * Meeszeitpunkt	Pillai's Trace	,160	,032
	Wilks' Lambda	,160	,032
	Hotelling's Trace	,160	,032
	Roy's Largest Root	,160	,032

a. Design: Intercept
Within Subjects Design: Produkttyp + Meeszeitpunkt + Produkttyp * Meeszeitpunkt

b. Exact statistic

Mauchly's Test of Sphericity[a]

Measure: MEASURE_1

Within Subjects Effect	Mauchly's W	Approx. Chi-Square	df	Sig.	Epsilon[b]
					Greenhouse-Geisser
Produkttyp	1,000	,000	0	.	1,000
Meeszeitpunkt	1,000	,000	0	.	1,000
Produkttyp * Meeszeitpunkt	1,000	,000	0	.	1,000

Mauchly's Test of Sphericity[a]

Measure: MEASURE_1

Within Subjects Effect	Epsilon[b]	
	Huynh-Feldt	Lower-bound
Produkttyp	1,000	1,000
Meeszeitpunkt	1,000	1,000
Produkttyp * Meeszeitpunkt	1,000	1,000

Tests the null hypothesis that the error covariance matrix of the orthonormalized transformed dependent variables is proportional to an identity matrix.

a. Design: Intercept
Within Subjects Design: Produkttyp + Meeszeitpunkt + Produkttyp * Meeszeitpunkt

b. May be used to adjust the degrees of freedom for the averaged tests of significance. Corrected tests are displayed in the Tests of Within-Subjects Effects table.

Tests of Within-Subjects Effects

Measure: MEASURE_1

Source		Type III Sum of Squares	df	Mean Square
Produkttyp	Sphericity Assumed	,681	1	,681
	Greenhouse-Geisser	,681	1,000	,681
	Huynh-Feldt	,681	1,000	,681
	Lower-bound	,681	1,000	,681
Error(Produkttyp)	Sphericity Assumed	27,569	61	,452
	Greenhouse-Geisser	27,569	61,000	,452
	Huynh-Feldt	27,569	61,000	,452
	Lower-bound	27,569	61,000	,452
Meeszeitpunkt	Sphericity Assumed	,036	1	,036
	Greenhouse-Geisser	,036	1,000	,036
	Huynh-Feldt	,036	1,000	,036
	Lower-bound	,036	1,000	,036
Error(Meeszeitpunkt)	Sphericity Assumed	41,214	61	,676
	Greenhouse-Geisser	41,214	61,000	,676
	Huynh-Feldt	41,214	61,000	,676
	Lower-bound	41,214	61,000	,676
Produkttyp * Meeszeitpunkt	Sphericity Assumed	1,165	1	1,165
	Greenhouse-Geisser	1,165	1,000	1,165
	Huynh-Feldt	1,165	1,000	1,165
	Lower-bound	1,165	1,000	1,165
Error (Produkttyp*Meeszeitpunkt)	Sphericity Assumed	35,085	61	,575
	Greenhouse-Geisser	35,085	61,000	,575
	Huynh-Feldt	35,085	61,000	,575
	Lower-bound	35,085	61,000	,575

Tests of Within-Subjects Effects

Measure: MEASURE_1

Source		F	Sig.	Partial Eta Squared
Produkttyp	Sphericity Assumed	1,508	,224	,024
	Greenhouse-Geisser	1,508	,224	,024
	Huynh-Feldt	1,508	,224	,024
	Lower-bound	1,508	,224	,024
Error(Produkttyp)	Sphericity Assumed			
	Greenhouse-Geisser			
	Huynh-Feldt			
	Lower-bound			
Meeszeitpunkt	Sphericity Assumed	,054	,817	,001
	Greenhouse-Geisser	,054	,817	,001
	Huynh-Feldt	,054	,817	,001
	Lower-bound	,054	,817	,001
Error(Meeszeitpunkt)	Sphericity Assumed			
	Greenhouse-Geisser			
	Huynh-Feldt			
	Lower-bound			
Produkttyp * Meeszeitpunkt	Sphericity Assumed	2,026	,160	,032
	Greenhouse-Geisser	2,026	,160	,032
	Huynh-Feldt	2,026	,160	,032
	Lower-bound	2,026	,160	,032
Error (Produkttyp*Meeszeitpunkt)	Sphericity Assumed			
	Greenhouse-Geisser			
	Huynh-Feldt			
	Lower-bound			

Tests of Within-Subjects Contrasts

Measure: MEASURE_1

Source	Produkttyp	Meeszeitpunkt	Type III Sum of Squares	df	Mean Square
Produkttyp	Linear		,681	1	,681
Error(Produkttyp)	Linear		27,569	61	,452
Meeszeitpunkt		Linear	,036	1	,036
Error(Meeszeitpunkt)		Linear	41,214	61	,676
Produkttyp * Meeszeitpunkt	Linear	Linear	1,165	1	1,165
Error (Produkttyp*Meeszeitpunkt)	Linear	Linear	35,085	61	,575

Tests of Within-Subjects Contrasts

Measure: MEASURE_1

Source	Produkttyp	Meeszeitpunkt	F	Sig.	Partial Eta Squared
Produkttyp	Linear		1,508	,224	,024
Error(Produkttyp)	Linear				
Meeszeitpunkt		Linear	,054	,817	,001
Error(Meeszeitpunkt)		Linear			
Produkttyp * Meeszeitpunkt	Linear	Linear	2,026	,160	,032
Error (Produkttyp*Meeszeitpunkt)	Linear	Linear			

Tests of Between-Subjects Effects

Measure: MEASURE_1

Transformed Variable: Average

Source	Type III Sum of Squares	df	Mean Square	F	Sig.	Partial Eta Squared
Intercept	4219,875	1	4219,875	1359,273	,000	,957
Error	189,375	61	3,105			

Estimated Marginal Means

Produkttyp

Measure: MEASURE_1

Produkttyp	Mean	Std. Error	95% Confidence Interval	
			Lower Bound	Upper Bound
1	4,177	,121	3,936	4,419
2	4,073	,119	3,835	4,310

```
 *** Verlgeich Naturastar vs. Goldblume am 28.11.12 & am 10.01.13 -> Sensorik 5

GLM Sensorik5Naturastar28.11.12 Sensorik5Naturastar10.01.13
    Sensorik5Goldblume27.11.12 Sensorik5Goldblume10.01.13
  /WSFACTOR=Produkttyp 2 Polynomial Meeszeitpunkt 2 Polynomial
  /METHOD=SSTYPE(3)
  /EMMEANS=TABLES(Produkttyp)
  /PRINT=DESCRIPTIVE ETASQ
  /CRITERIA=ALPHA(.05)
  /WSDESIGN=Produkttyp Meeszeitpunkt Produkttyp*Meeszeitpunkt.
```

General Linear Model

Notes

Output Created		11-NOV-2013 16:09:29
Comments		
Input	Data	C:\Documents and Settings\Dennis Boywitt\My Documents\My Dropbox\Freiberufliche Tätigkeit\Forschungsring\Daten\Sensorik_Gruppe_1_restructured.sav
	Active Dataset	DataSet2
	Filter	<none>
	Weight	<none>
	Split File	<none>
	N of Rows in Working Data File	65
Missing Value Handling	Definition of Missing	User-defined missing values are treated as missing.
	Cases Used	Statistics are based on all cases with valid data for all variables in the model.
Syntax		GLM Sensorik5Naturastar28.11.12 Sensorik5Naturastar10.01.13 Sensorik5Goldblume27.11.12 Sensorik5Goldblume10.01.13 /WSFACTOR=Produkttyp 2 Polynomial Meeszeitpunkt 2 Polynomial /METHOD=SSTYPE(3) /EMMEANS=TABLES (Produkttyp) /PRINT=DESCRIPTIVE ETASQ /CRITERIA=ALPHA(.05) /WSDESIGN=Produkttyp Meeszeitpunkt Produkttyp*Meeszeitpunkt.
Resources	Processor Time	00:00:00,03
	Elapsed Time	00:00:00,05

```
[DataSet2] C:\Documents and Settings\Dennis Boywitt\My Documents\My Dropbo
x\Freiberufliche Tätigkeit\Forschungsring\Daten\Sensorik_Gruppe_1_restruct
ured.sav
```

Within-Subjects Factors

Measure: MEASURE_1

Produkttyp	Meeszeitpunkt	Dependent Variable
1	1	Sensorik5Naturastar28.11.12
	2	Sensorik5Naturastar10.01.13
2	1	Sensorik5Goldblume27.11.12
	2	Sensorik5Goldblume10.01.13

Descriptive Statistics

	Mean	Std. Deviation	N
Sensorik5Naturastar28.11.12	4,23	1,078	62
Sensorik5Naturastar10.01.13	4,31	1,049	62
Sensorik5Goldblume27.11.12	4,11	1,073	62
Sensorik5Goldblume10.01.13	4,03	,868	62

Multivariate Tests[a]

Effect		Value	F	Hypothesis df	Error df
Produkttyp	Pillai's Trace	,111	7,585[b]	1,000	61,000
	Wilks' Lambda	,889	7,585[b]	1,000	61,000
	Hotelling's Trace	,124	7,585[b]	1,000	61,000
	Roy's Largest Root	,124	7,585[b]	1,000	61,000
Meeszeitpunkt	Pillai's Trace	,000	,000[b]	1,000	61,000
	Wilks' Lambda	1,000	,000[b]	1,000	61,000
	Hotelling's Trace	,000	,000[b]	1,000	61,000
	Roy's Largest Root	,000	,000[b]	1,000	61,000
Produkttyp * Meeszeitpunkt	Pillai's Trace	,018	1,139[b]	1,000	61,000
	Wilks' Lambda	,982	1,139[b]	1,000	61,000
	Hotelling's Trace	,019	1,139[b]	1,000	61,000
	Roy's Largest Root	,019	1,139[b]	1,000	61,000

Multivariate Tests[a]

Effect		Sig.	Partial Eta Squared
Produkttyp	Pillai's Trace	,008	,111
	Wilks' Lambda	,008	,111
	Hotelling's Trace	,008	,111
	Roy's Largest Root	,008	,111
Meeszeitpunkt	Pillai's Trace	1,000	,000
	Wilks' Lambda	1,000	,000
	Hotelling's Trace	1,000	,000
	Roy's Largest Root	1,000	,000
Produkttyp * Meeszeitpunkt	Pillai's Trace	,290	,018
	Wilks' Lambda	,290	,018
	Hotelling's Trace	,290	,018
	Roy's Largest Root	,290	,018

a. Design: Intercept
Within Subjects Design: Produkttyp + Meeszeitpunkt + Produkttyp * Meeszeitpunkt

b. Exact statistic

Mauchly's Test of Sphericity[a]

Measure: MEASURE_1

Within Subjects Effect	Mauchly's W	Approx. Chi-Square	df	Sig.	Epsilon[b] Greenhouse-Geisser
Produkttyp	1,000	,000	0	.	1,000
Meeszeitpunkt	1,000	,000	0	.	1,000
Produkttyp * Meeszeitpunkt	1,000	,000	0	.	1,000

Mauchly's Test of Sphericity[a]

Measure: MEASURE_1

Within Subjects Effect	Epsilon[b]	
	Huynh-Feldt	Lower-bound
Produkttyp	1,000	1,000
Meeszeitpunkt	1,000	1,000
Produkttyp * Meeszeitpunkt	1,000	1,000

Tests the null hypothesis that the error covariance matrix of the orthonormalized transformed dependent variables is proportional to an identity matrix.

a. Design: Intercept
Within Subjects Design: Produkttyp + Meeszeitpunkt + Produkttyp * Meeszeitpunkt

b. May be used to adjust the degrees of freedom for the averaged tests of significance. Corrected tests are displayed in the Tests of Within-Subjects Effects table.

Tests of Within-Subjects Effects

Measure: MEASURE_1

Source		Type III Sum of Squares	df	Mean Square
Produkttyp	Sphericity Assumed	2,323	1	2,323
	Greenhouse-Geisser	2,323	1,000	2,323
	Huynh-Feldt	2,323	1,000	2,323
	Lower-bound	2,323	1,000	2,323
Error(Produkttyp)	Sphericity Assumed	18,677	61	,306
	Greenhouse-Geisser	18,677	61,000	,306
	Huynh-Feldt	18,677	61,000	,306
	Lower-bound	18,677	61,000	,306
Meeszeitpunkt	Sphericity Assumed	,000	1	,000
	Greenhouse-Geisser	,000	1,000	,000
	Huynh-Feldt	,000	1,000	,000
	Lower-bound	,000	1,000	,000
Error(Meeszeitpunkt)	Sphericity Assumed	24,000	61	,393
	Greenhouse-Geisser	24,000	61,000	,393
	Huynh-Feldt	24,000	61,000	,393
	Lower-bound	24,000	61,000	,393
Produkttyp * Meeszeitpunkt	Sphericity Assumed	,403	1	,403
	Greenhouse-Geisser	,403	1,000	,403
	Huynh-Feldt	,403	1,000	,403
	Lower-bound	,403	1,000	,403
Error (Produkttyp*Meeszeitpunkt)	Sphericity Assumed	21,597	61	,354
	Greenhouse-Geisser	21,597	61,000	,354
	Huynh-Feldt	21,597	61,000	,354
	Lower-bound	21,597	61,000	,354

Tests of Within-Subjects Effects

Measure: MEASURE_1

Source		F	Sig.	Partial Eta Squared
Produkttyp	Sphericity Assumed	7,585	,008	,111
	Greenhouse-Geisser	7,585	,008	,111
	Huynh-Feldt	7,585	,008	,111
	Lower-bound	7,585	,008	,111
Error(Produkttyp)	Sphericity Assumed			
	Greenhouse-Geisser			
	Huynh-Feldt			
	Lower-bound			
Meeszeitpunkt	Sphericity Assumed	,000	1,000	,000
	Greenhouse-Geisser	,000	1,000	,000
	Huynh-Feldt	,000	1,000	,000
	Lower-bound	,000	1,000	,000
Error(Meeszeitpunkt)	Sphericity Assumed			
	Greenhouse-Geisser			
	Huynh-Feldt			
	Lower-bound			
Produkttyp * Meeszeitpunkt	Sphericity Assumed	1,139	,290	,018
	Greenhouse-Geisser	1,139	,290	,018
	Huynh-Feldt	1,139	,290	,018
	Lower-bound	1,139	,290	,018
Error (Produkttyp*Meeszeitpunkt)	Sphericity Assumed			
	Greenhouse-Geisser			
	Huynh-Feldt			
	Lower-bound			

Tests of Within-Subjects Contrasts

Measure: MEASURE_1

Source	Produkttyp	Meeszeitpunkt	Type III Sum of Squares	df	Mean Square
Produkttyp	Linear		2,323	1	2,323
Error(Produkttyp)	Linear		18,677	61	,306
Meeszeitpunkt		Linear	,000	1	,000
Error(Meeszeitpunkt)		Linear	24,000	61	,393
Produkttyp * Meeszeitpunkt	Linear	Linear	,403	1	,403
Error (Produkttyp*Meeszeitpunkt)	Linear	Linear	21,597	61	,354

Tests of Within-Subjects Contrasts

Measure: MEASURE_1

Source	Produkttyp	Meeszeitpunkt	F	Sig.	Partial Eta Squared
Produkttyp	Linear		7,585	,008	,111
Error(Produkttyp)	Linear				
Meeszeitpunkt		Linear	,000	1,000	,000
Error(Meeszeitpunkt)		Linear			
Produkttyp * Meeszeitpunkt	Linear	Linear	1,139	,290	,018
Error (Produkttyp*Meeszeitpunkt)	Linear	Linear			

Tests of Between-Subjects Effects

Measure: MEASURE_1

Transformed Variable: Average

Source	Type III Sum of Squares	df	Mean Square	F	Sig.	Partial Eta Squared
Intercept	4311,113	1	4311,113	1384,917	,000	,958
Error	189,887	61	3,113			

Estimated Marginal Means

Produkttyp

Measure: MEASURE_1

Produkttyp	Mean	Std. Error	95% Confidence Interval	
			Lower Bound	Upper Bound
1	4,266	,123	4,020	4,512
2	4,073	,111	3,850	4,295
