# Supplementary material for: Development and Application of a Test for Food-Induced Emotions
Source: PLoS One. 2016 Nov 18;11(11):e0165991. doi: 10.1371/journal.pone.0165991 (PMC5115674; doi:10.1371/journal.pone.0165991)
Supplement: S12 File — (PDF) [file pone.0165991.s015.pdf]

```
 ************************
*Zucker

*** Verlgeich Guarani vs. Diamant am 28.11.12 & am 10.01.13 -> Sensorik 1

GLM Sensorik1Guarani28.11.12 Sensorik1Guarani10.01.13
    Sensorik1Diamant28.11.12 Sensorik1Diamant10.01.13
  /WSFACTOR=Produkttyp 2 Polynomial Meeszeitpunkt 2 Polynomial
  /METHOD=SSTYPE(3)
  /EMMEANS=TABLES(Produkttyp)
  /PRINT=DESCRIPTIVE ETASQ
  /CRITERIA=ALPHA(.05)
  /WSDESIGN=Produkttyp Meeszeitpunkt Produkttyp*Meeszeitpunkt.
```

General Linear Model

Notes

Output Created		11-NOV-2013 15:46:07
Comments		
Input	Data	C:\Documents and Settings\Dennis Boywitt\My Documents\My Dropbox\Freiberufliche Tätigkeit\Forschungsring\Daten\Sensorik_Gruppe_1_restructured.sav
	Active Dataset	DataSet2
	Filter	<none>
	Weight	<none>
	Split File	<none>
	N of Rows in Working Data File	65
Missing Value Handling	Definition of Missing	User-defined missing values are treated as missing.
	Cases Used	Statistics are based on all cases with valid data for all variables in the model.

Notes

Syntax		GLM Sensorik1Guarani28.11.12 Sensorik1Guarani10.01.13 Sensorik1Diamant28.11.12 Sensorik1Diamant10.01.13 /WSFACTOR=Produkttyp 2 Polynomial Meeszeitpunkt 2 Polynomial /METHOD=SSTYPE(3) /EMMEANS=TABLES(Produkttyp) /PRINT=DESCRIPTIVE ETASQ /CRITERIA=ALPHA(.05) /WSDESIGN=Produkttyp Meeszeitpunkt Produkttyp*Meeszeitpunkt.
Resources	Processor Time	00:00:00,03
	Elapsed Time	00:00:00,03

```
[DataSet2] C:\Documents and Settings\Dennis Boywitt\My Documents\My Dropbo
x\Freiberufliche Tätigkeit\Forschungsring\Daten\Sensorik_Gruppe_1_restruct
ured.sav
```

Within-Subjects Factors

Measure: MEASURE_1

Produkttyp	Meeszeitpunkt	Dependent Variable
1	1	Sensorik1Guarani28.11.12
	2	Sensorik1Guarani10.01.13
2	1	Sensorik1Diamant28.11.12
	2	Sensorik1Diamant10.01.13

Descriptive Statistics

	Mean	Std. Deviation	N
Sensorik1Guarani28.11.12	4,33	1,295	63
Sensorik1Guarani10.01.13	4,48	1,216	63
Sensorik1Diamant28.11.12	5,65	1,003	63
Sensorik1Diamant10.01.13	5,68	1,029	63

Multivariate Tests[a]

Effect		Value	F	Hypothesis df	Error df
Produkttyp	Pillai's Trace	,581	85,877[b]	1,000	62,000
	Wilks' Lambda	,419	85,877[b]	1,000	62,000
	Hotelling's Trace	1,385	85,877[b]	1,000	62,000
	Roy's Largest Root	1,385	85,877[b]	1,000	62,000
Meeszeitpunkt	Pillai's Trace	,026	1,629[b]	1,000	62,000
	Wilks' Lambda	,974	1,629[b]	1,000	62,000
	Hotelling's Trace	,026	1,629[b]	1,000	62,000
	Roy's Largest Root	,026	1,629[b]	1,000	62,000
Produkttyp * Meeszeitpunkt	Pillai's Trace	,008	,472[b]	1,000	62,000
	Wilks' Lambda	,992	,472[b]	1,000	62,000
	Hotelling's Trace	,008	,472[b]	1,000	62,000
	Roy's Largest Root	,008	,472[b]	1,000	62,000

Multivariate Tests[a]

Effect		Sig.	Partial Eta Squared
Produkttyp	Pillai's Trace	,000	,581
	Wilks' Lambda	,000	,581
	Hotelling's Trace	,000	,581
	Roy's Largest Root	,000	,581
Meeszeitpunkt	Pillai's Trace	,207	,026
	Wilks' Lambda	,207	,026
	Hotelling's Trace	,207	,026
	Roy's Largest Root	,207	,026
Produkttyp * Meeszeitpunkt	Pillai's Trace	,495	,008
	Wilks' Lambda	,495	,008
	Hotelling's Trace	,495	,008
	Roy's Largest Root	,495	,008

a. Design: Intercept
Within Subjects Design: Produkttyp + Meeszeitpunkt + Produkttyp * Meeszeitpunkt

b. Exact statistic

Mauchly's Test of Sphericity[a]

Measure: MEASURE_1

Within Subjects Effect	Mauchly's W	Approx. Chi-Square	df	Sig.	Epsilon[b] Greenhouse-Geisser
Produkttyp	1,000	,000	0	.	1,000
Meeszeitpunkt	1,000	,000	0	.	1,000
Produkttyp * Meeszeitpunkt	1,000	,000	0	.	1,000

Mauchly's Test of Sphericity[a]

Measure: MEASURE_1

Within Subjects Effect	Epsilon[b]	
	Huynh-Feldt	Lower-bound
Produkttyp	1,000	1,000
Meeszeitpunkt	1,000	1,000
Produkttyp * Meeszeitpunkt	1,000	1,000

Tests the null hypothesis that the error covariance matrix of the orthonormalized transformed dependent variables is proportional to an identity matrix.

a. Design: Intercept
Within Subjects Design: Produkttyp + Meeszeitpunkt + Produkttyp * Meeszeitpunkt

b. May be used to adjust the degrees of freedom for the averaged tests of significance. Corrected tests are displayed in the Tests of Within-Subjects Effects table.

Tests of Within-Subjects Effects

Measure: MEASURE_1

Source		Type III Sum of Squares	df	Mean Square
Produkttyp	Sphericity Assumed	100,321	1	100,321
	Greenhouse-Geisser	100,321	1,000	100,321
	Huynh-Feldt	100,321	1,000	100,321
	Lower-bound	100,321	1,000	100,321
Error(Produkttyp)	Sphericity Assumed	72,429	62	1,168
	Greenhouse-Geisser	72,429	62,000	1,168
	Huynh-Feldt	72,429	62,000	1,168
	Lower-bound	72,429	62,000	1,168
Meeszeitpunkt	Sphericity Assumed	,480	1	,480
	Greenhouse-Geisser	,480	1,000	,480
	Huynh-Feldt	,480	1,000	,480
	Lower-bound	,480	1,000	,480
Error(Meeszeitpunkt)	Sphericity Assumed	18,270	62	,295
	Greenhouse-Geisser	18,270	62,000	,295
	Huynh-Feldt	18,270	62,000	,295
	Lower-bound	18,270	62,000	,295
Produkttyp * Meeszeitpunkt	Sphericity Assumed	,194	1	,194
	Greenhouse-Geisser	,194	1,000	,194
	Huynh-Feldt	,194	1,000	,194
	Lower-bound	,194	1,000	,194
Error (Produkttyp*Meeszeitpunkt)	Sphericity Assumed	25,556	62	,412
	Greenhouse-Geisser	25,556	62,000	,412
	Huynh-Feldt	25,556	62,000	,412
	Lower-bound	25,556	62,000	,412

Tests of Within-Subjects Effects

Measure: MEASURE_1

Source		F	Sig.	Partial Eta Squared
Produkttyp	Sphericity Assumed	85,877	,000	,581
	Greenhouse-Geisser	85,877	,000	,581
	Huynh-Feldt	85,877	,000	,581
	Lower-bound	85,877	,000	,581
Error(Produkttyp)	Sphericity Assumed			
	Greenhouse-Geisser			
	Huynh-Feldt			
	Lower-bound			
Meeszeitpunkt	Sphericity Assumed	1,629	,207	,026
	Greenhouse-Geisser	1,629	,207	,026
	Huynh-Feldt	1,629	,207	,026
	Lower-bound	1,629	,207	,026
Error(Meeszeitpunkt)	Sphericity Assumed			
	Greenhouse-Geisser			
	Huynh-Feldt			
	Lower-bound			
Produkttyp * Meeszeitpunkt	Sphericity Assumed	,472	,495	,008
	Greenhouse-Geisser	,472	,495	,008
	Huynh-Feldt	,472	,495	,008
	Lower-bound	,472	,495	,008
Error (Produkttyp*Meeszeitpunkt)	Sphericity Assumed			
	Greenhouse-Geisser			
	Huynh-Feldt			
	Lower-bound			

Tests of Within-Subjects Contrasts

Measure: MEASURE_1

Source	Produkttyp	Meeszeitpunkt	Type III Sum of Squares	df	Mean Square
Produkttyp	Linear		100,321	1	100,321
Error(Produkttyp)	Linear		72,429	62	1,168
Meeszeitpunkt		Linear	,480	1	,480
Error(Meeszeitpunkt)		Linear	18,270	62	,295
Produkttyp * Meeszeitpunkt	Linear	Linear	,194	1	,194
Error (Produkttyp*Meeszeitpunkt)	Linear	Linear	25,556	62	,412

Tests of Within-Subjects Contrasts

Measure: MEASURE_1

Source	Produkttyp	Meeszeitpunkt	F	Sig.	Partial Eta Squared
Produkttyp	Linear		85,877	,000	,581
Error(Produkttyp)	Linear				
Meeszeitpunkt		Linear	1,629	,207	,026
Error(Meeszeitpunkt)		Linear			
Produkttyp * Meeszeitpunkt	Linear	Linear	,472	,495	,008
Error (Produkttyp*Meeszeitpunkt)	Linear	Linear			

Tests of Between-Subjects Effects

Measure: MEASURE_1

Transformed Variable: Average

Source	Type III Sum of Squares	df	Mean Square	F	Sig.	Partial Eta Squared
Intercept	6390,321	1	6390,321	1910,055	,000	,969
Error	207,429	62	3,346			

Estimated Marginal Means

Produkttyp

Measure: MEASURE_1

Produkttyp	Mean	Std. Error	95% Confidence Interval	
			Lower Bound	Upper Bound
1	4,405	,146	4,113	4,697
2	5,667	,120	5,426	5,907

```
GLM Sensorik2Guarani28.11.12 Sensorik2Guarani10.01.13
   Sensorik2Diamant28.11.12 Sensorik2Diamant10.01.13
  /WSFACTOR=Produkttyp 2 Polynomial Meeszeitpunkt 2 Polynomial
  /METHOD=SSTYPE(3)
  /EMMEANS=TABLES(Produkttyp)
  /PRINT=DESCRIPTIVE ETASQ
  /CRITERIA=ALPHA(.05)
  /WSDESIGN=Produkttyp Meeszeitpunkt Produkttyp*Meeszeitpunkt.
```

General Linear Model

Notes

Output Created		11-NOV-2013 15:47:19
Comments		
Input	Data	C:\Documents and Settings\Dennis Boywitt\My Documents\My Dropbox\Freiberufliche Tätigkeit\Forschungsring\Daten\Sensorik_Gruppe_1_restructured.sav
	Active Dataset	DataSet2
	Filter	<none>
	Weight	<none>
	Split File	<none>
	N of Rows in Working Data File	65
Missing Value Handling	Definition of Missing	User-defined missing values are treated as missing.
	Cases Used	Statistics are based on all cases with valid data for all variables in the model.
Syntax		GLM Sensorik2Guarani28.11.12 Sensorik2Guarani10.01.13 Sensorik2Diamant28.11.12 Sensorik2Diamant10.01.13 /WSFACTOR=Produkttyp 2 Polynomial Meeszeitpunkt 2 Polynomial /METHOD=SSTYPE(3) /EMMEANS=TABLES(Produkttyp) /PRINT=DESCRIPTIVE ETASQ /CRITERIA=ALPHA(.05) /WSDESIGN=Produkttyp Meeszeitpunkt Produkttyp*Meeszeitpunkt.
Resources	Processor Time	00:00:00,02
	Elapsed Time	00:00:00,03

```
[DataSet2] C:\Documents and Settings\Dennis Boywitt\My Documents\My Dropbox\Freiberufliche Tätigkeit\Forschungsring\Daten\Sensorik_Gruppe_1_restructured.sav
```

Within-Subjects Factors

Measure: MEASURE_1

Produkttyp	Meeszeitpunkt	Dependent Variable
1	1	Sensorik2Guarani28.11.12
	2	Sensorik2Guarani10.01.13
2	1	Sensorik2Diamant28.11.12
	2	Sensorik2Diamant10.01.13

Descriptive Statistics

	Mean	Std. Deviation	N
Sensorik2Guarani28.11.12	4,05	1,260	62
Sensorik2Guarani10.01.13	4,32	1,225	62
Sensorik2Diamant28.11.12	4,94	1,006	62
Sensorik2Diamant10.01.13	5,02	,914	62

Multivariate Tests[a]

Effect		Value	F	Hypothesis df	Error df
Produkttyp	Pillai's Trace	,321	28,888[b]	1,000	61,000
	Wilks' Lambda	,679	28,888[b]	1,000	61,000
	Hotelling's Trace	,474	28,888[b]	1,000	61,000
	Roy's Largest Root	,474	28,888[b]	1,000	61,000
Meeszeitpunkt	Pillai's Trace	,045	2,865[b]	1,000	61,000
	Wilks' Lambda	,955	2,865[b]	1,000	61,000
	Hotelling's Trace	,047	2,865[b]	1,000	61,000
	Roy's Largest Root	,047	2,865[b]	1,000	61,000
Produkttyp * Meeszeitpunkt	Pillai's Trace	,021	1,316[b]	1,000	61,000
	Wilks' Lambda	,979	1,316[b]	1,000	61,000
	Hotelling's Trace	,022	1,316[b]	1,000	61,000
	Roy's Largest Root	,022	1,316[b]	1,000	61,000

Multivariate Tests[a]

Effect		Sig.	Partial Eta Squared
Produkttyp	Pillai's Trace	,000	,321
	Wilks' Lambda	,000	,321
	Hotelling's Trace	,000	,321
	Roy's Largest Root	,000	,321
Meeszeitpunkt	Pillai's Trace	,096	,045
	Wilks' Lambda	,096	,045
	Hotelling's Trace	,096	,045
	Roy's Largest Root	,096	,045
Produkttyp * Meeszeitpunkt	Pillai's Trace	,256	,021
	Wilks' Lambda	,256	,021
	Hotelling's Trace	,256	,021
	Roy's Largest Root	,256	,021

a. Design: Intercept
Within Subjects Design: Produkttyp + Meeszeitpunkt + Produkttyp * Meeszeitpunkt

b. Exact statistic

Mauchly's Test of Sphericity[a]

Measure: MEASURE_1

Within Subjects Effect	Mauchly's W	Approx. Chi-Square	df	Sig.	Epsilon[b] Greenhouse-Geisser
Produkttyp	1,000	,000	0	.	1,000
Meeszeitpunkt	1,000	,000	0	.	1,000
Produkttyp * Meeszeitpunkt	1,000	,000	0	.	1,000

Mauchly's Test of Sphericity[a]

Measure: MEASURE_1

Within Subjects Effect	Epsilon[b]	
	Huynh-Feldt	Lower-bound
Produkttyp	1,000	1,000
Meeszeitpunkt	1,000	1,000
Produkttyp * Meeszeitpunkt	1,000	1,000

Tests the null hypothesis that the error covariance matrix of the orthonormalized transformed dependent variables is proportional to an identity matrix.

a. Design: Intercept
Within Subjects Design: Produkttyp + Meeszeitpunkt + Produkttyp * Meeszeitpunkt

b. May be used to adjust the degrees of freedom for the averaged tests of significance. Corrected tests are displayed in the Tests of Within-Subjects Effects table.

Tests of Within-Subjects Effects

Measure: MEASURE_1

Source		Type III Sum of Squares	df	Mean Square
Produkttyp	Sphericity Assumed	38,726	1	38,726
	Greenhouse-Geisser	38,726	1,000	38,726
	Huynh-Feldt	38,726	1,000	38,726
	Lower-bound	38,726	1,000	38,726
Error(Produkttyp)	Sphericity Assumed	81,774	61	1,341
	Greenhouse-Geisser	81,774	61,000	1,341
	Huynh-Feldt	81,774	61,000	1,341
	Lower-bound	81,774	61,000	1,341
Meeszeitpunkt	Sphericity Assumed	1,952	1	1,952
	Greenhouse-Geisser	1,952	1,000	1,952
	Huynh-Feldt	1,952	1,000	1,952
	Lower-bound	1,952	1,000	1,952
Error(Meeszeitpunkt)	Sphericity Assumed	41,548	61	,681
	Greenhouse-Geisser	41,548	61,000	,681
	Huynh-Feldt	41,548	61,000	,681
	Lower-bound	41,548	61,000	,681
Produkttyp * Meeszeitpunkt	Sphericity Assumed	,581	1	,581
	Greenhouse-Geisser	,581	1,000	,581
	Huynh-Feldt	,581	1,000	,581
	Lower-bound	,581	1,000	,581
Error (Produkttyp*Meeszeitpunkt)	Sphericity Assumed	26,919	61	,441
	Greenhouse-Geisser	26,919	61,000	,441
	Huynh-Feldt	26,919	61,000	,441
	Lower-bound	26,919	61,000	,441

Tests of Within-Subjects Effects

Measure: MEASURE_1

Source		F	Sig.	Partial Eta Squared
Produkttyp	Sphericity Assumed	28,888	,000	,321
	Greenhouse-Geisser	28,888	,000	,321
	Huynh-Feldt	28,888	,000	,321
	Lower-bound	28,888	,000	,321
Error(Produkttyp)	Sphericity Assumed			
	Greenhouse-Geisser			
	Huynh-Feldt			
	Lower-bound			
Meeszeitpunkt	Sphericity Assumed	2,865	,096	,045
	Greenhouse-Geisser	2,865	,096	,045
	Huynh-Feldt	2,865	,096	,045
	Lower-bound	2,865	,096	,045
Error(Meeszeitpunkt)	Sphericity Assumed			
	Greenhouse-Geisser			
	Huynh-Feldt			
	Lower-bound			
Produkttyp * Meeszeitpunkt	Sphericity Assumed	1,316	,256	,021
	Greenhouse-Geisser	1,316	,256	,021
	Huynh-Feldt	1,316	,256	,021
	Lower-bound	1,316	,256	,021
Error (Produkttyp*Meeszeitpunkt)	Sphericity Assumed			
	Greenhouse-Geisser			
	Huynh-Feldt			
	Lower-bound			

Tests of Within-Subjects Contrasts

Measure: MEASURE_1

Source	Produkttyp	Meeszeitpunkt	Type III Sum of Squares	df	Mean Square
Produkttyp	Linear		38,726	1	38,726
Error(Produkttyp)	Linear		81,774	61	1,341
Meeszeitpunkt		Linear	1,952	1	1,952
Error(Meeszeitpunkt)		Linear	41,548	61	,681
Produkttyp * Meeszeitpunkt	Linear	Linear	,581	1	,581
Error (Produkttyp*Meeszeitpunkt)	Linear	Linear	26,919	61	,441

Tests of Within-Subjects Contrasts

Measure: MEASURE_1

Source	Produkttyp	Meeszeitpunkt	F	Sig.	Partial Eta Squared
Produkttyp	Linear		28,888	,000	,321
Error(Produkttyp)	Linear				
Meeszeitpunkt		Linear	2,865	,096	,045
Error(Meeszeitpunkt)		Linear			
Produkttyp * Meeszeitpunkt	Linear	Linear	1,316	,256	,021
Error (Produkttyp*Meeszeitpunkt)	Linear	Linear			

Tests of Between-Subjects Effects

Measure: MEASURE_1

Transformed Variable: Average

Source	Type III Sum of Squares	df	Mean Square	F	Sig.	Partial Eta Squared
Intercept	5203,613	1	5203,613	2103,695	,000	,972
Error	150,887	61	2,474			

Estimated Marginal Means

Produkttyp

Measure: MEASURE_1

Produkttyp	Mean	Std. Error	95% Confidence Interval	
			Lower Bound	Upper Bound
1	4,185	,139	3,908	4,463
2	4,976	,108	4,761	5,191

```
GLM Sensorik3Guarani28.11.12 Sensroik3Guarani10.01.13
   Sensorik3Diamant28.11.12 Sensorik3Diamant10.01.13
  /WSFACTOR=Produkttyp 2 Polynomial Meeszeitpunkt 2 Polynomial
  /METHOD=SSTYPE(3)
  /EMMEANS=TABLES(Produkttyp)
  /PRINT=DESCRIPTIVE ETASQ
  /CRITERIA=ALPHA(.05)
  /WSDESIGN=Produkttyp Meeszeitpunkt Produkttyp*Meeszeitpunkt.
```

General Linear Model

Notes

Output Created		11-NOV-2013 15:50:38
Comments		
Input	Data	C:\Documents and Settings\Dennis Boywitt\My Documents\My Dropbox\Freiberufliche Tätigkeit\Forschungsring\Daten\Sensorik_Gruppe_1_restructured.sav
	Active Dataset	DataSet2
	Filter	<none>
	Weight	<none>
	Split File	<none>
	N of Rows in Working Data File	65
Missing Value Handling	Definition of Missing	User-defined missing values are treated as missing.
	Cases Used	Statistics are based on all cases with valid data for all variables in the model.
Syntax		GLM Sensorik3Guarani28.11.12 Sensroik3Guarani10.01.13 Sensorik3Diamant28.11.12 Sensorik3Diamant10.01.13 /WSFACTOR=Produkttyp 2 Polynomial Meeszeitpunkt 2 Polynomial /METHOD=SSTYPE(3) /EMMEANS=TABLES(Produkttyp) /PRINT=DESCRIPTIVE ETASQ /CRITERIA=ALPHA(.05) /WSDESIGN=Produkttyp Meeszeitpunkt Produkttyp*Meeszeitpunkt.
Resources	Processor Time	00:00:00,03
	Elapsed Time	00:00:00,03

```
[DataSet2] C:\Documents and Settings\Dennis Boywitt\My Documents\My Dropbox\Freiberufliche Tätigkeit\Forschungsring\Daten\Sensorik_Gruppe_1_restructured.sav
```

Within-Subjects Factors

Measure: MEASURE_1

Produkttyp	Meeszeitpunkt	Dependent Variable
1	1	Sensorik3Guarani28.11.12
	2	Sensroik3Guarani10.01.13
2	1	Sensorik3Diamant28.11.12
	2	Sensorik3Diamant10.01.13

Descriptive Statistics

	Mean	Std. Deviation	N
Sensorik3Guarani28.11.12	4,85	1,329	62
Sensroik3Guarani10.01.13	5,10	1,067	62
Sensorik3Diamant28.11.12	5,39	,797	62
Sensorik3Diamant10.01.13	5,42	,821	62

Multivariate Tests[a]

Effect		Value	F	Hypothesis df	Error df
Produkttyp	Pillai's Trace	,129	9,041[b]	1,000	61,000
	Wilks' Lambda	,871	9,041[b]	1,000	61,000
	Hotelling's Trace	,148	9,041[b]	1,000	61,000
	Roy's Largest Root	,148	9,041[b]	1,000	61,000
Meeszeitpunkt	Pillai's Trace	,042	2,674[b]	1,000	61,000
	Wilks' Lambda	,958	2,674[b]	1,000	61,000
	Hotelling's Trace	,044	2,674[b]	1,000	61,000
	Roy's Largest Root	,044	2,674[b]	1,000	61,000
Produkttyp * Meeszeitpunkt	Pillai's Trace	,025	1,536[b]	1,000	61,000
	Wilks' Lambda	,975	1,536[b]	1,000	61,000
	Hotelling's Trace	,025	1,536[b]	1,000	61,000
	Roy's Largest Root	,025	1,536[b]	1,000	61,000

Multivariate Tests[a]

Effect		Sig.	Partial Eta Squared
Produkttyp	Pillai's Trace	,004	,129
	Wilks' Lambda	,004	,129
	Hotelling's Trace	,004	,129
	Roy's Largest Root	,004	,129
Meeszeitpunkt	Pillai's Trace	,107	,042
	Wilks' Lambda	,107	,042
	Hotelling's Trace	,107	,042
	Roy's Largest Root	,107	,042
Produkttyp * Meeszeitpunkt	Pillai's Trace	,220	,025
	Wilks' Lambda	,220	,025
	Hotelling's Trace	,220	,025
	Roy's Largest Root	,220	,025

a. Design: Intercept
Within Subjects Design: Produkttyp + Meeszeitpunkt + Produkttyp * Meeszeitpunkt

b. Exact statistic

Mauchly's Test of Sphericity[a]

Measure: MEASURE_1

Within Subjects Effect	Mauchly's W	Approx. Chi-Square	df	Sig.	Epsilon[b]
					Greenhouse-Geisser
Produkttyp	1,000	,000	0	.	1,000
Meeszeitpunkt	1,000	,000	0	.	1,000
Produkttyp * Meeszeitpunkt	1,000	,000	0	.	1,000

Mauchly's Test of Sphericity[a]

Measure: MEASURE_1

Within Subjects Effect	Epsilon[b]	
	Huynh-Feldt	Lower-bound
Produkttyp	1,000	1,000
Meeszeitpunkt	1,000	1,000
Produkttyp * Meeszeitpunkt	1,000	1,000

Tests the null hypothesis that the error covariance matrix of the orthonormalized transformed dependent variables is proportional to an identity matrix.

a. Design: Intercept
Within Subjects Design: Produkttyp + Meeszeitpunkt + Produkttyp * Meeszeitpunkt

b. May be used to adjust the degrees of freedom for the averaged tests of significance. Corrected tests are displayed in the Tests of Within-Subjects Effects table.

Tests of Within-Subjects Effects

Measure: MEASURE_1

Source		Type III Sum of Squares	df	Mean Square
Produkttyp	Sphericity Assumed	11,327	1	11,327
	Greenhouse-Geisser	11,327	1,000	11,327
	Huynh-Feldt	11,327	1,000	11,327
	Lower-bound	11,327	1,000	11,327
Error(Produkttyp)	Sphericity Assumed	76,423	61	1,253
	Greenhouse-Geisser	76,423	61,000	1,253
	Huynh-Feldt	76,423	61,000	1,253
	Lower-bound	76,423	61,000	1,253
Meeszeitpunkt	Sphericity Assumed	1,165	1	1,165
	Greenhouse-Geisser	1,165	1,000	1,165
	Huynh-Feldt	1,165	1,000	1,165
	Lower-bound	1,165	1,000	1,165
Error(Meeszeitpunkt)	Sphericity Assumed	26,585	61	,436
	Greenhouse-Geisser	26,585	61,000	,436
	Huynh-Feldt	26,585	61,000	,436
	Lower-bound	26,585	61,000	,436
Produkttyp * Meeszeitpunkt	Sphericity Assumed	,681	1	,681
	Greenhouse-Geisser	,681	1,000	,681
	Huynh-Feldt	,681	1,000	,681
	Lower-bound	,681	1,000	,681
Error (Produkttyp*Meeszeitpunkt)	Sphericity Assumed	27,069	61	,444
	Greenhouse-Geisser	27,069	61,000	,444
	Huynh-Feldt	27,069	61,000	,444
	Lower-bound	27,069	61,000	,444

Tests of Within-Subjects Effects

Measure: MEASURE_1

Source		F	Sig.	Partial Eta Squared
Produkttyp	Sphericity Assumed	9,041	,004	,129
	Greenhouse-Geisser	9,041	,004	,129
	Huynh-Feldt	9,041	,004	,129
	Lower-bound	9,041	,004	,129
Error(Produkttyp)	Sphericity Assumed			
	Greenhouse-Geisser			
	Huynh-Feldt			
	Lower-bound			
Meeszeitpunkt	Sphericity Assumed	2,674	,107	,042
	Greenhouse-Geisser	2,674	,107	,042
	Huynh-Feldt	2,674	,107	,042
	Lower-bound	2,674	,107	,042
Error(Meeszeitpunkt)	Sphericity Assumed			
	Greenhouse-Geisser			
	Huynh-Feldt			
	Lower-bound			
Produkttyp * Meeszeitpunkt	Sphericity Assumed	1,536	,220	,025
	Greenhouse-Geisser	1,536	,220	,025
	Huynh-Feldt	1,536	,220	,025
	Lower-bound	1,536	,220	,025
Error (Produkttyp*Meeszeitpunkt)	Sphericity Assumed			
	Greenhouse-Geisser			
	Huynh-Feldt			
	Lower-bound			

Tests of Within-Subjects Contrasts

Measure: MEASURE_1

Source	Produkttyp	Meeszeitpunkt	Type III Sum of Squares	df	Mean Square
Produkttyp	Linear		11,327	1	11,327
Error(Produkttyp)	Linear		76,423	61	1,253
Meeszeitpunkt		Linear	1,165	1	1,165
Error(Meeszeitpunkt)		Linear	26,585	61	,436
Produkttyp * Meeszeitpunkt	Linear	Linear	,681	1	,681
Error (Produkttyp*Meeszeitpunkt)	Linear	Linear	27,069	61	,444

Tests of Within-Subjects Contrasts

Measure: MEASURE_1

Source	Produkttyp	Meeszeitpunkt	F	Sig.	Partial Eta Squared
Produkttyp	Linear		9,041	,004	,129
Error(Produkttyp)	Linear				
Meeszeitpunkt		Linear	2,674	,107	,042
Error(Meeszeitpunkt)		Linear			
Produkttyp * Meeszeitpunkt	Linear	Linear	1,536	,220	,025
Error (Produkttyp*Meeszeitpunkt)	Linear	Linear			

Tests of Between-Subjects Effects

Measure: MEASURE_1

Transformed Variable: Average

Source	Type III Sum of Squares	df	Mean Square	F	Sig.	Partial Eta Squared
Intercept	6678,907	1	6678,907	3211,956	,000	,981
Error	126,843	61	2,079			

Estimated Marginal Means

Produkttyp

Measure: MEASURE_1

Produkttyp	Mean	Std. Error	95% Confidence Interval	
			Lower Bound	Upper Bound
1	4,976	,141	4,693	5,259
2	5,403	,083	5,238	5,569

```
GLM Sensorik4Guarani28.11.12 Sensorik4Guarani10.01.13
   Sensorik4Diamant28.11.12 Sensorik4Diamant10.01.13
 /WSFACTOR=Produkttyp 2 Polynomial Meeszeitpunkt 2 Polynomial
 /METHOD=SSTYPE(3)
 /EMMEANS=TABLES(Produkttyp)
 /PRINT=DESCRIPTIVE ETASQ
 /CRITERIA=ALPHA(.05)
 /WSDESIGN=Produkttyp Meeszeitpunkt Produkttyp*Meeszeitpunkt.
```

General Linear Model

Notes

Output Created		11-NOV-2013 15:51:56
Comments		
Input	Data	C:\Documents and Settings\Dennis Boywitt\My Documents\My Dropbox\Freiberufliche Tätigkeit\Forschungsring\Daten\Sensorik_Gruppe_1_restructured.sav
	Active Dataset	DataSet2
	Filter	<none>
	Weight	<none>
	Split File	<none>
	N of Rows in Working Data File	65
Missing Value Handling	Definition of Missing	User-defined missing values are treated as missing.
	Cases Used	Statistics are based on all cases with valid data for all variables in the model.
Syntax		GLM Sensorik4Guarani28.11.12 Sensorik4Guarani10.01.13 Sensorik4Diamant28.11.12 Sensorik4Diamant10.01.13 /WSFACTOR=Produkttyp 2 Polynomial Meeszeitpunkt 2 Polynomial /METHOD=SSTYPE(3) /EMMEANS=TABLES(Produkttyp) /PRINT=DESCRIPTIVE ETASQ /CRITERIA=ALPHA(.05) /WSDESIGN=Produkttyp Meeszeitpunkt Produkttyp*Meeszeitpunkt.
Resources	Processor Time	00:00:00,03
	Elapsed Time	00:00:00,03

[DataSet2] C:\Documents and Settings\Dennis Boywitt\My Documents\My Dropbox\Freiberufliche Tätigkeit\Forschungsring\Daten\Sensorik_Gruppe_1_restructured.sav

Within-Subjects Factors

Measure: MEASURE_1

Produkttyp	Meeszeitpunkt	Dependent Variable
1	1	Sensorik4Guarani28.11.12
	2	Sensorik4Guarani10.01.13
2	1	Sensorik4Diamant28.11.12
	2	Sensorik4Diamant10.01.13

Descriptive Statistics

	Mean	Std. Deviation	N
Sensorik4Guarani28.11.12	4,92	1,232	62
Sensorik4Guarani10.01.13	4,98	1,123	62
Sensorik4Diamant28.11.12	5,31	,879	62
Sensorik4Diamant10.01.13	5,24	,803	62

Multivariate Tests[a]

Effect		Value	F	Hypothesis df	Error df
Produkttyp	Pillai's Trace	,080	5,315[b]	1,000	61,000
	Wilks' Lambda	,920	5,315[b]	1,000	61,000
	Hotelling's Trace	,087	5,315[b]	1,000	61,000
	Roy's Largest Root	,087	5,315[b]	1,000	61,000
Meeszeitpunkt	Pillai's Trace	,000	,000[b]	1,000	61,000
	Wilks' Lambda	1,000	,000[b]	1,000	61,000
	Hotelling's Trace	,000	,000[b]	1,000	61,000
	Roy's Largest Root	,000	,000[b]	1,000	61,000
Produkttyp * Meeszeitpunkt	Pillai's Trace	,006	,356[b]	1,000	61,000
	Wilks' Lambda	,994	,356[b]	1,000	61,000
	Hotelling's Trace	,006	,356[b]	1,000	61,000
	Roy's Largest Root	,006	,356[b]	1,000	61,000

Multivariate Tests[a]

Effect		Sig.	Partial Eta Squared
Produkttyp	Pillai's Trace	,025	,080
	Wilks' Lambda	,025	,080
	Hotelling's Trace	,025	,080
	Roy's Largest Root	,025	,080
Meeszeitpunkt	Pillai's Trace	1,000	,000
	Wilks' Lambda	1,000	,000
	Hotelling's Trace	1,000	,000
	Roy's Largest Root	1,000	,000
Produkttyp * Meeszeitpunkt	Pillai's Trace	,553	,006
	Wilks' Lambda	,553	,006
	Hotelling's Trace	,553	,006
	Roy's Largest Root	,553	,006

a. Design: Intercept
Within Subjects Design: Produkttyp + Meeszeitpunkt + Produkttyp * Meeszeitpunkt

b. Exact statistic

Mauchly's Test of Sphericity[a]

Measure: MEASURE_1

Within Subjects Effect	Mauchly's W	Approx. Chi-Square	df	Sig.	Epsilon[b]
					Greenhouse-Geisser
Produkttyp	1,000	,000	0	.	1,000
Meeszeitpunkt	1,000	,000	0	.	1,000
Produkttyp * Meeszeitpunkt	1,000	,000	0	.	1,000

Mauchly's Test of Sphericity[a]

Measure: MEASURE_1

Within Subjects Effect	Epsilon[b]	
	Huynh-Feldt	Lower-bound
Produkttyp	1,000	1,000
Meeszeitpunkt	1,000	1,000
Produkttyp * Meeszeitpunkt	1,000	1,000

Tests the null hypothesis that the error covariance matrix of the orthonormalized transformed dependent variables is proportional to an identity matrix.

a. Design: Intercept
Within Subjects Design: Produkttyp + Meeszeitpunkt + Produkttyp * Meeszeitpunkt

b. May be used to adjust the degrees of freedom for the averaged tests of significance. Corrected tests are displayed in the Tests of Within-Subjects Effects table.

Tests of Within-Subjects Effects

Measure: MEASURE_1

Source		Type III Sum of Squares	df	Mean Square
Produkttyp	Sphericity Assumed	6,452	1	6,452
	Greenhouse-Geisser	6,452	1,000	6,452
	Huynh-Feldt	6,452	1,000	6,452
	Lower-bound	6,452	1,000	6,452
Error(Produkttyp)	Sphericity Assumed	74,048	61	1,214
	Greenhouse-Geisser	74,048	61,000	1,214
	Huynh-Feldt	74,048	61,000	1,214
	Lower-bound	74,048	61,000	1,214
Meeszeitpunkt	Sphericity Assumed	,000	1	,000
	Greenhouse-Geisser	,000	1,000	,000
	Huynh-Feldt	,000	1,000	,000
	Lower-bound	,000	1,000	,000
Error(Meeszeitpunkt)	Sphericity Assumed	33,500	61	,549
	Greenhouse-Geisser	33,500	61,000	,549
	Huynh-Feldt	33,500	61,000	,549
	Lower-bound	33,500	61,000	,549
Produkttyp * Meeszeitpunkt	Sphericity Assumed	,258	1	,258
	Greenhouse-Geisser	,258	1,000	,258
	Huynh-Feldt	,258	1,000	,258
	Lower-bound	,258	1,000	,258
Error (Produkttyp*Meeszeitpunkt)	Sphericity Assumed	44,242	61	,725
	Greenhouse-Geisser	44,242	61,000	,725
	Huynh-Feldt	44,242	61,000	,725
	Lower-bound	44,242	61,000	,725

Tests of Within-Subjects Effects

Measure: MEASURE_1

Source		F	Sig.	Partial Eta Squared
Produkttyp	Sphericity Assumed	5,315	,025	,080
	Greenhouse-Geisser	5,315	,025	,080
	Huynh-Feldt	5,315	,025	,080
	Lower-bound	5,315	,025	,080
Error(Produkttyp)	Sphericity Assumed			
	Greenhouse-Geisser			
	Huynh-Feldt			
	Lower-bound			
Meeszeitpunkt	Sphericity Assumed	,000	1,000	,000
	Greenhouse-Geisser	,000	1,000	,000
	Huynh-Feldt	,000	1,000	,000
	Lower-bound	,000	1,000	,000
Error(Meeszeitpunkt)	Sphericity Assumed			
	Greenhouse-Geisser			
	Huynh-Feldt			
	Lower-bound			
Produkttyp * Meeszeitpunkt	Sphericity Assumed	,356	,553	,006
	Greenhouse-Geisser	,356	,553	,006
	Huynh-Feldt	,356	,553	,006
	Lower-bound	,356	,553	,006
Error (Produkttyp*Meeszeitpunkt)	Sphericity Assumed			
	Greenhouse-Geisser			
	Huynh-Feldt			
	Lower-bound			

Tests of Within-Subjects Contrasts

Measure: MEASURE_1

Source	Produkttyp	Meeszeitpunkt	Type III Sum of Squares	df	Mean Square
Produkttyp	Linear		6,452	1	6,452
Error(Produkttyp)	Linear		74,048	61	1,214
Meeszeitpunkt		Linear	,000	1	,000
Error(Meeszeitpunkt)		Linear	33,500	61	,549
Produkttyp * Meeszeitpunkt	Linear	Linear	,258	1	,258
Error (Produkttyp*Meeszeitpunkt)	Linear	Linear	44,242	61	,725

Tests of Within-Subjects Contrasts

Measure: MEASURE_1

Source	Produkttyp	Meeszeitpunkt	F	Sig.	Partial Eta Squared
Produkttyp	Linear		5,315	,025	,080
Error(Produkttyp)	Linear				
Meeszeitpunkt		Linear	,000	1,000	,000
Error(Meeszeitpunkt)		Linear			
Produkttyp * Meeszeitpunkt	Linear	Linear	,356	,553	,006
Error (Produkttyp*Meeszeitpunk t)	Linear	Linear			

Tests of Between-Subjects Effects

Measure: MEASURE_1

Transformed Variable: Average

Source	Type III Sum of Squares	df	Mean Square	F	Sig.	Partial Eta Squared
Intercept	6483,161	1	6483,161	3790,279	,000	,984
Error	104,339	61	1,710			

Estimated Marginal Means

Produkttyp

Measure: MEASURE_1

Produkttyp	Mean	Std. Error	95% Confidence Interval	
			Lower Bound	Upper Bound
1	4,952	,126	4,699	5,204
2	5,274	,087	5,100	5,449

```
GLM Sensorik5Guarani28.11.12 Sensorik5Guarani10.01.13
   Sensorik5Diamant28.11.12 Sensorik5Diamant10.01.13
 /WSFACTOR=Produkttyp 2 Polynomial Meeszeitpunkt 2 Polynomial
 /METHOD=SSTYPE(3)
 /EMMEANS=TABLES(Produkttyp)
 /PRINT=DESCRIPTIVE ETASQ
 /CRITERIA=ALPHA(.05)
 /WSDESIGN=Produkttyp Meeszeitpunkt Produkttyp*Meeszeitpunkt.
```

General Linear Model

Notes

Output Created		11-NOV-2013 15:53:33
Comments		
Input	Data	C:\Documents and Settings\Dennis Boywitt\My Documents\My Dropbox\Freiberufliche Tätigkeit\Forschungsring\Daten\Sensorik_Gruppe_1_restructured.sav
	Active Dataset	DataSet2
	Filter	<none>
	Weight	<none>
	Split File	<none>
	N of Rows in Working Data File	65
Missing Value Handling	Definition of Missing	User-defined missing values are treated as missing.
	Cases Used	Statistics are based on all cases with valid data for all variables in the model.
Syntax		GLM Sensorik5Guarani28.11.12 Sensorik5Guarani10.01.13 Sensorik5Diamant28.11.12 Sensorik5Diamant10.01.13 /WSFACTOR=Produkttyp 2 Polynomial Meeszeitpunkt 2 Polynomial /METHOD=SSTYPE(3) /EMMEANS=TABLES(Produkttyp) /PRINT=DESCRIPTIVE ETASQ /CRITERIA=ALPHA(.05) /WSDESIGN=Produkttyp Meeszeitpunkt Produkttyp*Meeszeitpunkt.
Resources	Processor Time	00:00:00,03
	Elapsed Time	00:00:00,03

```
[DataSet2] C:\Documents and Settings\Dennis Boywitt\My Documents\My Dropbox\Freiberufliche Tätigkeit\Forschungsring\Daten\Sensorik_Gruppe_1_restructured.sav
```

Within-Subjects Factors

Measure: MEASURE_1

Produkttyp	Meeszeitpunkt	Dependent Variable
1	1	Sensorik5Guarani28.11.12
	2	Sensorik5Guarani10.01.13
2	1	Sensorik5Diamant28.11.12
	2	Sensorik5Diamant10.01.13

Descriptive Statistics

	Mean	Std. Deviation	N
Sensorik5Guarani28.11.12	4,65	1,147	62
Sensorik5Guarani10.01.13	4,69	1,080	62
Sensorik5Diamant28.11.12	5,40	,689	62
Sensorik5Diamant10.01.13	5,34	,745	62

Multivariate Tests[a]

Effect		Value	F	Hypothesis df	Error df
Produkttyp	Pillai's Trace	,312	27,692[b]	1,000	61,000
	Wilks' Lambda	,688	27,692[b]	1,000	61,000
	Hotelling's Trace	,454	27,692[b]	1,000	61,000
	Roy's Largest Root	,454	27,692[b]	1,000	61,000
Meeszeitpunkt	Pillai's Trace	,000	,011[b]	1,000	61,000
	Wilks' Lambda	1,000	,011[b]	1,000	61,000
	Hotelling's Trace	,000	,011[b]	1,000	61,000
	Roy's Largest Root	,000	,011[b]	1,000	61,000
Produkttyp * Meeszeitpunkt	Pillai's Trace	,008	,472[b]	1,000	61,000
	Wilks' Lambda	,992	,472[b]	1,000	61,000
	Hotelling's Trace	,008	,472[b]	1,000	61,000
	Roy's Largest Root	,008	,472[b]	1,000	61,000

Multivariate Tests[a]

Effect		Sig.	Partial Eta Squared
Produkttyp	Pillai's Trace	,000	,312
	Wilks' Lambda	,000	,312
	Hotelling's Trace	,000	,312
	Roy's Largest Root	,000	,312
Meeszeitpunkt	Pillai's Trace	,918	,000
	Wilks' Lambda	,918	,000
	Hotelling's Trace	,918	,000
	Roy's Largest Root	,918	,000
Produkttyp * Meeszeitpunkt	Pillai's Trace	,495	,008
	Wilks' Lambda	,495	,008
	Hotelling's Trace	,495	,008
	Roy's Largest Root	,495	,008

a. Design: Intercept
Within Subjects Design: Produkttyp + Meeszeitpunkt + Produkttyp * Meeszeitpunkt

b. Exact statistic

Mauchly's Test of Sphericity[a]

Measure: MEASURE_1

Within Subjects Effect	Mauchly's W	Approx. Chi-Square	df	Sig.	Epsilon[b]
					Greenhouse-Geisser
Produkttyp	1,000	,000	0	.	1,000
Meeszeitpunkt	1,000	,000	0	.	1,000
Produkttyp * Meeszeitpunkt	1,000	,000	0	.	1,000

Mauchly's Test of Sphericity[a]

Measure: MEASURE_1

Within Subjects Effect	Epsilon[b]	
	Huynh-Feldt	Lower-bound
Produkttyp	1,000	1,000
Meeszeitpunkt	1,000	1,000
Produkttyp * Meeszeitpunkt	1,000	1,000

Tests the null hypothesis that the error covariance matrix of the orthonormalized transformed dependent variables is proportional to an identity matrix.

a. Design: Intercept
Within Subjects Design: Produkttyp + Meeszeitpunkt + Produkttyp * Meeszeitpunkt

b. May be used to adjust the degrees of freedom for the averaged tests of significance. Corrected tests are displayed in the Tests of Within-Subjects Effects table.

Tests of Within-Subjects Effects

Measure: MEASURE_1

Source		Type III Sum of Squares	df	Mean Square
Produkttyp	Sphericity Assumed	30,520	1	30,520
	Greenhouse-Geisser	30,520	1,000	30,520
	Huynh-Feldt	30,520	1,000	30,520
	Lower-bound	30,520	1,000	30,520
Error(Produkttyp)	Sphericity Assumed	67,230	61	1,102
	Greenhouse-Geisser	67,230	61,000	1,102
	Huynh-Feldt	67,230	61,000	1,102
	Lower-bound	67,230	61,000	1,102
Meeszeitpunkt	Sphericity Assumed	,004	1	,004
	Greenhouse-Geisser	,004	1,000	,004
	Huynh-Feldt	,004	1,000	,004
	Lower-bound	,004	1,000	,004
Error(Meeszeitpunkt)	Sphericity Assumed	22,746	61	,373
	Greenhouse-Geisser	22,746	61,000	,373
	Huynh-Feldt	22,746	61,000	,373
	Lower-bound	22,746	61,000	,373
Produkttyp * Meeszeitpunkt	Sphericity Assumed	,198	1	,198
	Greenhouse-Geisser	,198	1,000	,198
	Huynh-Feldt	,198	1,000	,198
	Lower-bound	,198	1,000	,198
Error (Produkttyp*Meeszeitpunkt)	Sphericity Assumed	25,552	61	,419
	Greenhouse-Geisser	25,552	61,000	,419
	Huynh-Feldt	25,552	61,000	,419
	Lower-bound	25,552	61,000	,419

Tests of Within-Subjects Effects

Measure: MEASURE_1

Source		F	Sig.	Partial Eta Squared
Produkttyp	Sphericity Assumed	27,692	,000	,312
	Greenhouse-Geisser	27,692	,000	,312
	Huynh-Feldt	27,692	,000	,312
	Lower-bound	27,692	,000	,312
Error(Produkttyp)	Sphericity Assumed			
	Greenhouse-Geisser			
	Huynh-Feldt			
	Lower-bound			
Meeszeitpunkt	Sphericity Assumed	,011	,918	,000
	Greenhouse-Geisser	,011	,918	,000
	Huynh-Feldt	,011	,918	,000
	Lower-bound	,011	,918	,000
Error(Meeszeitpunkt)	Sphericity Assumed			
	Greenhouse-Geisser			
	Huynh-Feldt			
	Lower-bound			
Produkttyp * Meeszeitpunkt	Sphericity Assumed	,472	,495	,008
	Greenhouse-Geisser	,472	,495	,008
	Huynh-Feldt	,472	,495	,008
	Lower-bound	,472	,495	,008
Error (Produkttyp*Meeszeitpunkt)	Sphericity Assumed			
	Greenhouse-Geisser			
	Huynh-Feldt			
	Lower-bound			

Tests of Within-Subjects Contrasts

Measure: MEASURE_1

Source	Produkttyp	Meeszeitpunkt	Type III Sum of Squares	df	Mean Square
Produkttyp	Linear		30,520	1	30,520
Error(Produkttyp)	Linear		67,230	61	1,102
Meeszeitpunkt		Linear	,004	1	,004
Error(Meeszeitpunkt)		Linear	22,746	61	,373
Produkttyp * Meeszeitpunkt	Linear	Linear	,198	1	,198
Error (Produkttyp*Meeszeitpunkt)	Linear	Linear	25,552	61	,419

Tests of Within-Subjects Contrasts

Measure: MEASURE_1

Source	Produkttyp	Meeszeitpunkt	F	Sig.	Partial Eta Squared
Produkttyp	Linear		27,692	,000	,312
Error(Produkttyp)	Linear				
Meeszeitpunkt		Linear	,011	,918	,000
Error(Meeszeitpunkt)		Linear			
Produkttyp * Meeszeitpunkt	Linear	Linear	,472	,495	,008
Error (Produkttyp*Meeszeitpunkt)	Linear	Linear			

Tests of Between-Subjects Effects

Measure: MEASURE_1

Transformed Variable: Average

Source	Type III Sum of Squares	df	Mean Square	F	Sig.	Partial Eta Squared
Intercept	6250,101	1	6250,101	3864,767	,000	,984
Error	98,649	61	1,617			

Estimated Marginal Means

Produkttyp

Measure: MEASURE_1

Produkttyp	Mean	Std. Error	95% Confidence Interval	
			Lower Bound	Upper Bound
1	4,669	,126	4,418	4,921
2	5,371	,078	5,215	5,527
